# Consequences of Amide Connectivity in the Supramolecular Polymerization of Porphyrins: Spectroscopic Observations Rationalized by Theoretical Modelling

**DOI:** 10.1002/chem.202101036

**Published:** 2021-05-27

**Authors:** Elisabeth Weyandt, Ivo A. W. Filot, Ghislaine Vantomme, E. W. Meijer

**Affiliations:** ^1^ Laboratory of Macromolecular and Organic Chemistry Eindhoven University of Technology P. O. Box 513 5600 MB Eindhoven The Netherlands; ^2^ Institute for Complex Molecular Systems Eindhoven University of Technology P. O. Box 513 5600 MB Eindhoven The Netherlands; ^3^ Schuit Institute for Catalysis Eindhoven University of Technology P. O. Box 513 5600 MB Eindhoven The Netherlands

**Keywords:** amide connectivity, copolymerizations, porphyrins, supramolecular polymers

## Abstract

The correlation between molecular structure and mechanism of supramolecular polymerizations is a topic of great interest, with a special focus on the pathway complexity of porphyrin assemblies. Their cooperative polymerization typically yields highly ordered, long 1D polymers and is driven by a combination of π‐stacking due to solvophobic effects and hydrogen bonding interactions. Subtle changes in molecular structure, however, have significant influence on the cooperativity factor and yield different aggregate types (J‐ versus H‐aggregates) of different lengths. In this study, the influence of amide connectivity on the self‐assembly behavior of porphyrin‐based supramolecular monomers was investigated. While in nonpolar solvents, C=O centered monomers readily assemble into helical supramolecular polymers via a cooperative mechanism, their NH centered counterparts form short, non‐helical J‐type aggregates via an isodesmic pathway. A combination of spectroscopy and density functional theory modelling sheds light on the molecular origins causing this stunning difference in assembly properties and demonstrates the importance of molecular connectivity in the design of supramolecular systems. Finally, their mutual interference in copolymerization experiments is presented.

## Graphical abstract

**Amide connectivity** in the molecular design of porphyrin supramolecular monomers strongly impacts the assembly behavior in nonpolar solvents. While C=O centered monomers aggregate cooperatively into long, 1D supramolecular fibers, their NH‐centered counterparts only form isodesmic J‐aggregates. With a combination of spectroscopy and density functional modelling, the authors shed light on the molecular origins of these differences in properties.

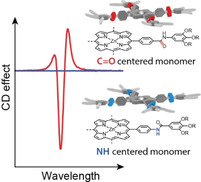

## Introduction

Porphyrin‐based supramolecular building blocks with their planar and aromatic macrocyclic structure are ubiquitous in the natural realm. They serve as coordination sites for molecular oxygen and carbon dioxide during cellular respiration,^[1]^ they play a vital role as light harvesting systems in the chlorophyll photosystem,[^2^, ^3^] and their metal binding abilities contribute to the catalytic activity of many enzymes.[^4^, ^5^] Due to their large conjugated π‐system, they exhibit strong absorption bands in the visible region of light and are usually deeply colored, which also led to their name deriving from the Greek word πορϕύρα (*porphyra*) for purple.^[6]^ Despite their fascinating photo‐optical properties, porphyrin‐based monomers often exhibit promiscuous assembly behavior in supramolecular polymerizations, leading to multiple aggregate types and aggregation pathways.[^7^, ^8^, ^9^] This pathway complexity is one of the reasons that porphyrin‐based systems have been crucial in the establishment of advanced polymerization techniques, such as living supramolecular polymerization[^10^, ^11^] or seeded, kinetically controlled polymerization methods.[^12^, ^13^, ^14^, ^15^]

**Figure 1:**
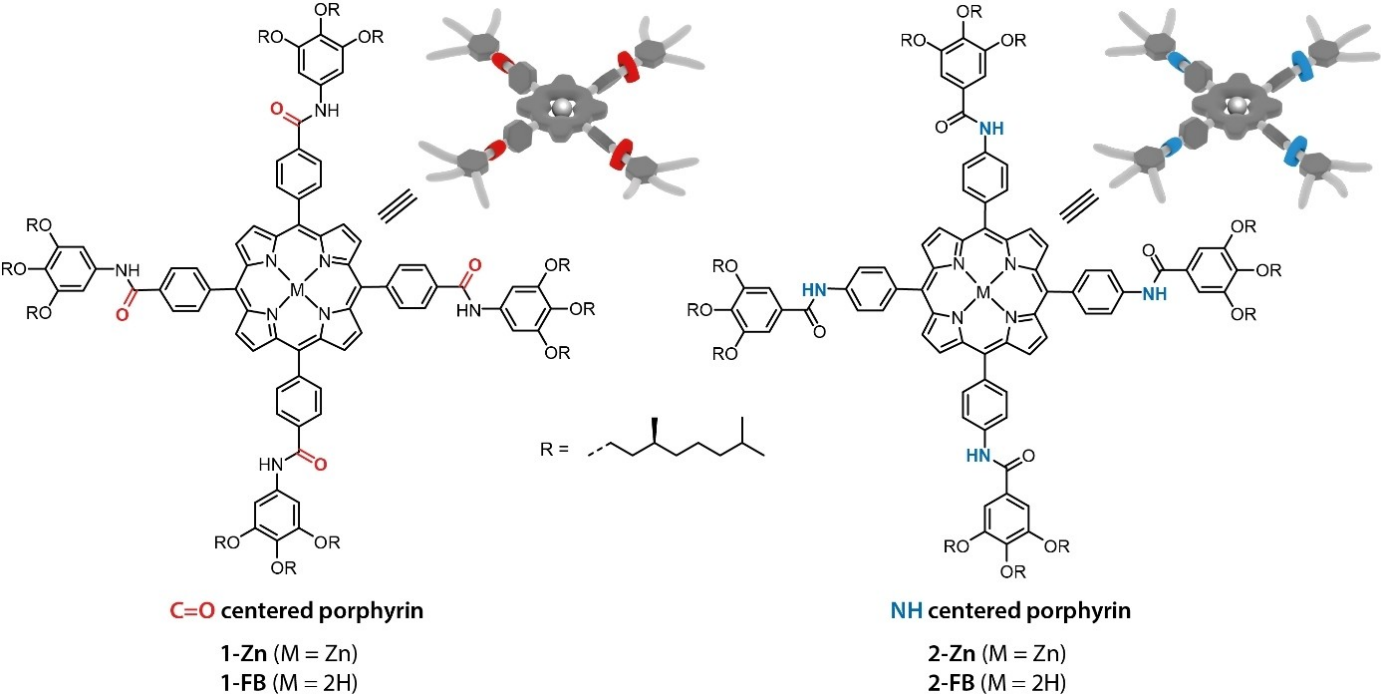
Molecular structures and cartoon representations of porphyrin monomers with the porphyrin core connected to either the carbonyl (**1**, C=O centered) or to the amine (**2**, NH centered) part of the amide group. The monomers were either investigated as the free base derivatives (M=2H) or with zinc inserted into the ligand (M=Zn).

The strong drive of porphyrins to aggregate arises from π‐stacking due to the solvophobic effect between the highly conjugated cores and the solvent, while ordering is attributed to other supramolecular interactions such as hydrogen bonding and dipole‐dipole interactions. While many studies have been conducted to investigate the influence of changes in molecular structure on the photo‐physical properties of porphyrins,[^16^, ^17^] much less is understood about the resulting aggregation behavior and the presence or absence of cooperativity.[^18^, ^19^, ^20^, ^21^] For many porphyrin‐based supramolecular systems, highly ordered H‐aggregates (supramolecular polymers) assemble via a cooperative or nucleation‐elongation mechanism, while small J‐aggregates form in competition to the H‐aggregates by an isodesmic mechanism. Depending on the molecular design, not only the mechanism of polymerization can be tuned, but also the resulting supramolecular architectures, ranging from ladder type structures through coordination bonds[^22^, ^23^, ^24^] to 1D supramolecular polymers via hydrogen bonding along the aggregate backbone.[^25^, ^26^, ^27^] In the past, we studied the supramolecular polymerization of chiral porphyrin **1‐Zn** (Figure 1) in apolar solvents. Under thermodynamically controlled conditions, **1‐Zn** polymerizes into helical and optically active supramolecular polymers.^[8]^ However, small changes in solvent or structure leads to smaller J‐aggregates that are either the thermodynamically most stable structure or a kinetically trapped state.[^7^, ^9^]

These results prompted us to have a closer look by a simple modification in molecular design, namely changing the amide connectivity from C=O centered to N−H centered amides. Other studies showed that reversing the amide connectivity usually leads to a change in conjugation of the amide with the core, which will influence the strength of the hydrogen bonding interactions between the monomers as well as the torsional angle between the amide and the core. For benzene‐1,3,5‐tricarboxamides (BTAs), the change in amide connectivity leads to a smaller cooperativity factor (σ) for the *N*‐centered compared to the *C*‐centered monomers, caused by a higher rotational penalty of the amide bond and weaker dipole‐dipole interactions.[^28^, ^29^, ^30^, ^31^] A similarly higher cooperativity factor for *C*‐centered monomers of *C*_2_‐ and *C*_3_‐symmetrical oligo(phenyleneethynylene)s (OPEs) was found when the amides were attached to an aromatic ring due to differences in hydrogen bond length and nucleus preorganization.^[32]^ When the substituents on the *C*_3_‐symmetric OPEs were of aliphatic nature however, this trend was reversed and the *N*‐centered derivative exhibited higher cooperativity.^[33]^

In this study, we compare the self‐assembly properties of two porphyrin monomers with either C=O or N−H centered amides connected to the porphyrin core: **1‐Zn** and **2‐Zn** respectively. This seemingly small change in molecular design has substantial influence on the photo‐optical properties and the assembly process. While **1‐Zn** forms long 1‐D supramolecular H‐aggregates via a nucleation‐elongation mechanism, **2‐Zn** forms preferably achiral J‐aggregates via an isodesmic pathway. With density functional theory (DFT) calculations using models of the free base ligands **1‐FB** and **2‐FB**, we rationalize the molecular origins of these differences. Lastly, when mixing the two monomers we observe mixed aggregate formation and a concentration independent destabilization of the H‐aggregates formed by **1‐Zn** with increasing ratios of **2‐Zn**.

## Results and Discussion

### Molecular design, synthesis and characterization

To elaborate on the influence of amide connectivity on the self‐assembly of porphyrin based supramolecular polymers, we compared two monomers bearing either four C=O centered (**1**, Figure 1) or four NH centered amide groups on the porphyrin core (**2**). For this study we compared both the free base porphyrin monomers (**1‐** and **2‐FB**, M=2H) and zinc centered monomers (**1‐** and **2‐Zn**, M=Zn). The porphyrin monomers were equipped with chiral gallic acid wedges bearing (*S*)‐3,7‐dimethyloctyl alkyl chains for enhanced solubility of the supramolecular polymer in apolar, aliphatic solvents. The syntheses and characterization of **1‐Zn** and **1‐FB** have been reported previously.^[26]^ Starting from gallic acid methyl ester, first the chiral side chains are coupled and then the methyl ester deprotected. Then, the acid chloride is formed and converted into an acyl azide. In a Curtius rearrangement reaction, the acyl azide is transformed via an isocyanate into the aniline, which is used in an amide coupling reaction to form **1‐FB**. For the NH‐centered **2**, the first steps of the synthesis are identical up to the acid chloride, which is directly coupled with commercially available 5,10,15,20‐(tetra‐4‐aminophenyl) porphyrin to obtain **2‐FB**.^[34]^ In comparison to the *C*‐centered, the *N*‐centered monomer is synthetically more accessible with standard reaction procedures and avoiding toxic reagents or intermediates. Finally, to obtain the porphyrin monomers, transmetalation with zinc (II) acetate yielded both **1‐** and **2‐Zn** in good overall yields (see Scheme S1–2 in the Supporting Information). Both ligands were obtained in high purity as evidenced by ^1^H and ^13^C NMR, MALDI‐TOF and FT‐IR.

A first indication of the differences in properties of the *C*‐ and *N*‐centered monomers can be derived from Fourier‐transform infrared spectroscopy (FT‐IR) in the bulk phase. The bands for the NH and C=O stretches are indicative of the donor‐acceptor properties of the amides and thereby the strength of hydrogen bonds. The free base monomers both exhibit the NH stretch at around 3320 cm^−1^ and the carbonyl stretch at 1654 cm^−1^ for **1‐FB** and 1647 cm^−1^ for **2‐FB** (see Table 1 and Figure S1). For the zinc centered monomers, the differences become more pronounced with the NH band appearing at much lower wavenumbers of 3264 cm^−1^ for **1‐Zn** compared with **2‐Zn** at 3336 cm^−1^. Interestingly the C=O bands are found at almost identical wavenumbers as for the free base ligands.

**Table 1:** Overview of melting (*T*_m_) and crystallization temperatures (*T*_c_) in DSC measurements and NH‐ and CO‐ stretches from FT‐IR in the solid state.

	*T*_c_ [°C]^a^	*T*_m_ [°C]^a^	*v*_NH_ [cm^−1^]^b^	*v*_CO_ [cm^−1^]^b^
**1‐FB**	214	232	3319	1654
**2‐FB**	224	252	3320	1647
**1‐Zn**	203	230, 239	3264	1654
**2‐Zn**	159	203	3336	1648

[a] Data derived from second heating run in DSC. [b] FT‐IR spectrum measured at room temperature on pristine sample.

The strength of molecular interactions can also be assessed through the thermal properties characterized by dynamic scanning calorimetry (DSC, Table 1, and Figure S4). For the free base ligands, we find a higher melting point for *N*‐centered **2‐FB** at 252 °C compared to 232 °C for *C*‐centered **1‐FB**. For zinc centered monomers however, we find a higher melting point for **1‐Zn** at 230 °C than for **2‐Zn** at 159 °C. Taking the results from both FT‐IR and DSC into account, it seems that molecular interactions between **1‐Zn** monomers are stronger than between **2‐Zn** monomers, although for free base ligands the differences are smaller. As both free base monomers have similar IR spectra, the variation in melting points likely arise from differences in molecular packing and space filling in the bulk phase.

### Supramolecular homopolymerization of 1‐Zn and 2‐Zn in MCH

**Figure 2:**
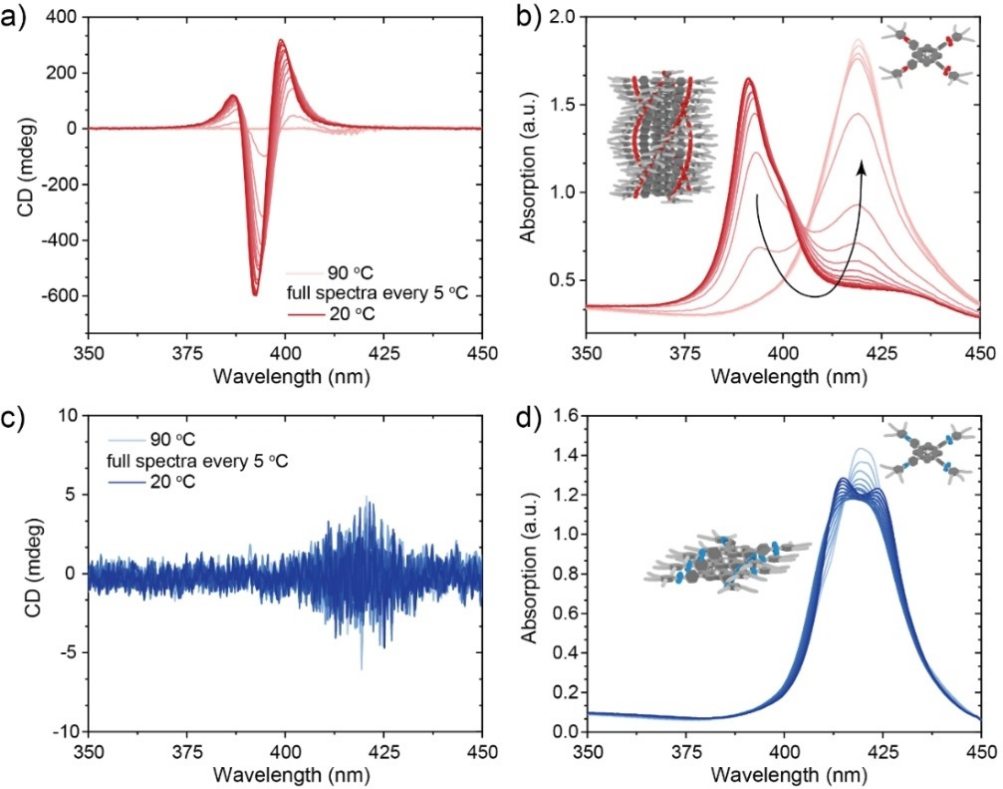
Full CD (a, c) and absorbance (b, d) spectra of monomers **1‐Zn** and **2‐Zn** (*c*=50 μM in MCH) measured in intervals of 5 °C from 90–20 °C with a cooling rate of 1 °C min^−1^. While **1‐Zn** forms mostly helical H‐aggregates via a cooperative polymerization pathway, **2‐Zn** seems to form only optically inactive, J‐type aggregates in MCH.

Next, we investigated the ability of **1‐Zn** and **2‐Zn** to form supramolecular polymers in nonpolar solvents by CD und UV‐Vis spectroscopy. Previous work has shown that **1‐Zn** in methylcyclohexane (MCH) polymerizes cooperatively into chiral H‐aggregates with a strong bisignate CD effect at λ=392 nm (Figure 2a) and to a lesser extend into isodesmic achiral J‐aggregates with a maximum absorption at 425 nm.[^7^, ^9^, ^26^]

With increasing temperature, the H‐ and J‐aggregates of **1‐Zn** depolymerize and convert into the monomeric state (Figure 2a and b). For **2‐Zn** however, no CD signal was found in MCH. In the UV‐Vis spectrum at low temperatures, **2‐Zn** exhibits two strong absorption bands at λ=414 and 424 nm, which merge into a single absorption peak at 420 nm upon heating (Figure 2c and d). This difference in assembly is quite striking as the change in the molecular design is only minor. Similarly, **1‐FB** forms helical supramolecular stacks, which convert into J‐aggregates and then into the molecularly dissolved monomeric state upon increasing the temperature (Figure S5c and d). For **2‐FB** a small CD signal can be observed at 425 nm, but mostly J‐type aggregates seem to be present as evidenced by the absorbance spectra (Figure S5a and b). In the free monomer state at 90 °C, the absorbance spectra of **1‐** and **2‐Zn** are almost identical, which indicates that the amide connectivity does not have a major influence on the photo‐optical properties in that state. The difference in CD and absorbance at lower temperature is thus purely caused by differences in aggregation and the exciton coupling in the respective aggregates.^[35]^ The electronic coupling for H‐aggregated **1‐Zn** is strong, therefore the absorbance shifts to lower wavelengths, while for the weakly coupled J‐aggregates of **2‐Zn** the changes in the spectrum are less pronounced and maximum absorption is shifted to higher wavelengths.

To study the influence of aggregation on the luminescence of the porphyrins, we measured variable‐temperature fluorescence spectroscopy (Figure S6). The *N*‐centered amide connectivity should slightly increase conjugation over the porphyrin core and thereby decrease the HOMO‐LUMO gap, making the electronic excitation easier for **2‐Zn**. When exciting at 390 nm (H‐aggregate of **1‐Zn**), the fluorescence measurements display a sharp change in the emission band close to the temperature of elongation (*T*_e_) of the polymers, with the maximum peak of emission shifting from 650 nm at 90 °C to 600 nm at 10 °C. The difference of absorption and emission over temperature is less pronounced for **2‐Zn**, with a bathochromic shift of the emission by roughly 20 nm with decreasing temperature (Figure S6c and d). The intensity of emission for **2‐Zn** is much stronger than for **1‐Zn**, which arises from higher conjugation but also hints to a lower degree of aggregation at room temperature.

**Figure 3:**
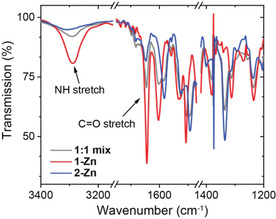
FT‐IR spectra of solutions of **1‐** and **2‐Zn** and a 1 : 1 mixture (*c*=2.0 mM in MCH). The NH stretch of **1‐Zn** is found at lower wavenumbers of 3289 cm^−1^ compared to 3312 cm^−1^ for **2‐Zn**, indicating stronger hydrogen bonding for **1‐Zn**.

In H‐type aggregates, the monomers are organized face on with four‐fold hydrogen bonding between the monomers, whereas in J‐aggregates they are packed in a staggered fashion, thus not forming ordered stacks with multiple hydrogen bonds. This difference in hydrogen bonding pattern is observable with FT‐IR in solution. In 2 mM solutions of **1**‐ and **2‐Zn** in MCH, the NH stretch appears at *ν*_NH_=3289 cm^−1^ for **1‐Zn** and at 3312 cm^−1^ for **2‐Zn** while the C=O stretch is found at around 1645 cm^−1^ for both (Figure 3). The lower *ν*_NH_ observed for **1‐Zn** and a much higher absorbance indicates stronger hydrogen bonding between the monomers. When repeating the same measurement for 2 mM solutions in chloroform (molecularly dissolved monomers), the NH stretch shifted to higher wavenumbers of around 3435 cm^−1^ for both monomers, which is in the range of free NH‐groups (Figure S3). Here, the intensity of the NH stretch was similar for both **1‐** and **2‐Zn**. These findings are in good agreement with the spectroscopic measurements, which indicate similar absorbance in the molecularly dissolved state, but pronounced differences upon aggregation.

### Density functional theory calculation of the polymerization

**Figure 4:**
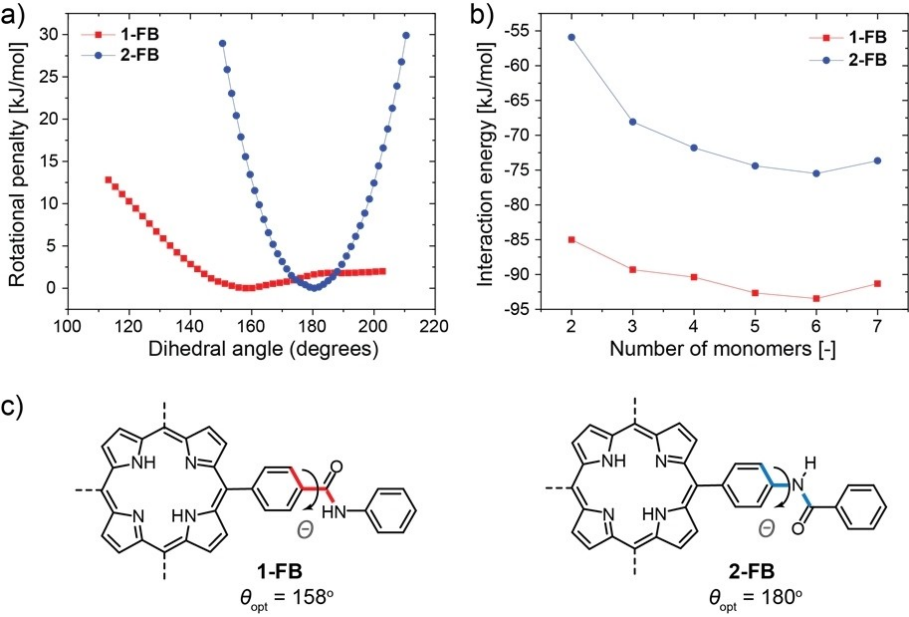
a) Energy profiles for torsion around the amide C−C=O bond of **1‐FB** or C−NH bond of **2‐FB**. b) Interaction energies for the association of monomers of **1‐** and **2‐FB** when arranged in a H‐type aggregate, showing a lower interaction energy after dimer formation typical for cooperative systems. c) Illustration of dihedral angle *Θ* for the rotation of the amide groups of ligands **1‐FB** (red) and **2‐FB** (blue). Details on the calculations can be found in the Supporting Information.

In order to understand the origins of the differences in assembly for monomers **1** and **2** we performed DFT calculations using the Vienna ab initio simulation package (VASP).[^36^, ^37^, ^38^] To simplify the calculations and reduce calculation time, the free base ligands without metal centers were used and the −O−R side chains of the phenyl rings were replaced by hydrogen atoms. First, we studied the flexibility and energetic costs of rotation of the amide groups in its free form, as it is an important parameter for the ability of the monomer to assemble into hydrogen‐bonded supramolecular polymers. When forming H‐aggregates, the amides must rotate out of the monomer plane to produce hydrogen bonds along the polymer backbone. To compare the conformational flexibility of both monomers, we computed torsional energy profiles for the rotation around the C−C=O bond for **1‐FB** or the C−NH bond for **2‐FB** (Figure 4a and c). Herein, it is found that the equilibrium angles *Θ* of the two monomers differ substantially, while **2‐FB** takes up a flat conformation (*Θ*=180°), the amides of **1‐FB** are already tilted out of plane (*Θ*=158°). The cost to rotate the amides of **2‐FB** out of their equilibrium angle of 180° is significantly larger compared to the cost of rotating the amides of **1‐FB** out of their equilibrium angle of 158°. This large difference can be rationalized with the sp^2^ hybridized structure of the C−NH functionality in **2‐FB**, that has a favorable resonance structure, making this configuration exceptionally stable.^[39]^ Out‐of‐plane rotation of this functionality is thus associated with a strong increase in energy, therefore only J‐aggregates are formed upon aggregation at low temperatures. The main interaction between the monomers in J‐aggregates are induced by solvophobic forces, resulting in π‐stacking that is reinforced by the planar configuration and thus leading to a larger π‐conjugated plane. In contrast, the rotation around the C−C=O bond of **1‐FB** shows a flatter, asymmetric energy profile. Here the dominant forces are steric and Pauli repulsion of the carbonyl group with the neighboring benzene ring, which is dependent on the direction of rotation giving an asymmetric profile in the calculated range of Θ.

Second, we calculated the association and interaction energies of dimers up to heptamers (Figure 4b) for both monomers in their H‐aggregated stack. The interaction energy of the *n*‐mers was obtained following equation (1) by subtracting *n* times the energy of free monomer from the energy of the *n*‐mer and divide that by the number of intermolecular interactions (*n*‐1.

(1) $$\Delta E{}_{avg}=\left(E{}_{n}\text{-}nE{}_{1}\right)/\left(n\text{-}1\right)$$

For *C*‐centered **1‐FB**, a lower interaction energy is found than for *N‐*centered monomers with a difference of roughly 30 kJ/mol, thus predicting more stable H‐aggregates for **1‐FB**. The maximum of the interaction energy, corresponding to the nucleus of the polymerization process, is found for the dimer in both cases. After the dimer, oligomers of larger size all show an increasingly lower interaction energy (images of *n*‐mer stacks in Figure S12). This is typical for highly cooperative systems, after nucleus formation the addition of another monomer is energetically more favorable, and elongation of the growing chain occurs. It should be emphasized that, although an increase in the interaction energy (less stable) is observed for the heptamer, this most likely corresponds to a geometry that is not fully optimized and it is reasonable to expect that further optimization would result in an interaction energy similar to or lower than the hexamer. Additionally, we analyzed the angles and intermonomer distances of the *n*‐mers to assess the changes in arrangement with increasing number of monomers (Figure S13–16). We observe the largest decrease in distance and shift in the dihedral angles for central monomers, as they experience, on average, the strongest effect of the cooperative interaction of neighboring monomers on both sides (i.e. both top and bottom side). Furthermore, we found that the oxygen functionality of the amides tends to favor an orientation at the chain ends, which was also found for similar BTA stacks and attributed to long‐range dipole‐dipole, non‐pairwise short‐range polarization and resonance‐assisted hydrogen binding in a chain of dipoles.^[30]^

When assembling into H‐aggregates, the monomers of **1‐FB** gain energy through formation of four intermolecular hydrogen bonds along the polymer backbone. Assuming that the energy gain is similar to hydrogen bonding in proteins, each hydrogen bond formation would give roughly 15–30 kJ/mol.[^40^, ^41^, ^42^] When comparing the energetic costs for rotation around the amide groups of **1‐** or **2‐FB**, it becomes apparent that the energetic costs for **2‐FB** quickly outweigh this benefit. Although the calculated interaction energies show that the H‐aggregate of **2‐FB** is thermodynamically allowed, it is kinetically inaccessible. Looking back at the results for the homopolymerization for ligands **1** and **2**, we therefore conclude that for *N*‐centered **2** the rotation penalty is too high, and therefore only J‐aggregates are formed through π‐stacking interactions rather than hydrogen bonding as for H‐aggregates of *C*‐centered **1**. The calculations also give an indication of the π‐stacking in the H‐aggregate as a function of the stack length. The more monomers are stacked, the more the π‐stacking distance is decreasing in an almost linear fashion for the center porphyrin in the stack (Figure S14 and S16). As a result, the rotation of the amide bond to form the H‐aggregate is lower, leading to a smaller penalty.

### Mixed polymerization of N‐ and C‐centered porphyrins

**Figure 5:**
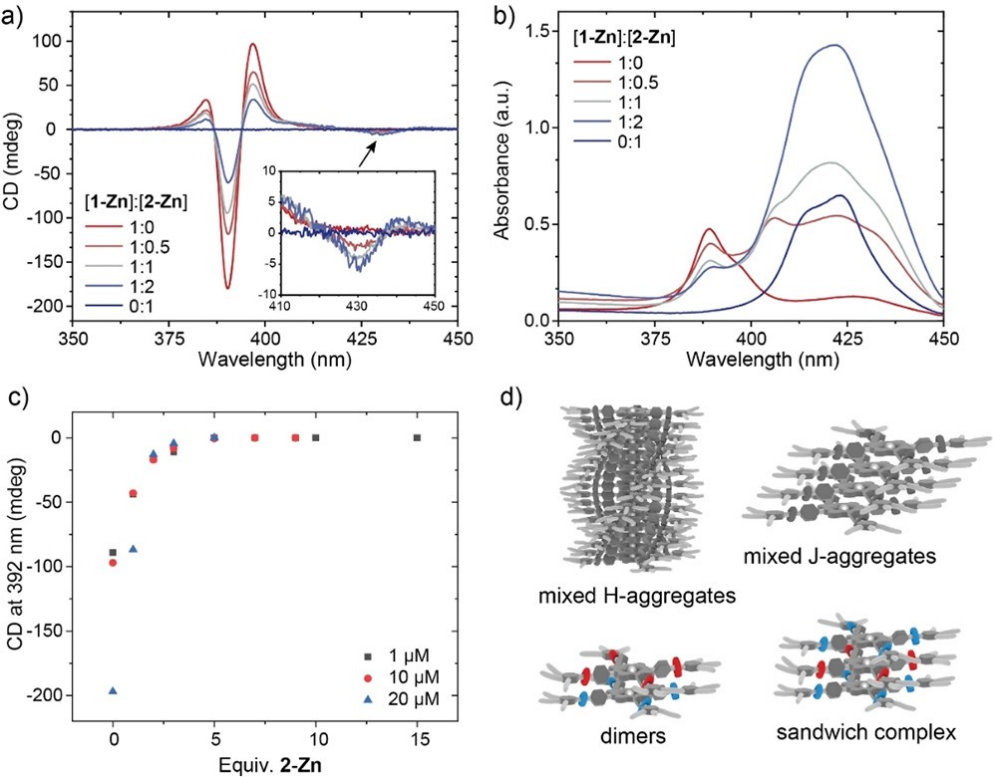
CD (a) and absorbance (b) spectra of mixtures of **1‐Zn** and **2‐Zn** (*c*=25 μM in MCH). For the mixtures, the concentration of **1‐Zn** was kept constant at *c*=25 μM and the respective equivalents of **2‐Zn** were added to exclude dilution effects. With increasing equivalents of **2‐Zn** the optically active helical H‐aggregate of **1‐Zn** at λ=392 nm becomes destabilized and mixed species are formed, which exhibit a weak CD effect at 430 nm (inset) and new absorbance maxima at 406 and 437 nm. Remeasuring the mixed samples after two weeks gave almost identical spectra, indicating that the co‐assembled state is close to the thermodynamically stable one. c) Monomers from the H‐aggregates are fully sequestrated with 3–5 equiv. of **2‐Zn** independent of concentration. d) Possible aggregate types during copolymerization.

Next, we investigated the copolymerization of the *C*‐centered monomer **1‐Zn** with *N*‐centered **2‐Zn**. Chiral porphyrins such as **1‐Zn** are known to be typical narcissistic self‐sorters. The energetic penalty for intercalating into a growing stack of opposite helicity is high and therefore self‐sorted *P* and *M* helical stacks co‐exist in solution.^[43]^ Based on the results of homopolymerization and previous studies, three outcomes are thus possible for a mixture of **1‐Zn** and **2‐Zn**: (1) self‐sorting of *C*‐ and *N*‐centered stacks; (2) copolymerization in mixed H‐ or J‐aggregates; or (3) new types of finite aggregates such as dimers or sandwich type complexes, while a mixture of the three scenarios is possible as well. To elucidate these possibilities, we prepared mixtures of [**1‐Zn**]:[**2‐Zn**] in the ratios of 1 : 0.5, 1 : 1 and 1 : 2 while keeping the concentration of **1‐Zn** constant at *c*=25 μM. After a thermal equilibration by heating the samples to 90 °C and cooling to 20 °C, the CD and absorbance spectra were recorded (Figure 5). In the CD curves, a destabilization of the H‐aggregate of **1‐Zn** can be observed. With one equivalent of **2‐Zn**, the CD signal at 392 nm decreases to roughly half of its original intensity (Figure 5a). When increasing the molar ratio of **2‐Zn** further to 3–5 equivalents of **1‐Zn**, no more H‐aggregate is observed and the intensity of the band at 437 nm does not increase any further (Figure 5c and Figure S8). A new CD signature becomes visible at λ=430 nm (see inset Figure 5a) that gets more pronounced with increasing molar ratio of **2‐Zn**. At 90 °C this new CD signal remains, demonstrating the high thermal stability of this chiral species (Figure S9). In the corresponding absorbance spectra, two new bands appear at λ=406 and 437 nm, indicating the presence of several other mixed species. The mixed aggregates of **1**‐ and **2‐Zn** show generally higher emission, but the strongest fluorescence is found for the aggregate at 437 nm (Figure S7).

As the absorption maximum of the H‐aggregate peak does not shift to higher wavelengths it is unlikely that **2‐Zn** intercalates into the **1‐Zn** H‐aggregates, but rather sequestrates **1‐Zn** monomers into mixed J‐type or other finite aggregates. Based on previous studies, the presence of **2‐Zn** sequestrator should reduce the temperature of elongation (*T*_e_) for the H‐aggregate of **1‐Zn** as it reduces the amount of monomer available for nucleus formation and subsequent elongation of the supramolecular polymer.[^44^, ^45^, ^46^] In variable temperature CD melting experiments, we indeed find a reduction of the *T*_e_ with increasing molar ratios of **2‐Zn**, going from 69 °C for pure **1‐Zn** to 55 °C for the 1 : 1 mixture and 46 °C for the 1 : 2 mixture (Table S1 and Figure S10). This is further supported when comparing the solution IR spectra of pure **1‐** or **2‐Zn** with the 1 : 1 mixture (Figure 3). In the mixture, the NH‐band indicative of strong hydrogen bonding in the polymers decreases significantly confirming the disappearance of H‐aggregates in the mixture. Interestingly, the quantity of added **2‐Zn** necessary to fully depolymerize and sequestrate monomers from the chiral H‐aggregates does not change in the concentration regime from 1–20 μM (Figure 5c). This weak concentration dependence of the existence of cooperative polymers originates from the presence of the competitive isodesmic pathway to co‐aggregate formation.[^7^, ^47^]

**Figure 6:**
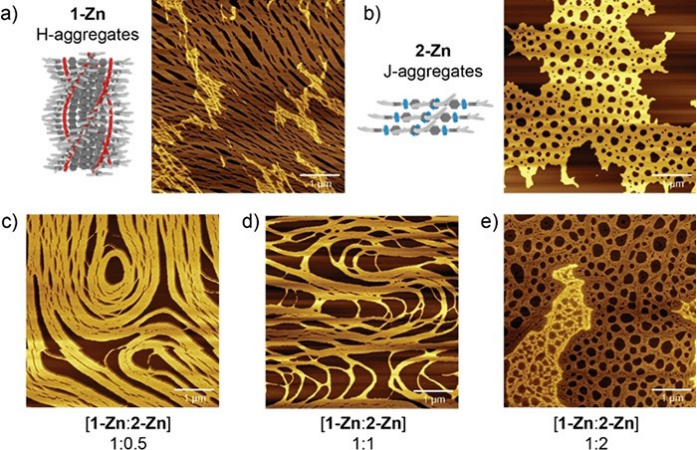
AFM images of pure **1‐** and **2‐Zn** and mixtures thereof with varying ratios drop‐casted on mica substrates. The concentration of **1‐Zn** was kept constant at 20 μM in MCH and the respective aliquots of **2‐Zn** were added to avoid dilution effects. With increasing ratio of **2‐Zn** the morphology changes from fibrous to disordered structures (Scale bars are 1 μm, height profiles of **1‐Zn** and **2‐Zn** images are given in Figure S11).

To gain insight into the morphology of the aggregates formed in the homo‐ and copolymerization, we performed atomic force microscopy (AFM). For **1‐Zn**, long and bundled fibrous structures were found on the substrate, while for **2‐Zn** only amorphous deposits were observed (Figure 6a and b). Although the morphology of the deposits differs, the height of the structures in the AFM images was 3–4 nm for both (Figure S11). For the mixed samples, the number and length of fibers decreased with increasing molar ratio of **2‐Zn** (Figure 6c–e). Based on the spectroscopic experiments together with the AFM images, we conclude that the two monomers **1‐Zn** and **2‐Zn** co‐aggregate and that **2‐Zn** acts as a sequestrator, which transforms **1‐Zn** from the H‐aggregated supramolecular polymer into mixed J‐aggregates.

## Conclusion

Here, we combined extensive spectroscopic analysis with DFT calculations to investigate the supramolecular polymerization of two porphyrins monomers, one with C=O centered and one with NH‐centered amide groups connecting the porphyrin core with the periphery. While the *C*‐centered derivative **1‐Zn** polymerizes cooperatively into helical supramolecular fibers, the *N*‐centered **2‐Zn** only forms short J‐aggregates via an isodesmic pathway. DFT calculations on the molecular differences in aggregation indicate that for both monomers the formation of H‐aggregates is thermodynamically favored via a cooperative mechanism after nucleus formation, although more stable for the *C*‐centered ligand **1‐FB**. For *N*‐centered **2‐FB** however, the rotation penalty for turning the amide groups out of the plane is higher than the energetic gain from forming hydrogen bonds, therefore only π‐stacked J‐aggregates are formed. In mixed polymerizations, **2‐Zn** sequestrates **1‐Zn** monomers from chiral H‐aggregates into achiral J‐aggregates. This shift in aggregate type suggests that in the mixture, π‐stacking interactions outweigh hydrogen bonding between monomers. The interaction between the *N*‐ and *C*‐centered monomers appears to be thermodynamically more favorable than self‐sorting. Interestingly, the transition from H‐ to mixed co‐aggregates is only dependent on the monomer ratio and not dependent on the concentration.

Supramolecular systems exhibit high sensitivity to minor changes in molecular design, which often has dramatic consequences for the assembly properties. As chemists we aim to design molecular systems that have precise functionality and structure, but our understanding of structure‐function relationships is limited. Therefore, this work participates in the efforts to develop an understanding of the properties of molecular assemblies by combining experimental data with theoretical calculations.

## Conflict of interest

The authors declare no conflict of interest.

## Supporting information

As a service to our authors and readers, this journal provides supporting information supplied by the authors. Such materials are peer reviewed and may be re‐organized for online delivery, but are not copy‐edited or typeset. Technical support issues arising from supporting information (other than missing files) should be addressed to the authors.

Supplementary
