# Supplementary material for: Consequences of Amide Connectivity in the Supramolecular Polymerization of Porphyrins: Spectroscopic Observations Rationalized by Theoretical Modelling
Source: Chemistry. 2021 May 27;27(37):9700–7. doi: 10.1002/chem.202101036 (PMC8362183; doi:10.1002/chem.202101036)
Supplement: Supplementary file 1 — Supplementary [file CHEM-27-9700-s001.pdf]

Chemistry–A European Journal

Supporting Information

Consequences of Amide Connectivity in the Supramolecular Polymerization of Porphyrins: Spectroscopic Observations Rationalized by Theoretical Modelling

Elisabeth Weyandt, Ivo A. W. Filot, Ghislaine Vantomme, and E. W. Meijer*

Table of Contents

1. Materials and methods: 3

   1.1 Materials 3

   1.2 Characterization 3

   1.3 Spectroscopy 3

   1.4 Sample preparation 3

2. Synthesis 4

   2.1 Synthesis of N-centered ligand **2-FB** 4

   2.2 Synthesis of N-centered porphyrin monomer **2-Zn** 5

3. Fourier-Transform Infrared Spectroscopy 6

4. Dynamic Scanning Calorimetry (DSC) 8

5. Assembly of 1-Zn and 2-Zn in apolar solvents 9

   5.1 Assembly of free base ligands in apolar solvents 9

   5.2 Emission spectra of **1-Zn** and **2-Zn** 10

   5.3 Copolymerization of **1-Zn** and **2-Zn** 11

6. Atomic force microscopy 13

7. Density functional theory calculations of monomers 14

8. Spectra of Compounds: 18

   8.1 ^{1}H NMR spectra: 18

   8.2 ^{13}C and 2D NMR spectra: 19

   8.3 MALDI-TOF: 22

9. References 23

10. Appendix: DFT coordinates 24

1. Materials and methods:

1.1 Materials

Unless stated otherwise, reagents and solvents were obtained from commercial sources and used without further purification. All solvents were of AR grade or of spectrophotometric grade, when used in spectroscopy experiments. Reactions were conducted under argon atmosphere and in dried glassware unless noted otherwise. Flash column chromatography was done on a Biotage Isolera One system with an ultraviolet detector.

1.2 Characterization

MALDI-TOF mass spectra were measured using a PerSerptive Biosystem Voyager-DE PRO spectrometer using either α-cyano-4-hydroxycinnamic acid (CHCA) or 2-[(2E)-3-(4-tert-butylphenyl)-2-methylprop-2-enylidene]malononitrile (DCTB) as matrices. IR spectra were obtained on a Perkin-Elmer spectrum two FT-IR spectrometer. Atomic force microscopy (AFM) experiments were performed on an Asylum MFP3 system with Olympus AC240 tips in tapping mode. Image analysis for AFM was done with Gwyddion software version 2.50. Mica sheets used in the experiment were of V1-grade and freshly cleaved before dropcasting the solutions.

1.3 Spectroscopy

UV/Vis and circular dichroism (CD) spectra were recorded on a Jasco J-815 spectropolarimeter with a Jasco PFD-425S/15 Peltier. The measurement parameters for sensitivity, scanning rates and ranges were chosen appropriately for each experiment. Cuvette cells with an optical path length of either 1 mm or 1 cm were used for all experiments.

1.4 Sample preparation

Stock solutions (100 µM) were prepared by weighing the compound into a vial and adding the appropriate amount of solvent. One milliliter of a polar solvent (dichloromethane or chloroform) was added to break up aggregates and re-evaporated. Then methylcyclohexane (MCH) was added, heated with a heat gun and sonicated until complete dissolution. Samples for measurements were prepared by diluting the stock solution to the respective concentration. The molar dichroism $\Delta\varepsilon$ was calculated as $\Delta\varepsilon$ = CD effect / ($c \cdot l$), with the concentration c (mol · L^{-1}) and the optical path length l (cm).

2. Synthesis

The synthesis of (*S*)-3,7-dimethyloctyl functionalized acid **3**, free base ligand **1-FB** and zinc porphyrin **1-Zn** has been published previously.[1] The synthesis of **2-FB** was adapted from literature.[2] Tetra-4-aminophenyl porphyrin was purchased from PorphyChem and used without further purification.

*2.1 Synthesis of N-centered ligand **2-FB***

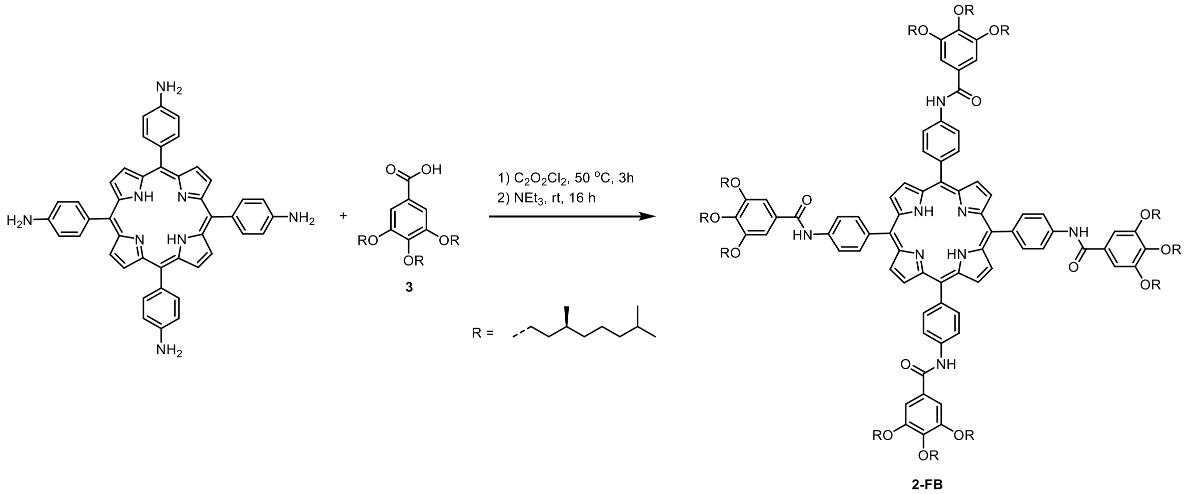

Scheme S1: Synthesis of *N*-centered free base porphyrin ligand **2-FB**.

Triple side chain functionalized acid **3** (650 mg, 1.1 mmol) was converted to the acid chloride by addition of oxalyl chloride (850 mg, 6.67 mmol) and refluxing for three hours. Due to the instability of the acid chloride it was immediately used for the next step without isolation. Tetra-4-aminophenyl porphyrin (150 mg, 0.22 mmol) was suspended together with the freshly prepared acid chloride in 100 mL dry chloroform with 1.5 mL triethylamine (NEt_3) and stirred overnight with protection from light. The dark red reaction mixture was extracted with 2 x 50 mL of water and 1 x 50 mL of 0.5 M hydrochloric acid solution. The combined organic phases were dried over $MgSO_4$, filtered and the solvent removed in vacuo. The product was purified by column chromatography (Biotage®, gradient of EtOAc/$CHCl_3$/heptane in the ratio of 2:4:4). The product was precipitated from 5% chloroform in MeOH and isolated as a dark red powder (456 mg, yield: 69%).

^{1}H NMR (400 MHz, $CDCl_3$) δ 8.92 (s, 8H), 8.24 (d, *J* = 8.3 Hz, 9H), 8.09 – 8.02 (m, 12H), 7.24 (d, *J* = 8.9 Hz, 8H), 4.15 (m, 24H), 1.91 (m, 9H), 1.81 – 1.58 (m, 24H), 1.46 – 1.08 (m, 38H), 0.99 (m, 37H), 0.89 (d, *J* = 6.6 Hz, 87H), -2.74 (s, 2H). ^{13}C NMR (101 MHz, $CDCl_3$) δ 171.24, 166.19, 161.18, 153.59, 141.91, 138.45, 137.96, 135.35, 130.16, 119.73, 118.54, 106.09, 72.04, 68.05, 39.54, 39.45, 37.69, 37.54, 36.58, 30.05, 29.86, 28.17, 24.93, 24.90, 22.89, 22.80, 22.78, 19.79, 19.76. MALDI-TOF MS: [M^+] calcd. for $C_{192}H_{290}N_8O_{16}$: 2964.21, found 2966.27. IR (cm^{-1}): 3316, 2953, 2925, 2869, 1677, 1648, 1582, 1517, 1492, 1468, 1426, 1383, 1365, 1330, 1234, 1205, 1111, 998, 966, 851, 800, 754, 734.

*2.2 Synthesis of N-centered porphyrin monomer **2-Zn***

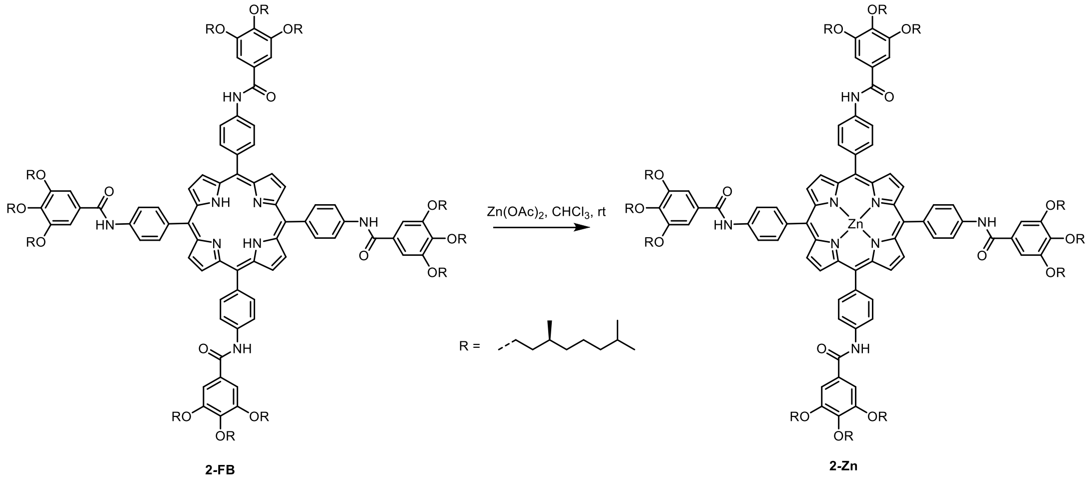

Scheme S2: Synthesis of **2-Zn** by zinc insertion into **2-FB**.

The ligand **2-FB** (250 mg, 0.85 mmol) was dissolved in 100 mL DCM and stirred together with $Zn(OAc)_2$ (170 mg, 1.26 mmol) overnight at room temperature with protection from light. The resulting purple reaction mixture was extracted with 2 x 50 mL of water, dried over $MgSO_4$, filtered and the solvent removed in vacuo. After purification by column chromatography (Biotage®, EtOAc/$CHCl_3$/Heptane in the ratio of 2:4:4) and precipitation from 5% $CHCl_3$ in MeOH the product was obtained as a purple, metallic shimmering powder (214 mg, yield: 84%).

^{1}H NMR (399 MHz, $CDCl_3$) δ 9.01 (s, 8H), 8.29 – 8.15 (m, 12H), 8.01 (d, *J* = 8.3 Hz, 8H), 7.17 (d, *J* = 15.1 Hz, 8H), 4.16 – 4.03 (m, 31H), 2.00 – 1.83 (m, 9H), 1.73 (ddq, *J* = 33.1, 14.2, 7.7 Hz, 13H), 1.56 (tdd, *J* = 13.0, 9.5, 7.0 Hz, 16H), 1.45 – 1.09 (m, 118H), 0.99 (t, *J* = 7.0 Hz, 31H), 0.90 (dd, *J* = 6.7, 3.3 Hz, 92H). ^{13}C NMR (100 MHz, $CDCl_3$) δ 166.21, 153.52, 153.49, 150.41, 141.82, 139.27, 137.69, 135.21, 132.12, 130.11, 120.56, 118.40, 106.08, 73.77, 72.00, 69.67, 67.98, 39.54, 39.49, 39.45, 37.69, 37.54, 37.25, 36.58, 32.91, 32.11, 32.08, 30.54, 30.19, 30.04, 29.93, 29.92, 29.87, 29.86, 29.83, 29.78, 29.61, 29.57, 29.53, 29.39, 28.16, 28.13, 27.24, 26.29, 24.93, 24.90, 22.87, 22.84, 22.79, 22.77, 19.88, 19.77, 19.75, 14.27, 14.21. MALDI-TOF MS: [M^+] calcd. for $C_{192}H_{288}N_8O_{16}Zn$: 3026.13, found 3029.11. IR (cm^{-1}): 3299, 2958, 2925, 2868, 1648, 1582, 1526, 1493, 1467, 1426, 1383, 1365, 1333, 1234, 1204, 1182, 1113, 999, 854, 812, 797, 753, 676, 571.

3. Fourier-Transform Infrared Spectroscopy

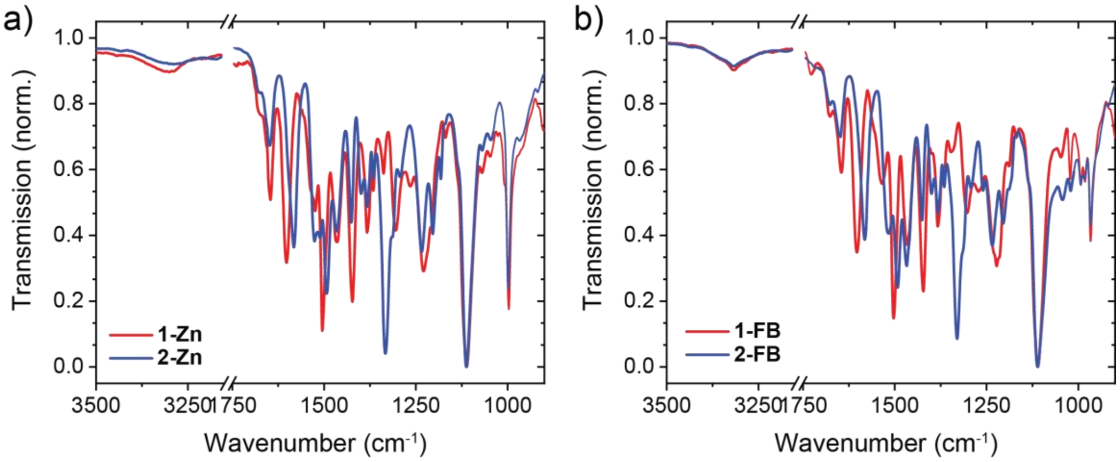

Figure S1: FT-IR spectra of zinc centered (a) and free base (b) ligands **1** and **2**.

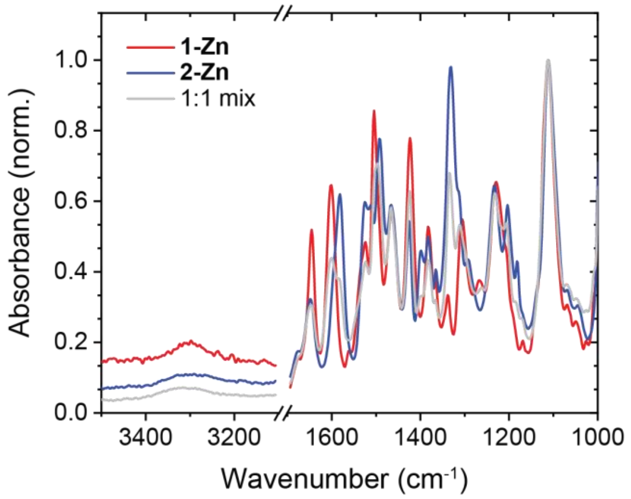

Figure S2: FT-IR spectra of **1-** and **2-Zn** and a 1:1 mixture. The mixture was prepared by dissolving both monomers together in a good solvent (chloroform) and letting the solvent evaporate slowly.

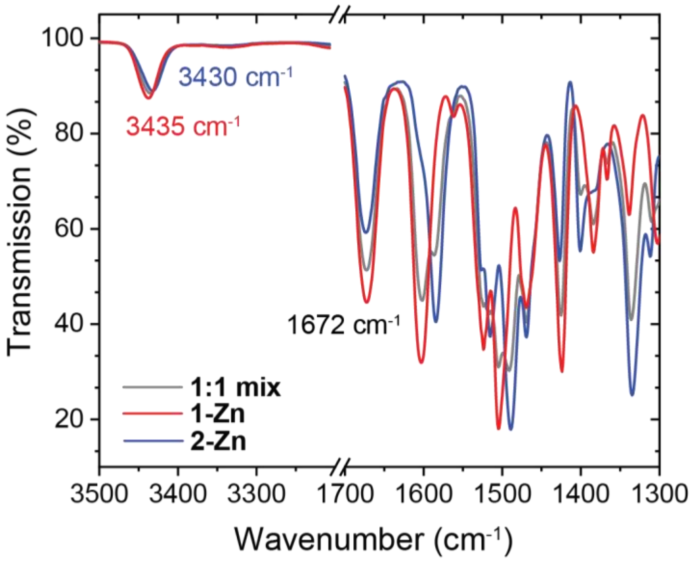

Figure S3: FT-IR spectra of 2 mM solutions of **1-** and **2-Zn** and a 1:1 mixture of both in chloroform. Both porphyrins exhibit a NH stretch of similar intensity at around 3430-3435 cm^{-1}, indicative of non-hydrogen bonded NH groups.

4. Dynamic Scanning Calorimetry (DSC)

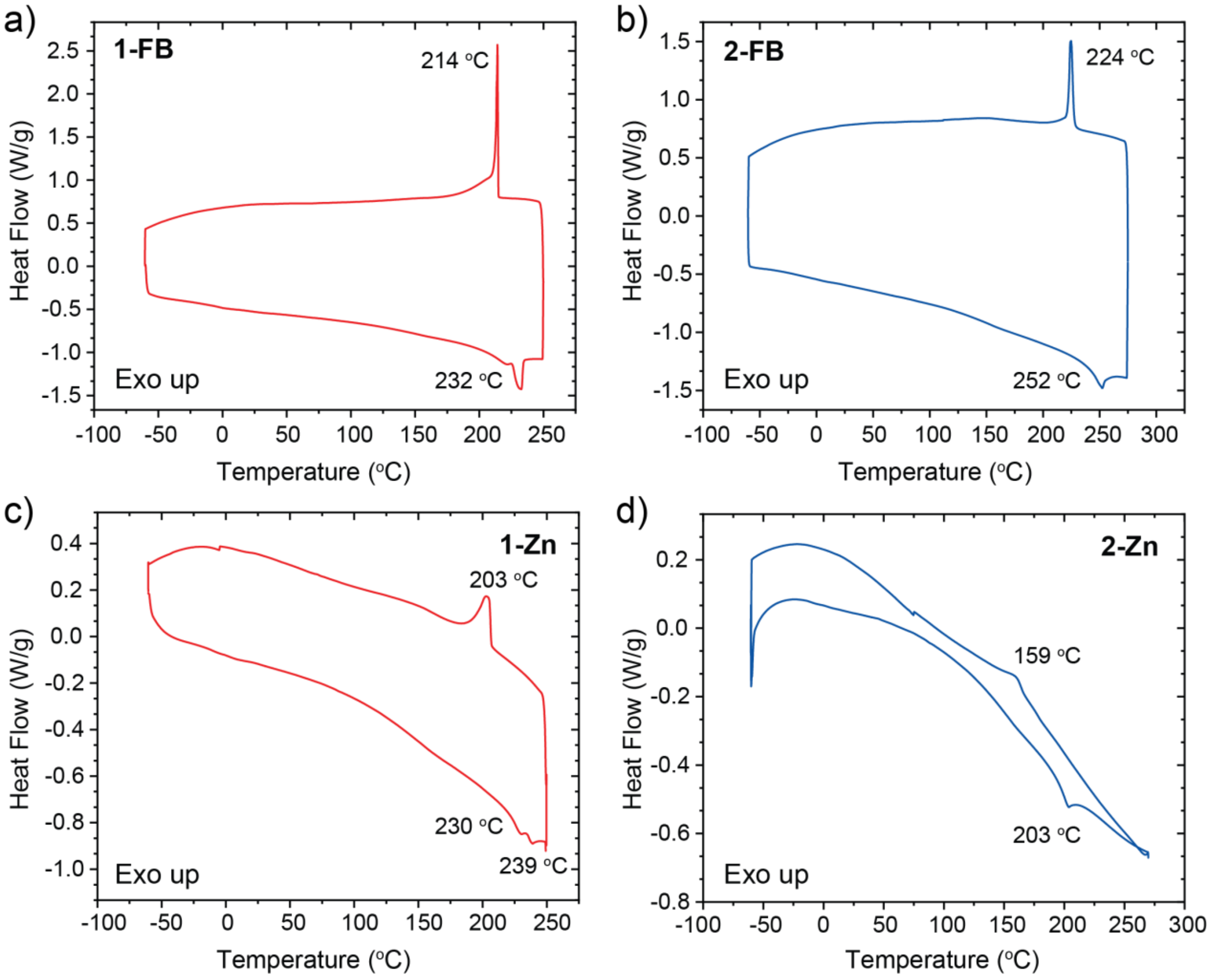

Figure S4: DSC graphs of free base (a, b) and zinc centered (c, d) ligands **1** and **2** with transition temperatures indicated in the plots. Data taken from the second heating/cooling run with a ramping rate of 10 °C/min.

5. Assembly of 1-Zn and 2-Zn in apolar solvents

5.1 Assembly of free base ligands in apolar solvents

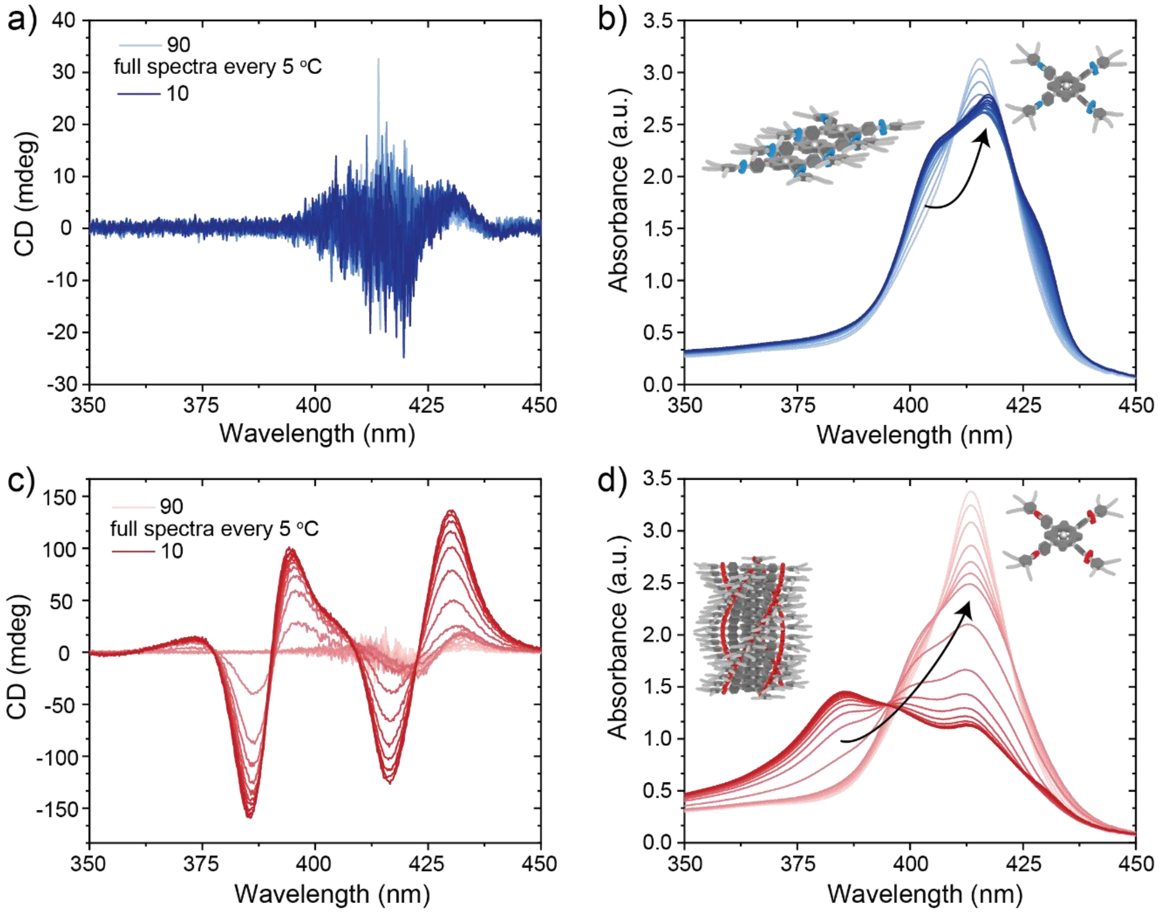

Figure S5: Full CD (a, c) and absorbance (b, d) spectra of free base monomers **1-FB** and **2-FB** (*c* = 50 µM in MCH) measured in intervals of 5 °C from 90-20 °C with a cooling rate of 1 °C/min.

*5.2 Emission spectra of **1-Zn** and **2-Zn***

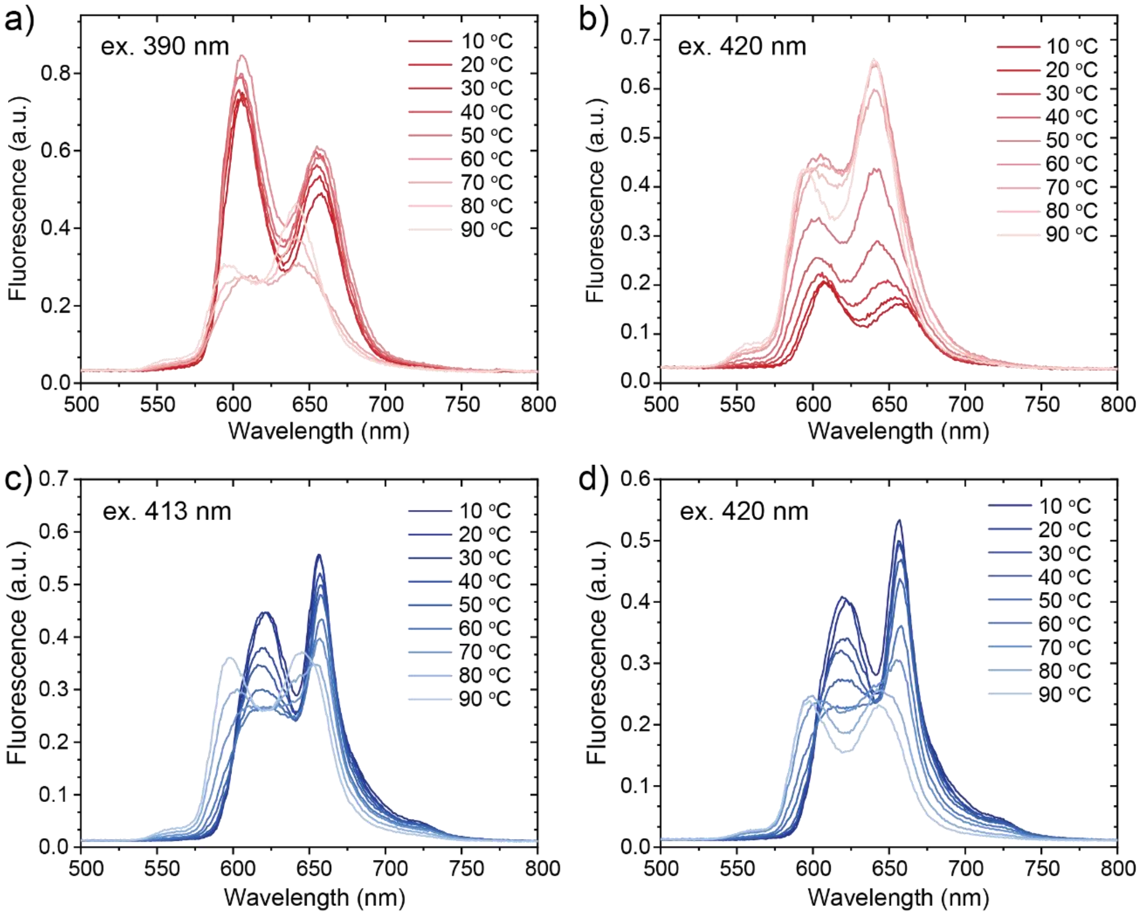

Figure S6: Emission spectra of **1-Zn** (a, b) and **2-Zn** (c, d) with respective excitation wavelengths noted in the plots (c = 10 µM, l = 10 mm). For both monomers the biggest absorbance peak at 20 and 90 °C were excited as emission properties are dependent on aggregation states. Detector sensitivity was set to 800 mV for **1-Zn** and to 700 mV for the more emissive **2-Zn**.

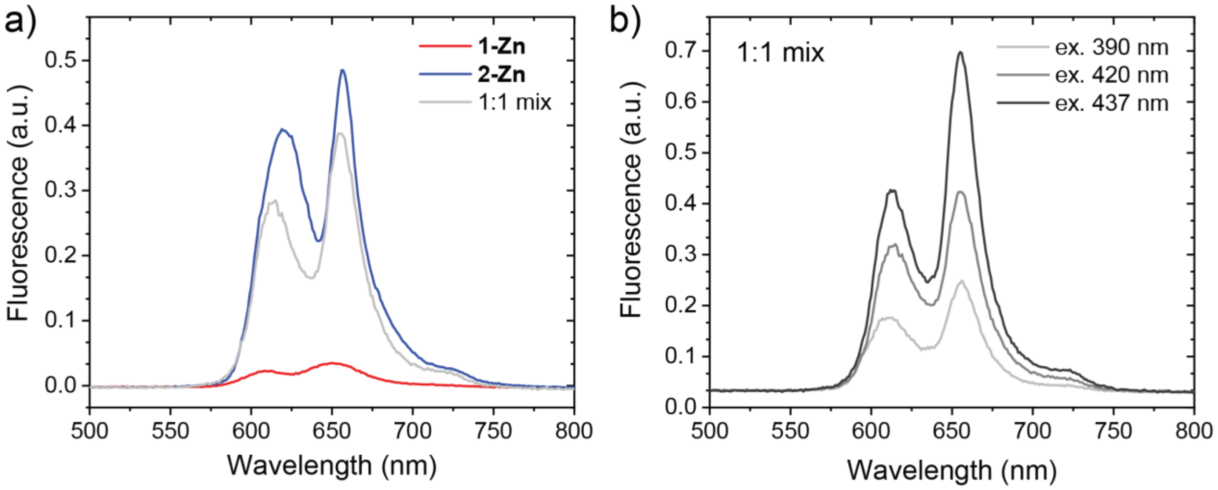

Figure S7: Emission spectra of a 1:1 mixture of **1-** and **2-Zn** (c = 10 µM of each). a) Comparison of emission of the pure polymers and a 1:1 mixture thereof (ex. 420 nm). b) Fluorescence intensity of the 1:1 mix plotted for excitation wavelength of 390 (H-aggregate), 420 (J-aggregate) and 437 nm (new co-aggregate). Detector sensitivity was set to 700 mV in all cases.

*5.3 Copolymerization of **1-Zn** and **2-Zn***

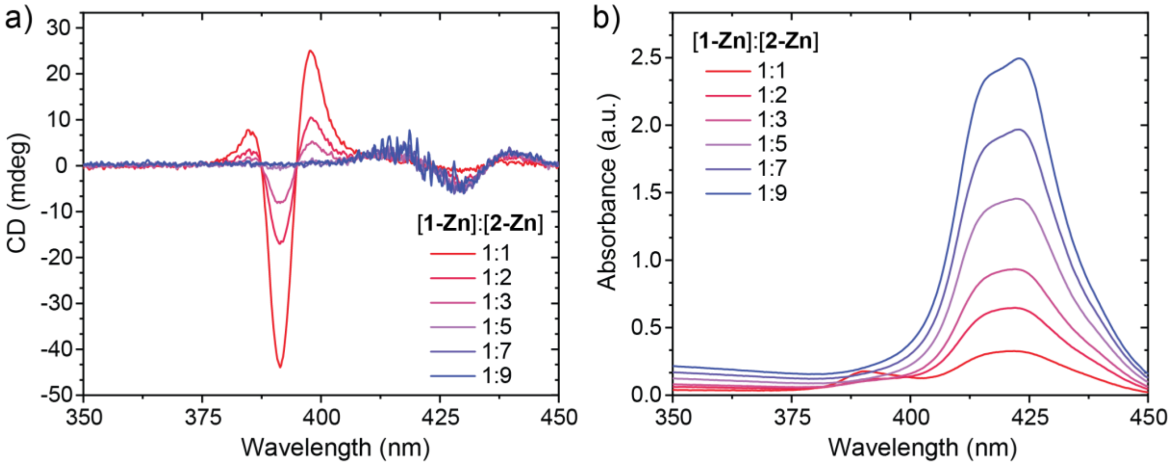

Figure S8: Spectral changes with increasing molar ratios of **2-Zn**. Concentration of **1-Zn** was kept constant at c = 10 μM in MCH.

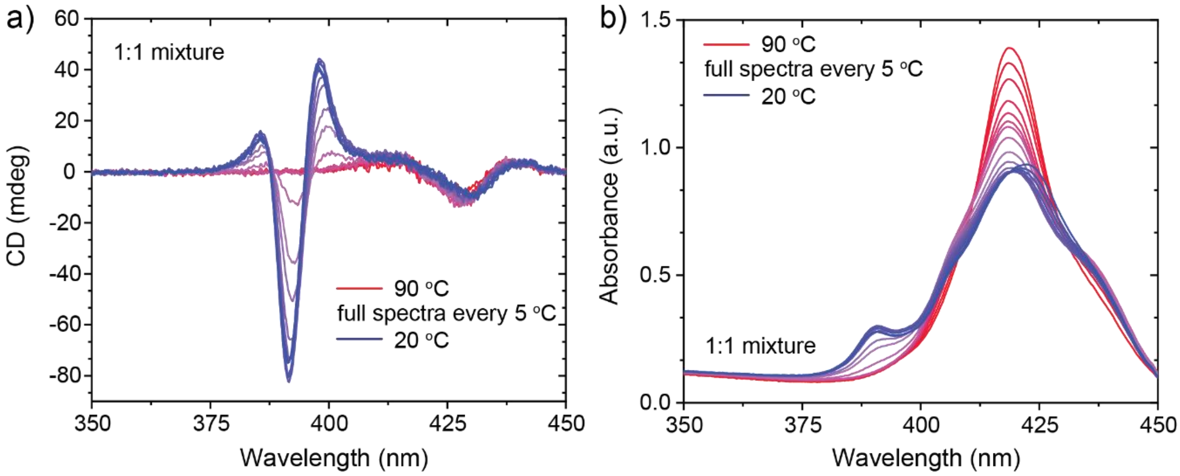

Figure S9: Full CD (a) and absorbance (b) spectra of a 1:1 mixture of **1-Zn** and **2-Zn** (c = 50 μM in MCH) measured in intervals of 5 °C from 90-20 °C with a cooling rate of 1 °C/min.

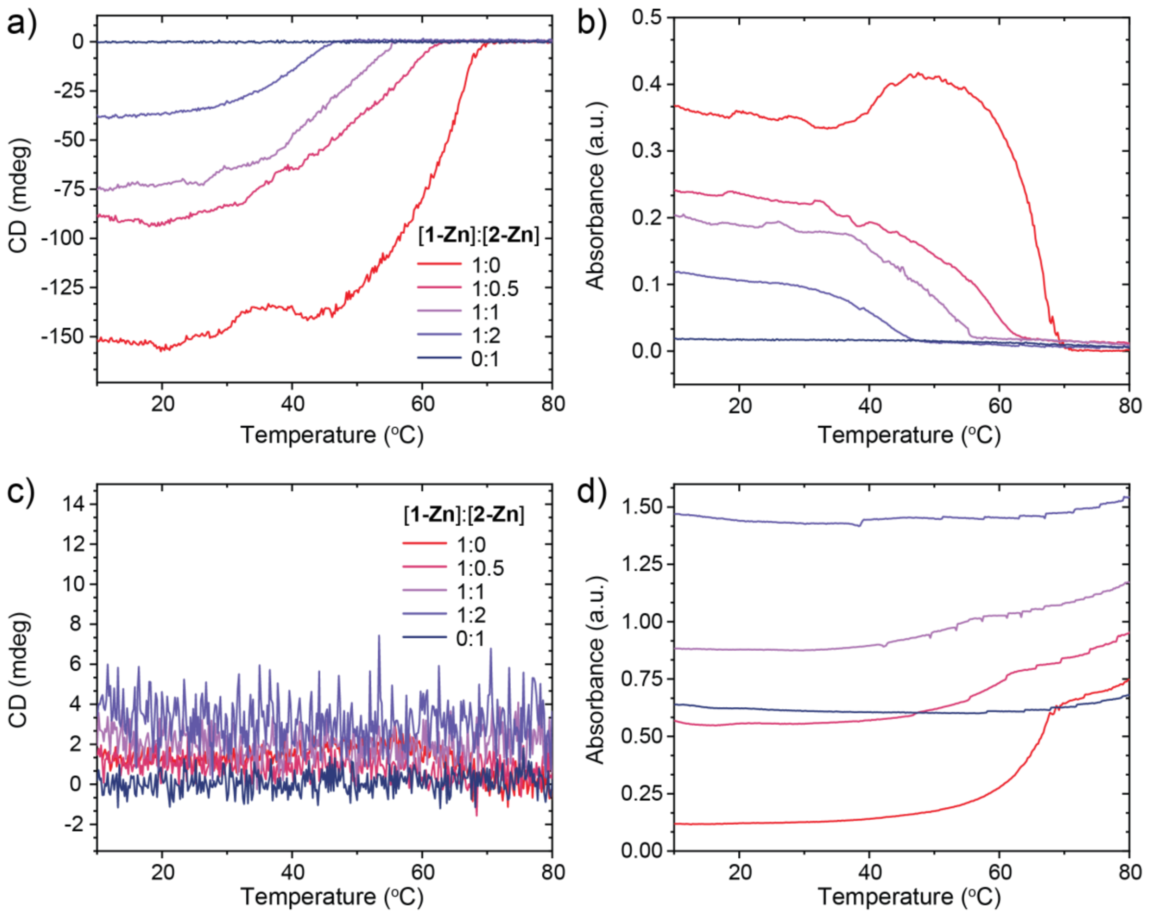

Figure S10: Melting curves of **1-Zn** and **2-Zn** and their mixtures followed by CD and UV at 390 nm (H-aggregate; a, b) and at 420 nm (J-aggregate plus monomer; c, d). The concentration of **1-Zn** and **2-Zn** was set to c = 25 µM in MCH, for mixtures in the respective ratios the concentration of **1-Zn** was kept constant at $c_{1\text{-Zn}}$ = 25 µM and aliquots of **2-Zn** were added to exclude dilution effects. Cooling rate was 1 °C/min.

Table S1: Temperatures of elongation (T_e) for homo- and copolymerization mixtures of **1-Zn** and **2-Zn**. Temperatures were taken from CD melting curves followed at 390 nm with a cooling rate of 1 °C/min ($c_{1\text{-Zn}}$ = 25 µM for pure samples and mixtures, $c_{2\text{-Zn}}$ = 25 µM for pure sample).

sample	T_e [°C]
**1-Zn** (1:0)	69
1:0.5	62
1:1	55
1:2	46
**2-Zn** (0:1)	-

6. Atomic force microscopy

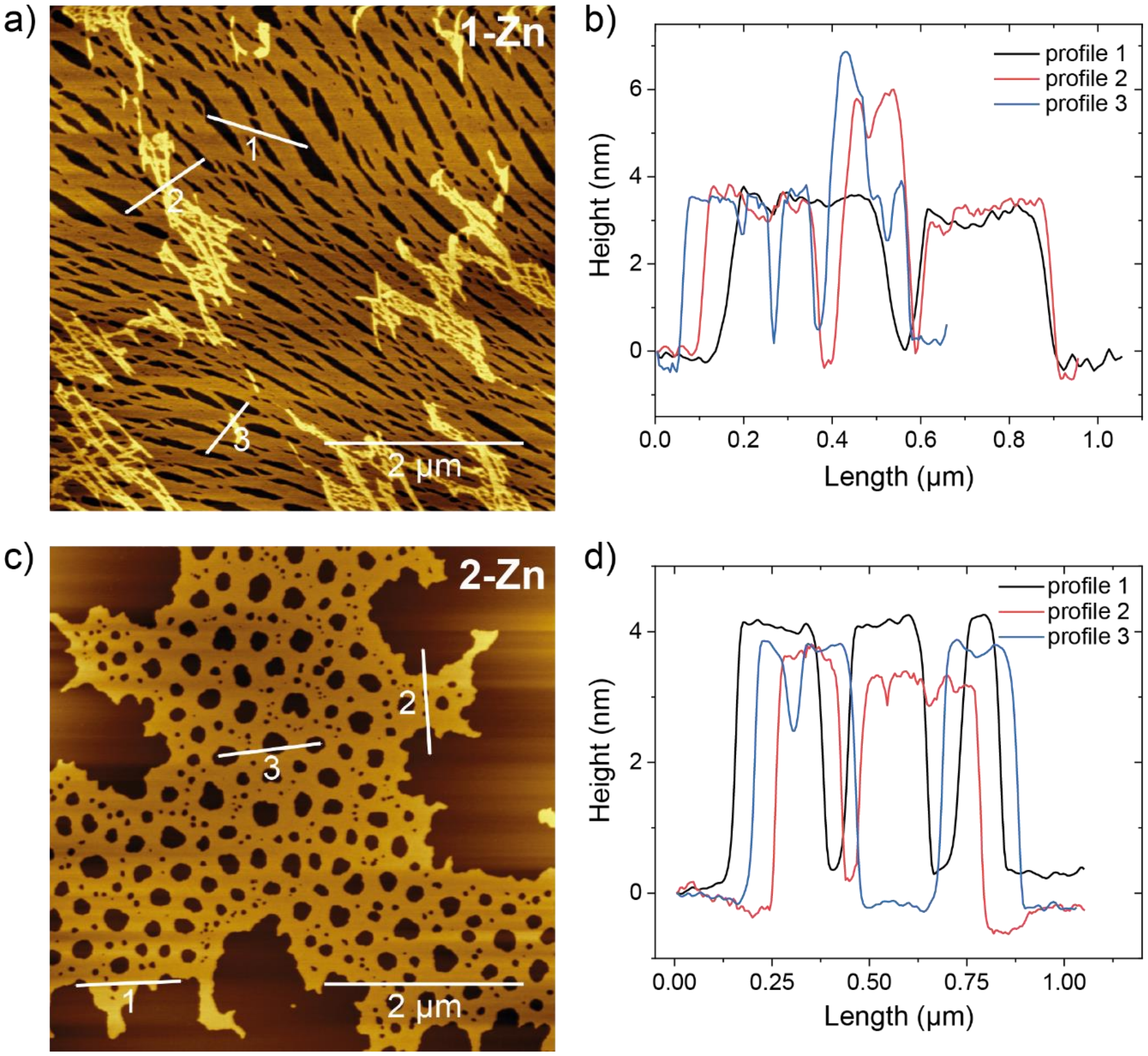

Figure S11: AFM images of **1-Zn** and **2-Zn** dropcasted from *c* = 20 µM solutions in MCH on mica. For **1-Zn** (a) bundled supramolecular fibers were found while for **2-Zn** (c) only disordered deposits were observed. Height profiles of b) **1-Zn** and d) **2-Zn** shows structures of 3-4 nm thickness in both cases.

7. Density functional theory calculations of monomers

All DFT calculations were performed using the Vienna Ab Initio Simulation Package (VASP).[3–6] The Perdew–Burke–Ernzerhof (PBE) exchange-correlation functional[7,8] was used in conjunction with the projector augmented wave approach.[9,10] All structures were optimized to their local minima using the conjugate gradient algorithm as implemented in VASP. Optimization and other electronic settings are given below.

Settings

Parameter	INCAR setting	value
Cutoff energy	ENCUT	500
Precision	PREC	High
Smearing type	ISMEAR	0 (Gaussian smearing)
Smearing width	SIGMA	0.0005
Electronic convergence threshold	EDIFF	1E-5
Ionic relaxation threshold	EDIFFG	1E-4

K-point grid

For all calculations, only the Γ-point was used.

Geometries

Below, the geometries of the optimized structures are given. These geometries are represented as POSCAR/CONTCAR files. Further information with regard to the structure of these files is provided in the link below: http://cms.mpi.univie.ac.at/vasp/guide/node59.html.

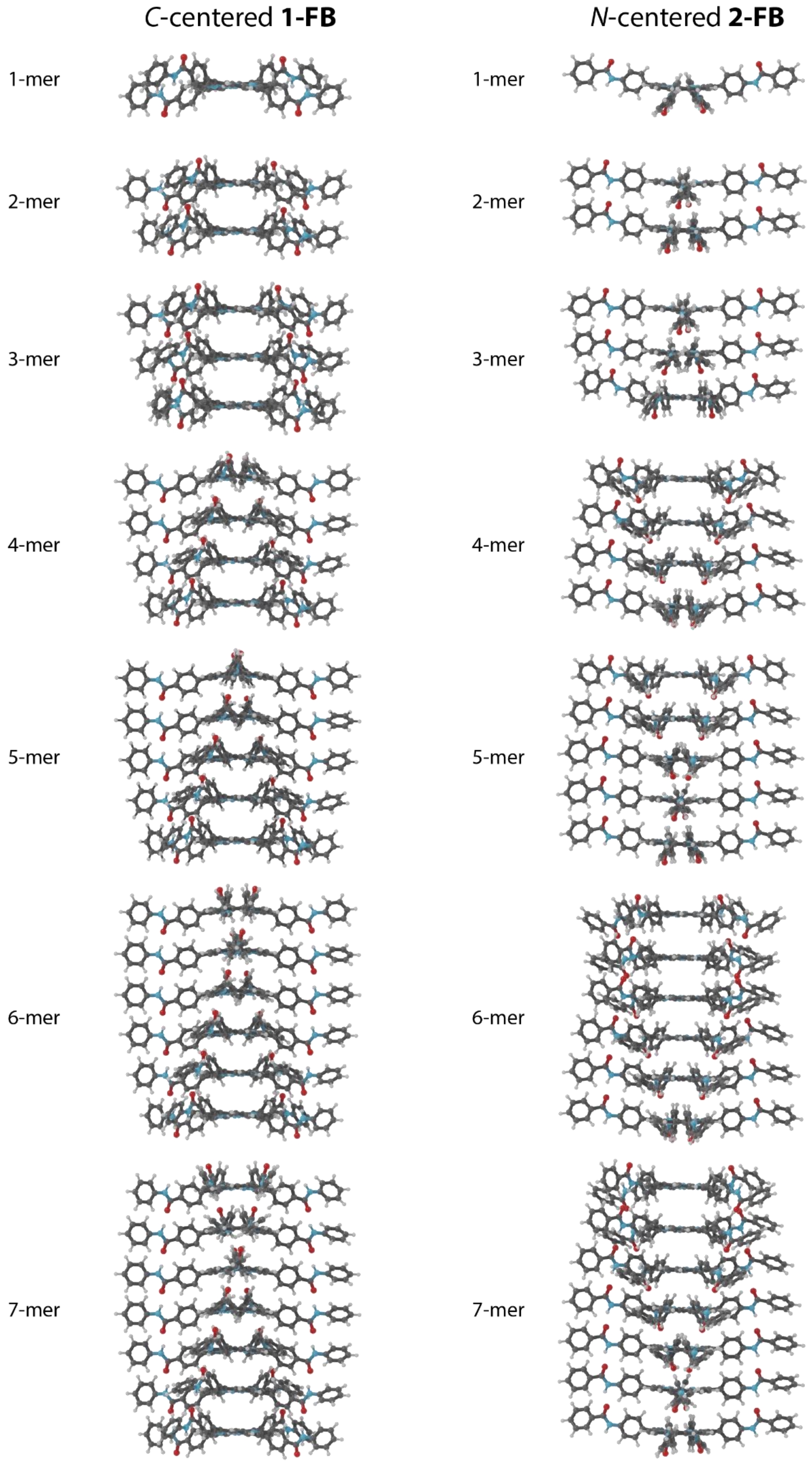

Figure S12: Images of optimized structures of mono- up to heptamers of *C*- and *N*-centered ligands.

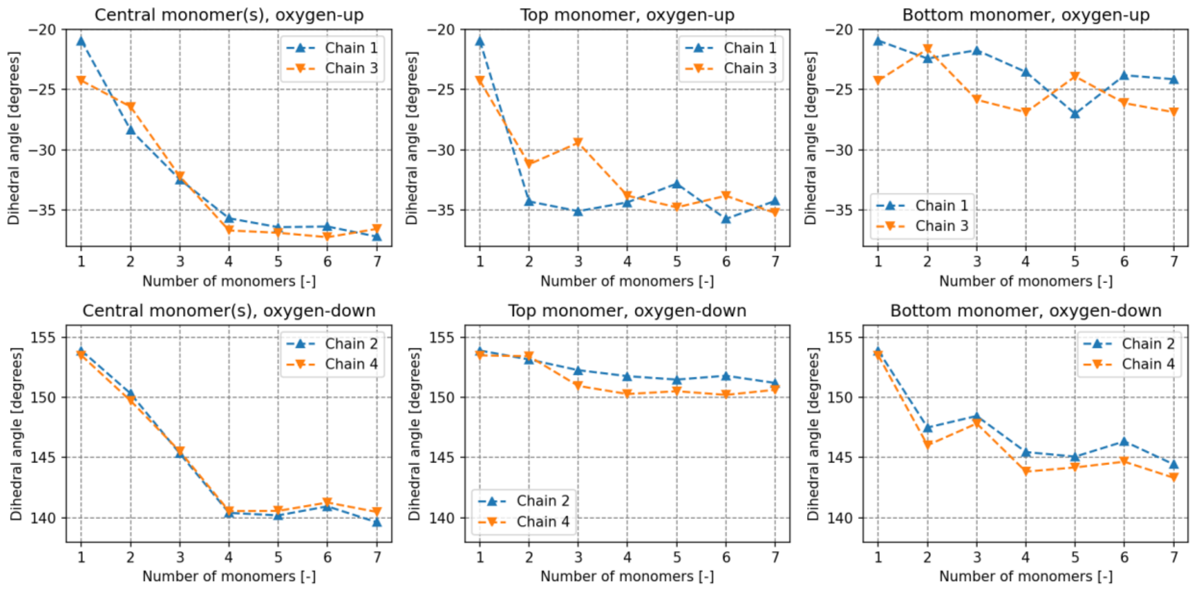

Figure S13: Dihedral angles of **1-FB** monomers in the stacks oriented either oxygen up or down.

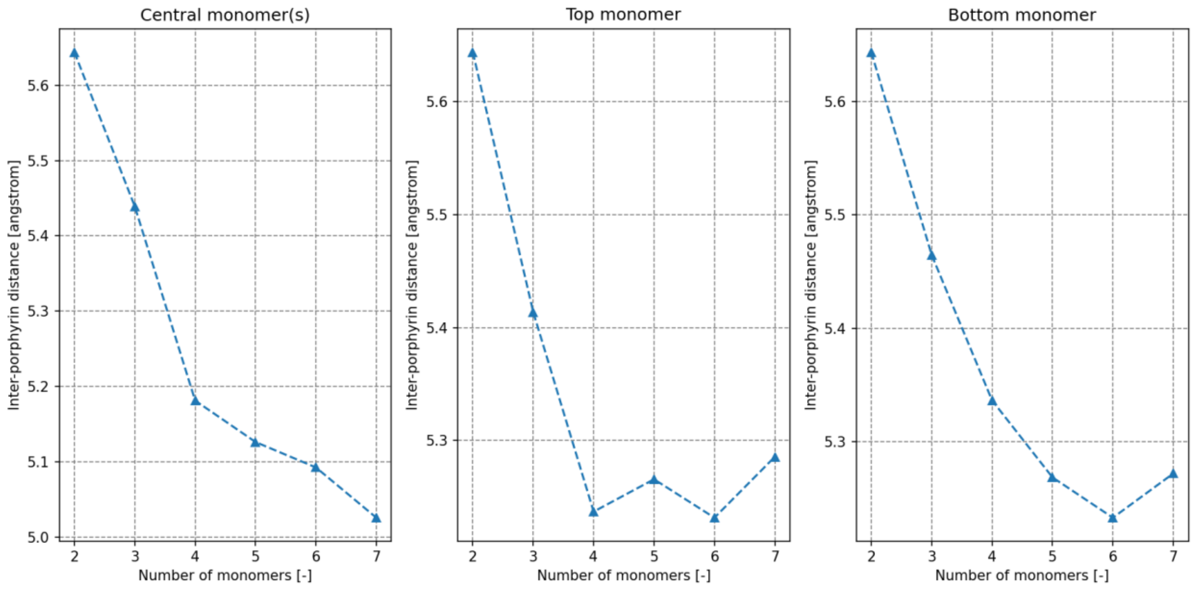

Figure S14: Distance between central, top or bottom monomers in the **1-FB** stack.

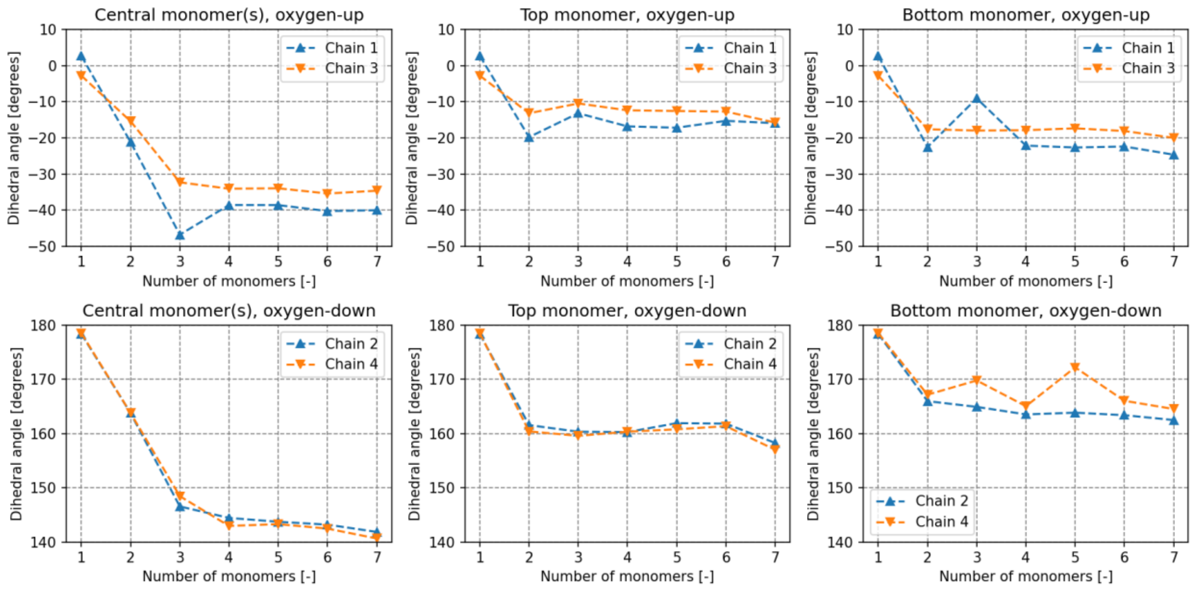

Figure S15: Dihedral angles of **2-FB** monomers in the stacks oriented either oxygen up or down.

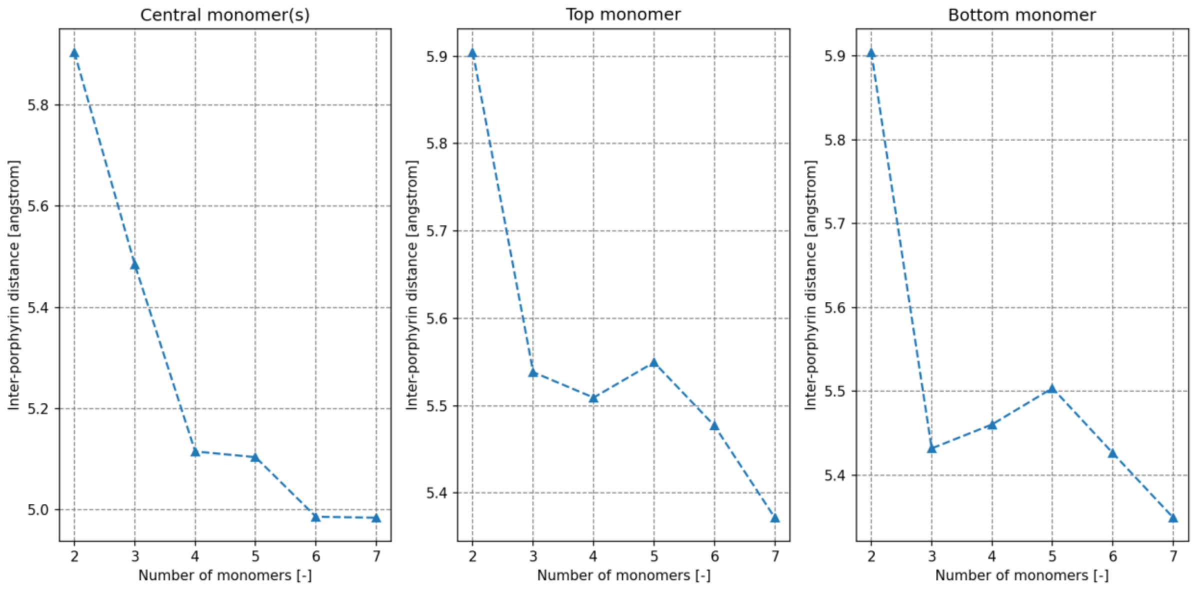

Figure S16: Distance between central, top or bottom monomers in the **2-FB** stack.

8. Spectra of Compounds:

8.1 ^{1}H NMR spectra:

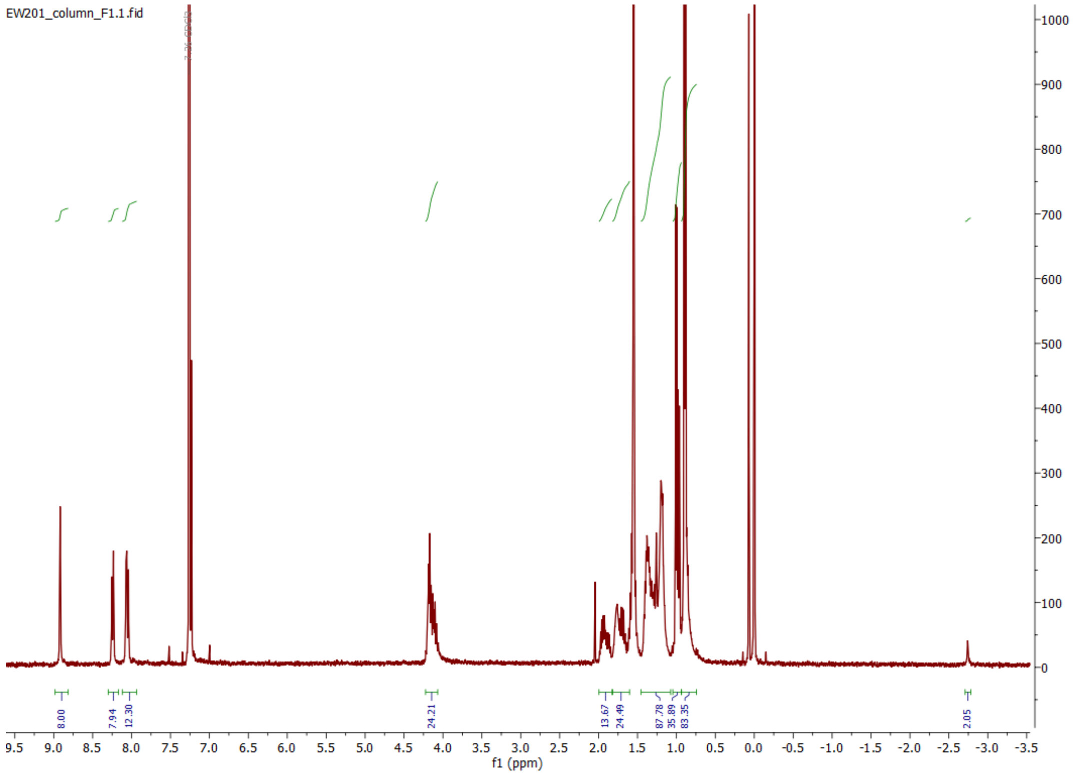

Figure S17: ^{1}H NMR spectrum of **2-FB** in chloroform-*d*.

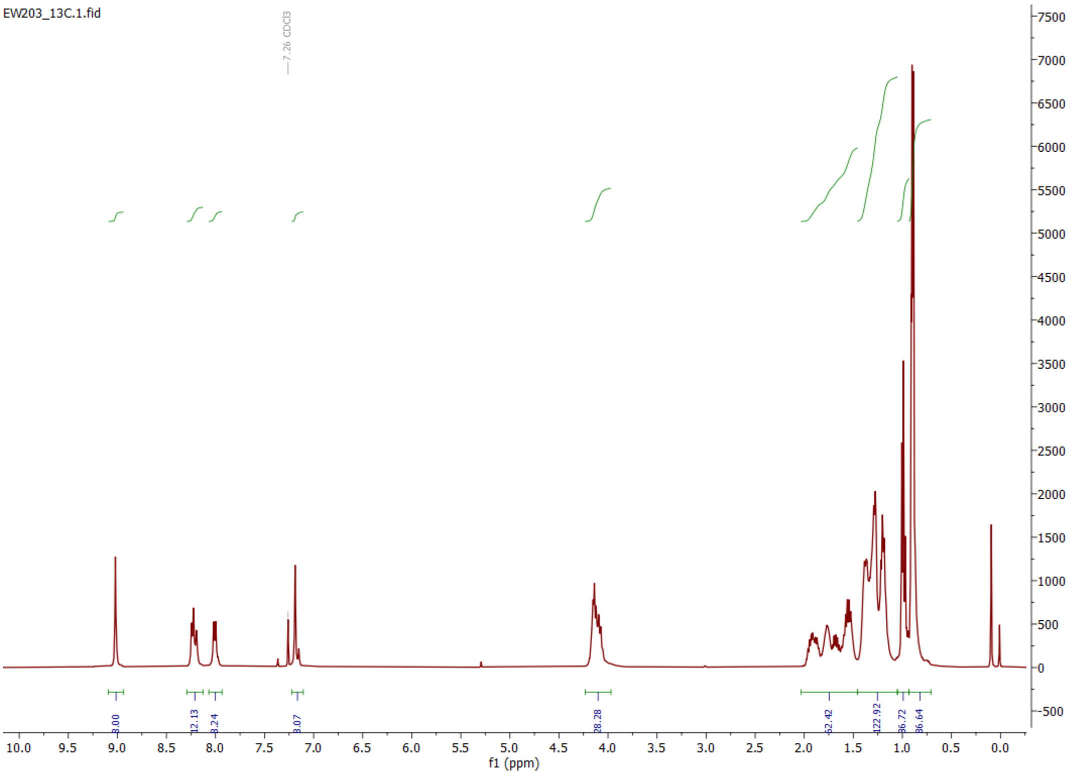

Figure S18: ^{1}H NMR spectrum of **2-Zn** in chloroform-*d*.

8.2 ^{13}C and 2D NMR spectra:

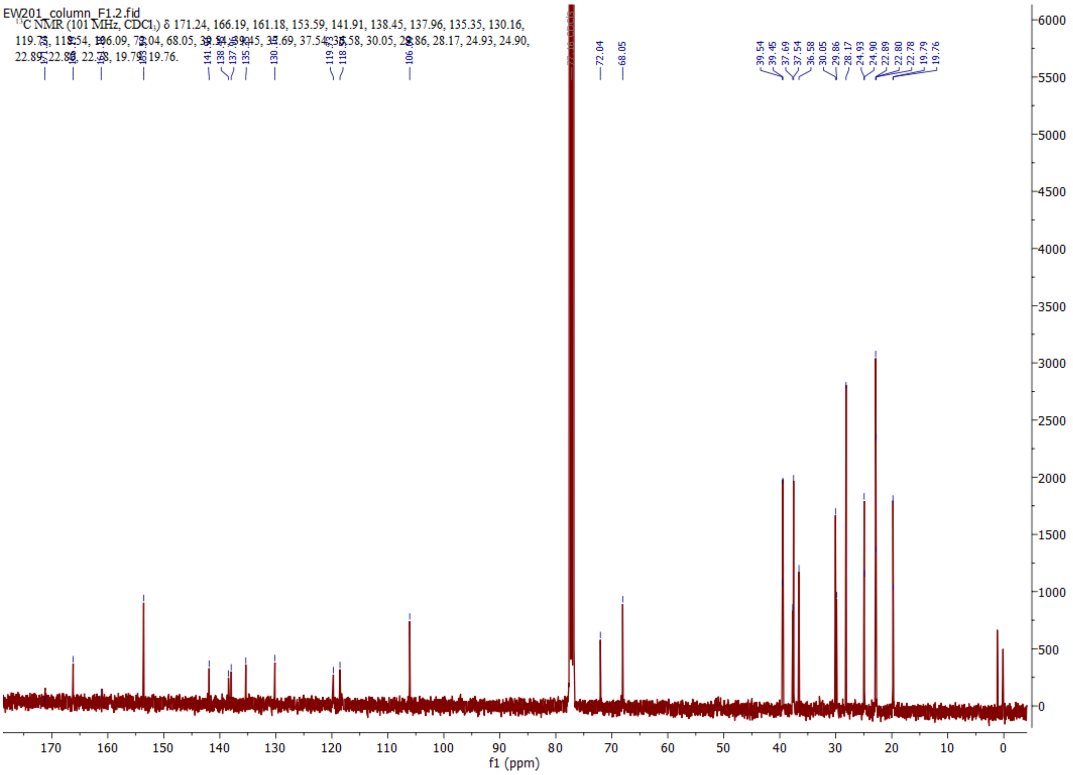

Figure S19: ^{13}C NMR spectrum of **2-FB** in chloroform-*d*.

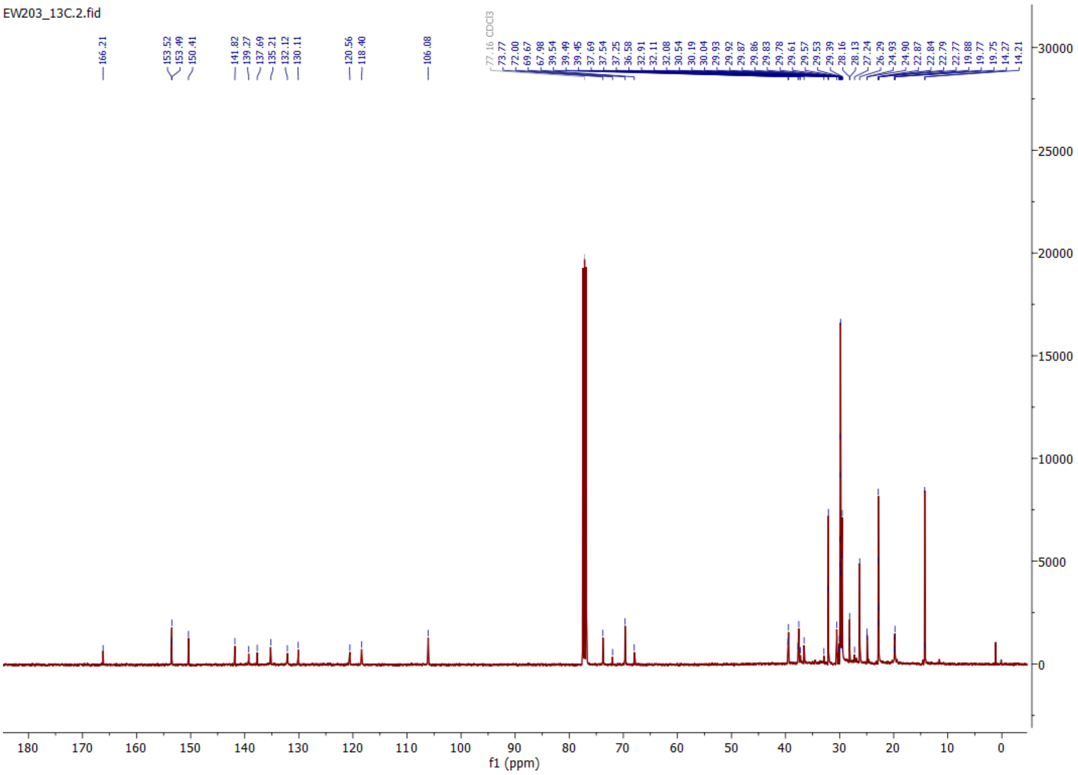

Figure S20: ^{13}C NMR spectrum of **2-Zn** in chloroform-*d*.

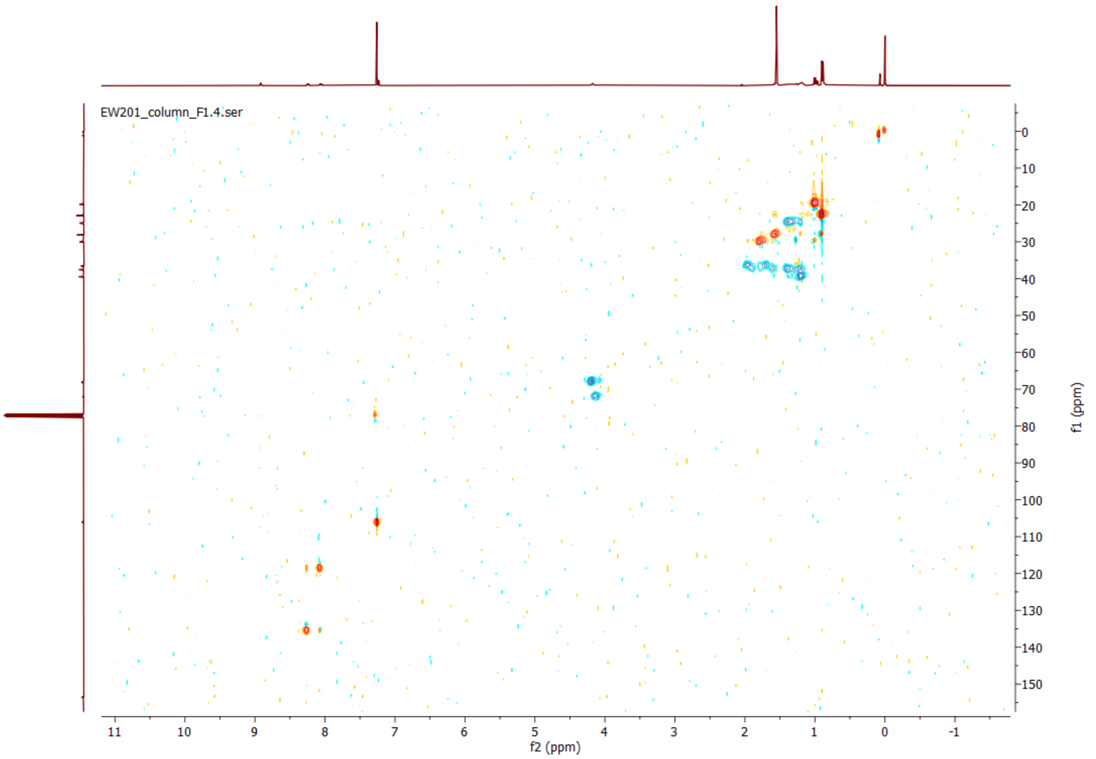

Figure S21: COSY NMR of **2-FB** in chloroform-*d*.

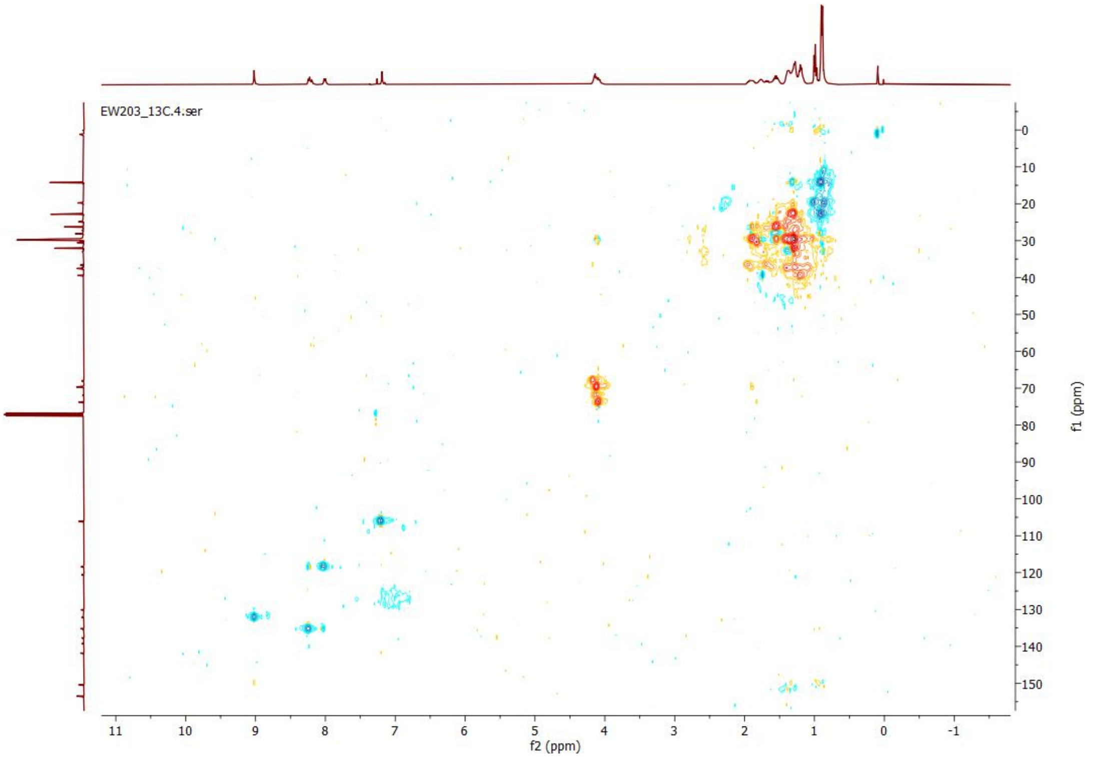

Figure S22: COSY NMR of **2-Zn** in chloroform-*d*.

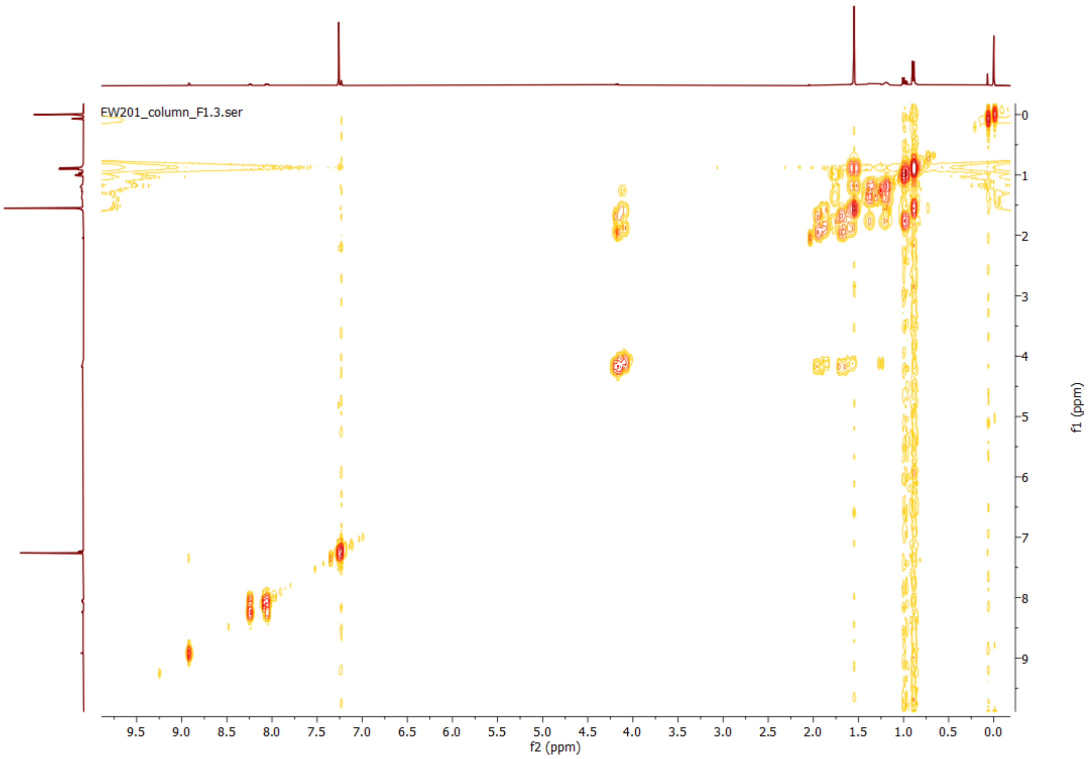

Figure S23: HSQC NMR of **2-FB** in chloroform-*d*.

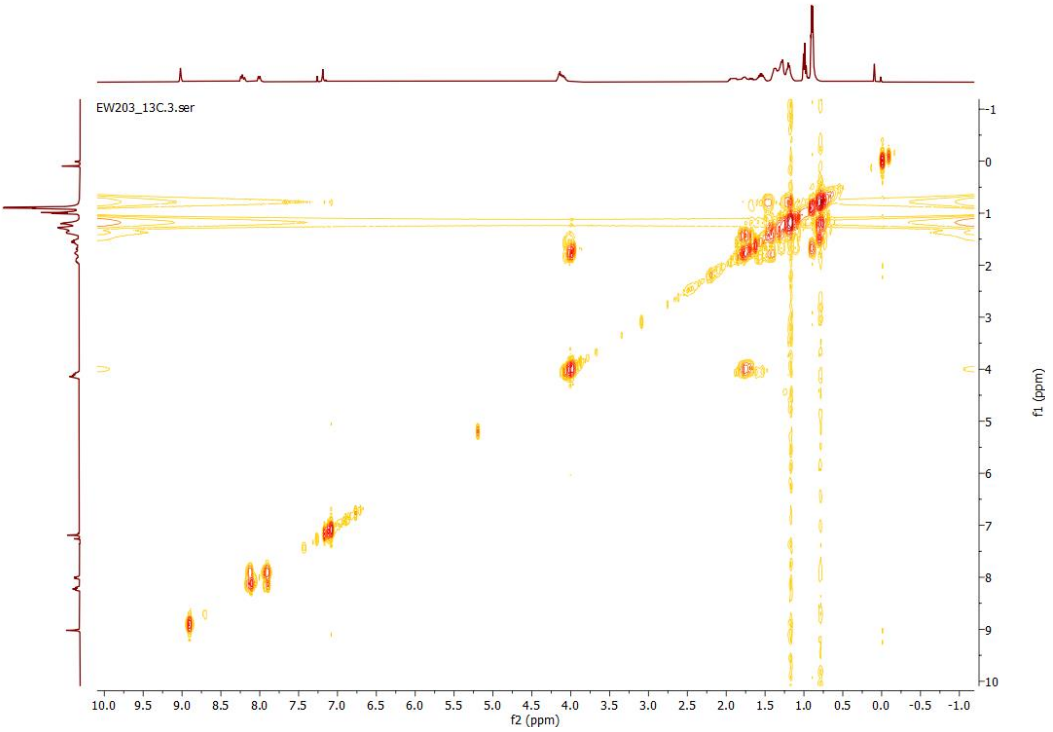

Figure S24: HSQC NMR of **2-Zn** in chloroform-*d*.

8.3 MALDI-TOF:

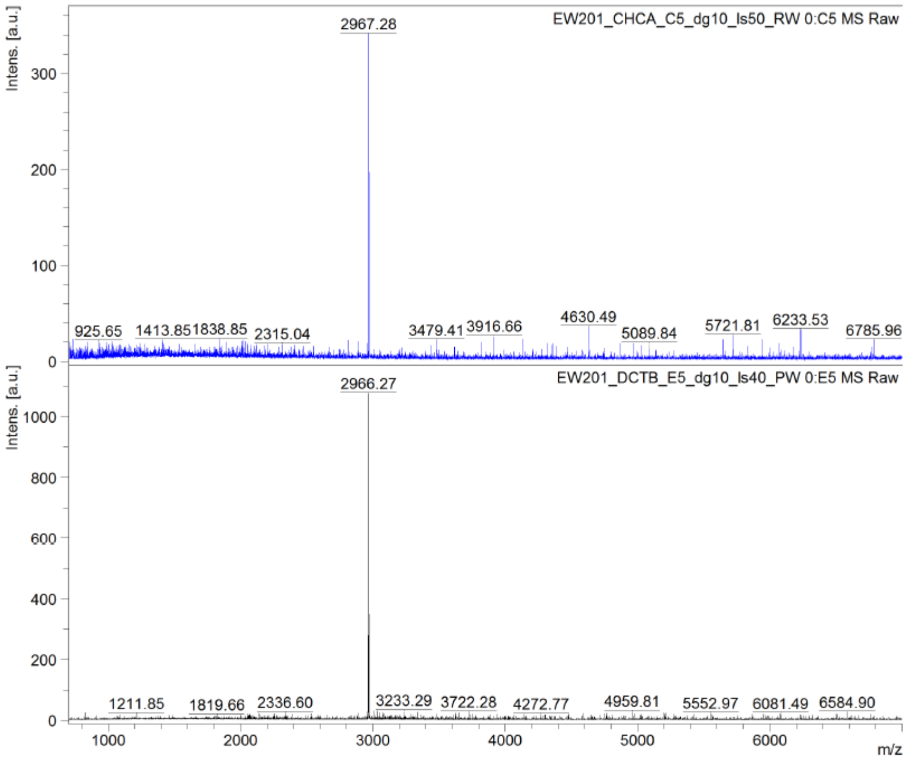

Figure S25: MALDI-TOF spectrogram of **2-FB**.

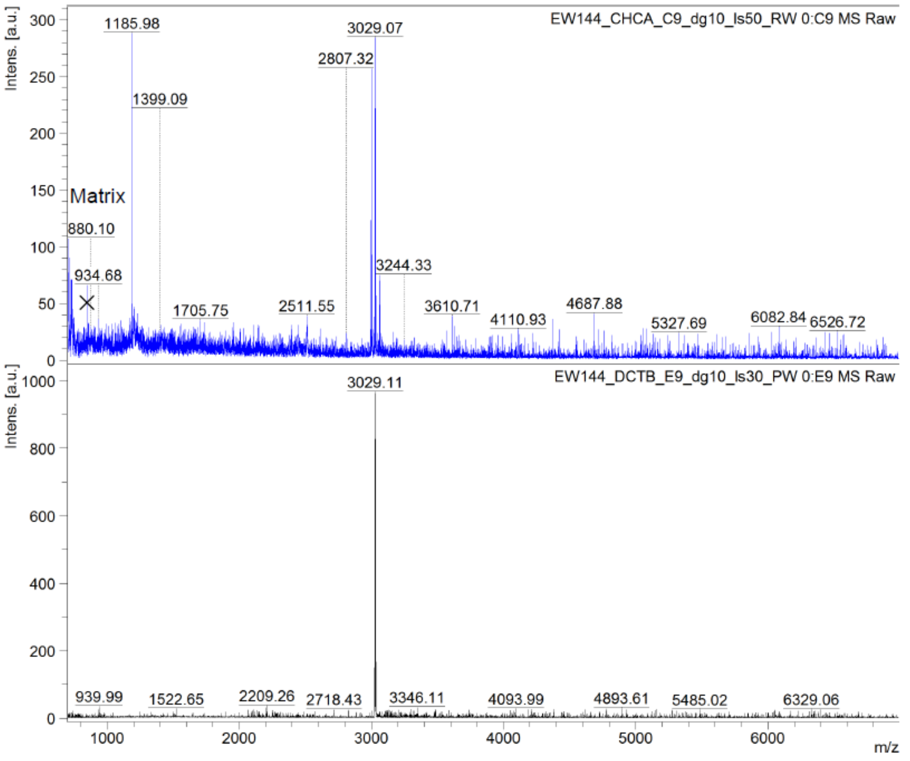

Figure S26: MALDI-TOF spectrogram of **2-Zn**.

9. References

(1) Helmich, F.; Lee, C. C.; Nieuwenhuizen, M. M. L.; Gielen, J. C.; Christianen, P. C. M.; Larsen, A.; Fytas, G.; Leclère, P. E. L. G.; Schenning, A. P. H. J.; Meijer, E. W. Angew. Chemie Int. Ed. **2010**, *49*, 3939.

(2) Lian, W.; Sun, Y.; Wang, B.; Shan, N.; Shi, T. J. Serbian Chem. Soc. **2012**, *77*, 335.

(3) Kresse, G.; Hafner, J. Phys. Rev. B **1993**, *47*, 558.

(4) Kresse, G.; Hafner, J. Phys. Rev. B **1994**, *49*, 14251.

(5) Kresse, G.; Furthmüller, J. Comput. Mater. Sci. **1996**, *6*, 15.

(6) Kresse, G.; Furthmüller, J. Phys. Rev. B **1996**, *54*, 11169.

(7) Perdew, J. P.; Chevary, J. A.; Vosko, S. H.; Jackson, K. A.; Pederson, M. R.; Singh, D. J.; Fiolhais, C. Phys. Rev. B **1992**, *46*, 6671.

(8) Perdew, J. P.; Chevary, J. A.; Vosko, S. H.; Jackson, K. A.; Pederson, M. R.; Singh, D. J.; Fiolhais, C. Phys. Rev. B **1993**, *48*, 4978.

(9) Blöchl, P. E. Phys. Rev. B **1994**, *50*, 17953.

(10) Kresse, G.; Joubert, D. Phys. Rev. B **1999**, *59*, 1758.

10. Appendix: DFT coordinates

```
C-CENTERED MONOMER
   1.00000000000000
    35.1161850000000015    0.0000000000000000    0.0000000000000000
     0.0000000000000000   32.8993200000000030    0.0000000000000000
     0.0000000000000000    0.0000000000000000   16.8059690000000010
   C    H    N    O
    72    50     8     4
Direct
  0.1880132670772022  0.7385597222002495  0.3652374573483335
  0.2036911045243609  0.6880265058295854  0.4936222662191727
  0.1688977844080555  0.7082034421765782  0.4894310472012576
  0.6592707537388143  0.7165112611714222  0.6193802523643850
  0.7141455556359159  0.7599099955319474  0.5735581510842449
  0.7161934242745622  0.7862088743140000  0.6393277937017402
  0.7448569989964373  0.8155830436255276  0.6421503680448980
  0.7410111253955382  0.7637065533290091  0.5118838026205981
  0.7694303182945816  0.7931501382361806  0.5157193826557057
  0.7715568262133876  0.8193519682186854  0.5810316420769357
  0.5418766603703898  0.4194045614180992  0.4875844641891928
  0.4972337838326614  0.3705594270479754  0.5014369805873689
  0.4238090712210153  0.4565028290181314  0.5103034759583398
  0.4153100470366424  0.5236845844007636  0.4879228047325040
  0.3784992281606315  0.5048433643747110  0.4906591497646944
  0.3836271274418574  0.4641762402526743  0.5058956603651800
  0.5860817078899290  0.4789616090433301  0.4908348505923667
  0.5773085625853154  0.5459183380703836  0.5155927914994900
  0.6174876493290090  0.5380883659973066  0.5152792619213381
  0.6228367667809707  0.4975697412976192  0.4989006646665863
  0.4596448402243894  0.5830456678138413  0.4891152338869726
  0.4660610182559700  0.6267868136578170  0.4923904168626388
  0.5036490497034543  0.6319232466737713  0.5092552667471992
  0.4810633185693518  0.4111830475813285  0.5084593094970670
  0.5349439616256487  0.3757107712841731  0.4858676484879181
  0.5201600445759923  0.5912984760517931  0.5127604135291957
  0.5594081684060063  0.5839033904650333  0.5237228437834782
  0.4233281660691776  0.5653203436867713  0.4795339008326208
  0.4416921713222526  0.4184869888520685  0.5178772072207368
  0.5783249862789828  0.4372930733099031  0.4800376600642740
  0.5847766639348283  0.6187641538847971  0.5452366627435330
  0.6001864028806142  0.6448977182205273  0.4877313438409038
  0.6245266555676163  0.6765245828699807  0.5098248116490781
  0.6338089605591304  0.6829621546431219  0.5900726550877898
  0.6177571492735769  0.6573526133357170  0.6477384334325911
  0.5939008951331878  0.6255328050499529  0.6255477300224186
  0.6116926823193387  0.4113574406331210  0.4591793993365563
  0.6254560350343005  0.3805503379201396  0.5093697615831240
  0.6571314213297980  0.3574097206195827  0.4877492109347342
  0.6758345086164422  0.3641909106444351  0.4151168378508626
  0.6617154539514424  0.3944876661211687  0.3643052807963614
  0.6304787877862233  0.4178863681055238  0.3863214036587334
  0.4161611195156232  0.3833577153076898  0.5368815352053333
  0.4064428957739484  0.3757964422419566  0.6166073404989239
  0.3825776120724168  0.3436509152831645  0.6368274350172823
  0.3671035460155663  0.3184945159380631  0.5777142083120982
  0.3769842472341946  0.3257209982391858  0.4980363453088239
  0.4013304583096214  0.3576918083641633  0.4779093571349246
  0.3903248373271397  0.5921595911408672  0.4591767867005063
  0.3799616871973814  0.5970678992101902  0.3790042174756086
  0.3492468935427011  0.6214782475010605  0.3577486574874660
  0.3274868294333605  0.6411434765356658  0.4161755385814241
  0.3379291022761880  0.6366120787945652  0.4962965435175186
  0.3690685289788253  0.6125098370547420  0.5174994388750968
  0.1608022862621165  0.7336692861506622  0.4250391561333302
  0.7098246413314505  0.3406777823820153  0.3860391595562406
  0.7644817544042052  0.2986379576295790  0.4374166826203070
  0.7801217649221430  0.2827398358651215  0.5079492197135781
  0.8131152026777826  0.2592987893875899  0.5057376575739041
  0.8311204794043348  0.2513389604128279  0.4332046118129909
  0.8155844573426121  0.2671535154333721  0.3632861509981073
  0.7824986667567609  0.2906753951591676  0.3643716526427189
  0.3414823030041711  0.2846285355182323  0.6050119840385301
  0.2868762753804730  0.2416682025408954  0.5562502309856086
  0.2603418581391064  0.2386128439158453  0.4937959280566119
  0.2316315480791100  0.2094563281853700  0.4960495318565753
```

0.2842316705649242	0.2149098377352149	0.6212045170703857
0.2552886905888060	0.1858279529745525	0.6224498264080974
0.2289039504923512	0.1828043996980720	0.5605676770718864
0.2947624506169045	0.6667458286112865	0.3876008730696999
0.2310684130151331	0.6929541044061488	0.4332647666936464
0.2230332535331334	0.7185423025324589	0.3684425641770884
0.4445252150009478	0.6501427154032859	0.4841732121410318
0.3771029449772459	0.6094919704320680	0.5799947015792346
0.4815784070359574	0.3421529364178105	0.5058531098306021
0.3517473470412604	0.5208878488695895	0.4828876397779586
0.3617894369216587	0.4410545385868792	0.5121940936612562
0.5189667260032108	0.6603212911471644	0.5174906080918890
0.6392300751999133	0.5609564481482773	0.5254740921746979
0.6497100141234969	0.4814642436024616	0.4942542146365299
0.5929578599882679	0.6404310830175691	0.4251402232006767
0.6352911386710723	0.6968748944477398	0.4635623330145413
0.4704589103011493	0.4977927282069008	0.5007561417427514
0.6244559387338037	0.6630418543308176	0.7101573751685034
0.5819052346579137	0.6055031018448163	0.6709055726582406
0.6113302674001923	0.3748851438603090	0.5661669424904667
0.6663850603711152	0.3332195051657543	0.5279773456631351
0.5307998980562640	0.5050245152047514	0.4994909951820616
0.6758288095372519	0.3990527374461353	0.3071416036129498
0.6199515182414945	0.4415053844383144	0.3463166126341887
0.4179592842915294	0.3954680552062777	0.6630953605011135
0.3754369846384567	0.3373384142337719	0.6988004097398149
0.3414362582402648	0.6258367260057884	0.2954870105794017
0.3667165543915754	0.3057207584532086	0.4507216599687536
0.4090022956093953	0.3627901755538535	0.4157346783548684
0.3964239508487832	0.5815276017472644	0.3330137960643849
0.1335421185395536	0.7495001757478086	0.4216007583562197
0.2064199156283564	0.1599260094659443	0.5624940691114172
0.7226734672902216	0.3273882181934185	0.5015551471656692
0.7661321916869713	0.2887878901334442	0.5648938312747294
0.8247657308249671	0.2471377724708803	0.5610620228126428
0.8569545923360159	0.2329335681272647	0.4313148132267012
0.8293879979235486	0.2611794078255663	0.3062321106344619
0.7704050922381085	0.3029645122326069	0.3099139320304946
0.3140920236709835	0.2883034630689665	0.4983129215198940
0.2622517900203442	0.2594648772235573	0.4432285524401353
0.2112662536414655	0.2076099055206631	0.4471058256622364
0.3045974378606109	0.2174230048561961	0.6696939607878143
0.2534528380078158	0.1651596652224839	0.6731441710208331
0.2679712202509858	0.6553042921069009	0.4928858884762180
0.2440761807320890	0.7221289184586839	0.3215180944648830
0.1820435878168343	0.7583667903311320	0.3146212864597479
0.2097967536513548	0.6680319275679939	0.5440664894784597
0.1479813071405846	0.7039193267708991	0.5367375732222687
0.6876817366335185	0.7133910854745262	0.5137831133372979
0.6955987580082003	0.7830987629458689	0.6872531603993084
0.7462090854442291	0.8358568692530810	0.6935176353588440
0.7395213411607723	0.7432858438716096	0.4605925749246416
0.7900362811787850	0.7955988383906752	0.4672963027115429
0.7938009277458649	0.8424484973386324	0.5841673152720216
0.5560408793086544	0.3522530496577068	0.4746777203682344
0.3226030373145354	0.6528104565923369	0.5432255006861310
0.2656337815214184	0.6717227280859344	0.4418329039124106
0.6861440538463482	0.7295908854506893	0.5652439547627500
0.5086633378888025	0.4403504069338736	0.5002529249228783
0.4416509880894430	0.4933186615266834	0.4997144827775865
0.5596225993717135	0.5093064189935625	0.5012274613010566
0.4929164925331769	0.5620975448192855	0.5008434906590359
0.7311382407906127	0.3221214823463343	0.4446029275613085
0.3151556261703128	0.2718162307112026	0.5494696319263931
0.7177895776825580	0.3391317301460123	0.3144759676636663
0.3439925809007342	0.2703686965953717	0.6728179757697006
0.2943940161888502	0.6813105659491825	0.3198665532106995
0.6561374998609892	0.7302926066937193	0.6874439278231749

```
C-CENTERED DIMER
   1.00000000000000
    37.7404459999999986    0.0000000000000000    0.0000000000000000
     0.0000000000000000   35.6461979999999983    0.0000000000000000
     0.0000000000000000    0.0000000000000000   21.8059699999999985
   C    H    N    O
   144   100    16     8
Direct
  0.2050685932769446  0.7150733810465413  0.2959506339868013
  0.2222844688671591  0.6636896103689912  0.3872511959730806
  0.1882899348660864  0.6787917201065272  0.3845049373418750
  0.6483701570139953  0.7058963957883291  0.4494722246943860
  0.6870289292575582  0.7560234892091841  0.4026432203205452
  0.6854151559206221  0.7829971684714546  0.4494020575820920
  0.7066278578197406  0.8149820547449265  0.4454490440892517
  0.7098966591636964  0.7615902571181067  0.3525964511543557
  0.7308924911856638  0.7936513090636528  0.3492607291979447
  0.7294176059750431  0.8205503042413932  0.3958201307925807
  0.5391860849522393  0.4266034400218829  0.3638806062382229
  0.4977165197508563  0.3814722734926517  0.3742437351164651
  0.4294636837959439  0.4614638778973831  0.3762558770701111
  0.4230301639718116  0.5240520347377025  0.3629374505463548
  0.3884435126451557  0.5073662736407367  0.3623694030332665
  0.3923109131777160  0.4694211803805819  0.3712540147654120
  0.5809910226874030  0.4813102249707538  0.3648100895946442
  0.5743287806314535  0.5437207064702273  0.3804435620934128
  0.6115306455813688  0.5356547149694901  0.3788398963448286
  0.6155802496404343  0.4978471682240234  0.3688321654363855
  0.4649143702696185  0.5785338184853203  0.3653088935047280
  0.4705476939711419  0.6188046174079533  0.3709237261334614
  0.5057425466856249  0.6235909820530809  0.3815878490358378
  0.4822450393208969  0.4189347336735098  0.3757983024817213
  0.5330403892567821  0.3862286067137344  0.3649772767785152
  0.5215786225620240  0.5862500256295066  0.3798816719898540
  0.5582987099114096  0.5792377003753674  0.3861749297627469
  0.4309767988206576  0.5624814555022746  0.3586304083532045
  0.4454480251033814  0.4258805591422268  0.3808792385682673
  0.5732534468911853  0.4428024829521936  0.3589454359645629
  0.5823547081077290  0.6113343152822273  0.3998698688614084
  0.5866646561507139  0.6413755972438913  0.3588459349503835
  0.6084228082063168  0.6716926488181921  0.3735293586061810
  0.6263230304612712  0.6730129304845643  0.4299502410474909
  0.6222765487054460  0.6429882167517552  0.4708748173264818
  0.6009522780270351  0.6124922939062137  0.4557831662314614
  0.6044202985105279  0.4178263739719255  0.3482657803098109
  0.6160128861615556  0.3921160302629749  0.3926853746374027
  0.6458539347648278  0.3698432321961771  0.3825235420158118
  0.6646914716683091  0.3723127586101360  0.3272222232589904
  0.6527538190376555  0.3974769266990559  0.2822608950247110
  0.6233734251631947  0.4201427263277928  0.2928381791270062
  0.4210666311416731  0.3935760999566696  0.3914279180949174
  0.4020065693167861  0.3910783993113792  0.4467381848865551
  0.3801260153356572  0.3605442136190009  0.4588979458111285
  0.3759831298162895  0.3318082739886571  0.4155760979537137
  0.3944287047457878  0.3344475628364067  0.3598017819644728
  0.4167295093093014  0.3648164100351465  0.3480354286962995
  0.4001663423216695  0.5880095165323608  0.3471863695321056
  0.3905059484905120  0.5971539572929334  0.2868449175601434
  0.3611730424742571  0.6197626652805127  0.2753191076833493
  0.3403056180353353  0.6333537640652215  0.3237089038166115
  0.3502658467500294  0.6249053013603371  0.3841425774833797
  0.3799952167737071  0.6026525170104502  0.3955617831340444
  0.1793893291113432  0.7045861283309248  0.3387326513445532
  0.6959596004423777  0.3480593119384645  0.3111388815823621
  0.7424738441594089  0.3075298361760223  0.3595274442371136
  0.7548367514262473  0.2948179578162431  0.4167705031198653
  0.7820760407510219  0.2685628570085630  0.4201310677526914
  0.7974545678548425  0.2544068863792469  0.3665893208882025
  0.7851485378047606  0.2669942717365567  0.3098244852120500
  0.7579225976330902  0.2933975710211472  0.3055535899836178
  0.3532741932666127  0.2988145799783797  0.4322058879765422
  0.3149241196427943  0.2499375589587263  0.3814742636355843
  0.2925085521891732  0.2455435816049191  0.3305196521885660
  0.2711262752267776  0.2138631596703390  0.3251527108930991
  0.3157681124324712  0.2221086012514600  0.4269627747968608
  0.2941617857843102  0.1905405969167597  0.4210023277591818
  0.2717507057322002  0.1861850536857746  0.3705398295239040
```

0.3085199603155868	0.6565142888612728	0.3064268751391604
0.2480666668765385	0.6741797684211145	0.3439102123127314
0.2392760555465086	0.7002155799206924	0.2979372381449709
0.1898511370354233	0.6923224208039631	0.5627036105856134
0.2083961619013949	0.6430831373493204	0.6563195177645805
0.1765025092978509	0.6626447604934768	0.6593629128588797
0.6176786646920094	0.7212968159287045	0.7007586292235038
0.6628544514967810	0.7670305536410723	0.6630872368665562
0.6604071029985246	0.7933398565166548	0.7109464975776858
0.6849356092776264	0.8225851942362227	0.7141762059539747
0.6899193986105282	0.7707406845333249	0.6191582807768395
0.7141647526144972	0.8001130887244480	0.6231100666248397
0.7119059095435418	0.8262398265327540	0.6707511597591005
0.5502758555399774	0.4309920717410113	0.6200456957540604
0.5166970373257538	0.3788225449479462	0.6275608938003738
0.4361981708424953	0.4443194768862381	0.6358097752444243
0.4175769048435239	0.5042160143199238	0.6235126315896736
0.3868745054723424	0.4805692131356411	0.6268419083547089
0.3981819746146854	0.4442658223296438	0.6349696602897060
0.5816981254939192	0.4927713815432664	0.6221219396219043
0.5631699494827478	0.5523393333952510	0.6383450218788437
0.6011848220866500	0.5523949849423250	0.6370175690678725
0.6124219547074912	0.5163092170332375	0.6264088342118855
0.4489914527143034	0.5660419253179018	0.6245213278268684
0.4487187766070205	0.6069794846551975	0.6246872950875284
0.4827345977835747	0.6179670253040341	0.6345954973623192
0.4955784311102389	0.4129074754574528	0.6325460968686605
0.5506879165072962	0.3900860115366643	0.6182547794921529
0.5037609611011946	0.5837532095092023	0.6377572326966259
0.5409931185683987	0.5838843318795881	0.6438630817963786
0.4182132208315565	0.5436277949900348	0.6189239649223415
0.4583727807828425	0.4126527124515251	0.6389786673118850
0.5809967841526704	0.4535066332850827	0.6153650100441942
0.5594616604603655	0.6202639255031425	0.6565178491141879
0.5676089388828096	0.6457999713703317	0.6096844800123855
0.5866904423836201	0.6785209151222011	0.6221546425970756
0.5974172117579342	0.6867785717058564	0.6822351893966091
0.5882505106656685	0.6618567883573582	0.7294440651720648
0.5699624257498770	0.6288717614950718	0.7166976746580294
0.6155994072924845	0.4349673919645031	0.6019378482171930
0.6323002161398026	0.4110426973880374	0.6441594292850078
0.6644505062524247	0.3937831382597707	0.6298733976372644
0.6805128583309501	0.3995625178743590	0.5725561226028172
0.6637207634852144	0.4231236616257393	0.5300811156326265
0.6320383339330639	0.4409351722258535	0.5449044779215573
0.4398293031312611	0.3760511915815747	0.6490268067212900
0.4281598416449117	0.3664145746178679	0.7081456332742686
0.4094603829421795	0.3333432949659113	0.7182381957012997
0.4010180210369147	0.3094047698416960	0.6692823398291413
0.4130648094099352	0.3185863190399710	0.6103221747148213
0.4325819686870562	0.3513592637312525	0.6005650094701078
0.3835732866914333	0.5623260258246904	0.6067865851244422
0.3657872288255485	0.5554920749610384	0.5511554445081660
0.3343457707879374	0.5739333993798197	0.5369667579914628
0.3191540389063097	0.5992370370804072	0.5785995457607247
0.3362634071627061	0.6054697147126913	0.6348754098744457
0.3680711524891934	0.5873804175745440	0.6486382685738863
0.1669983967679622	0.6873655278178482	0.6124232050127982
0.7145373010212137	0.3811823037026799	0.5534802927927794
0.7736565252371537	0.3627654585181960	0.5987932034366257
0.7907291783067419	0.3580801066568505	0.6556007132200552
0.8252203925953590	0.3443242669649702	0.6582291216945205
0.8431986310960534	0.3349871462964307	0.6043001818433822
0.8262318429977075	0.3397456237507330	0.5479469044198148
0.7916940829952830	0.3536201500837589	0.5445157828752405
0.3798315433446471	0.2749439837848888	0.6846220964703785
0.3345406229719561	0.2312110311120974	0.6414275889589072
0.3080932711959652	0.2294155835031672	0.5960947856537017
0.2832762709883719	0.2004909142246017	0.5965884955914935
0.3358268164340391	0.2034695005393331	0.6871996666204939
0.3107802258511612	0.1746576616754680	0.6869041628404526
0.2844074543378338	0.1728890730828320	0.6420716896362145
0.2862764986303514	0.6197664420557825	0.5594515364011030
0.2313267018042080	0.6480628634810215	0.6061820207075075
0.2219366917427924	0.6729621649183480	0.5590733359075403
0.4501021866511926	0.6402193390112513	0.3685711258319655
0.3876677472402954	0.5965127186044881	0.4427618302890229

0.4835326474652519	0.3551320434525883	0.3790645356544922
0.3639457836698021	0.5228253463648659	0.3559142966699800
0.3715106537567805	0.4484255278537054	0.3729419395276460
0.5195802587665103	0.6498214474012396	0.3898793819689133
0.6323403417355377	0.5564338833745607	0.3839239111585365
0.6402559287073807	0.4824092714711142	0.3648832142855460
0.5726013646593135	0.6409833384340345	0.3151092089846396
0.6103081435881936	0.6950950261420477	0.3413000825749083
0.4737853089772793	0.4981209902501907	0.3722884101964274
0.6358019473968823	0.6441275186188280	0.5150483255490422
0.5979033747537559	0.5893974237607744	0.4882909619011187
0.6016589242388948	0.3898208718474574	0.4359872745255446
0.6539608637021427	0.3501382824287788	0.4182172958079935
0.5300895855507760	0.5073746254390349	0.3706965118692545
0.6669910861321310	0.3986002155751399	0.2387425734507585
0.6145564898329564	0.4397867332119547	0.2575629404346150
0.4051037825873776	0.4131494571405108	0.4810727075469940
0.3662094659047389	0.3583308905244683	0.5026141962043845
0.3536137209899037	0.6271890124533682	0.2285677191470268
0.3925470513188192	0.3120357846874214	0.3257369640120530
0.4311896083558788	0.3662357555452780	0.3047480776170904
0.4061462083960317	0.5861884896992463	0.2487099463032879
0.1527026897377472	0.7163065849825264	0.3364005769397441
0.2549610214773629	0.1613896123277646	0.3664396135822221
0.7084946708439722	0.3441821028301212	0.4025582593875570
0.7428321915745011	0.3058274364991768	0.4586545840073720
0.7912302978845497	0.2590646591162080	0.4650393593806773
0.8187522970512952	0.2337499864935329	0.3691845931340060
0.7969145378002868	0.2561737982588847	0.2676176222010622
0.7484226299627238	0.3032153221536967	0.2613029612763944
0.3380501876482009	0.2966088861421724	0.3429698832731529
0.2918367166719160	0.2671874839614572	0.2949802570853782
0.2538465262814580	0.2108813126970906	0.2852926015307502
0.3331818614601054	0.2252162809297112	0.4663555382712174
0.2951220984911297	0.1690372104169219	0.4566264116589740
0.2852493975285221	0.6419664155382209	0.3881886544934761
0.2591168819922297	0.7081579927695184	0.2642164608180201
0.1984981072411549	0.7352252275782436	0.2599832211898456
0.2291775219283969	0.6435505694542824	0.4231131833284411
0.1685900734962888	0.6701074123064070	0.4183356597870945
0.6651057430753067	0.7096826503679807	0.3612628903210923
0.6677185817344278	0.7789164007635390	0.4881739720486175
0.7050802887589359	0.8358617443728897	0.4819775330993814
0.7111828963031178	0.7405333382046901	0.3161716540110733
0.7485222093360911	0.7975465755890973	0.3100984271416885
0.7458502458826513	0.8456905599528527	0.3932968505893063
0.5530679689524679	0.3645731484278026	0.3599816354718081
0.3352662849792634	0.6357261974941552	0.4232234870448137
0.4257224441872690	0.6248666127639821	0.6182389026734354
0.3812717239191405	0.5929071791236733	0.6922588753891949
0.5066433052570238	0.3502961534961246	0.6298877576252476
0.3597114773677338	0.4904558400832028	0.6243103166870417
0.3819079477934444	0.4193370147770153	0.6398156785612054
0.4928792824002830	0.6464079359966052	0.6382901949490944
0.6174838066981303	0.5771893771545659	0.6432922405438277
0.6395490026925074	0.5064417556026845	0.6227996504829173
0.5592055732370735	0.6398085032354844	0.5627951707833936
0.5928362518337204	0.6976068665306714	0.5843848229113311
0.4722197798703526	0.4904352024320290	0.6289481368423513
0.5959649111816947	0.6689898732475137	0.7762250298784837
0.5636036423300546	0.6093677538405667	0.7537225984433731
0.6198693706855029	0.4059061402310749	0.6885758804712574
0.6763638469268810	0.3747253080177108	0.6633292224441509
0.5270817944232287	0.5064574951865750	0.6291016934996738
0.6758368800249811	0.4271221519877662	0.4851323596655469
0.6193244961356726	0.4593411123172583	0.5114466330653432
0.4338709686662989	0.3852332611251758	0.7464140106006920
0.4007385960210449	0.3254543232922178	0.7641753076461096
0.3212782450149799	0.5692846670304434	0.4929729548208159
0.4075564203910546	0.3002164930332298	0.5713363467770818
0.4419295790466207	0.3581424471066917	0.5544952171490026
0.3773918353309723	0.5359872675794183	0.5181537938088977
0.1420056465755678	0.7026894412697483	0.6146944781911861
0.2648744204780117	0.1503056937475636	0.6426256969971765
0.7311569695486867	0.3877527156793118	0.6412866610162500
0.7767399929413273	0.3651754048952757	0.6979748646975312
0.8380565978577652	0.3408067502550177	0.7027125854615611

0.8702069402442840	0.3241341609613249	0.6063184938741735
0.8399663215271110	0.3326447793650981	0.5054283600586557
0.7786928126936116	0.3574454081519894	0.5004979908239487
0.3577947975136633	0.2763625300885419	0.5983280979917819
0.3071860742146608	0.2509669851383954	0.5605064899988104
0.2628275716837729	0.1997119578001672	0.5611726664647547
0.3560396071521398	0.2049661435900918	0.7226080988478414
0.3120444639811226	0.1532425565420902	0.7226437943185371
0.2695507187546939	0.6157196237619557	0.6476634900494023
0.2395710002889492	0.6769746903701607	0.5202014172081090
0.1828350535352010	0.7116493389045286	0.5258033639568979
0.2156653466870942	0.6236394637593043	0.6930334930108318
0.1589752775328677	0.6584319793758360	0.6985837942579793
0.6411505607662119	0.7226779870902965	0.6155838253180586
0.6396833223023399	0.7903988548217037	0.7452222610115865
0.6827552340154497	0.8428174713655604	0.7515976842910246
0.6917455966793272	0.7503368822913105	0.5819007829874551
0.7350921852953650	0.8023871641283544	0.5887151234450104
0.7309931189899012	0.8491601708305183	0.6739762631332302
0.5738129753148880	0.3724610558900426	0.6112012655410142
0.3255017191381535	0.6256409872550276	0.6677224365540899
0.2817763268202625	0.6575664604092114	0.3494221270159447
0.6657348451512746	0.7235180327656092	0.4020647972926479
0.5079543219884252	0.4459082902212004	0.3700453065442839
0.4468424647964555	0.4952240884396242	0.3711889479262329
0.5570500376829876	0.5101238258405817	0.3718719080041916
0.4962214657937094	0.5592936882433956	0.3706457975389570
0.7146271439758235	0.3338478040835854	0.3601383298765641
0.3366471488997069	0.2821238331733803	0.3831143095390718
0.2635755626896633	0.6280879806477848	0.6069020454406788
0.6392454613532542	0.7366296183842399	0.6563597033348223
0.5164990695276658	0.4442393259552133	0.6281223279256963
0.4465941603447299	0.4811191385633806	0.6291414179832414
0.5527153886421093	0.5157406530176961	0.6295147592373442
0.4827528911388448	0.5525666185721836	0.6317657232671104
0.7389288658570772	0.3774342611422384	0.5998912031915602
0.3588168704921289	0.2612221824201226	0.6380444036789088
0.7033985351828982	0.3416858023598209	0.2568205480648212
0.3500482018591994	0.2880196411609147	0.4860172041167088
0.3068595437764102	0.6723170123573069	0.2559959847147032
0.6504677414603448	0.7158050957265687	0.5039166077735376
0.7198230383554356	0.3711174568805322	0.4997420432711137
0.3810235234045891	0.2611065590604798	0.7366059793894498
0.2806100572907986	0.6281286194045996	0.5050906447882956
0.6152042418459183	0.7338318855139817	0.7535087607356207

```
C-CENTERED TRIMER
   1.00000000000000
     36.5045130000000029    0.0000000000000000    0.0000000000000000
      0.0000000000000000   34.5667380000000009    0.0000000000000000
      0.0000000000000000    0.0000000000000000   27.9898899999999990
   C    H    N    O
   216   150    24    12
Direct
  0.5295704076151166  0.4184554431577372  0.3001215843294321
  0.4814468517491031  0.3781779768341953  0.3089887857302409
  0.4211102692466029  0.4693085748733233  0.3066136762555501
  0.4219994332818950  0.5340843330785863  0.2948587530825706
  0.3844979026831306  0.5217001963587365  0.2953761325419044
  0.3839445619769131  0.4824392412482332  0.3029750150346064
  0.5790558172800377  0.4687450099368901  0.2993860168852696
  0.5795297063405513  0.5337151831529945  0.3096807898457702
  0.6167709036023242  0.5205655141701636  0.3085047654037579
  0.6164946028446752  0.4811854016417113  0.3019019623293146
  0.4714220210428379  0.5843054615503698  0.2968122321169506
  0.4823397227740816  0.6247657137724932  0.2997570081087623
  0.5189936115343524  0.6248215624826976  0.3082866245394407
  0.4701335786767770  0.4186298665579571  0.3083833341076182
  0.5183310620464487  0.3780455352168381  0.3024373430847936
  0.5305086615650031  0.5844005373434504  0.3083102197814411
  0.5673274727101454  0.5722578914353867  0.3133811768127164
  0.4346757495962870  0.5724217537930090  0.2912307432396088
  0.4332060328623367  0.4308312587017776  0.3113998011069307
  0.5665289470947756  0.4302805642945284  0.2962651780957232
  0.5958478318483117  0.6020534633708357  0.3234429804583983
  0.6037266392362026  0.6319180186537019  0.2909735950979673
  0.6293803318065886  0.6604328200554278  0.3020270483190238
  0.6479604430514740  0.6600278182907112  0.3459859576722790
  0.6404435760382939  0.6300530965577854  0.3782887505029772
  0.6150858417369254  0.6013958895507796  0.3670088083905305
  0.5953321320182590  0.4001278264335509  0.2893512236750766
  0.6035282115117094  0.3728594213459581  0.3249268122854999
  0.6306894628161600  0.3450159807679224  0.3178817496521450
  0.6500619503005715  0.3433547901049461  0.2746644813866043
  0.6417096221040999  0.3703992760900708  0.2388839178518247
  0.6151015020056601  0.3985780936628178  0.2462632750807035
  0.4044134902042026  0.4010376164805068  0.3201731245478313
  0.3844485095291177  0.4013755435697139  0.3631735761995307
  0.3589942497993398  0.3725598898498083  0.3734205627567631
  0.3520916207271304  0.3428089678726734  0.3405689675196156
  0.3712825695375934  0.3428434642041335  0.2970578576252925
  0.3970859292833222  0.3714744875818224  0.2870921504381035
  0.4059542277708337  0.6022714362483776  0.2814869414456724
  0.3909477335681980  0.6053815132928949  0.2354130436457786
  0.3633543217945821  0.6320133537709136  0.2257361332191196
  0.3491508677576574  0.6555736554516988  0.2621173929864390
  0.3642948741765011  0.6529141179214110  0.3080470343810026
  0.3926630731517272  0.6268585273976104  0.3173841250571403
  0.1960229812177170  0.7556928662322683  0.2785563009613232
  0.6776656451266806  0.3126170533528705  0.2623433076793847
  0.7184666484806080  0.2627688375314429  0.2994624439093282
  0.7313260529902680  0.2491195950122422  0.3437709795930468
  0.7545564445413794  0.2172203228532135  0.3459095529528002
  0.7653905223089732  0.1983153312000197  0.3040729290983350
  0.7526402114756767  0.2119138049780790  0.2601522829916572
  0.7293842509110839  0.2438998744504102  0.2572584191639223
  0.3260464539114637  0.3112385575810335  0.3545739969941036
  0.2838072937335654  0.2625191427141206  0.3160769362524114
  0.2628300140857559  0.2570775536058493  0.2744910330652012
  0.2392359938393868  0.2255801785126958  0.2705972633122471
  0.2808690160957351  0.2359351029516345  0.3537619354180682
  0.2571059313870435  0.2045087033883377  0.3493363496530520
  0.2361971139106238  0.1990815636582017  0.3081036949505022
  0.3187299725070739  0.6826734971862950  0.2482529080224627
  0.2622525971013176  0.7142635609053706  0.2806629243303320
  0.2530597225748817  0.7364757994747685  0.2403076829063163
  0.2200673514758664  0.7569058023886441  0.2397890872530335
  0.2382242201460801  0.7132484506068885  0.3199533192077949
  0.2054000687624896  0.7337459979240389  0.3186903519308003
  0.6741650559966346  0.6912067874330473  0.3608029925239706
  0.7171034533686799  0.7396792459786266  0.3229753885299355
  0.7200514343087662  0.7662255860153656  0.3606931209470363
  0.7440330714946821  0.7974832209046772  0.3564343293116989
```

0.7383202360915281	0.7449520985959676	0.2815616304310178
0.7621281131682356	0.7762798878645635	0.2778399606926802
0.7651531939318410	0.8027720646143853	0.3153582014099524
0.5390143541704774	0.4217337726490912	0.4964789906947925
0.4957156186445744	0.3756151200152583	0.5042798802860984
0.4256150573534878	0.4585145359954989	0.5054736877417378
0.4184671445267258	0.5230229192781277	0.4949824349766835
0.3827907976106222	0.5056692577333692	0.4963937081023873
0.3871266204656847	0.4665694602262580	0.5030853471578733
0.5822471458002874	0.4780189072855247	0.4962865253326506
0.5749483845037525	0.5424783036974471	0.5072143434873517
0.6134667158317892	0.5344534739407815	0.5051889072849073
0.6178968340848543	0.4953892794590727	0.4982556391722215
0.4616481257172954	0.5793385235910280	0.4963394111527721
0.4683122403793971	0.6208966184008610	0.4978925371754634
0.5047813124280794	0.6254062715811521	0.5058438846555825
0.4800665179141795	0.4144028598567857	0.5053395374319404
0.5323005163390429	0.3801635824658620	0.4973666448151566
0.5204660071474403	0.5866127017246432	0.5067318599599463
0.5584097183391155	0.5791742260002046	0.5111689829893932
0.4264278129670509	0.5628122864961355	0.4914548627442166
0.4420927925911462	0.4217912666698940	0.5091268614707429
0.5743097988595903	0.4382545565830389	0.4924762326953634
0.5838246671271635	0.6121709842636278	0.5202207586652112
0.5886772510281963	0.6421912863850893	0.4870196210024920
0.6128850038432091	0.6725453733497556	0.4961464872299453
0.6324123963234796	0.6740257528833757	0.5392725412841874
0.6274668499252726	0.6442806852521481	0.5727299020932983
0.6039282862413793	0.6136017712783320	0.5630873493079642
0.6063391090094552	0.4123482098424768	0.4839837083429269
0.6175189488595367	0.3846172313736568	0.5176268881042672
0.6478835505202860	0.3611008405098820	0.5090830772084671
0.6675035090058715	0.3642158619816754	0.4661601819511245
0.6561600725155599	0.3915420471031681	0.4322121819356615
0.6263701040636515	0.4155539785872657	0.4412325552153596
0.4165997759973269	0.3886799670833385	0.5171072396538574
0.3964283008243256	0.3864701032766339	0.5598433660520257
0.3728337612893159	0.3556514278422427	0.5684817645108247
0.3678654266668736	0.3266122896024162	0.5341160347870645
0.3874579883955408	0.3288716748500283	0.4910888034584738
0.4117574532611092	0.3593425094211586	0.4829779436356020
0.3943525551453339	0.5885046634880363	0.4821650149921868
0.3749971029858286	0.5849023107951263	0.4389043727520451
0.3448000140379291	0.6081563087619611	0.4292177970397656
0.3323948532093455	0.6351234058472557	0.4629544508353970
0.3513633340903596	0.6387238097548127	0.5063133244285022
0.3821379012324795	0.6159515855194350	0.5155367153613308
0.1818169330210311	0.7379170719774250	0.4810115760035560
0.6991421313316448	0.3385486877830103	0.4533273555842094
0.7519201112069079	0.3028757040750802	0.4889504830036621
0.7610527329457999	0.2825348748254318	0.5307315214451649
0.7915226968758872	0.2582527118282226	0.5315474698999096
0.8131867470669901	0.2538073571014814	0.4907515027749307
0.8041022582821644	0.2741084425674252	0.4492593275328352
0.7737552116061502	0.2986484967485382	0.4480026670560626
0.3427307272830645	0.2937626745741401	0.5462662357580048
0.2979209171895122	0.2479271411085871	0.5108242711142474
0.2667430220745499	0.2499278988289419	0.4816137507003730
0.2411399226786598	0.2199938266619717	0.4820376122277155
0.3032653032333311	0.2156404286360234	0.5403657521120293
0.2774648355944005	0.1858579561606457	0.5404346235426321
0.2463257949774070	0.1878241384849901	0.5114920566677295
0.3001604785171031	0.6597941541164983	0.4498374843124114
0.2455830734661611	0.6926895520090478	0.4837637931321692
0.2439077521949297	0.7253094734446811	0.4540293076464046
0.2120844921568734	0.7476508248395141	0.4529037230056424
0.2152748190116621	0.6829041934510155	0.5121324195369429
0.1836189630429765	0.7054674229718069	0.5106844935074873
0.6572834731533566	0.7068515381787763	0.5523980494023187
0.7011418406978742	0.7543094534849586	0.5181406458140029
0.6948995722802126	0.7860115696395873	0.5483405996285013
0.7199137947916451	0.8165247822187535	0.5491635618023426
0.7324266691475998	0.7536137458512812	0.4890257237568452
0.7572464140397549	0.7842694278798746	0.4902004641469311
0.7511610463613039	0.8158617249872053	0.5203218355690021
0.5519817305817604	0.4290988704489133	0.6896303523295825
0.5158186980048588	0.3764298382667934	0.6961236461794794

0.4342218603094109	0.4463127990227106	0.6986293850343125
0.4166542859547778	0.5084800931007822	0.6874866748688987
0.3842968362335238	0.4849914554050966	0.6896669971977839
0.3949505144177531	0.4473207725394311	0.6969213528885192
0.5860817374420619	0.4918235435313214	0.6912145967093184
0.5683421947692009	0.5537861641652346	0.7032846623355714
0.6076385570262135	0.5528064643761654	0.7025138242155685
0.6184063455345074	0.5152539583444744	0.6945993438697811
0.4508645948148964	0.5712284621486001	0.6887500749171396
0.4517040268784490	0.6134257853581476	0.6885146188674997
0.4869967356083494	0.6237786466421309	0.6971760342754688
0.4948528291755151	0.4122411421633697	0.6986229873900492
0.5513151993212140	0.3869199038791974	0.6891941818273764
0.5077822588568923	0.5879151617797942	0.7005381378471812
0.5461716787072672	0.5869913152345803	0.7065613338129010
0.4184558515902780	0.5490867983846554	0.6837579126469192
0.4563100869048141	0.4131099493431440	0.7026139169073347
0.5843652310321986	0.4513560703748608	0.6861299296477114
0.5661227293226163	0.6239618976968119	0.7162907672666330
0.5730394795531829	0.6511843643020211	0.6802555804740691
0.5937098437822231	0.6843390171258148	0.6895993402318976
0.6071736617885944	0.6914332659536599	0.7356897632107889
0.5991737847368809	0.6649106360697287	0.7721752211122233
0.5794296088470926	0.6313995574770486	0.7624914039670437
0.6196323008043124	0.4312469651757710	0.6756959990180937
0.6361382103375368	0.4057024645213749	0.7082398154484046
0.6687688761667641	0.3868111435478896	0.6968145166707144
0.6855086148063024	0.3925339232472992	0.6521975446204971
0.6690243551622139	0.4178822691995998	0.6195447888638822
0.6368975520998357	0.4373126355467474	0.6314080080458038
0.4363591481888245	0.3759808945609162	0.7114878268141304
0.4232440053755128	0.3677837653415545	0.7575851261723038
0.4040705559228767	0.3338082047906586	0.7667610164119641
0.3964200998426841	0.3075678944169617	0.7298327680689518
0.4095473116002937	0.3155146750237682	0.6837887078796524
0.4297082704780189	0.3491260539745480	0.6749613341139206
0.3832934236714471	0.5696963386540915	0.6744155072344376
0.3642505005131115	0.5635963928653209	0.6313383088060067
0.3324808871586359	0.5841831963235968	0.6207681633257273
0.3182088060197597	0.6110457736547231	0.6533752665536076
0.3366102037863283	0.6166059556655740	0.6969355952025348
0.3686483865029521	0.5962997616847245	0.7072277080908730
0.1698863071774962	0.7160170267005138	0.6868933249842087
0.7198086009755502	0.3720564265202347	0.6370662009657261
0.7800482375898191	0.3500966809301215	0.6724435016229817
0.7976925446783685	0.3454394443621637	0.7167331374430990
0.8325897678182310	0.3293081736741871	0.7190685669371301
0.8504056168034316	0.3175681124893913	0.6772996633272235
0.8328576503124919	0.3222891877361010	0.6333689045330145
0.7979008481012960	0.3384958022769249	0.6304204669923837
0.3752019117499045	0.2719474619642396	0.7433653325903342
0.3286565549100415	0.2253716160987589	0.7119921460995110
0.3028994510827853	0.2208875000557417	0.6750106363445574
0.2771154467849486	0.1912316259094579	0.6769887477805187
0.3284108018454212	0.1994672919245549	0.7507340618526921
0.3023769668571859	0.1698955007019943	0.7520117643456925
0.2765977311358839	0.1655367070346743	0.7155574057456353
0.2850122427170719	0.6339540643798228	0.6393933953468460
0.2318584516278822	0.6684728999678060	0.6782832623001551
0.2225712170861164	0.6939688108740242	0.6412077536225544
0.1916885969815706	0.7174652322155172	0.6459422417349205
0.2099630420046423	0.6669919572233218	0.7195626780548757
0.1792402770873166	0.6906203995834032	0.7237865647551285
0.6292025009142642	0.7263816592297558	0.7496996953444857
0.6754842694499785	0.7732346440845139	0.7183818768414975
0.6760540364573596	0.7987593545352926	0.7575039960185074
0.7020923131909143	0.8283160469298154	0.7588800779150543
0.7009689951180393	0.7780770881989256	0.6811582904633726
0.7267880016039778	0.8076970600034170	0.6832291738497195
0.7275972274326684	0.8330133174550184	0.7221613140649772
0.5362674358298667	0.3530711189873664	0.2998898119178968
0.3543506312525768	0.6712573714581351	0.3370928105360313
0.4642245349686979	0.6495685177768687	0.2965767720613385
0.4043022739133087	0.6251939497818201	0.3532478282770800
0.4636703454499128	0.3532448897404705	0.3134086005486583
0.3611734931735983	0.5407983555181697	0.2906735527307181
0.3600495954217627	0.4638420190884905	0.3052073458933648

0.5364886962189025	0.6498259523777110	0.3138222455447257
0.6405707227104483	0.5391907933293586	0.3117214685085770
0.6399783338692576	0.4620601660462494	0.2992400706214047
0.5892625794843992	0.6329190647043949	0.2568621486075786
0.6337484168343397	0.6839352669069790	0.2765234973325901
0.4709936367103901	0.5009016464677963	0.3024618200213409
0.6544535012671641	0.6298844197869038	0.4127159854715993
0.6092947151214111	0.5784628971381225	0.3927659100480778
0.5885486692961853	0.3736220912198134	0.3586642035139268
0.6361306229824474	0.3242405314214925	0.3464108556023940
0.5298416995573944	0.5019783877955524	0.3033788829054609
0.6562995286303955	0.3683353112963184	0.2049521628771699
0.6089348304355262	0.4194311768449471	0.2180715759712267
0.3897779260356469	0.4241424039835118	0.3893269492017354
0.3444926981383516	0.3724039887270185	0.4074993567425449
0.3520322920151724	0.6350204658299901	0.1898482144079021
0.3673233506030779	0.3195377671267668	0.2711722584959549
0.4121613378669135	0.3707369158535018	0.2534262381468206
0.4012335009381899	0.5866566043714952	0.2069901531846535
0.1702329268794490	0.7716574529245152	0.2775538334571460
0.2177265373760462	0.1743803847989828	0.3051154756321127
0.6905685963154741	0.3065695019836060	0.3334792509201933
0.7230314973098504	0.2639519639861146	0.3764909638075634
0.7642189744106562	0.2071581471036314	0.3806765856602272
0.7835720655154743	0.1732781429453887	0.3057439287205516
0.7608860362255363	0.1974775365178784	0.2271466437765682
0.7195895469898699	0.2545098344400744	0.2230194969938959
0.3109345470197346	0.3081059211918239	0.2849491312921065
0.2650555795415225	0.2777921411494455	0.2450862355297257
0.2231406212260025	0.2217889474341544	0.2380643626453038
0.2969412632006189	0.2400099239357023	0.3860565947805607
0.2550902171992123	0.1840091222864011	0.3788951376886314
0.2986382688788228	0.6795002836414805	0.3167221436509541
0.2715776221920146	0.7371677735075657	0.2099151185207924
0.2131772277620329	0.7740466078966360	0.2082621685055353
0.2455698669366830	0.6962662849859057	0.3514921534336775
0.1870241802589212	0.7323855672870606	0.3494269061736262
0.6897318957087146	0.6945214787942862	0.2914169657764569
0.7037407447771378	0.7623132106397905	0.3928190993363063
0.7460106141830670	0.8179899434161070	0.3859905340125757
0.7360772741887526	0.7242539070360910	0.2521433193375380
0.7783896973393064	0.7799576104277963	0.2454321295879220
0.7837696614679647	0.8273582396442301	0.3125079170579873
0.5527750604836460	0.3575725558770818	0.4936813596544121
0.3423787110934513	0.6596509084466858	0.5330938308552486
0.4478501640074790	0.6434795492263000	0.4939835718396167
0.3966445292853242	0.6190958142432443	0.5494152820878839
0.4807427134405998	0.3486236535183658	0.5078095771770684
0.3572170358963063	0.5214213478243070	0.4929440688844849
0.3657094695181350	0.4449136238915237	0.5058209143177199
0.5196400227279199	0.6523887325488531	0.5102112119079683
0.6348555196810651	0.5561042158423187	0.5083120698224000
0.6435174961059120	0.4796910381119152	0.4950090687504311
0.5735232922168099	0.6415161632516583	0.4534149896030019
0.6161620132904252	0.6953788655101329	0.4695188404761200
0.4711392501233650	0.4969242290052265	0.5008730565898404
0.6422225675529512	0.6457620790859071	0.6065807941237875
0.6004663848109799	0.5905409857364409	0.5893813152411169
0.6024337567844317	0.3817614310417022	0.5511049142107921
0.6558616417241856	0.3398852167661309	0.5360519581700176
0.5295110280446549	0.5040073230588648	0.5016925339405506
0.6709779804553265	0.3934363873606906	0.3984467213312511
0.6179580697205580	0.4368226898645398	0.4145429441540148
0.3998864520968716	0.4090087334452929	0.5868183410642617
0.3580057329547128	0.3535098920919540	0.6022280895817046
0.3303969846232003	0.6058170459415010	0.3951890546139711
0.3841793388611104	0.3065636619293398	0.4637881887691023
0.4270086663232764	0.3606374652048849	0.4494708464789915
0.3841802436403610	0.5637866656593319	0.4124547385874085
0.1569962938504030	0.7554988361308141	0.4797948945888427
0.2262513778529120	0.1644184301413670	0.5118714734265154
0.7151909597214340	0.3387898317314035	0.5237240073324859
0.7441185160670685	0.2860224674718881	0.5625928699484746
0.7982314998120146	0.2426546764736290	0.5643136079288580
0.8369629930618018	0.2346594564652697	0.4913035917063723
0.8208313361803004	0.2709807662537934	0.4171495609900528
0.7668451544282349	0.3143862899402295	0.4156007460123394

0.3249267498649468	0.2927689663689949	0.4764351669665691
0.2628224041129889	0.2750444360332752	0.4585134746606491
0.2169534081051447	0.2219893948835157	0.4592206205286997
0.3274602782353261	0.2139271924988048	0.5629895946015324
0.2819592966681506	0.1607789494649832	0.5634096794681105
0.2814368368521584	0.6559015489423473	0.5191266528507446
0.2673853705358309	0.7330437444331539	0.4320419172123838
0.2111017397625872	0.7730285319511102	0.4297142661929237
0.2167609345376189	0.6575525171198106	0.5353253278209532
0.1601785444715005	0.6974026367094539	0.5327782226361115
0.6752525852145570	0.7092476699454886	0.4826849855418049
0.6706294883046624	0.7866923913332103	0.5709099495270965
0.7147384399956990	0.8411177773595249	0.5726874734283832
0.7370794118168111	0.7289168397848939	0.4654697923647854
0.7815501847052699	0.7832760745005587	0.4674953624396865
0.7706204624108735	0.8398237204576218	0.5213031983167965
0.5747807555406885	0.3679537140656502	0.6846410915908625
0.3268093138990698	0.6380373690153742	0.7226517625137058
0.4285341772339955	0.6325460789547034	0.6826668574788191
0.3828141558945458	0.6013423806723042	0.7410001030246901
0.5046716362776842	0.3473558200668078	0.6985732136534685
0.3565107880393100	0.4959231083827467	0.6868124493669544
0.3774411682443027	0.4221279340979578	0.7007143558642498
0.4981481349448282	0.6528097167933422	0.7002226320121313
0.6250829464039942	0.5779575038525612	0.7071530562628301
0.6462222541325071	0.5043586781328129	0.6919659677006170
0.5625908362935853	0.6460667356428293	0.6442666207135892
0.5990005003916307	0.7046501929433310	0.6604867502044180
0.4726939608857396	0.4927773121026276	0.6933804831971588
0.6089887359212823	0.6712306068377067	0.8081577018277121
0.5740448830555436	0.6105810163036817	0.7909918064975396
0.6231482612572319	0.4005454896892750	0.7427871491980500
0.6804226246776877	0.3664446256928193	0.7225518241072691
0.5299539510618456	0.5074395821921555	0.6959402149690090
0.6816865763894587	0.4219667442656473	0.5846047052913387
0.6243838918954719	0.4570445049901886	0.6056659884412250
0.4284118262334489	0.3883383319296961	0.7864437260313331
0.3945012424420618	0.3268934811899580	0.8026960304910518
0.3185512922351799	0.5800984283467454	0.5866455896943260
0.4045420274960285	0.2954145822939944	0.6543590622499471
0.4400720978997844	0.3548147045774082	0.6390654098842801
0.3751927790562201	0.5430598316627346	0.6053393213161666
0.1458201291116044	0.7345486081018177	0.6901190909462428
0.2562970387102601	0.1423815084124701	0.7171486977244661
0.7374785962048057	0.3785116295388334	0.7051628355355450
0.7838203809482951	0.3544838554466853	0.7495323379184886
0.8458667089931047	0.3258517031579765	0.7537440536398246
0.8777411982310196	0.3048757284238679	0.6790553494072809
0.8464506922969769	0.3133137870141961	0.6004338922175511
0.7844582294596875	0.3421940947850511	0.5960993032190963
0.3523611078736679	0.2709690129028198	0.6762269428947940
0.3032981626479095	0.2408817907251879	0.6447585696018961
0.2571845072333947	0.1884032030208510	0.6481082891956207
0.3481883007254039	0.2030344548912102	0.7794170143477501
0.3023872107696468	0.1499690126031635	0.7822931310845892
0.2696524028688614	0.6320976422831838	0.7089898128485973
0.2393654575351697	0.6952550168881461	0.6091799615162015
0.1847960867298318	0.7372332668654654	0.6168756719467087
0.2171596234392465	0.6471224173123906	0.7484914442822020
0.1625376961461839	0.6891181082439697	0.7560811170931925
0.6509327987722671	0.7284653358787763	0.6819315662368609
0.6565001478620600	0.7949322838343335	0.7863961400890310
0.7023237918044046	0.8479530236075210	0.7894512545440796
0.7002619097474505	0.7584391039478704	0.6505612778418448
0.7464484478241242	0.8108448529640084	0.6540845590735931
0.7479173387549367	0.8561383727864516	0.7238295317265736
0.4998351516881601	0.4425767917762679	0.3034291680979284
0.4429993171724064	0.5014481129607172	0.3015796263318686
0.5578444548212672	0.5014832320012086	0.3039162203302143
0.5010964684766526	0.5603045717231857	0.3018546775400451
0.6945797061522953	0.2948089952768666	0.3003627984036228
0.3078869076049695	0.2944176421469415	0.3168919910229098
0.2947153888803704	0.6923474382771059	0.2842672160087586
0.6927456662051742	0.7080321993487829	0.3234713273892919
0.5068865418634253	0.4419504621859980	0.5009993418417221
0.4433279404676123	0.4934361307996131	0.5005563330557473
0.5573147331047396	0.5075674228438133	0.5017278097611719

0.4937235014747487	0.5590952832920626	0.5013926500782060
0.7211577390181462	0.3277513072179506	0.4906515847652442
0.3236284351829408	0.2785157756730672	0.5084496990278256
0.2775221283403176	0.6696341754218852	0.4871112218028437
0.6762039167488874	0.7230561722821346	0.5150177712072456
0.5173677301093443	0.4438527462854047	0.6949299517518150
0.4459809908651468	0.4838792066549238	0.6929209233729049
0.5566744444257899	0.5163022933974891	0.6967048856179000
0.4852997551725678	0.5563688495373669	0.6954927619759986
0.7449410734414994	0.3671477045185650	0.6730565862369706
0.3536381391675396	0.2563357639260431	0.7078989105971603
0.2632131963026781	0.6446740357227891	0.6772080139525438
0.6503521113264716	0.7424276360894253	0.7140971207220157
0.6836569846696210	0.3043565807151871	0.2199881595232139
0.3217159901935590	0.3014473398210614	0.3969186802875276
0.3157693458144659	0.6940978970145041	0.2065197037979074
0.6783201284983769	0.7006152440801916	0.4033129314907218
0.7042606180974413	0.3283653304837685	0.4111941449714187
0.3395921505648887	0.2821187270540749	0.5880889921486619
0.2947480583007073	0.6698690064381192	0.4077922889938866
0.6602831484626522	0.7177905646901597	0.5945346201709464
0.7246815325313661	0.3612906073539404	0.5951805378266146
0.3770019167850593	0.2589122012013607	0.7844884061581714
0.2782408554477163	0.6423175593184923	0.5971171516882777
0.6283504885971420	0.7385650941356832	0.7912666741140510

```
C-CENTERED TETRAMER
   1.00000000000000
    40.4062059999999974    0.0000000000000000    0.0000000000000000
     0.0000000000000000   39.7078599999999966    0.0000000000000000
     0.0000000000000000    0.0000000000000000   31.8059699999999985
   C    H    N    O
   288   200    32    16
Direct
  0.2253033050715281  0.6942685028914768  0.1959677967529256
  0.2424668301515440  0.6542205856293088  0.2644414680516443
  0.2112290801923776  0.6689650105901253  0.2624329623053278
  0.6403269964295215  0.6839350698658737  0.3037736298550020
  0.6800600592507866  0.7252085963230490  0.2705666554519807
  0.6795047273183866  0.7506061425574483  0.3012074177970707
  0.7009731020727740  0.7779732409529202  0.2974031759507498
  0.7021480987312434  0.7276989552864400  0.2365573557915985
  0.7234122334279901  0.7551700501946322  0.2331928825705233
  0.7229727727587346  0.7805001080258841  0.2636976636193359
  0.5371343038282158  0.4343917172385700  0.2470052600723598
  0.4982537088855230  0.3941121861254260  0.2547008345546636
  0.4346847705443838  0.4661592720542955  0.2560981447172128
  0.4283265524033107  0.5222194988704530  0.2460351029119310
  0.3960809035564318  0.5071309046146166  0.2467190338094585
  0.3999221997710849  0.4731628552395759  0.2533295138987200
  0.5760928212979438  0.4834233192125246  0.2472026957557954
  0.5694596626247157  0.5393445358246388  0.2582131197963409
  0.6042651346072586  0.5324181030862589  0.2564317495453592
  0.6082946702076202  0.4985328866598114  0.2493834969579860
  0.4672184103402099  0.5712124215285166  0.2480606499032176
  0.4729139689689953  0.6073675135223735  0.2507636830958098
  0.5058046130314490  0.6113902770755042  0.2582439554583726
  0.4839652938460103  0.4278226157888296  0.2558194626385106
  0.5312336629774658  0.3981959092151202  0.2479631235261272
  0.5202008372394024  0.5776888759498534  0.2581651415824506
  0.5544462092421570  0.5711666839394247  0.2623348386400068
  0.4355292427183495  0.5568134826782097  0.2430154283633588
  0.4496641481349089  0.4342648627303809  0.2596527486403866
  0.5689762265884692  0.4488413837989268  0.2432777287831298
  0.5771283949937694  0.5998986571576402  0.2714715520421484
  0.5823243478605248  0.6259999106196121  0.2424191255762648
  0.6031309091580959  0.6529414195473472  0.2520410859293502
  0.6190601928530612  0.6547037832250282  0.2912327167671874
  0.6139258913892569  0.6287119331815153  0.3203345141873225
  0.5936232355546872  0.6015390747880012  0.3103675586944773
  0.5980701111033383  0.4264363040587365  0.2358050173752663
  0.6092592442575145  0.4037475307584370  0.2664970133292195
  0.6372009644743971  0.3838426669711964  0.2595239162538366
  0.6543973929130660  0.3856879249648934  0.2212940100212549
  0.6428797846814746  0.4078790223258177  0.1902290818703560
  0.6154362994143602  0.4282212104444509  0.1975322990980188
  0.4268722629634299  0.4054189606911210  0.2678047081829517
  0.4098964673742976  0.4034330929917469  0.3063405456248786
  0.3892739761357247  0.3763095752801751  0.3154416879680323
  0.3842764698956230  0.3506853924376390  0.2858037545702422
  0.4007897473034742  0.3527441574822406  0.2470098991029789
  0.4218902322793464  0.3796368136089011  0.2382435934813051
  0.4065626009977277  0.5792041901605265  0.2343179282562224
  0.3926602998277610  0.5796663017322669  0.1938070946276347
  0.3649064275071037  0.5991811318725566  0.1851128379402717
  0.3495545944375124  0.6180462115608066  0.2169168967799420
  0.3635072558204051  0.6179763699133578  0.2572858227157721
  0.3919475524857881  0.5991053534501803  0.2656685607493537
  0.2023619267190133  0.6890574424731872  0.2281434995881673
  0.6834686190274225  0.3637753221848972  0.2101088782062135
  0.7282284143048514  0.3288519799085945  0.2427101902985676
  0.7421251713839491  0.3198046848711720  0.2816740345592053
  0.7681892213442642  0.2969082228461290  0.2835730510715689
  0.7808713503297827  0.2825783538845925  0.2467916014270952
  0.7670411003863320  0.2915656106254558  0.2081630917034141
  0.7409360153285264  0.3145227168577175  0.2056126909834239
  0.3622564619071150  0.3217128146596305  0.2972194304250468
  0.3228900155662956  0.2812471104485712  0.2617765645987234
  0.3015150372060970  0.2795901091148224  0.2269705722742732
  0.2798963137698051  0.2525414540700092  0.2222715712863178
  0.3224366450121451  0.2553788195438912  0.2917996158799739
  0.3006608125294505  0.2284077923088280  0.2866330344068422
  0.2793144185823302  0.2267598223377472  0.2521674654067197
```

0.3194051064038675	0.6378834246484189	0.2044222381865235
0.2654210279589188	0.6592904804682242	0.2317992601970228
0.2567032575190809	0.6796377518508949	0.1973378583665720
0.2086025300790224	0.6696458563865471	0.3819533103544220
0.2188519761938920	0.6111092292482637	0.4286544194549974
0.1875746744906463	0.6259158047960415	0.4273442682111157
0.6077378148447362	0.7036042007217493	0.4661897252225704
0.6444711457931497	0.7479601340722694	0.4369027624314704
0.6331950814646472	0.7763719144201137	0.4587781191197788
0.6525029317575597	0.8054967596714685	0.4595306916256613
0.6750629093898213	0.7490250034571477	0.4160832797008016
0.6941914329744339	0.7782501598253114	0.4171859787723743
0.6830274444654301	0.8066352745697133	0.4389292985916372
0.5465644831443248	0.4402326331060284	0.4148598776443667
0.5154600452630804	0.3933820249722660	0.4217537979269388
0.4402115112153300	0.4524832820308376	0.4235474961186496
0.4239601127980835	0.5065618320861098	0.4142491090124201
0.3949154825858804	0.4858083815233750	0.4154238482574102
0.4047657953015337	0.4530381948549349	0.4214210321716006
0.5762474919564281	0.4956413772195565	0.4151764244741653
0.5598600181700066	0.5495243324451654	0.4256589019490694
0.5953287271469152	0.5490417709379699	0.4239731738472205
0.6052689599281631	0.5163904382042364	0.4172392698366847
0.4535837653500235	0.5620097202701159	0.4156285939343669
0.4530015912661102	0.5987089330083010	0.4167634357498584
0.4846740870393443	0.6087295658030366	0.4238079452491607
0.4954450627287921	0.4239094002041141	0.4231032594996346
0.5471816205631409	0.4035175106820261	0.4153647761845420
0.5046351846869540	0.5781481490729714	0.4249650743998870
0.5394590087412258	0.5781528690764592	0.4291939117824119
0.4248843003289618	0.5419560306943164	0.4112874944334548
0.4605783099492864	0.4237921295436209	0.4267310273712490
0.5753181853708903	0.4603125512873175	0.4113476536531633
0.5568455456229743	0.6106370479921245	0.4375094258083707
0.5574809682809324	0.6368543941017065	0.4079026034953213
0.5743956549571776	0.6667644401351851	0.4162005218606620
0.5904354492992462	0.6714937631561478	0.4548610117993656
0.5894768934977177	0.6455902837829911	0.4847935602551007
0.5733739550177758	0.6154291752935812	0.4759691057217361
0.6077513623559679	0.4434936258848509	0.4035520028893912
0.6222806559024809	0.4218356344015338	0.4331615019443655
0.6528931980714743	0.4068171185937494	0.4253701190921395
0.6693842135598391	0.4124757054825450	0.3872270873654234
0.6547297257795527	0.4336908107291249	0.3573009098716963
0.6246476768832911	0.4493831542792086	0.3655971846493635
0.4430857481170461	0.3911985875310783	0.4339632588047574
0.4256067571981590	0.3861215340060681	0.4716965605716089
0.4091298319039974	0.3559886672316838	0.4794347164928989
0.4087201632263288	0.3303746719262356	0.4491126224311736
0.4257885720059089	0.3353286032808650	0.4112138004300954
0.4430767577513815	0.3652058170269344	0.4040121791748146
0.3924521398103601	0.5588268672855415	0.4035600910814681
0.3756959670196204	0.5534164747054008	0.3653738050210086
0.3455952574342240	0.5691231695285637	0.3572390881670149
0.3308194153040688	0.5898635516546746	0.3875800213845214
0.3471064651871396	0.5949969967097665	0.4259610002900536
0.3777470816779750	0.5799899116609014	0.4335806337421641
0.1822509274441148	0.6552094979168902	0.4039656223226256
0.7013053331650927	0.3955937197397940	0.3756364020624453
0.7547329083336176	0.3757916285944873	0.4065557705641918
0.7679857455584450	0.3644606589895639	0.4448685810654419
0.7987897515912451	0.3487294856711607	0.4458799291881756
0.8167947576042820	0.3440123206719310	0.4088167325227412
0.8036944673359609	0.3555251549642406	0.3708190184282114
0.7729134975156461	0.3714573584363916	0.3693211440490263
0.3907727775424754	0.2983949589826417	0.4592962101018320
0.3543129740422865	0.2546904116332870	0.4282679214844404
0.3239893634591586	0.2540670723762022	0.4067992280627544
0.3046022594980413	0.2250039898324412	0.4071588947800397
0.3650858066350722	0.2259864532933486	0.4499793472202953
0.3455265059778922	0.1970256463660831	0.4499842475585280
0.3152578271449214	0.1963430822361533	0.4287496754504033
0.2990069862809026	0.6070973223149896	0.3765310737569076
0.2451842669139525	0.6255346774149776	0.4062364066566169
0.2399846577751230	0.6550071108679516	0.3828368631135194
0.1798860986095084	0.6037251531213604	0.5427677178559853
0.2042370759139056	0.5482587946395224	0.5866805828961352

0.1702665346253304	0.5543904396790836	0.5853366100057399
0.5717545213539762	0.7195495133939728	0.6261318265007293
0.5964313384178010	0.7715407519365843	0.5964037041353265
0.5802666063819900	0.7959062324386784	0.6208512440632012
0.5924445378243782	0.8287737817216198	0.6219535535217720
0.6245868048401748	0.7804571990245046	0.5731139325523994
0.6365941554203857	0.8133620546473358	0.5746546061682541
0.6206538638168101	0.8376850258134907	0.5991013990435543
0.5559274888773954	0.4485740989038651	0.5781906367348373
0.5329468016764991	0.3970483776288456	0.5846037964357029
0.4489500648497462	0.4424367707244055	0.5856181556275964
0.4239029111126996	0.4929700759747639	0.5766927536594362
0.3987715993492537	0.4674506727622647	0.5772611782724992
0.4139489790510425	0.4368314654833310	0.5830094070610369
0.5758666535724489	0.5083577506721746	0.5784462986853496
0.5507382520186545	0.5587506282122643	0.5883099141210502
0.5857711529880143	0.5643677613357173	0.5863976222192669
0.6009960262063837	0.5338345344344485	0.5801334613140557
0.4438112469376934	0.5527356505209718	0.5789039769806851
0.4370812635967777	0.5887620075063732	0.5809075136817787
0.4666587253626395	0.6040707106519249	0.5878815068059627
0.5081577359662901	0.4237110380140472	0.5856841323081886
0.5625969244208531	0.4124881008383558	0.5786995231104107
0.4914997798835832	0.5774310652288460	0.5881049926279973
0.5258719249626822	0.5835114183462935	0.5917670124279272
0.4188941048601862	0.5280345119989677	0.5740795040746508
0.4737648736732367	0.4176177820389790	0.5887429418227872
0.5809097306544926	0.4733649503160830	0.5747806023796813
0.5376637053316310	0.6186096774782293	0.5992525011818420
0.5324980913828170	0.6445093679450882	0.5699052123094442
0.5442190699780345	0.6770196642658307	0.5773861985274568
0.5606909328548927	0.6846522546390528	0.6150017074845469
0.5655834408452041	0.6589995695461636	0.6446443728762628
0.5547196196410848	0.6263854814083213	0.6365941871641163
0.6156884488545591	0.4624852829818932	0.5666346369825966
0.6338736068087772	0.4434205513274629	0.5957773540705789
0.6664590254492198	0.4338997634713908	0.5873999542091507
0.6815113146539906	0.4426345563645961	0.5491856278421651
0.6633281340627186	0.4613300330394178	0.5197728920379102
0.6311285541961018	0.4715067470072565	0.5286165953879169
0.4618772496505460	0.3824179282148860	0.5951180994173273
0.4448174124128729	0.3739812053578667	0.6322256041910600
0.4339112439960842	0.3412369792789626	0.6393119559462606
0.4387423197776106	0.3161402166977693	0.6089161080682418
0.4551486328573967	0.3244721281345380	0.5714880015876114
0.4669238203030945	0.3570982733321365	0.5649708765028506
0.3841280319109678	0.5388491798251991	0.5656326355983178
0.3693134790675854	0.5304793808424668	0.5269835421631752
0.3368959738251425	0.5398647683631901	0.5180571291597688
0.3178947176484569	0.5570322018397179	0.5480534816796545
0.3323587160296946	0.5652804535132474	0.5868045041823730
0.3651924171305756	0.5566635724248393	0.5952268224724900
0.1578987903183657	0.5821170477675888	0.5633195862937977
0.7156970100177531	0.4315577749396919	0.5371727707128210
0.7718732881691933	0.4213589275202503	0.5676597871834363
0.7868118034405438	0.4116548982497187	0.6056752884571712
0.8199469672616970	0.4019676283125531	0.6065239598186349
0.8386282772862728	0.4017301278725488	0.5695357499584616
0.8237419380112422	0.4113940068176451	0.5317940502139330
0.7906009185285580	0.4212706701431427	0.5304763758684738
0.4276247943905928	0.2810569075054802	0.6190812777314240
0.4019821781564573	0.2299923491514426	0.5881278090696684
0.3736158287340710	0.2220140717896749	0.5647125086049367
0.3609811433758529	0.1893370601663763	0.5654299468616834
0.4177437479193109	0.2049040309823663	0.6118546068699422
0.4049513092761245	0.1722592122832366	0.6121063115890063
0.3765093475265720	0.1643021841324932	0.5891764318369194
0.2831351819079559	0.5665517309917961	0.5366989464968650
0.2262196641371814	0.5699210977297688	0.5658331601846169
0.2139143863623497	0.5978141557903451	0.5437776921455115
0.1679717946522459	0.5502283702482003	0.7065827603066209
0.2027347639409653	0.5137178716235092	0.7667501580304072
0.1686543585028965	0.5178291031922659	0.7707106108447226
0.5317142195902289	0.7260593674114799	0.7958005047016452
0.5562262344580614	0.7798276024534657	0.7694468093974721
0.5464667476287338	0.8009342103761670	0.8027132006040940
0.5586458749620610	0.8338038993329563	0.8045908477401825

0.5778180342512370	0.7922548769193488	0.7384155204574901
0.5897609447756640	0.8250967396851103	0.7408902042962126
0.5803303296489186	0.8461142048795991	0.7740524153338018
0.5656484287292551	0.4592346690888812	0.7423338307690089
0.5527623619752883	0.4040891530090786	0.7477205425015482
0.4619335424114090	0.4316547610392645	0.7511446561320682
0.4272056723661244	0.4756977037587899	0.7413808179369740
0.4076866348490866	0.4455614558625305	0.7435774518505338
0.4287433657455408	0.4188780137904149	0.7499983039957396
0.5736923388568721	0.5217419047577627	0.7437116575146231
0.5388291284828920	0.5655522498833145	0.7541915446156511
0.5720142586645520	0.5784024298104916	0.7537200017241340
0.5931544241216897	0.5518535164619714	0.7468434831273100
0.4352168329362246	0.5382313305882837	0.7421166971485255
0.4219365993279653	0.5724008765957609	0.7423054705400389
0.4479981038204862	0.5932390480677370	0.7496567566080177
0.5235693401773903	0.4254965616843638	0.7504437484294023
0.5789092937264066	0.4250624919998798	0.7415537652709601
0.4771910655539909	0.5717924947447839	0.7521269622226068
0.5095418311947018	0.5845616382803024	0.7572125104787223
0.4155106534433490	0.5089833465058444	0.7379335986851958
0.4911724783243511	0.4126092817077992	0.7543109980977906
0.5854066397119738	0.4885380692766339	0.7393592821297991
0.5140454434176133	0.6213100371868271	0.7658763691888252
0.5114356326136956	0.6456054443329827	0.7340626666897708
0.5175135221251117	0.6795758066094461	0.7425040714682496
0.5254821142092989	0.6900849806592766	0.7833507396144967
0.5269837023723577	0.6659453150133665	0.8155200896016354
0.5218666655380082	0.6320082545702770	0.8067955534026495
0.6214511181405604	0.4845692782300063	0.7305124877052014
0.6431813169827877	0.4692643335877228	0.7592064778542225
0.6765629597379716	0.4649741744478763	0.7493680472157690
0.6890182741200354	0.4754948700735431	0.7102903858569256
0.6673964912760434	0.4907563565566573	0.6815678225407176
0.6342641818939885	0.4956387572444932	0.6917982771910742
0.4866826773755596	0.3756766542865325	0.7618774190849781
0.4801182507350988	0.3640472005824728	0.8027527897850293
0.4749234055229153	0.3299590669179954	0.8104303555121946
0.4751449132100986	0.3066085822512450	0.7773227953713523
0.4819653966124731	0.3180355081568914	0.7365220637985503
0.4880863790776185	0.3521484938131350	0.7290648630887945
0.3794400781112349	0.5130209602980056	0.7293707696223035
0.3663175942730113	0.5024377099478154	0.6905935861756942
0.3330210114460120	0.5071413679268353	0.6810196305079234
0.3115933920334339	0.5218507886145870	0.7104183925847946
0.3244009210491579	0.5319293323765346	0.7495077395110571
0.3578822271255289	0.5277220335023475	0.7587446674597470
0.1510432010204494	0.5361498752183557	0.7405938012105159
0.7243272806367018	0.4707496879285806	0.6971511927379240
0.7817104598291953	0.4737829725800766	0.7284027419547082
0.7980992739907551	0.4751936136294252	0.7673601458817674
0.8325278140517619	0.4751627084405832	0.7691171401918496
0.8511223558738709	0.4736222424321277	0.7321090416635772
0.8347880756458167	0.4722505608097217	0.6934697370296382
0.8002958134272656	0.4723755885010640	0.6911629133136389
0.4687449540888425	0.2704524639377497	0.7887672245050761
0.4434725876297900	0.2174103486045101	0.7613056193030786
0.4217908830861420	0.2055802655925832	0.7300208306661344
0.4097102786328612	0.1727545865163330	0.7317239779938857
0.4531239970439319	0.1957568398096887	0.7940783853881741
0.4408391098534373	0.1629091411910565	0.7951418087204317
0.4191068074185731	0.1511645124660979	0.7643248844423609
0.2761532132415371	0.5273694014088768	0.6984059044626212
0.2197151941928037	0.5279412943452407	0.7324988888040410
0.2021240051504530	0.5462994880112010	0.7021581103676292
0.4541357099127125	0.6268127916419234	0.2479069795384405
0.4025896429142702	0.5993352643821918	0.2972146394982182
0.4848916316072366	0.3705431389044504	0.2581294531624432
0.3730672836331547	0.5208720181921299	0.2427921450597537
0.3805425189058871	0.4543161596159012	0.2554474976975187
0.5190371359811967	0.6349037383580740	0.2629458989525023
0.6235443834715050	0.5512755363203767	0.2595924809659759
0.6314217561945092	0.4848765531685650	0.2461761936429848
0.5697583968912454	0.6251719721690383	0.2120575500653762
0.6059739806131015	0.6732992887767419	0.2291891797931711
0.4758603262009115	0.4994615381283146	0.2522477690482909
0.6259031540077540	0.6302427909599463	0.3510227988550281

0.5898487910906973	0.5814380858378921	0.3333148316511444
0.5961477555214043	0.4019934468311563	0.2964279948915587
0.6451907572888442	0.3665709685280861	0.2842427802242692
0.5284111662191293	0.5060199030273062	0.2524247712278359
0.6558621023691884	0.4085274369650002	0.1601475510207185
0.6069324600274725	0.4455806150380931	0.1731770840837356
0.4135176209630587	0.4232412461022858	0.3297088661350658
0.3768919870492479	0.3745390892616124	0.3458534493849045
0.3544181543448230	0.6001519462936595	0.1535201326983974
0.3981094457407663	0.3326773843201828	0.2237296991820306
0.4348024844944182	0.3806987168384700	0.2081253265580021
0.4039555480513063	0.5645539021350006	0.1689570987910291
0.1778225036468617	0.7004884883879863	0.2265267875302421
0.2623857771996033	0.2055440320799966	0.2485647847647491
0.6966971524036978	0.3618630544808150	0.2724478845613402
0.7323669548686597	0.3310807722703584	0.3104409561937648
0.7786114563198633	0.2902913490172911	0.3141584347123549
0.8012947392153583	0.2646252494007547	0.2482801549857328
0.7766872798767777	0.2805967421472416	0.1791490471546343
0.7302579662408188	0.3215238023152619	0.1754840585243934
0.3460435167119682	0.3227878431148124	0.2369656533726489
0.3019014669857393	0.2997262453464821	0.2035036756134520
0.2634437927526481	0.2516386273131507	0.1950792808424951
0.3389666338990580	0.2564335455462466	0.3187342002728977
0.3006279395572896	0.2083341806335542	0.3100939924130391
0.3005149090155768	0.6319067119187750	0.2640393401535281
0.2743304667557789	0.6834094783336760	0.1719649143660759
0.2187912434506572	0.7099834281916244	0.1690201131153301
0.2493647739743239	0.6386072448376074	0.2913524328738039
0.1936769333481994	0.6644875965326139	0.2879307511155022
0.6582930870462143	0.6834265998381950	0.2443039369009186
0.6624867829948697	0.7488347282145567	0.3275811426020369
0.7002096809638355	0.7976674570911296	0.3213385765536990
0.7026174132897195	0.7078912636065012	0.2126576638365419
0.7403977979603118	0.7567423206453069	0.2065743671499082
0.7395910797777904	0.8020635220394100	0.2611076213130038
0.5498534645355923	0.3786727883151124	0.2444275592669359
0.3524709573499256	0.6327787762630417	0.2826293043748335
0.4313020066655908	0.6145630945926984	0.4130943326230184
0.3902900038620503	0.5843797128658685	0.4634649477939133
0.5064496052828833	0.3677287655575396	0.4247486655796755
0.3697735648108277	0.4950873318607524	0.4123282634436392
0.3890322320746457	0.4309810516844630	0.4239469307603208
0.4937205339359929	0.6343305059377187	0.4273171952974259
0.6109784859320839	0.5710988379566739	0.4272649427707678
0.6304380458466609	0.5071738032347763	0.4141666170091454
0.5448885366635479	0.6335579560933945	0.3778243105267912
0.5747208065106706	0.6866616899165709	0.3924949020695724
0.4748024520942214	0.4927940106932062	0.4194977645744044
0.6014641735212175	0.6495004617667127	0.5151449739688062
0.5730307378184943	0.5953159277361538	0.4993876706794989
0.6095870443881698	0.4170453242239842	0.4628487810477699
0.6636548418182079	0.3902720607600162	0.4491550952523911
0.5253571280500895	0.5092547275922771	0.4200865484632646
0.6672509921425769	0.4374953892607399	0.3272813815301582
0.6136480169959495	0.4660620306628497	0.3421243332099840
0.4254605374471481	0.4060039923094454	0.4954248992702519
0.3963545916037020	0.3518903297341534	0.5092249583664259
0.3331854488600531	0.5656861412203054	0.3270700705463214
0.4259182176820539	0.3156419416046134	0.3872374759046794
0.4563965418698745	0.3686791261150694	0.3744765720070179
0.3868375176324065	0.5371126572830011	0.3416049918926789
0.1577469074073234	0.6667620820384578	0.4029961525574662
0.3000919092892096	0.1735971999358827	0.4289923438548934
0.7170378075110958	0.4014860511420750	0.4365735563095085
0.7540302736625355	0.3684036882313474	0.4738469030356060
0.8087029933008830	0.3401208813058395	0.4759460540775510
0.8408460917044456	0.3315121045045588	0.4095849945642752
0.8175121025177212	0.3521613516583489	0.3416099030068618
0.7629002941716164	0.3804215645279452	0.3396474002744481
0.3712935697747178	0.2983748234562958	0.3994832694686178
0.3157737758976113	0.2764985781983316	0.3899133423740500
0.2809405970556322	0.2249178196117982	0.3905761645162788
0.3886148119931672	0.2263965361350719	0.4666728182513213
0.3542282571894227	0.1747348170096105	0.4667606252963406
0.2811248468087114	0.5966022180038520	0.4357673051900804
0.2604061154848336	0.6663104219653430	0.3655352089459911

0.2048463851557788	0.6926943747529509	0.3638426565553481
0.2231273145164843	0.5883542499188140	0.4471513640080558
0.1672272244965083	0.6142887985695006	0.4446497632997977
0.6283819492600974	0.7044757953905166	0.4070314843197437
0.6094899249838787	0.7756126655083055	0.4750096381820542
0.6433814716228805	0.8275610715320217	0.4764080151420618
0.6836501781235098	0.7268360525224277	0.3990083592648500
0.7180457676722573	0.7786714904861224	0.4010603235962421
0.6979886603031853	0.8295132561124324	0.4397651012374661
0.5689490642405427	0.3877288727944778	0.4118037421612796
0.3360627986466058	0.6111331146844207	0.4499617732553249
0.4129872565966412	0.6006527368450637	0.5779471157900001
0.3761727793930189	0.5633453887715030	0.6254478634605722
0.5282598703857896	0.3702237345897198	0.5875037793402497
0.3724677001893503	0.4722177746589701	0.5738285408097892
0.4021574342069308	0.4123327098625862	0.5850146276241368
0.4712592732237801	0.6308041549077464	0.5920901503740822
0.5975529165156060	0.5888223879249492	0.5892301867161311
0.6273374286879383	0.5290847544176031	0.5771277425911044
0.5195261401196611	0.6389511892804292	0.5406218608539561
0.5401170458803844	0.6966352278041190	0.5538995243252818
0.4762621788447898	0.4882350247307118	0.5823588489340367
0.5778208533724724	0.6651179433971146	0.6742606838500806
0.5588706512225999	0.6065928753524000	0.6598561219683420
0.6224137790959320	0.4362596005751866	0.6255299473600615
0.6799541504986053	0.4191119211149174	0.6107424421445575
0.5234456229672390	0.5130409716013007	0.5833412634396967
0.6748188223163970	0.4675753937696195	0.4897253505272499
0.6173332004075337	0.4862365334906766	0.5055128953902516
0.4406821209482405	0.3933376484447620	0.6560610092371266
0.4216788951855198	0.3345694722550573	0.6687483079429508
0.3258560983957579	0.5339928920773911	0.4876173004833835
0.4592047938376864	0.3052923665867514	0.5474231222674216
0.4798717977188014	0.3631894442354144	0.5358366567319394
0.3837144921122954	0.5167273852373707	0.5035395688600411
0.1313049387962220	0.5868693041670420	0.5622005080405220
0.3665625511203289	0.1387480794047226	0.5897453083018717
0.7304953063018530	0.4402238058822645	0.5978583506839236
0.7722301550002650	0.4119060079942156	0.6345635201524193
0.8311693473475205	0.3945317454270691	0.6363448102711933
0.8645785621563492	0.3941110768230028	0.5701533024510518
0.8380355771755519	0.4113792332829158	0.5026619970686660
0.7791727879398890	0.4287560653352691	0.5009781624031578
0.4088675482733264	0.2762893285942264	0.5590945371675972
0.3617467988395903	0.2415415572333083	0.5458354453536032
0.3387281080950776	0.1835473909527437	0.5473445564646143
0.4398303711699646	0.2109665257606688	0.6299284307450345
0.4174992356045889	0.1528744508215696	0.6305057938756649
0.2682057529722376	0.5514593194698745	0.5956426441709846
0.2309020430982314	0.6147263679126184	0.5276640183857785
0.1705673171380615	0.6256355977002004	0.5257383600911743
0.2140031562278294	0.5266388088405463	0.6038793755209240
0.1534203687015891	0.5372044277168544	0.6014681612971911
0.5900526526317935	0.7256380531726533	0.5660982122431368
0.5583233607510477	0.7891086575221153	0.6387844960411615
0.5795953923548318	0.8475685362107992	0.6409642484436220
0.6368304600413254	0.7614804763150557	0.5537425420566023
0.6586745729532559	0.8199009901395263	0.5566369084441832
0.6300925164426409	0.8634293252390635	0.6002799032335591
0.5866736675941339	0.4006098110367939	0.5754798406658346
0.3180463760807910	0.5787893041013525	0.6105497722372831
0.3962112414319322	0.5794634859167913	0.7377711635762334
0.3675879143042861	0.5360663557918476	0.7890309511658543
0.5529552450742916	0.3767634948520506	0.7495448826818092
0.3808956680035208	0.4447953580963332	0.7412636822929779
0.4222283658428886	0.3925122229682550	0.7535700205719551
0.4477291892510416	0.6204981581607640	0.7525754369843592
0.5784615583702845	0.6047264478152641	0.7579364064127101
0.6199532622382765	0.5527122290901891	0.7446626921321070
0.5050013486758591	0.6377721365984785	0.7022048236293454
0.5156877691196828	0.6977944062291183	0.7168669245755503
0.4793025740504767	0.4822696651517628	0.7460582404438330
0.5323392977747400	0.6744985591023624	0.8474105803778313
0.5237337905269465	0.6133803282374137	0.8319520689060015
0.6336979923025055	0.4605037845629983	0.7894264433615129
0.6926089612693657	0.4522365411371562	0.7720342697374419
0.5215925253182545	0.5150051264378599	0.7475926400136650

0.6769554041837056	0.4985519663478225	0.6509957273846328
0.6176735329372897	0.5075487309453739	0.6691369549757507
0.4791886850935368	0.3820738930715520	0.8286676626613892
0.4704297450470686	0.3206759169270897	0.8422119167302181
0.3232343118652051	0.4996610305094820	0.6504367954743950
0.4827989347975215	0.3004693181404560	0.7100970126203240
0.4935904434333707	0.3606903100605480	0.6972125934493313
0.3827674405307511	0.4910145413963929	0.6673827440094223
0.1243467769639054	0.5394783778486724	0.7436899971008448
0.4095468186775953	0.1254726974635039	0.7656980264024441
0.7368433737896982	0.4805262329884285	0.7575992884101924
0.7836646629229470	0.4762936585215353	0.7964181667152819
0.8448660774659670	0.4762988480111024	0.7996189411383209
0.8781068709385520	0.4735749443538655	0.7334338730936386
0.8489714240029211	0.4710896226808213	0.6642864715025969
0.7877083332946558	0.4714396988148310	0.6609963059162902
0.4477055503546650	0.2628729622980837	0.7306399282572060
0.4145296881984762	0.2223543157209329	0.7043810995378003
0.3927033098791263	0.1641278487059887	0.7073221772206061
0.4697410104285502	0.2049398055810835	0.8183782040124864
0.4484743331663278	0.1463107654090746	0.8207501859908594
0.2643813703600086	0.5140069411438759	0.7580257907906727
0.2151219312181140	0.5572914778318141	0.6755604604367871
0.1545755090033331	0.5647536618629886	0.6829192219798136
0.2164428881562175	0.4993512476587723	0.7903746855652848
0.1558310661156099	0.5066390777901555	0.7974966142909879
0.5520128328465129	0.7349297671755469	0.7375281022223708
0.5299085208307477	0.7913251046581586	0.8268199127430784
0.5509657016016538	0.8499732806557856	0.8306035247610757
0.5851046185333998	0.7759026306369402	0.7123624395724032
0.6066920459094612	0.8341758559572156	0.7166522617392315
0.5897815438167174	0.8718222360646248	0.7760354642550420
0.6046730784908624	0.4180861563969356	0.7371863873765415
0.3084508437485275	0.5442580460830373	0.7726072132565651
0.2964912782676749	0.6433675610534793	0.2357265367957299
0.6586188685071276	0.6971966396705247	0.2712894494173793
0.5080629722015997	0.4518787983212880	0.2514774274836715
0.4507390792935524	0.4964918206734127	0.2516520188867638
0.5535367195890000	0.5090571639479076	0.2525398878843046
0.4962596600026353	0.5536928201847519	0.2524216704140553
0.7017257121215522	0.3519226923470561	0.2434818186564101
0.3447668148493278	0.3088670920989049	0.2637739249036166
0.2766945394341060	0.6100789205085347	0.4088509434107842
0.6254607985890520	0.7180661359416067	0.4341631317508998
0.5148735330467606	0.4520574708428684	0.4192414726567645
0.4505926853732163	0.4853083482293688	0.4191788678793841
0.5495553078617285	0.5167747108393687	0.4203320579987750
0.4852131172530224	0.5500846755024044	0.4202897942755517
0.7235147833687505	0.3918648767316241	0.4078198160719303
0.3736716485212531	0.2844097019993443	0.4264126567716444
0.2605679522023506	0.5632049897697184	0.5686495542816304
0.5846656634406562	0.7380107893961682	0.5935768039224792
0.5227043048652912	0.4547868147521528	0.5822039480072768
0.4536772870311320	0.4766296371840456	0.5817883435513467
0.5460454770704101	0.5246236540627912	0.5835219366775976
0.4770177963387769	0.5464469950476504	0.5831546871892295
0.7383625474719698	0.4316193280102090	0.5691735855353628
0.4143473807519909	0.2633378476183625	0.5861200293538694
0.2543301726826480	0.5236503456795301	0.7312634031498821
0.5454300850762294	0.7462071125081671	0.7651812714379893
0.5319648298209995	0.4588668488793362	0.7472767795093983
0.4596763049699565	0.4660149451300437	0.7460148900118723
0.5411797987783946	0.5312952103278776	0.7482879152570769
0.4688502062510244	0.5385036436077691	0.7476164027949713
0.7468285413653472	0.4746987390883440	0.7291333340631596
0.4544516111262642	0.2510286269241044	0.7578480539792293
0.6895694677569155	0.3572155991786140	0.1728297490274352
0.3600848380944953	0.3108166515077688	0.3337343363876281
0.3159249067526124	0.6476118986385597	0.1677079129623021
0.6411607984938422	0.6947030939708210	0.3404296593481251
0.7063264488415454	0.3861093501675682	0.3388708831490638
0.3912593453309073	0.2862484572814358	0.4953614897855573
0.2939438141597741	0.6175273171284714	0.3402318570678695
0.6061607866811960	0.7154330508432319	0.5023692209130312
0.7221123947045087	0.4231447289613480	0.5003302893228474
0.4304295371761227	0.2697099499720464	0.6553576723311354
0.2758155370206209	0.5760970671000243	0.5006122011948234

0.5691820292280300	0.7301407382665934	0.6627916651790305
0.7320329582838865	0.4644747922445742	0.6601306116423196
0.4755082001570801	0.2599355872497251	0.8243850256862892
0.2677018767639853	0.5344770436207210	0.6619168432702918
0.5253222007986071	0.7357794680303408	0.8318746619203781

```
C-CENTERED PENTAMER
   1.00000000000000
     40.3491769999999974    0.0000000000000000    0.0000000000000000
      0.0000000000000000   40.3383690000000001    0.0000000000000000
      0.0000000000000000    0.0000000000000000   36.8000439999999998
   H    C    N    O
   250   360    40    20
Direct
  0.4539248376436968  0.6248874837913813  0.2140059453132374
  0.4028143854661482  0.5975703339032445  0.2569802781065351
  0.4857492466781002  0.3727042398372994  0.2232222384424609
  0.3731849157937699  0.5203753436532376  0.2093036924408248
  0.3807606270290171  0.4548093790869724  0.2197099631546312
  0.5189140849252730  0.6332296314352432  0.2264646052381869
  0.6241494231111538  0.5511498208808095  0.2233067437041950
  0.6319924941954850  0.4857213091701653  0.2123076698712683
  0.5687946023135605  0.6245447449042258  0.1825358293512855
  0.6049862484616889  0.6719988926688053  0.1970304188550807
  0.4761561262329543  0.4993422711968439  0.2176505641385869
  0.6274601999772066  0.6286887696488438  0.3012706745962566
  0.5912910520423573  0.5806213201225613  0.2863254813631745
  0.5969734501365340  0.4046239168922443  0.2568416920194238
  0.6461468339101429  0.3697081925303152  0.2466056929383413
  0.5288367425177255  0.5066889658092192  0.2176131923470033
  0.6562836730750357  0.4096766251666681  0.1387087192110447
  0.6072981166779735  0.4462251966385214  0.1496636441393915
  0.4126231567228157  0.4242617251958924  0.2833746105962838
  0.3760162508594106  0.3760968337527602  0.2965294457723194
  0.3539568831927861  0.5986070626998499  0.1330773270576385
  0.3998892849261385  0.3347134477566998  0.1917287753706536
  0.4364904762546570  0.3822656783156195  0.1789994667466739
  0.4036269783232603  0.5635182662551179  0.1460649831425679
  0.1767832458915520  0.6957842318952956  0.1974789653703097
  0.2630740848052197  0.2094065831085921  0.2120826659708021
  0.6982021635384595  0.3653267920820314  0.2360815267443201
  0.7346243528852042  0.3358622619830700  0.2688663879359164
  0.7804100476193019  0.2951432098478876  0.2722008484467285
  0.8016365246740382  0.2681512426412967  0.2155115614149551
  0.7761503822458269  0.2828599321666102  0.1558706678525759
  0.7301905371706465  0.3237448229799734  0.1525859295274234
  0.3473711522281390  0.3245143710356018  0.2020709181720988
  0.3033315235449392  0.3017050419404025  0.1728140143727389
  0.2646859015516970  0.2544730910383363  0.1655144421098211
  0.3394050837765686  0.2596549343184045  0.2731570036435652
  0.3008766993614458  0.2124149047479414  0.2657007635504962
  0.3005311206796893  0.6296785779295521  0.2290465690732020
  0.2731662347318415  0.6796829121098107  0.1493882637350377
  0.2172393108071022  0.7052361211713878  0.1472409303481030
  0.2495564635304856  0.6359864638241696  0.2531749510150143
  0.1935066810303463  0.6608605732993765  0.2506274924738299
  0.6575647446876760  0.6820021305902806  0.2087282627694337
  0.6629279377659973  0.7461709564145976  0.2808520011000291
  0.7002310586727429  0.7945507546091267  0.2745720552161968
  0.7016096235974475  0.7061061211296491  0.1806859048415988
  0.7389347701609286  0.7545103636655272  0.1745374515222036
  0.7386088907020902  0.7991487565900711  0.2216030013873616
  0.5508311858821008  0.3810812718131867  0.2119599257771305
  0.3525825469527758  0.6305445534644440  0.2447347605139828
  0.4328741328400029  0.6129007893246242  0.3557891462561320
  0.3918955643336902  0.5832370491848208  0.3994271319218172
  0.5071226641335157  0.3695064306192075  0.3657498568762722
  0.3707683272123605  0.4954868273691950  0.3547458963903528
  0.3899219292531863  0.4322979732726252  0.3642381042206687
  0.4955632621237040  0.6319706192777419  0.3675240791408036
  0.6127001772907342  0.5691978587558338  0.3667998505879875
  0.6319967025998590  0.5061815894580421  0.3560206591504534
  0.5463207231373101  0.6313555002805922  0.3246006277190975
  0.5764640149408717  0.6833952184941317  0.3375260817859919
  0.4759595121760474  0.4929686112926053  0.3602462719279591
  0.6039899106439413  0.6458547961285092  0.4428709973312856
  0.5751631455786576  0.5927550766907383  0.4290237024150210
  0.6104255869653110  0.4175521735902388  0.3988608790372044
  0.6649844619258460  0.3917231993232755  0.3882028120268164
  0.5267775958961076  0.5085010896643994  0.3607094533073765
  0.6701378727944800  0.4382672605702411  0.2829947345483504
  0.6159053227747220  0.4657912947057904  0.2946020434645406
  0.4260440007649539  0.4078371922807228  0.4258220755835131
```

0.3964332331915772	0.3548061734115988	0.4377948138079514
0.3335217303023345	0.5650086075518117	0.2822325490382959
0.4262635015775879	0.3186255996279116	0.3326293871348831
0.4571319287523350	0.3706058607625605	0.3215613877362567
0.3873549442551340	0.5368626049321127	0.2940913846742530
0.1582682603409361	0.6643730490080588	0.3460220187545278
0.3000278770614254	0.1791198165150977	0.3683281714342009
0.7185210011614568	0.4025488208607249	0.3787378323375130
0.7539118651187906	0.3669069100337819	0.4107219453982918
0.8085254387084855	0.3388307316612181	0.4130207798488341
0.8426567676209308	0.3333085305878019	0.3566088836032741
0.8214045005814195	0.3568060242775314	0.2983323711692309
0.7668130373954911	0.3848108971903247	0.2961093006543048
0.3716541551492167	0.3017628396798992	0.3428501679414356
0.3157069127732025	0.2805042044564094	0.3348589186810834
0.2808089356681588	0.2297447534180278	0.3353316606258944
0.3887973014601678	0.2309421891414744	0.4007650983248761
0.3543583966236232	0.1800994374039432	0.4007355027099437
0.2817921548374285	0.5957193953353517	0.3765354295412072
0.2619756455503949	0.6652893088744838	0.3173665320206871
0.2062922352743788	0.6910748066792678	0.3148326108903907
0.2229842671308469	0.5863700165643201	0.3837595762613217
0.1669685178820518	0.6117349666837832	0.3805498266305997
0.6300887084051814	0.7010072934533462	0.3494530674929275
0.6107646524649443	0.7703379103594735	0.4086503706644804
0.6441457707988124	0.8218651597714278	0.4102518997348971
0.6856576025525276	0.7235087341037953	0.3427863104072454
0.7194582598124333	0.7749726930421491	0.3448377515791170
0.6987690693427371	0.8245879782608018	0.3785923268955752
0.5699138950449248	0.3887241210100573	0.3550043188790215
0.3375153454477346	0.6097134865879947	0.3884128380341388
0.4116546643877556	0.5988492475476347	0.4940830626100214
0.3762258191220270	0.5631565499244116	0.5375931021456416
0.5275449028563212	0.3723214548170249	0.5056296596032681
0.3711970385574864	0.4723207415959171	0.4943787224000823
0.4010795499201719	0.4135102696994781	0.5044406711950663
0.4698780832212918	0.6288643863684861	0.5058981639459800
0.5963503089540848	0.5876849721999922	0.5062373086639347
0.6263316785442183	0.5289617161989436	0.4959716744701269
0.5195375984716135	0.6369967176898068	0.4632280264055324
0.5404219932198995	0.6935851343638439	0.4751002178171879
0.4751857754519362	0.4882700797606783	0.5003166631762626
0.5755950027858103	0.6624419951787047	0.5801728606409225
0.5562003273913398	0.6050425514780585	0.5674184122463540
0.6213318553409685	0.4378458749455461	0.5386322615406858
0.6793041612879908	0.4215618550335456	0.5266810884375873
0.5223153354111827	0.5129182532704667	0.5011026778565646
0.6746693210782262	0.4685428839558435	0.4216678192870254
0.6168385356226674	0.4865033133845048	0.4345477489861237
0.4406810126229942	0.3951986810284663	0.5656925226125009
0.4215705301568956	0.3374672544607318	0.5772188163508658
0.3229960609794137	0.5330842891753033	0.4203674306551065
0.4576727190642255	0.3081660178293162	0.4718546883154632
0.4784234163587138	0.3650389156955490	0.4613117838843557
0.3808942928421226	0.5152021067317890	0.4331398756170385
0.1310516337472822	0.5941843896308774	0.4871341571455784
0.3616945037329026	0.1461963989268337	0.5071580180338472
0.7302560149119264	0.4436404137133255	0.5155389175303050
0.7727750506621214	0.4205126854607797	0.5488017133026692
0.8315907709504770	0.4029898100014284	0.5513511136558077
0.8643561165629450	0.3970233741291262	0.4939954192466662
0.8372554431792161	0.4092635686585107	0.4344783706224645
0.7784537098734502	0.4266518274665827	0.4320347121553846
0.4072582505531106	0.2806571985382413	0.4824201766672140
0.3601230206029292	0.2475639751258102	0.4694958405232646
0.3357000522606381	0.1910843115853257	0.4699583853138070
0.4359410123775631	0.2153722057003782	0.5441430528790034
0.4122462506692083	0.1587942321456899	0.5437637223715273
0.2669536911228699	0.5545510115788536	0.5143024767312934
0.2305900820268551	0.6142447544802674	0.4503605637114581
0.1706776303207319	0.6272469851978948	0.4496867278907499
0.2128441897807654	0.5359652461810736	0.5277726068878323
0.1526063396210038	0.5483143125617407	0.5262515925244257
0.5913107615013710	0.7206573903310275	0.4866618203886280
0.5613101351053663	0.7851807341944317	0.5483035102506302
0.5855732889604363	0.8415068661102006	0.5502634705007861
0.6397262482353911	0.7534234378353488	0.4767539224781895

0.6646443963573412	0.8096715856633525	0.4795076525014277
0.6376528577949516	0.8541762977599245	0.5163656606024607
0.5859485243536625	0.4023716551623637	0.4950550858731935
0.3181161570540320	0.5793016259709008	0.5257436595423929
0.3960626866159526	0.5816905985773057	0.6344711202509713
0.3673454752230637	0.5390131774908448	0.6776413636743739
0.5505577298555706	0.3794309660575504	0.6432527275093697
0.3789361565278753	0.4500709615927314	0.6334186058387558
0.4188230916670834	0.3974287758059327	0.6428891610208204
0.4481730040181737	0.6215840723608729	0.6458235905296179
0.5798487351827293	0.6035544700702079	0.6446211553763959
0.6197867816432723	0.5511754376021128	0.6339652414090314
0.4955518250809529	0.6384820871563234	0.6021761819040259
0.5067748504876840	0.6978095167926779	0.6138990588323937
0.4784748180700580	0.4841656647304366	0.6390836374027424
0.5473343108079648	0.6730758196488072	0.7186838943122864
0.5378463635277615	0.6131915535228215	0.7060279524089722
0.6313595045577969	0.4598291844609751	0.6752616891304153
0.6912540888448672	0.4546402234382987	0.6629093211059816
0.5202973069502890	0.5168406879170090	0.6390852162666515
0.6777673235267555	0.5021567848639590	0.5591155534341807
0.6176393493930914	0.5088196858942449	0.5723698768080631
0.4614235160930030	0.3860658135504547	0.7037970886151488
0.4524393978967001	0.3258294027801583	0.7147187879890815
0.3210658516042477	0.5022824246166661	0.5591105882363832
0.4922831144808862	0.3040062414156470	0.6088372238026430
0.5030314600173060	0.3636780610906428	0.5988137448396966
0.3812071025885662	0.4951687392190292	0.5720175536391768
0.1211037009268058	0.5227301173969676	0.6253898480653476
0.4307279293264101	0.1262821073444703	0.6477967193366200
0.7370818477487812	0.4854228829601202	0.6528059883544781
0.7830593023148135	0.4656430752390570	0.6846456984487326
0.8441975820519774	0.4596962866362213	0.6866806445977529
0.8776016205279337	0.4659752336787475	0.6298559546122120
0.8488226038354927	0.4785564950520423	0.5714154905664515
0.7877472146210219	0.4845317603324998	0.5694743295868070
0.4478963411951534	0.2669918396930381	0.6195382538335128
0.4059022235282747	0.2248692548842220	0.6110904990286465
0.3936334954393405	0.1645720017129650	0.6132163111960217
0.4917891998918573	0.2094747238650744	0.6786671579849457
0.4800700215890331	0.1492010748476189	0.6801066031514872
0.2621551567833368	0.5094496474630890	0.6530797563987534
0.2151266692133917	0.5666886922502998	0.5970240579807995
0.1537224305182059	0.5678288195812330	0.5957963310212030
0.2124070781558605	0.4758942159905005	0.6586853683274698
0.1508407741480491	0.4766139417552908	0.6569103662790285
0.5514043435168150	0.7338914564540281	0.6251291964412448
0.5099117211147178	0.7905371783687852	0.6876961870143400
0.5225160599013199	0.8505713053448979	0.6905141802987622
0.5926982586945795	0.7759041216738245	0.6154056355201118
0.6058482282454869	0.8359552174236121	0.6188174705878338
0.5707918432839769	0.8737520500500838	0.6564989966900174
0.6026239689132638	0.4194064801454095	0.6322903264370429
0.3072529964559805	0.5443803223573397	0.6656119559552169
0.3835987032762727	0.5586389374375598	0.7749686252312459
0.3625173137388217	0.5120555908045870	0.8189024857375876
0.5720492768022196	0.3883476911440182	0.7838806788434706
0.3908996175034417	0.4251842451019590	0.7778693065849605
0.4404823925593419	0.3816437558067617	0.7885405775863960
0.4278074376116949	0.6070764973984413	0.7873465578813120
0.5594966815228826	0.6137341834708397	0.7903717634543869
0.6089796518797060	0.5703300594434351	0.7784918216477028
0.4799565093283062	0.6339044994277703	0.7437677035191349
0.4818465769695233	0.6938724745733749	0.7567400945007468
0.4815886478937996	0.4785256831919241	0.7817088922149458
0.5062853230097119	0.6732405688952631	0.8683785803180953
0.5065025997589143	0.6125637078078746	0.8546714143023013
0.6380787571157885	0.4823254958756711	0.8165067263262601
0.6976007591057961	0.4859964641116098	0.8019120384268692
0.5182513687754707	0.5170290425341201	0.7817900924456060
0.6740394066054449	0.5305785304106674	0.6985917187286396
0.6140104809080785	0.5274637083757948	0.7137865826427732
0.4944635444673710	0.3813982591359575	0.8524431394976558
0.4948504170873664	0.3203820027453562	0.8643860575653084
0.3244588250891847	0.4679497263025171	0.6998371781054417
0.5175847836355849	0.3024591628194805	0.7517490061586382
0.5192887636666862	0.3626898098552813	0.7404975430596897

0.3847012813932962	0.4698984019480884	0.7141539792063982
0.1219265986999448	0.4687715522239854	0.7794389306789944
0.4701175302900577	0.1205986971432448	0.7971581212152298
0.7351429447909598	0.5231275951688499	0.7912860492404258
0.7809136296459883	0.5279918025444559	0.8261046239002632
0.8409617717318996	0.5396443248883650	0.8309373531933368
0.8756088520391176	0.5440535345252792	0.7748381637766008
0.8490256157856919	0.5367689508811967	0.7141136048477437
0.7888369275879087	0.5253721324322499	0.7091873249702920
0.4873588518932485	0.2601951368164585	0.7675650536049541
0.4628424697985358	0.2152713561603622	0.7430286242342844
0.4506827368957886	0.1551385909640954	0.7448246060989647
0.5134802846258077	0.2078725460925603	0.8459467755714313
0.5018631570437323	0.1474510750746724	0.8473444618758302
0.2642695435439656	0.4729524672814081	0.7933515733589610
0.2078529551738504	0.5047158614359692	0.7216660331153648
0.1469724466787675	0.4995026812391769	0.7273310040740446
0.2198810363893342	0.4488413333118462	0.8208124351210477
0.1589455045384744	0.4436094501436845	0.8263777644049700
0.5129313095399980	0.7359110096841454	0.7736201708140661
0.4862210188784417	0.7861062943357421	0.8533705704021554
0.4985722098281481	0.8462751646397830	0.8573515480615989
0.5385452586959483	0.7813004969919518	0.7512895255451634
0.5514507594326454	0.8411796150522585	0.7556710949995740
0.5316078219618676	0.8742627070113305	0.8089352031558505
0.6161356291652993	0.4370235829923262	0.7719452235496856
0.3027725238636427	0.5096770160306586	0.8050461350346781
0.2241371793920518	0.6899738608157797	0.1705628162779083
0.2422694710544550	0.6511230385485106	0.2298687324625668
0.2108268186105413	0.6652971603475587	0.2283570023874276
0.6408931185966297	0.6819591397432757	0.2606495149394917
0.6797675304392404	0.7230358957431113	0.2311753402257782
0.6795271107596870	0.7480327656862477	0.2576722983413208
0.7007391408698895	0.7751509832175396	0.2538867390058859
0.7013322124471852	0.7256353343176658	0.2013059567461694
0.7223496266513552	0.7528361468783231	0.1979021456838061
0.7221841352600130	0.7777913067380596	0.2242455324483382
0.5377070150130171	0.4358463062760217	0.2135511114200429
0.4989903259986057	0.3959811113307328	0.2201868616597584
0.4349520018435379	0.4665724702656724	0.2206565381723285
0.4285195160452980	0.5218036867654239	0.2123126244292300
0.3962498913583979	0.5068818590842500	0.2126938686363467
0.4001364044477452	0.4734138154915785	0.2181439188936709
0.5765996165431057	0.4842758576649517	0.2133280520775863
0.5699524675546453	0.5394107587367371	0.2223254602401576
0.6048114866960237	0.5325921967247008	0.2207795568536975
0.6088418586493964	0.4991885186581442	0.2150086407390618
0.4673382155692204	0.5702073514868147	0.2140236170558035
0.4728455580923426	0.6058393947334904	0.2162991553004840
0.5058032650861659	0.6099994485212771	0.2224825280363214
0.4844602192059569	0.4290785663512151	0.2207298096375505
0.5320292414958551	0.4001857848072201	0.2146586835061539
0.5204299447833682	0.5769171980757211	0.2223233798389601
0.5548015131330528	0.5707173639758121	0.2257258925749349
0.4356389877573827	0.5558917116091063	0.2098391640654169
0.4500476058558220	0.4352160566296157	0.2237089805812707
0.5695313630406885	0.4501878821848357	0.2103174270925289
0.5774041258976118	0.5991396649003431	0.2333750386003544
0.5818902172014151	0.6251614526832598	0.2084832086682243
0.6027034953436764	0.6517449871286478	0.2166156162462160
0.6193943894165416	0.6532434590551833	0.2500821918144517
0.6149194665784125	0.6273784449172941	0.2750433338418526
0.5945684111349899	0.6005981296109211	0.2666186429242277
0.5987150303871425	0.4281041267626101	0.2041327884774879
0.6100332211957282	0.4060871405818391	0.2309170359109519
0.6380389505851516	0.3864722542560027	0.2250617925843955
0.6551197073881446	0.3879173726159048	0.1919174121988093
0.6434142421887271	0.4093788155240226	0.1647878447501393
0.6159463068689108	0.4294438867285210	0.1709368383313043
0.4273000080951216	0.4066811164503622	0.2302981466356700
0.4096001040137437	0.4046660112648423	0.2631640737520279
0.3890007732441227	0.3778238281815542	0.2705869343725211
0.3847870644831788	0.3524573305089362	0.2449652844649602
0.4019932645649740	0.3545383864609521	0.2118501517985775
0.4230502365984278	0.3811779046597868	0.2047167657117348
0.4065290510471726	0.5778704589738096	0.2025553799100368
0.3924273541548165	0.5783699651357651	0.1676356548521920

0.3646093567823477	0.5976063910055588	0.1603151059420722
0.3493826867698094	0.6161417862309743	0.1879312445330661
0.3635308070692348	0.6160200811578042	0.2227349184256813
0.3920316981861465	0.5974142261160745	0.2297789574181556
0.2014787101959334	0.6847889960228918	0.1986665391816488
0.6842146146240994	0.3663010798637967	0.1823541660802090
0.7292880123237601	0.3322565184168942	0.2105121895370244
0.7437439280719884	0.3240096119378941	0.2441198299133724
0.7695684054236521	0.3011709172022932	0.2458252275408060
0.7814425694572350	0.2861170824492564	0.2141706351032839
0.7671105041720993	0.2943765840629245	0.1808435323506851
0.7412390271937543	0.3172689621846959	0.1785711379365595
0.3628364336452265	0.3237570187997227	0.2544439467229607
0.3238966070631820	0.2837300784023760	0.2234973845669325
0.3027437895844267	0.2819827783281043	0.1932080021332034
0.2810069075989557	0.2554281854997776	0.1891530821957791
0.3230921829934216	0.2584746007165339	0.2496827482052804
0.3011927715657077	0.2320008340995753	0.2452263791766942
0.2800829788212643	0.2302444891603359	0.2152150650188851
0.3190903277308626	0.6356099865411524	0.1773444787751208
0.2649462169150082	0.6561693927827010	0.2013625621319651
0.2557409041458262	0.6759067350128588	0.1715177042580882
0.2096988010259982	0.6679639650389549	0.3298163074318888
0.2190470086398429	0.6091915520745986	0.3684134373978789
0.1877017290008749	0.6236925582427242	0.3667124595079446
0.6102340185428005	0.6993855680002944	0.4009987667304887
0.6461778683868536	0.7436663534756298	0.3756680289202633
0.6345345995791369	0.7714220725936248	0.3946975638616769
0.6535102305742876	0.8003266388797257	0.3955236784062147
0.6767914727658333	0.7451725916587197	0.3576672275914211
0.6955812427513930	0.7741783448844332	0.3587724149955119
0.6840527457792109	0.8018939127247858	0.3777194838142800
0.5477916255360475	0.4405592620331991	0.3568081020544476
0.5163219749379699	0.3946812922928711	0.3629710299176229
0.4412538460788551	0.4532987716726142	0.3637439890367395
0.4250673397354336	0.5066107508712534	0.3560664877918363
0.3959379617297983	0.4862671540848037	0.3571800776321770
0.4057430608014732	0.4539621439511185	0.3620871893191023
0.5776912883684694	0.4949964088743094	0.3566670189773188
0.5614171345939226	0.5481441012443747	0.3653462039076470
0.5969398757562335	0.5475441955594447	0.3640130466790880
0.6068078560350793	0.5153419159276886	0.3584570023850258
0.4549294800489565	0.5610405246911615	0.3573568166176675
0.4545522960905702	0.5971584010989246	0.3586073106862304
0.4863754505699238	0.6068264298697102	0.3644129060686630
0.4964453984709734	0.4248528006047820	0.3636154039902325
0.5481924746358257	0.4044299353256045	0.3576780038584110
0.5062110790540182	0.5766108393481297	0.3650123920273852
0.5410994275353911	0.5764065974629564	0.3684943893203035
0.4260576336309589	0.5414616817989573	0.3537769887485594
0.4615149839582786	0.4249617665338552	0.3665560233702657
0.5766918124868402	0.4601776934390244	0.3537531152759203
0.5586373933896188	0.6082834977821199	0.3758224273445022
0.5591381910658467	0.6343232688917602	0.3505143248988686
0.5762432552009462	0.6636399008594820	0.3578202886522298
0.5926325127965264	0.6679605527220458	0.3911003406312170
0.5917427227401286	0.6422590161920131	0.4167276763540270
0.5754370341454287	0.6127032939702420	0.4089646154254206
0.6092469692902004	0.4435749002630346	0.3475627669290620
0.6235077904749021	0.4223452472508559	0.3734448892235751
0.6544309537872278	0.4078769885664319	0.3673942813708111
0.6715599904799950	0.4136807136577342	0.3348518591934838
0.6571448296248484	0.4343931216750923	0.3086448765975607
0.6267208889806253	0.4494951059568781	0.3151248165807684
0.4437845805524324	0.3929941347226572	0.3728529959684548
0.4261398897618709	0.3881937521181928	0.4054030220208972
0.4093819695803285	0.3586748243992699	0.4121072223833950
0.4089081178024485	0.3333795469411102	0.3859890478879839
0.4261668166480143	0.3380592104291755	0.3532958346227318
0.4436878950052064	0.3673418368516648	0.3470456815301416
0.3935464206634788	0.5581708077814119	0.3475864369111403
0.3764184862031557	0.5529001097073929	0.3147874684989108
0.3462061394717726	0.5683660323686242	0.3081592256789744
0.3316714645540269	0.5888028519671115	0.3345499022627708
0.3483299520487068	0.5938003434246427	0.3675355431031993
0.3790738854860255	0.5789782891242612	0.3737376674054992
0.1828266505515203	0.6530881770037728	0.3473658750946955

0.7038832221249020	0.3974003965351597	0.3255364379315048
0.7566695514068007	0.3773249511278688	0.3532002867630936
0.7687429933608766	0.3643737827179087	0.3860682143721854
0.7995116452557666	0.3487201534013753	0.3872368494111648
0.8186248907567694	0.3457135403025507	0.3557366930539886
0.8066804420438161	0.3588137718322172	0.3231335295118975
0.7759514049683344	0.3746397446500030	0.3215649453058148
0.3906968244645010	0.3020369463351951	0.3947980047226423
0.3543612656373581	0.2589334136301381	0.3678471049355633
0.3239489791188760	0.2583868800433360	0.3493818456616077
0.3045204407298008	0.2297820917758228	0.3496256412258255
0.3651820147017260	0.2306134437528779	0.3864564356843627
0.3455865943056456	0.2021094182329666	0.3863973648510745
0.3152234887356489	0.2015046734122746	0.3681486315438460
0.2997601284325091	0.6057934922664021	0.3251989493705704
0.2458904186847799	0.6240396104560274	0.3504849623685668
0.2411454470487499	0.6536293587671238	0.3311656331325791
0.1796789999148094	0.6069504006958855	0.4666465079577273
0.2033345980744434	0.5556911823482817	0.5103359500408996
0.1695579500881004	0.5629398078326630	0.5095881437989772
0.5715691013370041	0.7158583011451689	0.5380267314387536
0.5992109952443303	0.7656562413977170	0.5126651819668014
0.5839025361166819	0.7905824682530328	0.5332639973564267
0.5977943430091203	0.8222397732336695	0.5343110792923800
0.6282256820694063	0.7728055833772832	0.4931698952267174
0.6419467294645930	0.8045062767078941	0.4946262536044397
0.6268753129169110	0.8293741278455316	0.5152450981306618
0.5550298829898782	0.4494954107298288	0.4972389079750242
0.5321589442897174	0.3987410624115209	0.5030626191528329
0.4479219784946684	0.4431953100302738	0.5040036947352076
0.4227018755634038	0.4928229707671061	0.4958599696968469
0.3975779242829977	0.4676803638243358	0.4970276874001478
0.4128543045212003	0.4376120679567540	0.5022304914318017
0.5748330903069692	0.5084094834972450	0.4970653869529885
0.5495249029983973	0.5579914335094222	0.5052499175288936
0.5846037655105606	0.5635895941864787	0.5037865204354498
0.5999327091782738	0.5335615156019649	0.4984974385074170
0.4425528795738721	0.5517247880264361	0.4965383120108415
0.4357600119460066	0.5872183557235425	0.4972182990315233
0.4653203905399660	0.6024588280696868	0.5030607750224654
0.5072675635472260	0.4249293032128793	0.5039437864423594
0.5618046136317569	0.4139957539806001	0.4978342968105359
0.4902145156873740	0.5762833617325465	0.5042219178226823
0.5245790207437264	0.5823439954813268	0.5078212520412506
0.4176136369485950	0.5272983304266960	0.4930459305403158
0.4728513793209232	0.4188568702510299	0.5067996029839661
0.5799795950570895	0.4739520937997115	0.4941637077174026
0.5363199450101296	0.6169123534736388	0.5144559078129161
0.5319533074368675	0.6423979484213637	0.4888963942878571
0.5438810937350689	0.6742991702991564	0.4955760723136528
0.5597841153660830	0.6817378614890218	0.5284619000513585
0.5637757056728144	0.6565156339633142	0.5543054486259509
0.5526878167222022	0.6245209408002335	0.5471718572696944
0.6148885402105926	0.4633376538425634	0.4874757207386471
0.6329862837318162	0.4448363024477116	0.5129921960334543
0.6657980145168908	0.4357942899079804	0.5062310900084438
0.6811166126661911	0.4443758014956521	0.4733362539259135
0.6629822447471748	0.4624412668644984	0.4475563353002096
0.6305815848134804	0.4722121762408674	0.4547560941026767
0.4610902012980616	0.3842020812896366	0.5127392667469290
0.4444856892361659	0.3760397406129942	0.5451432064796154
0.4335625000884479	0.3438642695355938	0.5515846224218517
0.4379117393911230	0.3190787800294052	0.5253000532220444
0.4539844248214930	0.3271043371830881	0.4926762384917811
0.4657925086231304	0.3591650827977820	0.4867308390380241
0.3827335842869282	0.5379745555928177	0.4862691859778290
0.3670959936340387	0.5293251848604082	0.4534429497662519
0.3346767004018530	0.5390755916222019	0.4462938265917086
0.3165057028056686	0.5569024137811887	0.4722362240855976
0.3317615521707803	0.5653139073591117	0.5052067331480254
0.3645908421759029	0.5563066207911990	0.5118975868807673
0.1575063560337003	0.5885424620978812	0.4876933644345114
0.7155011713841746	0.4335634833157972	0.4633706812470835
0.7717053034752455	0.4243271053611340	0.4902422566146828
0.7869979381529596	0.4176567420539933	0.5237684518745038
0.8201091357138277	0.4079679902002025	0.5250455287564207
0.8384397677177504	0.4047370084142590	0.4930086539678676

0.8232517145942038	0.4115708977375145	0.4597470818919880
0.7901132405061803	0.4214031923076019	0.4580212849277784
0.4263732991458511	0.2847064017733753	0.5340933839128377
0.3992885761434698	0.2351414853395163	0.5071253106339606
0.3711514326062940	0.2280404826937295	0.4861098846470521
0.3577225644422375	0.1961944801263112	0.4862589170740959
0.4140394556028554	0.2100171664446133	0.5279211697569258
0.4004468663635355	0.1782112926812456	0.5276472022005658
0.3722292856025546	0.1711189635266177	0.5070658180102587
0.2819344566715267	0.5673238001340399	0.4627373573442206
0.2254679512114184	0.5739916650297712	0.4888049240927280
0.2135215137115234	0.5998571701905692	0.4669516838482842
0.1664281072702822	0.5474489083623714	0.6094667517193728
0.1993151314139724	0.4959168246111667	0.6448127077199090
0.1647640085759434	0.4965293330346457	0.6438456462913482
0.5337668340883887	0.7250031918173903	0.6768228866785365
0.5507693253048368	0.7793823410991187	0.6516840921559317
0.5309040709656803	0.8005346319780544	0.6726960375516857
0.5382124219222862	0.8343107373797860	0.6741872609496733
0.5776143757593466	0.7924004020268309	0.6321194896369985
0.5847223675560037	0.8261962793824080	0.6339562053254445
0.5651530084630899	0.8473187259889855	0.6550417917995868
0.5638142516390832	0.4602733975097749	0.6348365804889189
0.5503957947953860	0.4062432815983462	0.6406548859177207
0.4596387971362254	0.4349676678483472	0.6425016302538856
0.4259834412792251	0.4793745741830961	0.6349335385783494
0.4057296745861071	0.4501690289507923	0.6359687991234304
0.4261148327193663	0.4232535668097235	0.6408379847701037
0.5727651294952527	0.5217730607383443	0.6348988217123513
0.5390886814762450	0.5659948480858353	0.6435424657252196
0.5725887651385375	0.5777648533451955	0.6420212463959230
0.5929971936295487	0.5509765566254994	0.6365251476031684
0.4349497507656610	0.5408701704067165	0.6359512790041573
0.4219088583691649	0.5745637946725005	0.6370982461512659
0.4483365404688510	0.5948123542577444	0.6427028127661195
0.5212440935947700	0.4275520346723861	0.6417954614172310
0.5768182232928309	0.4265527178684060	0.6352749745137932
0.4775065067582707	0.5734920612716258	0.6433019825471579
0.5102676056371004	0.5855303380673901	0.6465382615633734
0.4148081648288346	0.5123880782666090	0.6324502959256530
0.4884974909902565	0.4154155345013645	0.6448905165603438
0.5839656965491576	0.4888073067243655	0.6317304671047823
0.5158502630752562	0.6215779308785436	0.6532426334391261
0.5070567702798514	0.6459295660723281	0.6277424637078768
0.5135119752138667	0.6793830111014770	0.6343437533215434
0.5281576978956000	0.6893731053459392	0.6671092464348969
0.5365179088834926	0.6652077021760785	0.6929121394029757
0.5309270554280382	0.6318038038873393	0.6858477871661932
0.6201640844022158	0.4847258718522771	0.6247553686466751
0.6414424628257852	0.4693365833910347	0.6497969297328260
0.6753214089139414	0.4665987471030751	0.6428142297124694
0.6887103628426273	0.4785647890739922	0.6101736903490182
0.6674737822287461	0.4934664655935456	0.5848268491957759
0.6338510536881783	0.4969269913689704	0.5922377520061076
0.4830258848818454	0.3791861828270765	0.6505579977558295
0.4682649531803045	0.3680295627207653	0.6829668263699918
0.4629453177204352	0.3344142402220142	0.6890681696867700
0.4712424501310394	0.3109421654359895	0.6624753892061863
0.4855469189563644	0.3218680701216676	0.6298833500167759
0.4917355249785174	0.3555282008478677	0.6242408192401249
0.3785720111936527	0.5166620205706325	0.6257230805252488
0.3649486303242244	0.5060665541943271	0.5925142616020004
0.3313130029184719	0.5098004872925210	0.5852857678553138
0.3100629307205514	0.5233169460730769	0.6115248484678412
0.3233620284947519	0.5336371965512857	0.6448735123009831
0.3572688375039266	0.5307009858605114	0.6516706426592253
0.1481428147226273	0.5222732855149073	0.6261528054094619
0.7244929125978067	0.4747595253991232	0.6000793149188339
0.7814346652101171	0.4752933372398564	0.6269193074850646
0.7976507375287293	0.4683290496148200	0.6598417236563184
0.8320162538489497	0.4650902885021792	0.6608543607356908
0.8506900415716075	0.4686340521856888	0.6291016209890126
0.8345490646259450	0.4756420081329665	0.5964217969489933
0.8001407444369109	0.4790794764375143	0.5950156646938664
0.4660586552325914	0.2750219046622810	0.6712540611616015
0.4493731134224880	0.2210044258342915	0.6450480363861351
0.4218836277739915	0.2081389329340276	0.6264649546931593

0.4152713496826776	0.1742145475181830	0.6275512195900836
0.4703281304602638	0.1995880625792103	0.6643822812029531
0.4635289577534673	0.1656839894465452	0.6651154908905627
0.4359831235800136	0.1528176552291523	0.6469189036105342
0.2741302290744024	0.5270041659861123	0.6021985830729525
0.2175770835171891	0.5211510114642706	0.6278714847897096
0.2009979334863229	0.5470383653924161	0.6101163305381522
0.1628340639086931	0.4881808443878554	0.7479145647902745
0.2037543129940722	0.4599888105780519	0.8002835484295100
0.1694874194765410	0.4570290638017502	0.8033577564867681
0.4962800672387169	0.7234810247066314	0.8246920329528242
0.5116093885535355	0.7798243452328149	0.8021404057249887
0.5002041406984181	0.7983544781258423	0.8319987685515050
0.5074974226420986	0.8321388746857254	0.8340023225598039
0.5299017779573276	0.7956731710014011	0.7746198083507013
0.5369767019973517	0.8293911229740246	0.7771542659270400
0.5259065601986682	0.8478783420611291	0.8069067551929582
0.5709110710687578	0.4704542212130424	0.7769204477276600
0.5673192634964905	0.4148120230248509	0.7820673376452166
0.4730835842850031	0.4264214335825085	0.7863132452438222
0.4313604453804020	0.4631352052917484	0.7778448769109644
0.4171882643067487	0.4305752161721810	0.7798676758718623
0.4424758819550538	0.4083297897360179	0.7854348511582695
0.5684719991479363	0.5324463365861896	0.7779096699434592
0.5268363583701094	0.5690371126114172	0.7874238747327581
0.5574564390740571	0.5871119992653446	0.7867130037515715
0.5826915711187816	0.5649464592699704	0.7805299033128930
0.4289156244297885	0.5251439407220362	0.7782536976270695
0.4101883136413641	0.5560811772548192	0.7786295450345236
0.4325431816167273	0.5806687758045456	0.7847754024546144
0.5349950570699638	0.4307066608270969	0.7847919092795402
0.5896074073194618	0.4395066910816525	0.7761527490762211
0.4649152827203149	0.5647645175608456	0.7865181299156391
0.4948129028648764	0.5825870015070284	0.7904744829007277
0.4142606397627993	0.4934358192076268	0.7747757006058874
0.5051338924319305	0.4128366827689500	0.7887674233273149
0.5855409815531547	0.5022030298100607	0.7740064309031718
0.4934882088012504	0.6189969905825925	0.7981503209246298
0.4861071308087572	0.6423664320024246	0.7711219992106074
0.4872568655756955	0.6763266793892548	0.7785723142368984
0.4949857300253449	0.6876219180128624	0.8135900933540671
0.5011734684188357	0.6642292194069935	0.8409871795398458
0.5009353343381013	0.6304809033862107	0.8332728023056031
0.6217317483473619	0.5046640673816745	0.7662249339143281
0.6458629582612464	0.4932410052611492	0.7907366462784611
0.6795194431452212	0.4957314996310209	0.7825177358503053
0.6899946860396321	0.5093268240461887	0.7493059351502600
0.6659833367080642	0.5204555702060969	0.7246294689697657
0.6324465803631446	0.5185605177592962	0.7331962120437242
0.5065572998234953	0.3762884954838125	0.7955141153655177
0.4996729571506963	0.3639915418493956	0.8304372633467471
0.4995361321051185	0.3300639505525129	0.8371776141463665
0.5052728928022172	0.3073340878866386	0.8090037637589457
0.5125123734133029	0.3194596271244603	0.7741912800060098
0.5135449229811498	0.3535724239914497	0.7677041492771256
0.3779493777700578	0.4912012166348480	0.7675303671104464
0.3666361395872944	0.4783736455431360	0.7342079010521568
0.3329573445405183	0.4771235015439733	0.7261274537712534
0.3094092886877691	0.4878966803217440	0.7515299127857112
0.3204945719955756	0.5002580246086231	0.7850876885678094
0.3542740597780928	0.5020355444147215	0.7928722847719174
0.1487869717488769	0.4710783730207393	0.7770920804465477
0.7257066485330086	0.5122698399602622	0.7385755793010731
0.7809923915739011	0.5258397680358196	0.7672626627116788
0.7959675886654577	0.5299609208364930	0.8014748251962214
0.8297513255517376	0.5364898966220762	0.8041661353367703
0.8491201376917072	0.5389487659961688	0.7727886169599031
0.8342058137194789	0.5348529755700893	0.7388541772701774
0.8003372574566602	0.5283792456542409	0.7356884440341408
0.5039846597142841	0.2712112673935154	0.8190118374138589
0.4889307943409245	0.2154553894557596	0.7944356954399474
0.4712952116186395	0.2002701883274917	0.7659630999877887
0.4646207862254345	0.1664090770826610	0.7670569281563561
0.5000361137307421	0.1961355108560950	0.8238283127363228
0.4931740788620948	0.1622161792089929	0.8243614253642463
0.4754674089101462	0.1471142839083725	0.7962855987687579
0.2734859255984069	0.4866098674107091	0.7413152855530027

0.2178384515915515	0.4771026273786594	0.7708143040284385
0.1971418367157713	0.4913510316495076	0.7444670763663959
0.2962491706095735	0.6408310685943718	0.2045443261185772
0.6584611004955968	0.6953387424457562	0.2321887095919330
0.5084646615262797	0.4528984089163393	0.2170352071914422
0.4509902706319099	0.4964791933465702	0.2170764363268786
0.5540127626060118	0.5095575180211567	0.2177023337027141
0.4965609880204659	0.5531396780202086	0.2175756672136468
0.7028957351185724	0.3551600700965936	0.2111406434795826
0.3458230367651430	0.3109059777615808	0.2252979615524802
0.2774984852121842	0.6089067856405435	0.3531648634366717
0.6274035555065662	0.7140624186982353	0.3731668768611441
0.5160941604396404	0.4524146912709590	0.3601672649880533
0.4517169717950400	0.4855959819447993	0.3599860259383542
0.5510064982499923	0.5159190506426000	0.3608028506382844
0.4865788188378404	0.5491210234366111	0.3609556230886711
0.7254361875964025	0.3932804686754936	0.3539078381969121
0.3737819867178496	0.2881741551731964	0.3662707357235510
0.2595861291308383	0.5659713447765399	0.4907512328269532
0.5858040958979119	0.7332737456431440	0.5101189677552445
0.5217492218127937	0.4555381378713554	0.5007284813679700
0.4525816002445562	0.4768211877754343	0.5001525224148723
0.5449157638709614	0.5243777146009880	0.5012287610695959
0.4757962478245155	0.5456849600692599	0.5005295458749144
0.7381258679108458	0.4344109892093737	0.4910304511503261
0.4123768267302623	0.2677029366037920	0.5057427578356873
0.2526175199467779	0.5200661602708919	0.6300386253521488
0.5440630643952591	0.7450044233330018	0.6488786652570879
0.5300145263337204	0.4602672130282299	0.6385597434428545
0.4582426474654580	0.4689096809858260	0.6389537443268632
0.5405056182402698	0.5321260785016474	0.6392281438695870
0.4687614655850714	0.5408382279409587	0.6394908829848076
0.7465594579052427	0.4788774194408051	0.6279726825749129
0.4556498080061041	0.2555287108327722	0.6429423330172561
0.2526750994795104	0.4798042606465522	0.7699646759054600
0.5057155030621653	0.7455262192808594	0.7980728663081387
0.5377627544794908	0.4644776648721516	0.7817470172576103
0.4650401241980914	0.4593329309359658	0.7818291719623589
0.5348234467941780	0.5361994556619836	0.7822039678289983
0.4621256226782427	0.5310567301855638	0.7826651627028105
0.7465483897780821	0.5199536983492137	0.7668574964332588
0.4945283328887377	0.2499001887173288	0.7916962200153316
0.6899718166986204	0.3593651525821444	0.1501704161716849
0.3602824429164715	0.3130616456526082	0.2859821257213208
0.3154448827578906	0.6452160444398003	0.1456409376498820
0.6425656988664468	0.6922505283302061	0.2924188640306346
0.7097672112745397	0.3886008189929532	0.2937457838287822
0.3908332736021621	0.2902528608751585	0.4260593045649891
0.2945513786600647	0.6159230090359487	0.2937837859216164
0.6093447893127708	0.7105767812688168	0.4325143839072220
0.7219853975909939	0.4246242633439066	0.4317249114715382
0.4293203974522649	0.2732644513584099	0.5653648452706865
0.2747314468787619	0.5760577020174745	0.4312519672875056
0.5686483567899582	0.7267626271173885	0.5695230533162250
0.7326801826958905	0.4683556122745110	0.5681898558010180
0.4709136813809570	0.2644996236250267	0.7025831355336241
0.2650843244020904	0.5352473258617689	0.5711525229467789
0.5293517718653257	0.7348593698764885	0.7084865811002016
0.7350033094561136	0.5086690860088712	0.7066107702721840
0.5104207113773985	0.2622708291052526	0.8504124719358317
0.2638324426832491	0.4909944246774099	0.7096215744504814
0.4898625902819916	0.7315830637349120	0.8563661193566511

```
C-CENTERED HEXAMER
   1.00000000000000
    40.4062059999999974    0.0000000000000000    0.0000000000000000
     0.0000000000000000   40.4090679999999978    0.0000000000000000
     0.0000000000000000    0.0000000000000000   41.8059700000000021
   C    H    N    O
   432   300    48    24
Direct
  0.2245560910164219  0.6909821257943695  0.1520573669723204
  0.2422596714463161  0.6515981725613192  0.2039691072514266
  0.2110765314312004  0.6662477102394206  0.2028386405650975
  0.6381506104358576  0.6843057441883292  0.2269185305638640
  0.6747223149624333  0.7269395485189507  0.2004700663236336
  0.6746256722016871  0.7513372406011568  0.2243404200816292
  0.6948344051371015  0.7791208167618753  0.2208698412753247
  0.6950543822566028  0.7308645217205707  0.1734481216583092
  0.7150765567211687  0.7587220496491902  0.1703255971771725
  0.7151180089463869  0.7830427660046090  0.1940919333974083
  0.5377126300651794  0.4363295120252786  0.1877440735379836
  0.4991177104242256  0.3963933202258057  0.1931063261945287
  0.4349443813086414  0.4666689384729026  0.1936874614079070
  0.4283403122757876  0.5218351527914422  0.1868035666748830
  0.3961708965945681  0.5068195364832712  0.1868944066858701
  0.4001599765303790  0.4733833881278023  0.1914099795568163
  0.5764118155563970  0.4847838606851138  0.1877109173357688
  0.5695855757117860  0.5398409131194977  0.1953810310127172
  0.6044122558972003  0.5331689292478267  0.1938827584760087
  0.6085515636183649  0.4998056918601081  0.1890052556509111
  0.4669296201493124  0.5702413293935182  0.1888165149115849
  0.4722732817413406  0.6057957517325815  0.1912172928331971
  0.5052070731044906  0.6100344210846886  0.1964207214433815
  0.4845134641460417  0.4293948433683298  0.1937163537317186
  0.5321307660689817  0.4007086526207992  0.1884810649310086
  0.5199692311875995  0.5770785847383677  0.1957808370265377
  0.5543584088170983  0.5710406182762782  0.1983612801482613
  0.4353251325606574  0.5558996539491671  0.1850021621146091
  0.4501100948497394  0.4354028335153429  0.1962080825797195
  0.5694760491124243  0.4507267329240802  0.1850649193783391
  0.5767805314052805  0.5996777126099144  0.2044566421419112
  0.5802899208930756  0.6255214555468239  0.1822389593306587
  0.6004996969108481  0.6526430155994016  0.1888323765903195
  0.6176080315327257  0.6548512661200290  0.2180094603104586
  0.6143327167017423  0.6290190105542508  0.2401704146412783
  0.5945625714652450  0.6017134627439494  0.2333216613471631
  0.5987106793719412  0.4287508859294936  0.1797589197914795
  0.6098967944974318  0.4068113220778476  0.2034258840404393
  0.6378594530112849  0.3871907684332992  0.1984219734782688
  0.6550764665446814  0.3885671588612506  0.1693331082734863
  0.6435643345223093  0.4100173646048119  0.1453886876358828
  0.6161068279886384  0.4300707077634599  0.1506352385137254
  0.4274415016785071  0.4068038090830698  0.2016351259343309
  0.4094917266392026  0.4044904742144501  0.2303919008255336
  0.3889948523459862  0.3775442634137199  0.2365456321134565
  0.3851633243607867  0.3523422174100548  0.2137975785651250
  0.4026170184596105  0.3547390042197928  0.1848183291550383
  0.4235339658052082  0.3814995613578916  0.1789032941113017
  0.4061738276159290  0.5778154753514013  0.1788691301326825
  0.3921606008287605  0.5788254399685976  0.1481116477546323
  0.3642892029824720  0.5979955698467871  0.1419603733180184
  0.3489464887787087  0.6159873046522510  0.1665493815650740
  0.3630109181128240  0.6153562239717408  0.1972058762091665
  0.3915355833741559  0.5967890499010132  0.2031342735945523
  0.2019535394741680  0.6860242257110714  0.1768556078237140
  0.6840133710804158  0.3667539510821858  0.1610572936986102
  0.7284376821110959  0.3321887152051688  0.1860822821410613
  0.7425747381424302  0.3237295347598452  0.2157414113268450
  0.7680654898219885  0.3006101002938035  0.2173883224434289
  0.7799112659427342  0.2854497229510216  0.1895895818345243
  0.7658754296580972  0.2938864587416340  0.1601831938781560
  0.7403475477196367  0.3170717857231419  0.1580416443565069
  0.3635018330724746  0.3234162031379598  0.2218793640020678
  0.3265879355722565  0.2820749048759840  0.1939135331209781
  0.3071222363597647  0.2789608196952537  0.1662061091517094
  0.2865392774173037  0.2516522777596966  0.1621106984370059
  0.3252177737412847  0.2574185770992711  0.2174972283051961
  0.3044838357033883  0.2301767092206347  0.2130420961040035
  0.2850684418990861  0.2270588073031933  0.1855729691325013
```

0.3187002850996616	0.6355335477494640	0.1574468140807153
0.2648939169316324	0.6564566361166944	0.1788357778746483
0.2558909649160553	0.6764397707856847	0.1526982574642718
0.2110127406417029	0.6700999126346889	0.2927470638549360
0.2194422033129498	0.6118116992623164	0.3275614702672417
0.1885775909781893	0.6272347486009365	0.3265741678157162
0.6069486391589249	0.7014736468742955	0.3499042450003240
0.6420891184518774	0.7462219529527694	0.3276540433607401
0.6294357280377806	0.7738470849216981	0.3438228185281194
0.6477008088899817	0.8031322108775528	0.3446299040875138
0.6729696062602594	0.7482272550114714	0.3124495309920222
0.6910522959628463	0.7776136091632673	0.3135000313023078
0.6785246537257520	0.8052011655659781	0.3296072435841533
0.5479492286347556	0.4415745827940133	0.3124686490866857
0.5168676112819002	0.3954200372935712	0.3168403514402037
0.4414519673574876	0.4532740959577753	0.3192704602711129
0.4247289807101929	0.5063999164496724	0.3132871791079305
0.3958370152543867	0.4858123115313035	0.3143647266757759
0.4059731084265751	0.4536075697449434	0.3182000882155938
0.5773904540831846	0.4961606505858636	0.3130057096750193
0.5606556221849659	0.5491267623723052	0.3202498519302205
0.5961323370737067	0.5488042673530050	0.3193036014428559
0.6062879011035190	0.5167035076983537	0.3147294572805324
0.4540966414276259	0.5610043131306978	0.3142394784444265
0.4533861969816286	0.5970564485081105	0.3153050735971394
0.4851561542482934	0.6070115261216604	0.3198588219434699
0.4968124825372048	0.4253709519141195	0.3182000741955366
0.5486104806853827	0.4054980981381314	0.3123651669205836
0.5052564955954850	0.5770400168457118	0.3201341431430510
0.5401280328744716	0.5771805225913069	0.3228112355879961
0.4253937062512949	0.5412042261004100	0.3114962599700089
0.4619593087056323	0.4251485128599902	0.3210898667183899
0.5766850603410858	0.4614089115444575	0.3102643801250052
0.5573451900014098	0.6092685344228427	0.3287609515313712
0.5573114766149431	0.6349930825957042	0.3061752199112908
0.5738601448932293	0.6646505197521999	0.3122310216018463
0.5902164373773313	0.6695948712130795	0.3414339994337867
0.5899999205145535	0.6441483022041378	0.3642275459166422
0.5742466165199351	0.6142691088242160	0.3577781040874122
0.6093358418100394	0.4450340313555063	0.3050844341747086
0.6231307783088479	0.4234143874617660	0.3277554977372449
0.6539959713645261	0.4088632088597512	0.3226445365577036
0.6715465728253400	0.4149689010510021	0.2943138923003551
0.6576705058166125	0.4361818667079036	0.2714044523369182
0.6273038636461578	0.4513627620251761	0.2769167602305204
0.4446038002270908	0.3930041586503656	0.3263196820245807
0.4273378975542984	0.3876234137683118	0.3550753996707485
0.4110039116830637	0.3578936447239687	0.3607472387505463
0.4105644754664439	0.3329788966378672	0.3374207911629799
0.4273894316120341	0.3382474437514715	0.3085249573890627
0.4445203817728909	0.3677398137305038	0.3032536560199244
0.3927519928032480	0.5576814034625251	0.3065838930844060
0.3751903662076821	0.5523588649824254	0.2779774568881993
0.3450117533994216	0.5679091246150767	0.2725026183168522
0.3309504174578891	0.5884584979725415	0.2958641087825191
0.3479653435466357	0.5933862353691775	0.3246948918643552
0.3786734401194676	0.5784760102007274	0.3297915343645381
0.1841642865266436	0.6563980428010988	0.3091387619017180
0.7036695162713577	0.3983412246646646	0.2862464514710037
0.7558913378507083	0.3774310265725731	0.3109094243276656
0.7677997094808756	0.3648533140363429	0.3400495223502770
0.7982330007427515	0.3486992220347457	0.3412963488998616
0.8171829481737698	0.3447979334446358	0.3135812319192193
0.8054182874088363	0.3575341929070888	0.2846829637949960
0.7750332046936704	0.3738902030052411	0.2830828115630091
0.3929061598334554	0.3013555576372939	0.3450184683839132
0.3560588188754616	0.2586857070529790	0.3213355844885566
0.3255561809772606	0.2583073803015794	0.3053122446469242
0.3062344627556159	0.2297062227441412	0.3052622123847706
0.3670545328850932	0.2302029021153076	0.3372581966678447
0.3475464229042722	0.2017092438439167	0.3369505628948655
0.3171140375055875	0.2012602480067893	0.3210777282589863
0.2992764023559588	0.6058795306107966	0.2879014555681048
0.2462492556654951	0.6254820625168036	0.3108331169035732
0.2419756453100291	0.6548443604698327	0.2934222836831606
0.1807556209057582	0.6086625270895263	0.4141696498234655
0.2031098855657891	0.5542405592941447	0.4490192431077983

0.1694887876691632	0.5619860366481555	0.4484518749971533
0.5712068092524766	0.7156869913252528	0.4716325068669888
0.5972095965157013	0.7660486490658401	0.4487758946725638
0.5818912242346864	0.7905733428488608	0.4673234594626434
0.5949958432268111	0.8225109896769373	0.4678080401737582
0.6254574004434666	0.7738576507823277	0.4307814546066823
0.6384155490824009	0.8058260292476424	0.4316307877615667
0.6233117402214522	0.8303120202883617	0.4501660914218258
0.5552572441774557	0.4496893795570605	0.4362626403281729
0.5325717991982930	0.3989604093697098	0.4413717973086367
0.4482787611123903	0.4430575627781048	0.4423154078265074
0.4229044466800419	0.4925843126848773	0.4357049407970752
0.3978986607578424	0.4673983903282501	0.4365962743230524
0.4132700443489979	0.4373878878109091	0.4408105138379101
0.5748839921991072	0.5085467626525955	0.4359509886980867
0.5494906707950956	0.5580224477793237	0.4428453427219958
0.5845062390123954	0.5636811931599867	0.4415551335271670
0.5998907568981239	0.5337073501393046	0.4371546137193560
0.4425795911058668	0.5514573645716326	0.4363578601322827
0.4356969277062182	0.5868710962748094	0.4370749659263849
0.4652475994566713	0.6021781241613428	0.4417619259825085
0.5076182111139632	0.4250194085035038	0.4420418164879352
0.5621404523711071	0.4142812760366161	0.4368709876374833
0.4901986232261777	0.5761266356227667	0.4424423591408714
0.5245338759615193	0.5822716294081559	0.4452246338430957
0.4177082655803918	0.5270004154413470	0.4335463502523934
0.4732694035799728	0.4188374806907068	0.4445883699114727
0.5801277472757055	0.4741508841708240	0.4336270003727702
0.5362134095751085	0.6168052776819732	0.4509967018550407
0.5314939035315456	0.6422882761709238	0.4286113222027397
0.5433152174604489	0.6741742900556601	0.4344570941775128
0.5595116656751810	0.6815917909536116	0.4632546257482392
0.5638754672142345	0.6563591594310256	0.4858841392406788
0.5528499348524355	0.6243958735984958	0.4796467010134978
0.6150705082685735	0.4635906537307549	0.4281619851482237
0.6328864427224700	0.4452359141641624	0.4508995832250828
0.6657201574818831	0.4361823657188442	0.4453556709075168
0.6813548268179587	0.4445948787379724	0.4165286995373462
0.6635241304743678	0.4625553230438609	0.3935818413402064
0.6310996634619598	0.4723428054501297	0.3995148004862629
0.4617095475869860	0.3841734685313932	0.4496970887538637
0.4449719875939429	0.3758035036197845	0.4780797635698231
0.4343057762902608	0.3435702105969879	0.4835934294077425
0.4391145707068430	0.3189010934748763	0.4604656559616918
0.4553654799346225	0.3271280838827208	0.4319140754388726
0.4668440373007731	0.3592583736203435	0.4268113119552868
0.3827691991536920	0.5375861989830852	0.4280841781896551
0.3667826978068090	0.5290186807027302	0.3993608180872548
0.3343195334658147	0.5387406857904390	0.3934931655472605
0.3164546780871693	0.5564260436020537	0.4166079179060544
0.3320217868404779	0.5646620334649936	0.4455105650832306
0.3649002557585141	0.5557026776929574	0.4509599050929093
0.1581038875462683	0.5891795514430106	0.4309964293335852
0.7157230371648438	0.4336562649882993	0.4080708940237368
0.7716059023010967	0.4240288650985969	0.4320155220595657
0.7865270017228823	0.4167175055430210	0.4615469961126014
0.8194781049846204	0.4066840330596779	0.4628080246096413
0.8380201791516948	0.4037570760030850	0.4347242598193200
0.8232138060361914	0.4112418530260837	0.4054253465666288
0.7902391051536545	0.4214139561903177	0.4037792948658593
0.4279563290014707	0.2844377088309436	0.4680289636451199
0.4031872267740096	0.2341018545430791	0.4436971305866539
0.3756396728658212	0.2260971503697984	0.4247841028394421
0.3633584792762310	0.1938528702494192	0.4246434915412063
0.4185116854909149	0.2094985263904750	0.4621428966767934
0.4060818170917079	0.1772931335381267	0.4616302276483155
0.3784535569965772	0.1692812224088025	0.4430874768641327
0.2818821776041761	0.5669834075597646	0.4086489928655138
0.2257233339917978	0.5736669104667821	0.4318220392429132
0.2144353667895012	0.6010723769771439	0.4143712312527789
0.1673750738857656	0.5506194055092997	0.5359313774168109
0.1986285728791107	0.5011733969613070	0.5713208428599642
0.1641867184657473	0.5032805273323375	0.5709429820844100
0.5332634306647999	0.7243353132631900	0.5933343008092264
0.5515587316828976	0.7778780343782958	0.5704993460876918
0.5314328793667580	0.7998745389822927	0.5876588007868185
0.5393361167556751	0.8334752062523295	0.5883202933462280

0.5793306592139245	0.7898262124068410	0.5540199537328467
0.5870664960146377	0.8234414117906078	0.5550200489906809
0.5671761048743673	0.8454307347775621	0.5722109346980940
0.5641672625238163	0.4601259801237992	0.5578410868409014
0.5509900543210865	0.4061182874454710	0.5627440405850824
0.4601680048164173	0.4343600810218988	0.5639459192691113
0.4262254002707261	0.4784620459700051	0.5573810204156409
0.4061946007410966	0.4491758624213635	0.5584347668156294
0.4267595116239905	0.4224550142988724	0.5626254945431871
0.5728244559587835	0.5215402492686918	0.5579487683811424
0.5388888647876108	0.5655056509801358	0.5652170301368525
0.5722931831016931	0.5774447132524326	0.5640795692825057
0.5928560364244919	0.5508054223154371	0.5594356251476665
0.4349261794717157	0.5398711633039199	0.5583177769952953
0.4217972220319480	0.5734648926902947	0.5592728873740319
0.4481185398434138	0.5937804339433368	0.5641466397875550
0.5217629974337689	0.4272507830746504	0.5635960229950131
0.5773147617391435	0.4265354970382694	0.5582639676312051
0.4773238086930306	0.5726187902572232	0.5647137660129319
0.5099642714680290	0.5848224461490338	0.5676595867017544
0.4148579594939387	0.5113673420591925	0.5553173632551158
0.4890893363228815	0.4150004660877962	0.5661587644710928
0.5841958839579007	0.4886922334784401	0.5552934643826377
0.5152925828509721	0.6208849750067176	0.5734258132665772
0.5066242725062562	0.6450118513996393	0.5507484899032167
0.5129374283149098	0.6784671010593665	0.5563779482264969
0.5273299992829402	0.6887058082499892	0.5852542169927836
0.5354629349895843	0.6647754488782456	0.6082237761797938
0.5300117943228401	0.6313670564383747	0.6021905302306915
0.6204169961809486	0.4848011798768971	0.5494232363693290
0.6416660847755444	0.4698477638718739	0.5717264029398582
0.6755579218631070	0.4673134234397723	0.5657653181606452
0.6889580854762470	0.4789967067660389	0.5369502385869458
0.6677546018549502	0.4934869403118449	0.5143945299335504
0.6341381256198215	0.4967937935112470	0.5207498247628208
0.4837558879956579	0.3788250595279620	0.5712803223475221
0.4689032639227444	0.3677653993848313	0.5997814496994153
0.4635965276729163	0.3342266377227679	0.6052559327855181
0.4720094720476904	0.3107251458501770	0.5819643610844841
0.4864653372522684	0.3215448730441330	0.5533135743046831
0.4926306513280481	0.3551271380862992	0.5482557643485061
0.3785821508806775	0.5155174613171903	0.5498507774754960
0.3643534033954421	0.5041602844981565	0.5211580623710714
0.3306897093084393	0.5079946531107534	0.5152458650261150
0.3099518059563959	0.5223463674255048	0.5382811969172702
0.3238217833564717	0.5332956857452975	0.5671528185384089
0.3577593209014713	0.5302808979733628	0.5726491591901635
0.1483566382076474	0.5279541725152860	0.5532209863632086
0.7247042420836330	0.4752010223764234	0.5280881441933297
0.7816644148297676	0.4763958075797561	0.5514678240755345
0.7981293359337762	0.4713805467565715	0.5806744841709991
0.8324642213758281	0.4683740880637238	0.5814464899611527
0.8508695871948518	0.4702821400541365	0.5531977124143364
0.8344691366529080	0.4754812575513782	0.5242293777909713
0.8000856149516691	0.4786184781744224	0.5230665872176902
0.4664676004641826	0.2749111532966668	0.5895702422532690
0.4487187676568985	0.2214569556691092	0.5660246961892497
0.4213567479930007	0.2094027004069250	0.5489764148894988
0.4140493789466979	0.1756783160590355	0.5493720105628385
0.4688462800310983	0.1994491196242545	0.5831777285951157
0.4613666059154612	0.1657438501660752	0.5832318796679605
0.4339553785174048	0.1536726676725001	0.5665262382876022
0.2740667606430440	0.5267131996622660	0.5302985929795901
0.2176238408977829	0.5237771165008186	0.5536467764777820
0.2018506499628906	0.5486760485280094	0.5359341910226870
0.1638273870428114	0.4863020414773143	0.6582719465674668
0.2051459193691082	0.4420686662973168	0.6902601467248946
0.1710346225634885	0.4368064142662805	0.6898106491319277
0.4920723352030976	0.7259798617370502	0.7144292754810648
0.4995342595862310	0.7822264891961657	0.6918167919223149
0.4762259590095922	0.7997557125691090	0.7100225630688481
0.4774046328073910	0.8342566952804898	0.7108219867183769
0.5236467755666052	0.7995290996596582	0.6743895778966184
0.5246394952182764	0.8340043099351492	0.6755343316863632
0.5016170321546225	0.8515596339056589	0.6937994482404337
0.5704225172451800	0.4711920686745489	0.6795939856197948
0.5667843948795653	0.4156810682554600	0.6841321530543421

0.4724594769533335	0.4276885166096540	0.6859030487594278
0.4312501616620207	0.4651173946592850	0.6796007651241730
0.4167182257252290	0.4327443649265032	0.6803109720397920
0.4416925281940540	0.4100573674492925	0.6843553845836623
0.5683016102738332	0.5331521168353566	0.6798952350498646
0.5271003705503692	0.5704282889243362	0.6871115562974195
0.5578568577338843	0.5880891426403076	0.6858054308626527
0.5828226289318579	0.5655073930518613	0.6812401887155113
0.4290780615465933	0.5270556032905372	0.6808332392767369
0.4103030050129181	0.5578277477569524	0.6821165935679451
0.4327296734047827	0.5823995009365260	0.6868890173393764
0.5343592820173813	0.4314574116366081	0.6853095964532645
0.5891485289808069	0.4403578194930309	0.6796483167953991
0.4651915084262145	0.5666469833495286	0.6870585936919562
0.4952369240346756	0.5843808322047931	0.6896137617189901
0.4143033439695955	0.4955174554474472	0.6776550323296644
0.5043352586612897	0.4136914187869352	0.6878755970516408
0.5852285016347295	0.5027943397725788	0.6771520303854303
0.4941321944605824	0.6208777962523676	0.6950102142755146
0.4807603616946977	0.6427634430015059	0.6723439573636710
0.4807765613374736	0.6768673521153949	0.6776882229051485
0.4934107649986425	0.6898694019175861	0.7062834611817213
0.5063624581500616	0.6680899995267504	0.7291969061464671
0.5071917918900412	0.6341989875318786	0.7234304302354547
0.6215911680427650	0.5051835857024766	0.6712994163988242
0.6450118323532745	0.4936580588272295	0.6934864379016970
0.6788575747291278	0.4967845869413569	0.6875897516516827
0.6902159769282672	0.5109223831141048	0.6589735872645713
0.6669285668745413	0.5220194057681350	0.6365367727096732
0.6332037634635480	0.5196338961154153	0.6428110060512451
0.5054742231773176	0.3771067408647595	0.6926413102505881
0.4928558479064693	0.3633263395070825	0.7210462166718995
0.4938503248831354	0.3293573444668920	0.7263094307149682
0.5065261449608172	0.3079760527245503	0.7029078155340677
0.5186285737574228	0.3214280485536017	0.6742956694802196
0.5184744860755205	0.3556039471648996	0.6694417379530669
0.3779202595407614	0.4932200655755994	0.6718529132286406
0.3663093921993031	0.4801419627094475	0.6427517521603995
0.3325731629520904	0.4777207263859754	0.6365263569515458
0.3093570519296355	0.4873797322817519	0.6596300074458623
0.3206970484764746	0.5001246426944531	0.6888567384258063
0.3545475863572901	0.5033588132741469	0.6946957843760295
0.1501952645952159	0.4588792980883220	0.6738003700401950
0.7261425088593453	0.5132730305913538	0.6502899927394415
0.7819944222396481	0.5229653937535484	0.6743311127574012
0.7990279797307145	0.5185411113855356	0.7033264980113192
0.8333760707788990	0.5213338686903878	0.7043971678946873
0.8512479452238806	0.5284580228992993	0.6766089169671621
0.8342732468196153	0.5329275388868082	0.6478342419079708
0.7998529483350545	0.5303207896520908	0.6464190657039200
0.5082586853059462	0.2717940642907242	0.7105905580448665
0.5020113234535974	0.2157679123719121	0.6872696469496837
0.4781220441000566	0.1980345551166749	0.6699776969698501
0.4781356928708228	0.1635295049363873	0.6706842448876157
0.5260777363134296	0.1986783052985540	0.7049370493219913
0.5258966025820049	0.1641594627737145	0.7053179312532992
0.5019389748833024	0.1464033464974294	0.6883891233629591
0.2733533341689417	0.4845618912331083	0.6515322320971370
0.2187394601675715	0.4695867030975054	0.6745743622065686
0.1979164255024009	0.4917777480610358	0.6584767277163112
0.1706459734293429	0.4314958200215848	0.7824724380514501
0.2159286839926274	0.4113122237778604	0.8285980697823704
0.1825126776048227	0.4037954163984991	0.8322454806768957
0.4542236929516473	0.7190513641811520	0.8441427259070697
0.4590580540386162	0.7770802191052922	0.8240444400838565
0.4439218482237107	0.7932472878946002	0.8499947714611025
0.4444451794518154	0.8277606575593579	0.8514443601048809
0.4742442537813701	0.7959412594341623	0.7998652668918512
0.4745720311582132	0.8303491330391352	0.8017914063066084
0.4597313714816051	0.8465410573886089	0.8276246381073592
0.5755769387746976	0.4842376656287694	0.8042053064112504
0.5822413193231650	0.4289597100820056	0.8086142390996295
0.4875938701857354	0.4230904900708624	0.8121483717782013
0.4398966719501040	0.4515737138351245	0.8051131135102538
0.4318904926900665	0.4170600387679297	0.8072002133434795
0.4607988314297063	0.3998038294481343	0.8118167252895676
0.5617940436273273	0.5446503786516599	0.8049402528823726

0.5141932459907461	0.5730617244726995	0.8128259699919488
0.5409855965701681	0.5963544977894605	0.8123357397997300
0.5698320015710676	0.5791498480711559	0.8071907038554906
0.4261175619540296	0.5119986856410365	0.8051842083148184
0.4020583544692648	0.5389305599285704	0.8052594977497919
0.4195013174769903	0.5672273305160271	0.8102809633735198
0.5475821773751111	0.4386227702800382	0.8107966532939674
0.5996292334929023	0.4573099575389541	0.8036681371173309
0.4541904463317571	0.5575605664250599	0.8118913103032793
0.4802715867530262	0.5805415422373774	0.8152747724499542
0.4175275801742702	0.4781652504032451	0.8025065191639309
0.5215381514490732	0.4156113451771479	0.8142038338329098
0.5841153697645985	0.5180835268168655	0.8017417434308544
0.4721642906417255	0.6160642349331688	0.8216963279914463
0.4611109832837157	0.6374385799470348	0.7975333256388141
0.4555870651988906	0.6709621333870285	0.8037914568208147
0.4601536490149002	0.6836302340469779	0.8346980024583395
0.4700693107791146	0.6620130285155588	0.8591611674798052
0.4764960184070723	0.6288700101377194	0.8526664592948301
0.6192743188675224	0.5270713075981512	0.7951546732639183
0.6448014547768588	0.5207051596764644	0.8171231178143629
0.6774917768610669	0.5290709201095517	0.8100448523240080
0.6856317498387171	0.5436078756856837	0.7805590808342501
0.6602654368965772	0.5497635809808383	0.7584724126981063
0.6275854521045193	0.5420420292257063	0.7658662017660102
0.5296590421865268	0.3800063340188854	0.8201832279451396
0.5261023796962782	0.3669763716565188	0.8511551208418758
0.5321908944277863	0.3336686188382491	0.8571597547019145
0.5410316500259411	0.3121508109452452	0.8322370714989271
0.5449755471898055	0.3250797183785624	0.8013480668758430
0.5397414595063932	0.3587314991415433	0.7955430057989137
0.3822009531199396	0.4693663570999053	0.7966151883159375
0.3730127445509265	0.4543977390745599	0.7675573318895281
0.3400381605766555	0.4472795842856792	0.7608066226877643
0.3152358061600418	0.4541022597111768	0.7833072804588459
0.3242893781223270	0.4684913273677520	0.8126240571777370
0.3572679664441215	0.4761640161069952	0.8190911857132241
0.1596246463896878	0.4138283295400907	0.8091328235493426
0.7203431804450677	0.5523745729232662	0.7711742741514382
0.7721543345535141	0.5760382605547332	0.7962220082738373
0.7855435128577521	0.5843595418724241	0.8262113638296565
0.8177843404567521	0.5962395421599845	0.8286095424602088
0.8371867113102777	0.5999638143227342	0.8011582173800417
0.8238159914562931	0.5917472462049145	0.7714070944089555
0.7914945812817344	0.5798810141135825	0.7685855661264157
0.5464198402741333	0.2765071795403471	0.8411857806389198
0.5408233135050396	0.2188791118030995	0.8201391328511768
0.5258875060375722	0.2006745568925760	0.7953510746929806
0.5252839692856683	0.1662360006544888	0.7965432632385796
0.5554062632911621	0.2020324568137546	0.8459824662847665
0.5546315592686420	0.1674971921339251	0.8466800788136315
0.5396157579835870	0.1493612799618692	0.8222398734193350
0.2799764808365874	0.4468470539513574	0.7746921704862213
0.2269605896206462	0.4290494022870771	0.8017048228856772
0.2040619772960044	0.4392190308635043	0.7784745046341061
0.4532684934446296	0.6247398318299275	0.1896057967936562
0.4022536728940567	0.5965544867579254	0.2271014371612022
0.4859464342703421	0.3730984548695349	0.1955329043272361
0.3731069961478730	0.5202506706953344	0.1839526336706443
0.3808725931012893	0.4547357123781755	0.1925796874570989
0.5182220567145759	0.6332362776842549	0.2001140008401003
0.6236555939165010	0.5517839592815135	0.1959143706041565
0.6317179497565097	0.4864438091407498	0.1866045847541829
0.5667925151991478	0.6243643753153154	0.1596500529481575
0.6018972372722716	0.6727588061976890	0.1713964432567599
0.4759858452457258	0.4995605721368442	0.1914286052139826
0.6272719912933840	0.6308179539814538	0.2630096671200311
0.5921458971451566	0.5818307126109820	0.2508614083115763
0.5967401134067795	0.4053925366864491	0.2261884150324434
0.6458269766852882	0.3704505115967027	0.2174310705242337
0.5286449883016687	0.5069687712548537	0.1915323415462186
0.6565368598898835	0.4102883313256933	0.1224952195425173
0.6075925110790242	0.4468057743476014	0.1318407758854940
0.4122574942353806	0.4239200755385146	0.2483523981478163
0.3758225384790884	0.3755621570459372	0.2592500884586184
0.3536973432730116	0.5993924780651159	0.1179889701130398
0.4008897563281159	0.3350486185490291	0.1669626893783624

0.4371922332170338	0.3828191861371711	0.1564132303508577
0.4034608078323978	0.5644169258218908	0.1288931427273222
0.1774698586204506	0.6974112384949539	0.1759689181622847
0.2689620532259837	0.2056343599579002	0.1824071939932149
0.6978347462865448	0.3657517568030644	0.2083983965954504
0.7334699000374952	0.3356414059431505	0.2374782870302106
0.7786732434992981	0.2944588841696648	0.2406662002369986
0.7998646885105452	0.2672845166753137	0.1908703560581038
0.7749003029475500	0.2822979931374301	0.1382497837000987
0.7295514784207442	0.3236786120993935	0.1351039684658836
0.3494144344681841	0.3232476112103057	0.1753744424199491
0.3081157771070280	0.2981993502605357	0.1478221302090300
0.2715550833376515	0.2496402085650011	0.1404711699459497
0.3402086317416995	0.2596594532239693	0.2389763640919620
0.3037521376937147	0.2110603407951893	0.2314886658379483
0.3002214715817164	0.6295003729161657	0.2029746570409678
0.2732693537640669	0.6800427116486796	0.1331702997091287
0.2178282464278580	0.7064317055759277	0.1316269768947970
0.2493758439246520	0.6362872761478245	0.2243938551978155
0.1937960422816118	0.6619979020412917	0.2224923105226898
0.6536364416615155	0.6853439586539986	0.1808370470456851
0.6589391770768391	0.7484772831224478	0.2452810499789877
0.6944755305796819	0.7980076655437538	0.2395491575444579
0.6951369829982067	0.7118644500160497	0.1548104502115057
0.7307463364796536	0.7613738393047816	0.1492136766457420
0.7308031194290319	0.8048866532855267	0.1916997784346982
0.5509742089270527	0.3817049986476651	0.1860979650647544
0.3519928762473981	0.6294422297619515	0.2168139331114164
0.4315543946120856	0.6125660702526310	0.3131553947443353
0.3918146018086791	0.5827171289453823	0.3522285744515176
0.5078674889597042	0.3701687506995160	0.3186650253088834
0.3705985107759056	0.4948066867361217	0.3125891967844418
0.3904135640484128	0.4318009314091075	0.3200114175004367
0.4941489989512965	0.6322001785989484	0.3223976766973596
0.6116869570298944	0.5705526526798010	0.3217400682820472
0.6315346635962227	0.5077556245236617	0.3128664628020119
0.5444262585309795	0.6315400270642892	0.2834734952466335
0.5736265054149082	0.6841968972410924	0.2941891147263769
0.4757062343270033	0.4932330956395458	0.3163028177987260
0.6022666730019542	0.6482058161365926	0.3871470242983675
0.5744396056835547	0.5945430044688749	0.3756342385876575
0.6096846855458052	0.4183202937607035	0.3498521663726537
0.6641561078806497	0.3923366746152115	0.3408296390462289
0.5264104045537159	0.5092606203543449	0.3162180695714026
0.6710007455339840	0.4403400786955373	0.2490660117431002
0.6168867994309618	0.4679805038314303	0.2589416474125125
0.4272176041510130	0.4069752284745021	0.3733121088347749
0.3983722502633358	0.3535688428372866	0.3834344294978422
0.3320259555534141	0.5645871353863691	0.2498470275894198
0.4274765370118546	0.3191053876197100	0.2900809997575457
0.4576574645680385	0.3714788898165092	0.2807327426091122
0.3858176948232417	0.5362951067531437	0.2596453269266346
0.1599742003903213	0.6684017000330904	0.3083557110397163
0.3019962758405500	0.1788725888217760	0.3210257743414264
0.7180734817482067	0.4031705106948428	0.3331711746245787
0.7531189032759109	0.3681283241954222	0.3617393128495305
0.8071306665077558	0.3391531335225473	0.3641642394006142
0.8409611946142104	0.3320140191651780	0.3145326412944723
0.8200207046277724	0.3548412570758067	0.2628597816763154
0.7660322426461263	0.3837676845581555	0.2605188425581654
0.3726736940898240	0.3019198684799725	0.2998220205717551
0.3171802329832200	0.2805549180940603	0.2929061818123536
0.2824493784136910	0.2298099731295221	0.2928687890430605
0.3907260658628907	0.2304029601818807	0.3496936889650381
0.3564428253592146	0.1795728263838262	0.3492107665697864
0.2815944349394927	0.5961819007109491	0.3332516105469621
0.2627749685323401	0.6655740647697223	0.2805261750138364
0.2079679179829787	0.6930029812133667	0.2792041185271172
0.2230536975609980	0.5892004749522389	0.3414190587949473
0.1678571346127430	0.6161901006810775	0.3394910515108920
0.6272484543965328	0.7030774981011992	0.3047010564299219
0.6054640975665682	0.7723451460429510	0.3556268626136590
0.6375694534694049	0.8245643686973771	0.3571508128034355
0.6826139959319798	0.7266428622901623	0.2998131600332120
0.7151526784695311	0.7788121619873284	0.3017430219156527
0.6926825821723652	0.8281927472884080	0.3304308074760262
0.5703969410883952	0.3900378741052279	0.3095598241499437

0.3374893082879687	0.6093592934112314	0.3431715973732025
0.4115420685094374	0.5983972210274894	0.4347011112529581
0.3767894530041146	0.5624028293064161	0.4734877090071495
0.5280636201946314	0.3725756421528025	0.4436638734275796
0.3715299313969013	0.4719614537534627	0.4344398049029224
0.4016000189279946	0.4132704158832750	0.4425645038103487
0.4697529079249127	0.6285516421973764	0.4441848426975554
0.5961902682241960	0.5877674961553655	0.4435954865346740
0.6262694231286481	0.5291490492945621	0.4350750602069037
0.5188492179991935	0.6369076125850593	0.4061371901703821
0.5395732088107037	0.6934486955323683	0.4165071422493060
0.4753554693039459	0.4881601985861330	0.4393388229558115
0.5759556800254060	0.6622532535110804	0.5085226463189655
0.5566456153754505	0.6049186618420394	0.4973839933848849
0.6209837958258034	0.4383668208858493	0.4733718291285812
0.6789958619030170	0.4220854092045499	0.4635800468027386
0.5224137022665696	0.5129543800356836	0.4393998468608999
0.6754615458896021	0.4685390171824737	0.3708985169757919
0.6175892535655024	0.4865349967770554	0.3815204267289831
0.4408747772742194	0.3948427955606378	0.4961919899093354
0.4221841742025828	0.3370236746806368	0.5060455710108127
0.3223797665465372	0.5328426155854048	0.3707872059251805
0.4594851772008975	0.3082881569084592	0.4136125578075624
0.4796152862143930	0.3652814178667022	0.4045632471910706
0.3803474580542485	0.5150048284662180	0.3812768361101787
0.1317726569591117	0.5952061387151431	0.4305781593201241
0.3688139237814810	0.1440453479203162	0.4429572729937354
0.7302450823405610	0.4436581943548875	0.4541044822041972
0.7721533783009085	0.4193563238259819	0.4835034905366405
0.8306666245883647	0.4012024871788007	0.4859791395223425
0.8638161446265948	0.3957941081783299	0.4356985234121620
0.8374078414584433	0.4092009265936423	0.3832877076471360
0.7788837016528348	0.4271829495560318	0.3808876122387858
0.4102795985858196	0.2797511417535418	0.4220519710323408
0.3641649800442363	0.2452407420391247	0.4100699717887372
0.3417508887826960	0.1880315487065763	0.4100146250940525
0.4399489798817114	0.2155732481354226	0.4767633541694952
0.4182836215196701	0.1583036385633650	0.4759776900693062
0.2670375778344407	0.5534329112580832	0.4539465104346271
0.2319215731276079	0.6162948206519769	0.4011192504161659
0.1722588368868862	0.6301886500482951	0.4006930260228159
0.2121230573226993	0.5332538795235411	0.4629628138909790
0.1521494440693325	0.5465295119047192	0.4617834048810502
0.5894188530803789	0.7211365381629425	0.4258919768105897
0.5599126986109932	0.7846411164787978	0.4812365087966030
0.5827823823353240	0.8414527660305792	0.4822059156527223
0.6369295777081329	0.7547952585296436	0.4159759561749597
0.6605171415833209	0.8115076695903816	0.4176522208566081
0.6334776803783935	0.8553350536470834	0.4508017123123162
0.5863022972828660	0.4027648089783012	0.4345342278128193
0.3185738437912339	0.5784519989578777	0.4638344660096992
0.3959735396435060	0.5804931480855536	0.5569026614080681
0.3682383473897063	0.5390730182732634	0.5951562676994572
0.5512613115608227	0.3793364123245903	0.5648928692050659
0.3794240701401417	0.4488925637848562	0.5564005967281547
0.4196513085998735	0.3966348722922555	0.5645108755422484
0.4478529060496058	0.6205174068673421	0.5667584026266336
0.5793997029508595	0.6032314510430813	0.5663495778613986
0.6196217013011526	0.5511258814876292	0.5573639768066649
0.4953354661057663	0.6373870253287124	0.5282068550903743
0.5063213871189144	0.6967017492692843	0.5381888091488851
0.4785987057766005	0.4836393657098157	0.5608338528075532
0.5460775418447940	0.6728178691113051	0.6309297546553714
0.5367972950913106	0.6129352993361675	0.6201332271055343
0.6315692187048493	0.4605463267794416	0.5942046569696255
0.6914904264086573	0.4557599643094666	0.5836729286296041
0.5204544025579926	0.5162910409368209	0.5613549203050996
0.6780783977414790	0.5019620928811150	0.4917113441322886
0.6179464080635980	0.5083843261014886	0.5030888581379230
0.4619417752832284	0.3858470616209305	0.6179953224174299
0.4529303102506360	0.3257440255464843	0.6277914922427187
0.3200122777065029	0.4999986301418341	0.4925515263152047
0.4932952184732497	0.3036540481747556	0.5348800889897181
0.5039990943309171	0.3631917797237375	0.5258957859088170
0.3801762565728893	0.4926918667954226	0.5031111227703716
0.1213902179263187	0.5295651590877858	0.5529494175049550
0.4282090861196170	0.1272774924876937	0.5668073090665550

0.7374276757223948	0.4863876855106404	0.5743568345232645
0.7837873959305157	0.4702585000022768	0.6027748608054933
0.8448268819771800	0.4645599429958738	0.6043727837510560
0.8777628901545350	0.4678043835006523	0.5537897056147053
0.8485146076564449	0.4771524318514236	0.5019732264102517
0.7874950336660861	0.4826290480729811	0.5004269588957481
0.4482340945102517	0.2675529105807966	0.5440361379375054
0.4061315497992518	0.2265879502507254	0.5352216535993570
0.3926039468813049	0.1666574221009632	0.5360988050613181
0.4901806132559380	0.2086932228285379	0.5963233904903555
0.4773202955359582	0.1487999796169399	0.5964948040111475
0.2618163668174192	0.5105623936550645	0.5755106638414400
0.2164937502140289	0.5663400819306523	0.5223048601136614
0.1553577996354121	0.5702052310794063	0.5221973895466747
0.2110684722159016	0.4821792375762400	0.5854731823341744
0.1497168237846811	0.4853669166194549	0.5846000101792578
0.5514662727294587	0.7320985175934003	0.5478428537062923
0.5097854585032620	0.7906852253963452	0.6003596109836585
0.5234005981970871	0.8504227638786921	0.6015990880062414
0.5945687638419841	0.7726519903903993	0.5402628198243161
0.6088494674726430	0.8323912710148945	0.5422246935709538
0.5732741647199373	0.8717368825554007	0.5729848046930559
0.6031498494097604	0.4195696399428698	0.5558317567163260
0.3081484169661666	0.5446279676322182	0.5854030222369886
0.3836198839038433	0.5602546564043372	0.6800642227878504
0.3630412030336714	0.5134554355319955	0.7174743437173633
0.5716994586911250	0.3893436404142506	0.6861221419391927
0.3904313944593098	0.4277298055894523	0.6781213695628613
0.4393006935617916	0.3833634100424014	0.6859531818745667
0.4278287777920791	0.6086561303523637	0.6897447077108639
0.5602672247810131	0.6147403344046880	0.6879269369375447
0.6090980551265606	0.5705981056384748	0.6790856300817382
0.4706832981132972	0.6329575690031536	0.6500551231972750
0.4705124734276662	0.6933823051654922	0.6595431161607527
0.4818020124836371	0.4795364339764999	0.6832640387653311
0.5155897736949437	0.6781559183453489	0.7517165740858308
0.5176006849838979	0.6175376752191098	0.7413758814737419
0.6365551189153528	0.4824822557670561	0.7158034452480146
0.6964359828551988	0.4876453803903584	0.7053621040929708
0.5177706932445593	0.5186355504164334	0.6834573491471313
0.6757277098758377	0.5324065847594022	0.6139975545869975
0.6153555265105837	0.5285755053557911	0.6252019781566894
0.4826816236750951	0.3796904353632815	0.7393721814885261
0.4850027422510297	0.3189057386351341	0.7488056286211516
0.3237527175442740	0.4683139488544124	0.6135948631726615
0.5286451507763910	0.3052156722728914	0.6557707349914724
0.5281409052855052	0.3657851793685455	0.6471417408976666
0.3841614508582604	0.4722186074898518	0.6246972703886511
0.1235028720199778	0.4547437462999027	0.6734212921312170
0.5018786168369869	0.1193948223822755	0.6889422152567709
0.7365221486902329	0.5252628439861925	0.6969023668033724
0.7850531541985846	0.5130735965269263	0.7250420524209682
0.8461929136796301	0.5178447451120174	0.7271403219530700
0.8781662946264915	0.5305466960758258	0.6774123013171148
0.8478863668439569	0.5386040245270599	0.6259503485568713
0.7868106217168591	0.5338502859138430	0.6239366345644335
0.4915867777324137	0.2606692648422319	0.6651956725449282
0.4597609197174534	0.2114781365151867	0.6559018063512544
0.4592618785945650	0.1500320757273498	0.6573118866693539
0.5447368025076246	0.2123697779588659	0.7182601431012782
0.5448135272196195	0.1510873756705418	0.7190371441692386
0.2646385536346843	0.4662605847503740	0.6967238960522474
0.2083973681319634	0.5131589744432522	0.6462866794999461
0.1478051101906946	0.5038379568666714	0.6458325789544231
0.2215117325095486	0.4249105170281289	0.7027809222371748
0.1607836736728884	0.4151804552058935	0.7019427185714415
0.5087710469661205	0.7374266885671975	0.6691363880402904
0.4573650529404324	0.7864327628647562	0.7234215230346265
0.4590762458457705	0.8476705980407747	0.7249689606391880
0.5413690005404740	0.7857794128857030	0.6598316039142266
0.5436806975458435	0.8471319825919997	0.6620365115328197
0.5024932573910814	0.8785409343013183	0.6947267119059606
0.6157713415316876	0.4379364922713437	0.6769844644693995
0.3030371713133589	0.5079355673610739	0.7071373478757906
0.3754805173073025	0.5365356528892977	0.8021382269605483
0.3638639710022552	0.4877449842133502	0.8418036211784867
0.5917151160138534	0.4038466845695095	0.8101688939885399

0.4070098509550559	0.4070334686212656	0.8058588562902358
0.4636913469984515	0.3732640640833765	0.8146938842251410
0.4100306477107415	0.5923079647980197	0.8122520752209457
0.5381184088032024	0.6228683489730250	0.8154486584284490
0.5946847951060761	0.5892012655104248	0.8055606655472188
0.4572694644366821	0.6278329359904373	0.7734200265765213
0.4474733477800542	0.6870328360776744	0.7842829738035926
0.4865244248400400	0.4757503904427264	0.8078789607149204
0.4726924651635312	0.6719293935675336	0.8833078519084598
0.4848677304983693	0.6124628139120382	0.8717855514201324
0.6388901466295163	0.5090516095556453	0.8399786190552058
0.6968365293574459	0.5231648338673807	0.8274227224125665
0.5151930021676943	0.5204499422915483	0.8080653781249237
0.6665941705819447	0.5606192625189621	0.7353728303822122
0.6080420429208776	0.5471074588508923	0.7484963963954080
0.5185094530721230	0.3833045864042948	0.8706390079266875
0.5300567221997633	0.3235157847804599	0.8812576642400924
0.3330504465396247	0.4365137570939540	0.7378411128598431
0.5523170840737823	0.3091287023706184	0.7814646647541337
0.5429930016645094	0.3685116012093358	0.7714113116648660
0.3920921073631033	0.4489789006360580	0.7498068058847812
0.1334255016012229	0.4079565824194442	0.8119450180154912
0.5390357951969145	0.1223714472184801	0.8232009715161753
0.7270716321167658	0.5668210221587281	0.8170730544125981
0.7704914558669490	0.5814195414588679	0.8477749474573313
0.8277774956144019	0.6025780868953112	0.8520818560759744
0.8624710415544522	0.6092578341666930	0.8029865329578787
0.8386417469587536	0.5946204791129450	0.7497479784692426
0.7811820937512983	0.5736770837880028	0.7453414134926553
0.5310563676641550	0.2623684115433770	0.7963048965583680
0.5147859499601569	0.2137804496059977	0.7751495432139317
0.5133690475204722	0.1525741356841568	0.7771720292814001
0.5667696513440705	0.2160761908798249	0.8652363086914010
0.5659857663054551	0.1546689221317398	0.8668883712822915
0.2738687987546605	0.4316898728523478	0.8205126122136374
0.2124269097249743	0.4529432856832716	0.7576246598115660
0.1530256005116396	0.4396548626246286	0.7643101626657683
0.2337707742671673	0.4033451870459515	0.8467073907630882
0.1743662592479694	0.3899931883921937	0.8532640280744894
0.4693507939701513	0.7341244450620602	0.7994565909221846
0.4323573864867072	0.7787047555889662	0.8687868411691245
0.4326746604914965	0.8400565719023843	0.8717415159667304
0.4857752386749850	0.7833594740659250	0.7795806228939942
0.4867315779133820	0.8445303337579160	0.7829194740299305
0.4601345042455143	0.8735048608688801	0.8291695576867368
0.6261619993383560	0.4597661943314495	0.8002305954891711
0.3053889825891712	0.4748893566839806	0.8302815020148454
0.2959746541728562	0.6407443077223062	0.1814601698849172
0.6545107017840641	0.6985169543816923	0.2015705048896403
0.5084399316790481	0.4532578826117854	0.1907355923435444
0.4508658349086436	0.4966052030503889	0.1908352194005856
0.5537711187915582	0.5099481751912079	0.1915489649807788
0.4961943085899438	0.5532837762518625	0.1915793260801407
0.7024051674509018	0.3554216255045038	0.1864943608618217
0.3474985351613669	0.3099263103242093	0.1959501127042204
0.2773707617709659	0.6094475973766298	0.3127201496910977
0.6240714644637804	0.7162115905616446	0.3254558301563307
0.5162070184325923	0.4530960100848585	0.3157063719068224
0.4515697517778958	0.4856419880148219	0.3162507982030991
0.5505487125292402	0.5168464823686115	0.3163920615703344
0.4858603913922748	0.5494039637387910	0.3169168244787740
0.7249648316339556	0.3938521306419857	0.3113362822605351
0.3753241422373952	0.2879583131457848	0.3201303551882461
0.2597302933086295	0.5652748088112710	0.4334107661265619
0.5845089823960792	0.7334241869186643	0.4468474045962160
0.5219877789861094	0.4556168765191510	0.4392042661146991
0.4528131859502598	0.4766670947175135	0.4391944860540379
0.5449579019561214	0.5244447166698992	0.4394085170030732
0.4758431102798443	0.5455340062453369	0.4393947954474904
0.7381513783019883	0.4344013133915153	0.4325581315498208
0.4152021132183086	0.2670253014586844	0.4427421731891233
0.2525527394304149	0.5211074906598027	0.5550554703312743
0.5441954592920057	0.7436365262921109	0.5685591243421767
0.5303870703609991	0.4599471533096804	0.5608494858061160
0.4585345973805078	0.4682264002906404	0.5607209112513427
0.5405227585340499	0.5316989049504448	0.5615030394670770
0.4687053328920565	0.5399792055265623	0.5613852629130175

0.7468066356525581	0.4796780789706684	0.5524959534103854
0.4556808009791511	0.2558110166610853	0.5645852300436640
0.2534090518996772	0.4745564739130967	0.6762556997619021
0.4991071798496117	0.7472235967313389	0.6897717969052879
0.5371933594122659	0.4651761619431454	0.6828080842538476
0.4648434868543415	0.4607596414235246	0.6830576345068434
0.5347153847513448	0.5374260142412405	0.6835490678911100
0.4623539916020322	0.5330071107797150	0.6836939090499516
0.7470720842948436	0.5204599558526387	0.6750848407383238
0.5015213734450815	0.2507871621154765	0.6856686707031221
0.2609966225620389	0.4365216946038831	0.8001104115132603
0.4600134355688681	0.7423272475080930	0.8207534808241217
0.5441063706781645	0.4722764992183742	0.8081665139934739
0.4737039999589687	0.4539400162290071	0.8081740443213152
0.5280290588167107	0.5422496145280844	0.8084457651546224
0.4576319599469788	0.5239363510048234	0.8088455421439241
0.7391680046453798	0.5646408712595307	0.7958182736496041
0.5402062240077736	0.2536854474877726	0.8174907087422112
0.6898196532301983	0.3597405635315814	0.1327599205762806
0.3604079055792875	0.3130383440215097	0.2497048023254543
0.3150196268775428	0.6452838519324609	0.1295977975081476
0.6400569167556049	0.6945400766806958	0.2548886121218402
0.7095721063128801	0.3895452302308944	0.2582665047632397
0.3940566553470314	0.2890101676555710	0.3722741293240246
0.2940481368325395	0.6160500659610483	0.2602688200306789
0.6054263672168915	0.7129866892991986	0.3774945958922858
0.7223590708299856	0.4246567400467010	0.3802710146281390
0.4301935631721166	0.2731832996064388	0.4956882774481402
0.2745187076538427	0.5761928710670596	0.3811181615846909
0.5689826560849810	0.7263220198092742	0.4995268459730152
0.7327447914947205	0.4684708373348877	0.5000349375287714
0.4712203745036698	0.2640885731960154	0.6170900019828074
0.2651871926533826	0.5346124831868669	0.5027995001338631
0.5287261813894863	0.7348586124403784	0.6209982149897385
0.7353997322735546	0.5088459322137647	0.6221744812571777
0.5152610057819011	0.2623788364781586	0.7381070029351755
0.2629855989596200	0.4904892525485420	0.6240830712475829
0.4852027972719479	0.7350922742730254	0.7420702934598981
0.7304798074772503	0.5492017271440076	0.7432668938654873
0.5551020872680685	0.2690996248493090	0.8686676036622496
0.2693522448302568	0.4498344798707828	0.7469528423240398
0.4454800612118104	0.7258830259393583	0.8717455620536428

```
C-CENTERED HEPTAMER
   1.00000000000000
    40.4062059999999974    0.0000000000000000    0.0000000000000000
     0.0000000000000000   40.4090679999999978    0.0000000000000000
     0.0000000000000000    0.0000000000000000   46.8059700000000021
   C    H    N    O
   504   350    56    28
Direct
  0.2242384090314729  0.6897008748332815  0.1327715294074308
  0.2412903594384121  0.6505899404612839  0.1794909780066171
  0.2099445390419383  0.6648131820012971  0.1778683499681965
  0.6413377899832183  0.6796612758865587  0.2076212425187142
  0.6819305368456595  0.7194824440906481  0.1853789807368469
  0.6809147779580472  0.7449391614645237  0.2057282200978448
  0.7031387524526352  0.7712553833595547  0.2035263897675649
  0.7051766646669746  0.7208674651649460  0.1630743442218075
  0.7271806854955453  0.7472966919195470  0.1611496619032023
  0.7263310212329845  0.7726546749664577  0.1814394506228215
  0.5367299801560206  0.4356565654409217  0.1689137175055937
  0.4977729289818582  0.3961969866463682  0.1744125120774159
  0.4343159034221738  0.4670610459397032  0.1747499319972295
  0.4280134275667676  0.5220723642458884  0.1674885444084084
  0.3957544840561455  0.5072504564762632  0.1679525124485650
  0.3995627345870653  0.4739268013591085  0.1727251297724765
  0.5756930309802679  0.4838121963160451  0.1686310536567159
  0.5690760008847686  0.5388160186932330  0.1759396500895124
  0.6038752296870485  0.5320113535440278  0.1746117739042645
  0.6078916017736142  0.4986923934275808  0.1699234806376044
  0.4668698540477121  0.5702372586626908  0.1688671764633794
  0.4725655323117824  0.6057623282946965  0.1708325265530879
  0.5054426004257615  0.6096821193839619  0.1760383631099058
  0.4835576220775849  0.4293594404776390  0.1749629960012163
  0.5307569684973666  0.4001117170649372  0.1697847670231686
  0.5198483326899936  0.5765741089938110  0.1759016887908449
  0.5540903981026319  0.5701182422841343  0.1787695307998273
  0.4351989535329748  0.5560636740239161  0.1653318613328572
  0.4492639289113752  0.4357525144205634  0.1774482309533309
  0.5685823506341925  0.4497902915328766  0.1662064997470104
  0.5768873367453772  0.5982083957879942  0.1851652680519512
  0.5821082141859706  0.6241912955382379  0.1657520254752146
  0.6032783517088777  0.6503183167499665  0.1724796609294716
  0.6195213393169662  0.6514104359887886  0.1990010707593967
  0.6143201745439070  0.6255755075399387  0.2184727184634220
  0.5936792016385698  0.5992114882317695  0.2114870149960904
  0.5976892235771813  0.4277937037116989  0.1610694111626769
  0.6090425786034751  0.4054134058974402  0.1817944957061268
  0.6371426441264991  0.3861070365976553  0.1769389941184484
  0.6542879242456648  0.3882297148588891  0.1509540245282878
  0.6425486092434144  0.4100431395334405  0.1299372956098675
  0.6149827541071827  0.4297952168162471  0.1350356799169413
  0.4263916029966987  0.4074663417520207  0.1829610820077174
  0.4094196922625321  0.4054322275766016  0.2091448625800470
  0.3887348049811907  0.3787911527675842  0.2152108690700236
  0.3836972857836142  0.3537345422511790  0.1949655513497975
  0.4001425380876451  0.3558824399605644  0.1685855318076859
  0.4213164425587037  0.3822862271710599  0.1627519692575186
  0.4061386144949301  0.5778954910580939  0.1593420214617028
  0.3920705132990835  0.5780194963091669  0.1318751391940712
  0.3641214316188925  0.5969212435740862  0.1259855246170509
  0.3487336826919559  0.6154688051259636  0.1475806120267477
  0.3627810501460928  0.6156396621364513  0.1749680489457223
  0.3914199136230598  0.5973906165582745  0.1806223715965326
  0.2011282213120982  0.6844240604939394  0.1544730509100943
  0.6835826817625776  0.3670784289017180  0.1431826997717137
  0.7290822062673501  0.3333674320874080  0.1648208534176402
  0.7429098609270361  0.3238856978342395  0.1911700467546122
  0.7692054176876314  0.3016436480170323  0.1921202099787512
  0.7821956093290185  0.2883950393648437  0.1669073183984688
  0.7684735775099867  0.2978536666513651  0.1407802892710464
  0.7421382186862663  0.3201679951331235  0.1393903789026228
  0.3617586174474184  0.3251847168023428  0.2026015779908797
  0.3217608752773907  0.2860186423096881  0.1787013277263883
  0.2989889666998073  0.2854169682373660  0.1560055714844749
  0.2770409320539631  0.2590711642312049  0.1530331219759000
  0.3223437332180228  0.2598483822082490  0.1983843577500627
  0.3001879961399898  0.2336138410703742  0.1951321714826108
  0.2774847806284641  0.2329941508301799  0.1726406756456597
```

0.3185051351415454	0.6348121319436465	0.1390776301004131
0.2644067308052646	0.6556898937318553	0.1574591409970957
0.2557418597854853	0.6755798107383015	0.1339587305781827
0.2068948450644829	0.6632174458979588	0.2585011871579900
0.2177696185031354	0.6043512547877583	0.2881704044093383
0.1861208596614705	0.6180519549289014	0.2870645849750181
0.6079214990438325	0.7002573093431302	0.3153627941600709
0.6447300146307288	0.7439113697190107	0.2960156803614474
0.6327044031498553	0.7720971753064896	0.3100401508497115
0.6521358459592291	0.8006107790099019	0.3112154083337887
0.6761649656689923	0.7445885688477422	0.2833116023090618
0.6954088912963527	0.7732025145460784	0.2846934978436635
0.6835059948482851	0.8013452284247771	0.2986634997801624
0.5470684687230022	0.4412102647951745	0.2811481070195276
0.5160225683396634	0.3951527417042464	0.2859986803854225
0.4407377803778073	0.4531770181601265	0.2873294434263543
0.4244062659942946	0.5062734480711729	0.2808665710896212
0.3954033204445631	0.4858160337004238	0.2815071786962812
0.4052958108740496	0.4536513703628377	0.2856922221676870
0.5766209327793070	0.4957418561022310	0.2808760367875824
0.5602367456589735	0.5487312668861030	0.2878623688493538
0.5956858145645815	0.5482839350961040	0.2864559650105106
0.6056212535354556	0.5161834248028775	0.2819703956287748
0.4538845184619328	0.5608159734746350	0.2819334110370545
0.4531893680521939	0.5968632010804045	0.2830304196577960
0.4848812826129545	0.6067622338789624	0.2876385348527717
0.4959890062372266	0.4251289099424922	0.2870182107925905
0.5477034430606312	0.4051331985178409	0.2814870990035026
0.5049591730566795	0.5767788612734657	0.2879744290829178
0.5398270249020402	0.5768521823438433	0.2904208395133411
0.4252242193253544	0.5410575561948820	0.2789320694992363
0.4611251051603680	0.4249978516478312	0.2895691367134410
0.5757755108418259	0.4609860499016962	0.2785085620250404
0.5572015894364201	0.6088152026908918	0.2959240894355622
0.5570963755371859	0.6348678856115070	0.2760660631799495
0.5739693528114076	0.6642982943952358	0.2816601922754157
0.5906753167495425	0.6687056146734913	0.3076487423619662
0.5904638324026142	0.6429746274667966	0.3277307720945134
0.5744467444034840	0.6132974337565693	0.3217444216350363
0.6082543524206904	0.4445291577134061	0.2732161354543496
0.6225445817121833	0.4228690015824706	0.2931716181935190
0.6532287955985571	0.4082303924755126	0.2879792907691842
0.6700747822694898	0.4142984554909911	0.2623212415636643
0.6556998951256255	0.4355455993082932	0.2421428537782664
0.6255180576077298	0.4508171272410695	0.2476839529488107
0.4435910606878042	0.3930308387819429	0.2946821377657002
0.4258196498673496	0.3884538503123817	0.3202313476259596
0.4091715823561686	0.3589935880123509	0.3257112344784319
0.4089335158559567	0.3335063698435562	0.3054002892579216
0.4263520217629187	0.3379116037225159	0.2797567290664879
0.4437520240632932	0.3671527597178434	0.2746452742021950
0.3927545689600851	0.5575181156013784	0.2735448455785146
0.3762593389378069	0.5521204098479658	0.2474847462024526
0.3460851288977204	0.5673278756335777	0.2417728505275961
0.3309502136942818	0.5875556939468560	0.2623053768173096
0.3469968086635858	0.5927073820462676	0.2884843264305990
0.3777199264778345	0.5781974815318475	0.2938483021028264
0.1804906057553472	0.6475141854563140	0.2722140192430738
0.7020224792967987	0.3977137937418249	0.2545221000779270
0.7554111242058488	0.3781708296160918	0.2753930297889004
0.7685510201377000	0.3662394176335205	0.3012007330610067
0.7994425496305054	0.3509277135776713	0.3016586396610374
0.8176308587943987	0.3472378359360334	0.2764589201725004
0.8046273586318373	0.3593283468903086	0.2508631539713913
0.7737706953330170	0.3748333064279779	0.2500858037564050
0.3907288714353335	0.3022886652537592	0.3124457255516815
0.3543953369173899	0.2592050893910708	0.2914316966747444
0.3241432486428745	0.2586141132520320	0.2767381073667723
0.3045889735068018	0.2301709992370425	0.2771431820131474
0.3649537866991298	0.2310993193722406	0.3064269696750497
0.3452195855165657	0.2027687426497202	0.3066123759028316
0.3150067599318515	0.2021163966465712	0.2920971880195529
0.2989527245193117	0.6040952535788539	0.2546608407686354
0.2441646336524399	0.6200493227816236	0.2741892310039252
0.2386452171833267	0.6496740688697441	0.2593172731622960
0.1790518309030530	0.6016102162289454	0.3649724099798997
0.2024821554381172	0.5483697080134029	0.3970442024035075

0.1686403801121658	0.5550363851909647	0.3959389113449647
0.5714177920226928	0.7154938509589449	0.4223030348647392
0.5977550120160120	0.7657699554457394	0.4022770236656198
0.5816286368322134	0.7905261511311977	0.4179869969572913
0.5948592540505947	0.8224022076513944	0.4187962603345659
0.6269675358443508	0.7732624377727756	0.3874405763366485
0.6400612676696985	0.8051661497911189	0.3885713547532299
0.6241153268657602	0.8298924756994221	0.4042535929148476
0.5555355122852316	0.4499426367450322	0.3897992258318879
0.5329290906269927	0.3992214620338306	0.3948318275278327
0.4486057308907664	0.4432354223923378	0.3955264122512123
0.4231038830750198	0.4925828814064893	0.3889429178495144
0.3981712231058862	0.4673383744617400	0.3899594775559614
0.4136205426715081	0.4374516846498662	0.3941712406878812
0.5749864885253891	0.5088244204690131	0.3892210054328081
0.5494201100557995	0.5581756914531663	0.3957386589485198
0.5844086385918215	0.5639729348652068	0.3943630283453162
0.5998893365543393	0.5340950270264488	0.3901625965094090
0.4425444693862825	0.5514888043889026	0.3895185514121461
0.4355838973366819	0.5868740356421153	0.3902678470672940
0.4650552863202491	0.6022043857002370	0.3948070845868208
0.5079594425787759	0.4252582817387220	0.3954170075024050
0.5624435757770581	0.4145389712524099	0.3904936249149030
0.4900695518142393	0.5762030160517534	0.3954434772135599
0.5243997235254568	0.5823673886749486	0.3980350155181793
0.4177785972286511	0.5269689462180623	0.3867371321610437
0.4736292936959785	0.4190704744678420	0.3978162953784298
0.5803456444425681	0.4744385074110922	0.3871292190983293
0.5360460665971477	0.6168680025797117	0.4034033105430274
0.5310941247444787	0.6425977495476605	0.3836830524883776
0.5430173983861909	0.6744080316204411	0.3890717497450432
0.5595383328397814	0.6814959850067119	0.4147064718785923
0.5640921287784041	0.6560415251380906	0.4346721339328631
0.5529661443001200	0.6241563462388352	0.4289171058761903
0.6152403863409429	0.4639488158068148	0.3818527321012986
0.6333575603653846	0.4454280067718317	0.4018539567089375
0.6660892687937037	0.4363392960505694	0.3964782903436829
0.6813400069516270	0.4448577764375571	0.3705873437693958
0.6632342364092756	0.4630188663603585	0.3504072064220508
0.6309158380873687	0.4728635280312925	0.3561412956263900
0.4619712283460197	0.3845085103454034	0.4028323844096636
0.4450655044993180	0.3768153887720218	0.4282606611389718
0.4339303054574983	0.3448395891063549	0.4336188219317744
0.4383976222661211	0.3197509295459793	0.4132974854858316
0.4548731458799740	0.3272519853622408	0.3877403681724657
0.4668745141200519	0.3591240901134858	0.3827747322702786
0.3828632182127939	0.5371880550364133	0.3812136670194051
0.3675010612011917	0.5276231026679404	0.3555306492396578
0.3349321625310486	0.5364434281781939	0.3496898599116719
0.3163195162950374	0.5542219593866563	0.3697625875336324
0.3312981747096152	0.5636156338362066	0.3955408724707806
0.3642871079329700	0.5555563225981874	0.4010001398226631
0.1567260178458011	0.5816372651251284	0.3798832356426626
0.7155996611398789	0.4339174233231168	0.3626394307717725
0.7718528532094395	0.4245951329441536	0.3833519786044473
0.7870747787712623	0.4166274807090060	0.4094495549374945
0.8201437090811585	0.4069281550797055	0.4101352184262282
0.8385123364915099	0.4050324596990494	0.3848828348632699
0.8234051319759618	0.4131753170007381	0.3589887802048209
0.7902989491096155	0.4229905375759998	0.3579655524440901
0.4264445217523737	0.2856556429812035	0.4204338550452140
0.3983657075083100	0.2364914837236309	0.3999171962095825
0.3691972158428992	0.2299824006433648	0.3846580034458746
0.3552167272703384	0.1984453895301228	0.3853006521076722
0.4135719997103677	0.2111003755616601	0.4155593249598590
0.3994157410696288	0.1796047793235789	0.4159048718962827
0.3701951105574325	0.1731027358788265	0.4009634411075567
0.2815429404742530	0.5634700643565532	0.3620709063274906
0.2247836076940927	0.5683079941298315	0.3818268766139879
0.2129431439054140	0.5951026569539331	0.3657546099113344
0.1675886408200420	0.5478850422969143	0.4738630097745370
0.1989499603211579	0.4980764560605034	0.5049965758569801
0.1644822133479666	0.4994242806070395	0.5038704355805734
0.5342583719451569	0.7251016700112981	0.5300486231086066
0.5533583260565786	0.7786478841828223	0.5102176688532533
0.5339136461320726	0.8007669129150010	0.5260651738020931
0.5425433367611644	0.8341750870100254	0.5270948360353152

0.5811992603946700	0.7903103839855333	0.4954136723499916
0.5896673390956763	0.8237285759854186	0.4967417892361764
0.5704540333014980	0.8458278659881207	0.5126162114480564
0.5642235797099791	0.4610096373791876	0.4971612205816396
0.5510777762933117	0.4070358980161812	0.5019659288183124
0.4603035684896129	0.4354286124214182	0.5028346579023797
0.4265525672105719	0.4795846913188903	0.4964043482697003
0.4064335234244407	0.4503622830938201	0.4974550567693593
0.4268710851292509	0.4236234775660910	0.5015668238875015
0.5728719912255152	0.5224414952721211	0.4970510783327319
0.5390664139689700	0.5664996565556776	0.5038022523488397
0.5724978135925115	0.5783356405741320	0.5026746009235776
0.5929669744728425	0.5516668545908711	0.4983325213508380
0.4351974650914607	0.5410334296729878	0.4970393417623305
0.4220784864625263	0.5746396949957568	0.4976591551830825
0.4483412088807621	0.5949498743296362	0.5023262857920702
0.5218511478782956	0.4281681355935413	0.5026724727481836
0.5773715140855803	0.4274174309775448	0.4976822527235457
0.4775328666927843	0.5737792125948572	0.5031897724484278
0.5101915318621247	0.5859227582840619	0.5060063486628519
0.4152047118052439	0.5124893035325588	0.4942265074775436
0.4891611529341032	0.4159868519010744	0.5050396136632508
0.5842300327429025	0.4895762264609177	0.4946669432250818
0.5156608783775609	0.6219276612802862	0.5113926276252945
0.5068278036738723	0.6463784527810621	0.4914723591172094
0.5133383612424751	0.6797423977747933	0.4967727745444169
0.5280944709835560	0.6895943314116483	0.5225298477692104
0.5364420743238341	0.6653477797861903	0.5427087834513650
0.5307846315074406	0.6320361657230459	0.5370315648504329
0.6204148255709279	0.4856002078458124	0.4892363121002325
0.6417111203047556	0.4704318393420709	0.5090002079078617
0.6755335658145609	0.4676146261644184	0.5034759033043712
0.6888165290632613	0.4791828654177190	0.4776591544991373
0.6675875066256793	0.4939616008844659	0.4576922885037250
0.6340451984961147	0.4975945064476136	0.4635870004429830
0.4837008635303119	0.3799003770893205	0.5099712370267365
0.4682332591931846	0.3693518711343158	0.5353218264745970
0.4626798301663289	0.3359374231725241	0.5405910159009726
0.4714807964806900	0.3120196919334707	0.5202671312789843
0.4865824430470796	0.3223021281988164	0.4947958554961069
0.4929804136947002	0.3557739717147007	0.4899087575149174
0.3790074266587491	0.5163774521771820	0.4887991819977427
0.3653481768643387	0.5041676037811621	0.4632299669275733
0.3316957484241546	0.5071946635815359	0.4575473404389386
0.3103981745523070	0.5216318659997567	0.4776419151277498
0.3237267880976926	0.5335621351617827	0.5033226954837321
0.3576458363574664	0.5313363147029487	0.5086356565513250
0.1486196321616047	0.5242904108104434	0.4882936095166865
0.7244451645317460	0.4748939901969186	0.4695257170431807
0.7816181488835614	0.4759867076006233	0.4897370925754813
0.7984276616963976	0.4698892247805334	0.5154794939227326
0.8327412932476401	0.4665189284254488	0.5156822167311741
0.8507689725630371	0.4691252623915663	0.4902949995950153
0.8340380163872481	0.4754255320795653	0.4647625827680829
0.7996812345086177	0.4789592425474494	0.4642393619871901
0.4656104183973803	0.2763701201848728	0.5274294173557577
0.4475265263823020	0.2227052419502923	0.5072525781837218
0.4196804302940019	0.2106140385579319	0.4927230199317510
0.4120200113041352	0.1769892878047400	0.4938123350262511
0.4677336754261223	0.2008348973081248	0.5226369284248134
0.4598979023151749	0.1672242926544448	0.5234275159404732
0.4320277694694648	0.1551189639745953	0.5091772682638783
0.2744922832188659	0.5248810640853495	0.4701466823503828
0.2178943820437807	0.5216350638252580	0.4902735045866274
0.2020797679447805	0.5466743802326335	0.4746679105112542
0.1640665103036689	0.4836378651841974	0.5817764465338015
0.2058565793802087	0.4424732900028320	0.6131524972309268
0.1719816316590847	0.4360221011853886	0.6121190622671004
0.4956950331831205	0.7277043763359278	0.6382238042230702
0.5058545424323623	0.7837612579967087	0.6185620682110978
0.4835828451598029	0.8023844375103729	0.6349814360333264
0.4867811403183206	0.8367463170818484	0.6360237511715308
0.5309559589551165	0.7998223091581180	0.6031820015478081
0.5339979838850845	0.8341521873901434	0.6045534063674579
0.5119985183543138	0.8527980405442052	0.6210216506634932
0.5700398494236235	0.4730750974204294	0.6039531881521764
0.5662160050616684	0.4176628717427751	0.6089000727982053

0.4719846209670118	0.4301584208486861	0.6104239383040225
0.4310708997830068	0.4677596936089123	0.6039628897633297
0.4163215159570071	0.4354999677130573	0.6050902003922624
0.4411016675835227	0.4127292569374640	0.6092333055593143
0.5681414972542395	0.5350551774841410	0.6039990826947076
0.5272160857191660	0.5725344260771299	0.6110164578536639
0.5581108558244120	0.5899835682610026	0.6100721000039154
0.5828833121597439	0.5672941038942982	0.6055764617266458
0.4291208758252004	0.5297369013239845	0.6045063770247991
0.4104755482023631	0.5606144295232706	0.6053107073601726
0.4329262381599658	0.5850854909144469	0.6099164377044854
0.5338614139998391	0.4335712663364789	0.6098556724713742
0.5886431965983382	0.4421689362252820	0.6043660089221153
0.4653052907186682	0.5691840894994222	0.6105643579249450
0.4954363788265002	0.5866751837526601	0.6133833927878055
0.4142483603398540	0.4982252506262059	0.6017976028174395
0.5037669179389668	0.4159876488263377	0.6125551904312925
0.5849508574026496	0.5046156394551801	0.6014478877899984
0.4946883480152755	0.6230252112412310	0.6190137111864304
0.4818421724883105	0.6457750180756177	0.5992287230483482
0.4826303902057917	0.6797147774335910	0.6047150487088417
0.4955179534036008	0.6917121596697989	0.6305338799131628
0.5078019577321955	0.6690693852042738	0.6505980576820684
0.5078476793656734	0.6353278853863873	0.6447366601630563
0.6212694859407146	0.5069581741713933	0.5959336148578737
0.6449740370091007	0.4951647967747506	0.6153780827118333
0.6787603639876844	0.4984619922839857	0.6098547755748649
0.6897764120959907	0.5129264472807928	0.5843297437663272
0.6662181081387186	0.5243094411528266	0.5646446027175672
0.6325713776006801	0.5218418152020539	0.5705642187300101
0.5046110859455234	0.3795100494269671	0.6176774873097909
0.4916690456342239	0.3666118163362964	0.6432707626920735
0.4919179903345306	0.3327442215116540	0.6486230880985572
0.5041508293833915	0.3105352035184220	0.6281717182635127
0.5168139645882867	0.3231288339571648	0.6024824153070392
0.5174077455835667	0.3571911837356547	0.5974988471712576
0.3778932474112359	0.4958608316935922	0.5964758901648564
0.3665306099988886	0.4817213854487119	0.5708204087647654
0.3328546331015526	0.4787847548451698	0.5652254169839130
0.3094245829690899	0.4889635264656339	0.5854911452436014
0.3205006004683828	0.5028445024471814	0.6112376365135374
0.3543032594458869	0.5066171039966099	0.6164628093201865
0.1509086798762334	0.4565169771167564	0.5964023003809277
0.7256080780003836	0.5151749002169759	0.5761924686251934
0.7817218055858197	0.5263260105987783	0.5964230719681305
0.7992445763133231	0.5239960980633890	0.6222779876730280
0.8335995759172214	0.5268432749478145	0.6225439679208303
0.8509822099239868	0.5320547563797327	0.5971187974764809
0.8335062321020257	0.5346018978162279	0.5714735286497216
0.7990720752291941	0.5318483307119130	0.5708672568503601
0.5042562687007371	0.2743719170173063	0.6353888422618410
0.4945598100033207	0.2185431137950686	0.6150643252477687
0.4691956018358533	0.2024342457729659	0.6000530048205946
0.4666644187846415	0.1680325043659312	0.6009707627457471
0.5175490109671750	0.1998966884440980	0.6307035115737231
0.5148459703308805	0.1654833025185224	0.6313214790590000
0.4894092765911947	0.1493642986242286	0.6166435921121021
0.2735188070320143	0.4854450409029794	0.5781131516384918
0.2190179630767560	0.4695227483939295	0.5981960302490987
0.1979523227347018	0.4902311452683328	0.5824696082543405
0.1725673198395687	0.4271209383930764	0.6898551698094082
0.2210783490243300	0.3920811633350046	0.7195312719237288
0.1887993370412759	0.3799361984966427	0.7181950124916204
0.4559192073367119	0.7224186571946986	0.7459211961967753
0.4539923526256949	0.7793104528756205	0.7263904924441476
0.4270963297943325	0.7922242436902025	0.7417011465622196
0.4224463415604025	0.8263992463373734	0.7429978598684608
0.4758623441639650	0.8008146118842018	0.7123688301780414
0.4710226415406102	0.8349393243710220	0.7139382314846768
0.4443698716022195	0.8479248869401148	0.7292947341522934
0.5745609899534115	0.4843039668149116	0.7122009037239165
0.5803312915993099	0.4290774824202953	0.7172537105501325
0.4853585154160036	0.4253245991598262	0.7190766195044515
0.4387016240617692	0.4554439848393364	0.7125232718912130
0.4295976550721142	0.4211681008449454	0.7138396877914479
0.4578613960653889	0.4029158565442417	0.7180490710631180
0.5621401909376462	0.5450752092850265	0.7122526946580495

0.5155317767718135	0.5751145832058429	0.7193346879803719
0.5430247594835007	0.5975282624263777	0.7183023625930308
0.5712654103384659	0.5793341208259386	0.7137712430483205
0.4262375653727812	0.5162012295882917	0.7128310868102344
0.4025857954510553	0.5434304864671147	0.7136657175935941
0.4205203564228240	0.5713641015467444	0.7183446233704951
0.5457329286884363	0.4392167405408763	0.7182586428578703
0.5982114192491230	0.4570515778879068	0.7126156487593092
0.4551415164969097	0.5612330186905553	0.7189354291319394
0.4818252297838135	0.5836430705050474	0.7217143975847776
0.4169925280047236	0.4826050041075273	0.7101833168320754
0.5190838506044405	0.4167807951668229	0.7210874413138414
0.5838128132324167	0.5179182108834263	0.7096447937462558
0.4747503030122860	0.6193433203847248	0.7271447225023184
0.4581849784962932	0.6392410514223016	0.7071350812148123
0.4527230068917578	0.6727950386718333	0.7124072244473657
0.4629196163262745	0.6870916871874001	0.7382394050401134
0.4789819695579780	0.6672231846325202	0.7585027069773292
0.4852791980957787	0.6340109841819931	0.7528538059134845
0.6191706140544108	0.5263458935420890	0.7040235847970473
0.6445087358721416	0.5191135167045436	0.7235980856655896
0.6772344583081631	0.5279957117093359	0.7179571206765686
0.6856090776036567	0.5437422569458704	0.6922441504160248
0.6604776759998686	0.5505680473871778	0.6724475724692176
0.6277515078015697	0.5424520380051664	0.6784278329773219
0.5262316798498327	0.3810164921220957	0.7260995773307608
0.5161030155025943	0.3659886149230827	0.7517750453833391
0.5226859575500661	0.3327562636070540	0.7570919194918785
0.5386336294118702	0.3132253525476825	0.7365146643170772
0.5483120037638506	0.3278538425227548	0.7106685305093005
0.5425584477596616	0.3614256773749694	0.7057231418022342
0.3815945607133662	0.4741106338717181	0.7047803049114844
0.3727849768854556	0.4585092846317274	0.6790092500760099
0.3400760686294843	0.4500553999733835	0.6733052355078709
0.3152384659214699	0.4560549473547339	0.6935693794391893
0.3238220108443398	0.4713342376402751	0.7194414035991891
0.3565225411584210	0.4806075729882325	0.7247891036923789
0.1643945925937943	0.3973622207864516	0.7033263952275118
0.7204220108373002	0.5524553273667289	0.6841506193478437
0.7737709053361496	0.5726521896677619	0.7046258489954756
0.7926240399963856	0.5697075398342325	0.7297075934037816
0.8258534965252442	0.5789526473763853	0.7298687461218307
0.8407348393161493	0.5911651741097260	0.7050618442887325
0.8219326764643143	0.5941075699462510	0.6801497066522009
0.7886093933891803	0.5850177330295490	0.6797015447471171
0.5461807712466441	0.2779066287664143	0.7438191798319290
0.5483025679659197	0.2212742717331970	0.7235311129945134
0.5270862273196436	0.1999141651216079	0.7086058509245199
0.5323276982145773	0.1658161949966184	0.7094791133144341
0.5749918607769187	0.2082295987869059	0.7390393555260159
0.5800579454622455	0.1740811248230361	0.7396111405212322
0.5587696404393450	0.1526965635665396	0.7250242239526696
0.2804552114078052	0.4466253524627345	0.6860711039201391
0.2292231030209102	0.4218969772794450	0.7058781159198687
0.2047881249764268	0.4394651420773694	0.6909887986633556
0.1876860190212841	0.3751176943378397	0.8003329645793148
0.2363534211102572	0.3608252575132833	0.8405047403983696
0.2052126468699186	0.3463255374122072	0.8428512647264238
0.4188932720893989	0.7097444018118200	0.8613564259494014
0.4134807137064019	0.7676608714952277	0.8433560120724665
0.3954878615446052	0.7809109229255311	0.8663954660177221
0.3898930027325117	0.8149726941628391	0.8676355582753781
0.4254058508479516	0.7888988556335617	0.8218889360535915
0.4196611963407525	0.8228285699126759	0.8235560877973712
0.4018893807556954	0.8361476547985253	0.8464554584206377
0.5762196545074076	0.4975844310467648	0.8250546791191965
0.5915406500766437	0.4441600353555686	0.8298608484120303
0.4990218485992622	0.4233514425283852	0.8322892252322840
0.4475671294573956	0.4438330243192553	0.8250891954409351
0.4451132498143113	0.4084882766873097	0.8268989604992932
0.4762944284558738	0.3960820183465193	0.8316335669942070
0.5530643925061729	0.5550369029959378	0.8252945676253551
0.5015957078914393	0.5755023996440558	0.8326014321958991
0.5243392879016812	0.6027678660824241	0.8320404170839304
0.5555222024882610	0.5903780937847543	0.8272417516067850
0.4243912279514595	0.5012656642227185	0.8251576176591744
0.3963363693990882	0.5239814635745190	0.8254528594328895

0.4090332081250411	0.5546291411230490	0.8303082098407929
0.5557684273212428	0.4482235198766002	0.8314706819308276
0.6042357503235047	0.4748124790951371	0.8250578525940245
0.4448298234849436	0.5506324398145812	0.8316940544373678
0.4669138879818457	0.5774570054226567	0.8348936774908229
0.4213207027775920	0.4665412752052316	0.8224591951379038
0.5336882434183304	0.4213983668013881	0.8345783525559585
0.5793025840727756	0.5323216209828033	0.8224750851432281
0.4532028971425032	0.6111565710378597	0.8408495730260185
0.4385866683832676	0.6305171905181873	0.8194255318957444
0.4277089587806771	0.6626532106215517	0.8251926796723645
0.4304128963042592	0.6757903983933558	0.8528141836744407
0.4438918458753320	0.6560000182805781	0.8745323505818687
0.4556018957580699	0.6243730321123103	0.8685483445823815
0.6125747806246451	0.5466135165849877	0.8162763974736943
0.6390162675483805	0.5443409388449981	0.8356704095484956
0.6700536543048988	0.5572887273122672	0.8289142407738094
0.6756572592435551	0.5725499788859694	0.8023862644987978
0.6493436708434493	0.5747872491076710	0.7829544772468952
0.6182315478295342	0.5624310133063019	0.7899590957363474
0.5473703865758655	0.3876865150521023	0.8404167974037300
0.5446426633056222	0.3742318053597040	0.8680021373870787
0.5562539425238650	0.3425502405455663	0.8738610758247739
0.5699656596990442	0.3229582767745732	0.8521202865681599
0.5730187889297496	0.3363514150754029	0.8246173196034098
0.5622174999656986	0.3685334000795530	0.8189776099090241
0.3880245549829349	0.4521204423403230	0.8165414917160065
0.3819851330523494	0.4366378360155111	0.7901337060031806
0.3509009139846720	0.4239181260418051	0.7835159807330538
0.3250704707428902	0.4254552229502100	0.8034627654613615
0.3310555092457151	0.4403711458667879	0.8300803917963154
0.3620397534395098	0.4537117065853016	0.8364319201009630
0.1806652651082006	0.3533701123668486	0.8227129113912925
0.7086380302159849	0.5861019462137613	0.7934600147177215
0.7573312777028001	0.6165401190172306	0.8148294891627103
0.7712570219841771	0.6249552762971534	0.8413887267616542
0.8014742003918238	0.6414378020891369	0.8427176497095167
0.8182781954578049	0.6497430107104260	0.8176167507519136
0.8043897543375053	0.6413966064709669	0.7912750237508264
0.7740822613420217	0.6249319631737955	0.7895811130120809
0.5813474034174664	0.2889132009314011	0.8605183066644339
0.5875363079921431	0.2313098433373309	0.8418728441171361
0.5765072015802518	0.2104296988078150	0.8197881187251453
0.5824506042236941	0.1765137366624036	0.8211040916837147
0.6048968298586483	0.2177324581921984	0.8651286841880093
0.6107060215402431	0.1836931788793521	0.8660020712094020
0.5995454857771439	0.1628613090934727	0.8442431634010643
0.2918447001734510	0.4120491508214000	0.7952286892958397
0.2433895056181093	0.3826313694417143	0.8179056480775846
0.2187865724856716	0.3898389270088169	0.7976953071560120
0.4537962959532238	0.6248936826832512	0.1689367214920636
0.4021066743700624	0.5977230065694895	0.2020368156045314
0.4843672761135194	0.3730740343957103	0.1768713126940557
0.3727613853233647	0.5207363836670051	0.1651062613023885
0.3801749775064006	0.4554291091400327	0.1742773680816335
0.5186622961430357	0.6327670566166252	0.1793744573661256
0.6231885369752341	0.5505299362836011	0.1765973929066950
0.6310097671144598	0.4852725961785146	0.1676530958117175
0.5693823411470232	0.6238539575247511	0.1451834690296756
0.6062264631918305	0.6705496127542283	0.1571865060532455
0.4755226623886825	0.4997521282121808	0.1720135167324395
0.6265603509411302	0.6265462530390050	0.2392369404457744
0.5898986009484638	0.5792040076301458	0.2268322049576589
0.5960103516729205	0.4034943492310345	0.2021522396261779
0.6453395034259922	0.3691343786653039	0.1936601104146399
0.5280325798628959	0.5060385303821793	0.1722171601219379
0.6554912040134317	0.4109034805032956	0.1094839789826914
0.6063260599275578	0.4468947750310683	0.1185847026848167
0.4130418555826627	0.4248273816428595	0.2250934606595285
0.3763125702932584	0.3769852251580256	0.2358542332052729
0.3534853701004879	0.5976300483796266	0.1045657121913396
0.3973546487944558	0.3362792763949819	0.1526715855386127
0.4342091333645101	0.3834520463202000	0.1422815487250251
0.4033561446691769	0.5630918139195695	0.1150377536695221
0.1764960128817058	0.6954257439506730	0.1531781628106943
0.2602718196621219	0.2123560612013817	0.1703656639266712
0.6973794344350538	0.3653116462748016	0.1854674978707345

0.7329480932458862	0.3343358205836992	0.2108927140876252
0.7795864766577487	0.2946897815771192	0.2128077645465721
0.8027818520558698	0.2709149694422759	0.1676430798276128
0.7783894188766211	0.2877922014921143	0.1208802462022945
0.7315665092737423	0.3275485630373408	0.1190195499406969
0.3447468055952379	0.3270597269054815	0.1619127060494787
0.2984639384751149	0.3058612325684012	0.1406957940256477
0.2594602780400829	0.2590074268040412	0.1353209498510158
0.3399176049837708	0.2601376340066558	0.2159717547846865
0.3009338451006964	0.2133146423715980	0.2105141444982912
0.2995992183882917	0.6291921937208695	0.1796543581972600
0.2734956069001133	0.6794193713235195	0.1168426575062617
0.2177724886162852	0.7050508173896652	0.1143895888319750
0.2481264642509149	0.6353435937951948	0.1978556044188528
0.1922730956853961	0.6603169904166030	0.1950828826847725
0.6594065246672111	0.6789335268185863	0.1672476238144170
0.6629584760004481	0.7440519873815982	0.2230045808932019
0.7020653904596005	0.7909794880883151	0.2194406168108067
0.7060224385006884	0.7009746808634225	0.1472442167200602
0.7451199051830905	0.7479923688115949	0.1437250220426361
0.7435948187656150	0.7933522752642862	0.1799961697074046
0.5493542346197087	0.3808923761962808	0.1674700006939936
0.3516262547022530	0.6300694091730885	0.1922026565025689
0.4314029111130442	0.6124003818379427	0.2809051063379416
0.3900260590941782	0.5825333734557206	0.3142600715535355
0.5070473130626174	0.3699337098975086	0.2880370142656274
0.3702605001464530	0.4948781647839697	0.2792271039736344
0.3896002155346892	0.4319466006851822	0.2873367880359614
0.4938454422101978	0.6319275477737673	0.2901817007636279
0.6113651221710467	0.5699621122896401	0.2884873412363542
0.6307906092670468	0.5071589577050803	0.2797969812730901
0.5439770036377762	0.6318230317676512	0.2558553191163667
0.5737820015850780	0.6840720942327143	0.2657580712483567
0.4752635499843364	0.4928498897560468	0.2846658928044237
0.6029884012884916	0.6466255221257831	0.3481450043841731
0.5746853403835462	0.5933281531021325	0.3374601877067440
0.6096413467150816	0.4178422145402433	0.3131871601748946
0.6638136835198032	0.3917097773198371	0.3040192207693060
0.5257456766692221	0.5091040685401506	0.2845166146493011
0.6684899217153392	0.4396697618228927	0.2219255795764989
0.6147200252704023	0.4674685999691304	0.2318383404666995
0.4255469064240244	0.4082527009081339	0.3361029714498163
0.3961062712969223	0.3553563104620510	0.3458726738259793
0.3338848364402197	0.5638213413790010	0.2211951112266827
0.4266253515025413	0.3183033890199705	0.2637039912491899
0.4572535266879678	0.3702156794654931	0.2546248292698007
0.3876359386196684	0.5361661723429518	0.2314104990588428
0.1557011694760857	0.6582101553646970	0.2713800200039674
0.2996909746873510	0.1798651025904066	0.2924177868640548
0.7177124435131641	0.4033409033570730	0.2960034786815114
0.7544849363770078	0.3693820234452158	0.3209235003929963
0.8093006453599536	0.3418797954297897	0.3219223484951387
0.8417826866181948	0.3351352810814516	0.2768028306457088
0.8185993559514088	0.3567718056472688	0.2310186215738294
0.7638278839051972	0.3842128989981591	0.2300881636649530
0.3717580763372531	0.3018513249239170	0.2716234925709909
0.3161308517126306	0.2805463129832486	0.2650069985754917
0.2809809606949590	0.2300867632179858	0.2657914057086212
0.3884428108703732	0.2314866029363994	0.3178171213045374
0.3537364165453264	0.1809519202897576	0.3182307606109526
0.2804590461315620	0.5921458249806079	0.2944011895848992
0.2591013253345011	0.6619716239731230	0.2485331484880920
0.2028803135753431	0.6863767734919625	0.2469944445805171
0.2223216119820989	0.5815363506751269	0.3000310848417094
0.1657683219191014	0.6054583527918254	0.2978866147321182
0.6288997245054454	0.7012370380684467	0.2752600119121289
0.6083295011484333	0.7716226631092329	0.3199550215577800
0.6425274434948919	0.8224691329257672	0.3221205272850007
0.6853367348032705	0.7225653358586177	0.2723712921760388
0.7199229755221355	0.7733443836244112	0.2748803623475009
0.6985811569114055	0.8237292970479689	0.2997557336596176
0.5694291291313135	0.3896059626311167	0.2788873397689333
0.3356592813206124	0.6084340031868456	0.3047408670209672
0.4114308109179732	0.5983641709340352	0.3879824622977973
0.3756402277787666	0.5630350419342314	0.4211414613022269
0.5284524732927449	0.3728471439694120	0.3970500477698389
0.3717996229246222	0.4717745291545646	0.3878750543438687

0.4020077063053645	0.4133395372719590	0.3960551707525778
0.4694636884013818	0.6285732727622947	0.3971597620695584
0.5960135777048713	0.5880964567827140	0.3961945009393550
0.6262681836305785	0.5296760918764132	0.3880664629898383
0.5182477506723040	0.6374536309857974	0.3636611765635472
0.5391431721802562	0.6938820743261471	0.3732443159803123
0.4755270400631973	0.4884199993159219	0.3924174055664481
0.5763877541398663	0.6617014216640366	0.4548426432670745
0.5569182980960702	0.6045012388297452	0.4445571904211666
0.6217788088975317	0.4384882346214404	0.4220439325907078
0.6795990553795908	0.4221696778899918	0.4125593136720084
0.5225555415573289	0.5129587534182632	0.3926841907110241
0.6748514780115287	0.4691087122032796	0.3300304000396381
0.6172049066003049	0.4871959101760874	0.3402937885333024
0.4411458582416961	0.3962168915158236	0.4441449657734172
0.4216273177490791	0.3388642058651259	0.4537217115963372
0.3234538733268091	0.5296679305403690	0.3294146118492352
0.4586542323535834	0.3080449906373599	0.3716543945120880
0.4797264489380297	0.3645980468204308	0.3628219943746639
0.3816241824698977	0.5134178384230950	0.3398711045842371
0.1302274948408620	0.5868291759755769	0.3790423153989015
0.3592133325709088	0.1484252771180388	0.4014656607125046
0.7306050426495583	0.4437485956784032	0.4036611076592356
0.7728425670535644	0.4184937671902741	0.4292049019942665
0.8315776960659161	0.4009287951384318	0.4306253019057003
0.8644132175670831	0.3973683804513905	0.3854151608138485
0.8374653430002874	0.4119408525601747	0.3390942458047854
0.7787130034833502	0.4292790050860035	0.3377251171294959
0.4069043361856866	0.2815722756874415	0.3799251905184525
0.3578033869339448	0.2497382871320075	0.3721549673938011
0.3324047754676542	0.1937997958622835	0.3734639307194718
0.4362572601040324	0.2160158583381200	0.4273562168463234
0.4115244795773862	0.1599641526864798	0.4280369404051136
0.2663920190964723	0.5497442285048936	0.4024186813576131
0.2301784195031889	0.6106895506848006	0.3539994476582580
0.1701346522216483	0.6226534075640939	0.3525276148567774
0.2118903606105887	0.5278731677602556	0.4098861247953893
0.1515459796441290	0.5392352527904025	0.4077768426178707
0.5899607321548720	0.7209529911530336	0.3815284257241322
0.5588788323435411	0.7848142817305633	0.4294281149274818
0.5820391359085454	0.8415348305807118	0.4309884865025619
0.6391123525133342	0.7539976416696292	0.3749055644122781
0.6629211703146553	0.8106148724884188	0.3770647238178635
0.6343956020188333	0.8548601520650425	0.4051020261480498
0.5865879269678714	0.4030223465678426	0.3882771624557783
0.3172338824887951	0.5774997882421998	0.4114510202434792
0.3963029867471566	0.5816912932576969	0.4951782037164492
0.3676505639083533	0.5407322681932097	0.5287146879363173
0.5513913162659736	0.3802744287654425	0.5040832652274891
0.3796814175894267	0.4501187252832039	0.4954975394877191
0.4196389427951475	0.3978577362033207	0.5034508889504902
0.4480418662468646	0.6216926257157617	0.5046086606526841
0.5796934692647520	0.6040939772684732	0.5047439518825153
0.6197211624072234	0.5519411236692185	0.4963352454039879
0.4953310066961586	0.6390817001979918	0.4713381820209758
0.5066609812816267	0.6982090755236433	0.4807404441501047
0.4789505818444479	0.4845688088742952	0.4997672857252510
0.5474067916710961	0.6730438456588910	0.5629510926574761
0.5377301000756266	0.6133713483630266	0.5528067596423255
0.6316972969537702	0.4612020918672427	0.5291315641271518
0.6915132214825593	0.4559669869417869	0.5193780945888512
0.5204708520394588	0.5173766578168859	0.5003069613235492
0.6777982154924146	0.5023808078852153	0.4373706566538142
0.6178368601909693	0.5093879868647659	0.4479381087490460
0.4609472570871495	0.3877532578442296	0.5512086791383719
0.4514803305259209	0.3278968003976436	0.5606374301700778
0.3214371906010007	0.4984499858906918	0.4373487852521630
0.4936738311302238	0.3040787051024661	0.4786940479288856
0.5047857505838483	0.3634185343221271	0.4700040790583727
0.3816002149639072	0.4925649562436717	0.4475104679709838
0.1216399506806247	0.5253157888771217	0.4874299123952005
0.4259849670974377	0.1288022446390609	0.5099970082223650
0.7377331125115654	0.4868241288527271	0.5106021823045664
0.7843866831642391	0.4681675238403743	0.5353442991991580
0.8453994817062943	0.4619108764569965	0.5358965129781421
0.8776451963635773	0.4663693272166691	0.4904340896743861
0.8477925466767509	0.4776728769882558	0.4447765233759984

0.7868322678274291	0.4838417662192251	0.4442801962515477
0.4475392584044173	0.2683873577266671	0.4867631372250358
0.4043186138224849	0.2276730554753572	0.4804384342181157
0.3901671312937364	0.1679279030794195	0.4825516929527344
0.4893790795529344	0.2101401416311043	0.5339145347119344
0.4758542226714652	0.1503967090759313	0.5353887543511990
0.2619727008754127	0.5100151939398698	0.5108991759864651
0.2167266013246477	0.5650415492476052	0.4632758517927084
0.1555148065618215	0.5675681352894929	0.4617565448048511
0.2114316560433672	0.4789314833001821	0.5174308943686922
0.1500318004126719	0.4807872416784745	0.5152508112674272
0.5523576065764754	0.7331682690171768	0.4894518764993107
0.5122333087742752	0.7918184986635197	0.5375046755084049
0.5271663246383715	0.8511993098414349	0.5394184553452893
0.5959026502680507	0.7730636722217995	0.4827276469257744
0.6114783446383595	0.8324423859294000	0.4852171274433493
0.5771260877241378	0.8719781770259833	0.5136523727875614
0.6031855180979973	0.4204139089124374	0.4954313396432289
0.3076061835088855	0.5449282829904903	0.5192692066905142
0.3838528500357876	0.5631534165886204	0.6029953987207375
0.3625877631325694	0.5174619650553522	0.6365830061333471
0.5710370379222783	0.3913424991887117	0.6110362839778524
0.3900125295501366	0.4306263977332154	0.6031258767654348
0.4384697352295000	0.3860981010933551	0.6111293424505935
0.4280824519550980	0.6113745776779853	0.6123107070073029
0.5607570234500264	0.6165685411843058	0.6124053735152098
0.6091897224371982	0.5722149996034087	0.6036876972393329
0.4717295825126631	0.6367960720167823	0.5790496290834560
0.4729542758385942	0.6969214249875083	0.5887796616512005
0.4817429324780421	0.4818458915230681	0.6072366620759847
0.5172505678629571	0.6783492643347009	0.6709150921718522
0.5177999591019836	0.6179738819779218	0.6604231328033104
0.6368098102923661	0.4837813402042084	0.6353090336824332
0.6965702549760610	0.4893156663745767	0.6255282779762131
0.5174621178043686	0.5209186496095939	0.6071109129179100
0.6747231759957429	0.5349930101613073	0.5445287090247849
0.6145345530743505	0.5310977039330469	0.5551391416955901
0.4817308120265599	0.3835751529676552	0.6592826586938797
0.4826480763632750	0.3230047633448608	0.6688393432744890
0.3242272732848001	0.4685215909006447	0.5449906201115318
0.5265249914874736	0.3063173256051272	0.5862510350012514
0.5273802817258306	0.3666200788594541	0.5774196848993101
0.3845265146951409	0.4733183125402464	0.5549972744548750
0.1243866381390106	0.4514482128088041	0.5956302109188576
0.4873972307162346	0.1224361529990660	0.6173445508581358
0.7366565484429132	0.5287714175216830	0.6173564896510109
0.7856307406686798	0.5203143470241161	0.6421671308705473
0.8467917160259901	0.5250328483927661	0.6428383079019993
0.8779093739294151	0.5342323623107001	0.5973293540342776
0.8467248252059871	0.5387861301465240	0.5514458903566015
0.7856641378430824	0.5338016204310022	0.5508116769749345
0.4876518840572254	0.2636879412195366	0.5947118327614610
0.4517545311346833	0.2170434271137241	0.5874870155980633
0.4467179384213684	0.1558454914465320	0.5892785001085062
0.5373789988736115	0.2122860012754385	0.6422501837705831
0.5329746908357849	0.1511985736320046	0.6434431222490693
0.2646088919435809	0.4674838592982697	0.6186024082447905
0.2080633348803574	0.5113102496833526	0.5709205585592262
0.1478433156988957	0.5000266669251063	0.5696227771340433
0.2223331024080224	0.4267621383812842	0.6257055752138718
0.1621050345547784	0.4147724955935541	0.6237245102656451
0.5122480427838667	0.7388422238103863	0.5976917077943420
0.4639685068389764	0.7900447565379602	0.6468335126335522
0.4692228140037139	0.8510317857206837	0.6487536671235448
0.5478118779737888	0.7852211759566375	0.5900212692107052
0.5537886411194864	0.8462936114373870	0.5926270458775836
0.5144467628094360	0.8796721410935724	0.6220933440193648
0.6152338300551616	0.4395945274217086	0.6018747968866104
0.3026788487047916	0.5109783670969436	0.6273142679506973
0.3759173046126002	0.5414034552309755	0.7113959946366208
0.3628447018850560	0.4925695190244644	0.7449746125865229
0.5895984588851618	0.4039847362020672	0.7194772614732228
0.4044911849297130	0.4119209795702269	0.7118982821435683
0.4597950329733529	0.3762359171142112	0.7200677904621960
0.4112694267015154	0.5964253226925922	0.7208775376705140
0.5411359362523691	0.6241867121804541	0.7205742410238740
0.5963666052751658	0.5886092531331502	0.7117784938064068

0.4500142510899713	0.6283904809039037	0.6869849476577645
0.4401498129507795	0.6878195615269387	0.6963364113753541
0.4863162151192059	0.4778665994602176	0.7156379283081801
0.4862901401946375	0.6782133644296242	0.7788409410687174
0.4980951862261700	0.6188965576408252	0.7687303866297792
0.6383759318807750	0.5068352545903415	0.7436800828742056
0.6963180389632845	0.5222259546002905	0.7337091837803115
0.5145537219017147	0.5226218578577081	0.7154901520145182
0.6670689076695918	0.5622616540074687	0.6522223639198266
0.6083982911519576	0.5481680499283600	0.6629090961304449
0.5033866131719610	0.3808421406577054	0.7678915119313188
0.5156961897480278	0.3215091804666192	0.7774109167378154
0.3333302031302474	0.4386491074030193	0.6529933417580087
0.5607563784561215	0.3130848773462613	0.6943466821751964
0.5503696129548683	0.3725415681804041	0.6855720527941878
0.3919632238527977	0.4533581145377119	0.6631848284227233
0.1391577918611826	0.3878080830453390	0.7022268894176713
0.5628138846030318	0.1260004356987378	0.7257162222986575
0.7291909973553384	0.5659947747072379	0.7257394586131902
0.7809580615429316	0.5602294710404692	0.7490908930545915
0.8401530048699246	0.5765067452274043	0.7495460826425007
0.8667782102018425	0.5983538014036537	0.7051670597056372
0.8332332865835729	0.6036476770208125	0.6606269205120036
0.7741380213469801	0.5873506461356288	0.6602225888620580
0.5308580737283366	0.2637926588568107	0.7036046846019030
0.5066461589715726	0.2102452411136727	0.6962289929651757
0.5154672090401489	0.1494707943957003	0.6979448468108042
0.5916019713915620	0.2247529859895885	0.7505171227390591
0.6010075622368418	0.1641907125702209	0.7516172634048768
0.2741900013493030	0.4281953649342823	0.7267705021535226
0.2110454913563463	0.4626230494864285	0.6803964896172957
0.1537049418916334	0.4410376940453482	0.6782624232295020
0.2402046432174077	0.3785463496492052	0.7311379247958709
0.1828259440545900	0.3565752790676874	0.7287262283070369
0.4704860504159489	0.7369489723116115	0.7055386961661065
0.4100165406185988	0.7755592655039519	0.7524986281347308
0.4013785003840925	0.8361999716963413	0.7548981661227836
0.4964894666457078	0.7905836210101835	0.7001647360412844
0.4883628839654575	0.8514153766777033	0.7030933967991136
0.4406588106580168	0.8746465627041998	0.7305402877929229
0.6248509127343503	0.4590665536472171	0.7100764931651483
0.3048703724418750	0.4763395034676884	0.7355052803367983
0.3704831519866782	0.5173773488340747	0.8226124769192336
0.3662106957245903	0.4656220692047452	0.8570538711076648
0.6048816550610984	0.4209187134868795	0.8317182946436473
0.4221871196905099	0.3946255929323336	0.8252572989047352
0.4833263652430950	0.3703522405351045	0.8343604328052340
0.3956575807181877	0.5778283811068347	0.8323691609658759
0.5173254534289902	0.6284916768988638	0.8348678343006699
0.5784526755406871	0.6042486717195977	0.8256446637001864
0.4361764216570684	0.6205069467500982	0.7978692980554901
0.4168925631822453	0.6772492535556442	0.8078966347210348
0.4896695765006683	0.4751542500178587	0.8281730689544291
0.4450320994969487	0.6660986204910133	0.8961364373547599
0.4666562578677843	0.6094517101440510	0.8855073720384269
0.6351573875277045	0.5321783841879327	0.8562286853975689
0.6902350484357616	0.5543718523858875	0.8442745358627604
0.5109546018815030	0.5237146704989228	0.8283048289733125
0.6537270433856120	0.5862517694445236	0.7621933988455630
0.5979725227581838	0.5643925067043116	0.7746079471613306
0.5333783944267380	0.3889994183952246	0.8849544357510960
0.5548441174608134	0.3322182300369140	0.8953656251863344
0.3462309310194857	0.4127728049947775	0.7626728437498546
0.5840244669828860	0.3219348644127036	0.8073014960538044
0.5648549606994256	0.3787473903745341	0.7975130215673494
0.4019308348209235	0.4352391329096035	0.7744300863657712
0.1562496659367116	0.3420233019051001	0.8245155865375909
0.6040531833974353	0.1362523264210937	0.8453019119273437
0.7148288377311092	0.6005063016778479	0.8345776606464124
0.7582680799550344	0.6184356947177087	0.8611042770957854
0.8119451867534757	0.6477835355345248	0.8635048711761016
0.8419597156655337	0.6626765781672329	0.8186276752106759
0.8171926032034036	0.6477848841358306	0.7714853562051541
0.7633493068319713	0.6186172699220075	0.7690099307357988
0.5703118597988179	0.2721350170850498	0.8201279051385447
0.5632377679815685	0.2210519548041742	0.8016169609477750
0.5734186123885862	0.1606839023975996	0.8038828202874032

0.6132600690955213	0.2338112844318904	0.8822795134081285
0.6241604693298191	0.1734005518611640	0.8841850390093283
0.2887820698487091	0.3935604357374068	0.8353816236880907
0.2240521782593821	0.4066793599031697	0.7801667731726349
0.1686937974960416	0.3809625261770516	0.7845256974665366
0.2555332390977704	0.3551768220337833	0.8562537339315576
0.2001623103895924	0.3293979853690751	0.8604929961169685
0.4309912171330171	0.7271115795038335	0.8213647096166490
0.3864728684329586	0.7645653208419837	0.8831100584912096
0.3758928197073325	0.8250015636070643	0.8856255560489988
0.4392011182739972	0.7785328004149334	0.8039063775429270
0.4293538916630494	0.8389190280322112	0.8067863676694850
0.3974753911023024	0.8627559114909199	0.8477677824080867
0.6300706062279803	0.4813647003315253	0.8220573670140656
0.3111286790698364	0.4426384818109868	0.8457553517745021
0.2955770298577358	0.6402748732713626	0.1603260000004617
0.6597980893428612	0.6924858697189334	0.1855727401240773
0.5076995701365116	0.4529312716312902	0.1718662829593673
0.4504001662726438	0.4968239256454131	0.1715434919308447
0.5531511210253411	0.5090376287971607	0.1722055208896154
0.4959097027320317	0.5530128171062774	0.1718670719018293
0.7022827724589871	0.3556943328759261	0.1656878153262210
0.3438870773178037	0.3129781640428699	0.1799235138780418
0.2760693618823462	0.6056987937681214	0.2762524742069110
0.6256330347059375	0.7145904158015084	0.2936869007820870
0.5153874741626489	0.4527996761750526	0.2843057005258330
0.4510684375789791	0.4854487137891372	0.2843753320435904
0.5499365839431459	0.5165118973856945	0.2844424989758548
0.4855780469489120	0.5491741785918512	0.2847924472607133
0.7241289112200231	0.3938725170942524	0.2764281344326990
0.3739018300752265	0.2883146770495858	0.2900553414806640
0.2590200900162513	0.5610847640472177	0.3838449579039830
0.5848624026441384	0.7332227888847974	0.4002405465199617
0.5222818144109193	0.4558495168758133	0.3926076807926967
0.4530452700837159	0.4768119541804481	0.3923501570627729
0.5450211668765030	0.5245916686626053	0.3925707059587970
0.4757905685248797	0.5456101216667518	0.3924785512332348
0.7383215143237611	0.4346956193549354	0.3842775904371544
0.4119929788868031	0.2687616578686953	0.3983531730472286
0.2528625831631769	0.5198420036181888	0.4922548951009955
0.5452932759850201	0.7445876080303916	0.5080891550224702
0.5304618967558440	0.4608459308238423	0.4999942390695882
0.4587983611578263	0.4692753537134399	0.4997072083606344
0.5406090382561354	0.5326881616619098	0.5004009364254662
0.4689295139360826	0.5411436141480497	0.5001747232672537
0.7468029366152843	0.4796228608939319	0.4910681459102929
0.4548624353006668	0.2569597150505409	0.5053276877914380
0.2535142491056694	0.4754345569556455	0.6000988618583175
0.5034677504466927	0.7488615655581223	0.6163742869589385
0.5368081972786632	0.4672325543562593	0.6070454581860026
0.4646217581945817	0.4632184919226877	0.6072124662021124
0.5345857948550932	0.5395421203918591	0.6073175084786683
0.4623703556638016	0.5355775874722820	0.6074606005917889
0.7468178375838288	0.5236314732116030	0.5977488356104030
0.4965481816579509	0.2535127893831519	0.6132953175554323
0.2621556711558610	0.4337666916534951	0.7080894809627764
0.4593709160093253	0.7447594019868053	0.7240755671276030
0.5428462174273786	0.4728662749735727	0.7153846714669422
0.4725268777595650	0.4566527892327940	0.7157663780887771
0.5283391275193835	0.5438302284157513	0.7156725583519626
0.4579957899360249	0.5276203061259184	0.7157851453716915
0.7399455102807587	0.5636415945470971	0.7060075679489294
0.5424632303187499	0.2557799364593308	0.7218313378473292
0.2752022305330021	0.3969810830957891	0.8173047388401313
0.4204129461892022	0.7336026432704008	0.8403991851450677
0.5470112782775971	0.4808445288058521	0.8286400543851286
0.4804873209716686	0.4515794067116688	0.8283844624841320
0.5201423461228715	0.5472864149622495	0.8286129599662662
0.4536023530239566	0.5180318913433229	0.8286536926921610
0.7263288482351239	0.6004744318576699	0.8152278713148012
0.5803949036769143	0.2653566755423735	0.8392801603752382
0.6894803160961668	0.3607145133176251	0.1177983774590061
0.3599702720579540	0.3140171499155173	0.2272815317012154
0.3150246274711257	0.6442358723791920	0.1140924978874026
0.6425348493809211	0.6901311510961220	0.2325613055806024
0.7070944920908515	0.3884748739223636	0.2295138554708022
0.3907702645011354	0.2906732818476269	0.3370869605370844

0.2942337731704087	0.6150892187698160	0.2301715597918485
0.6063402937952720	0.7118086713262106	0.3399906281604481
0.7219056648017976	0.4249235315913503	0.3377412205186203
0.4293951891324782	0.2743272641530118	0.4450662259297072
0.2743783983413625	0.5721556272759096	0.3372925951804896
0.5692380665556044	0.7260818151902158	0.4472414725589200
0.7321462360713177	0.4675271096795990	0.4445157064591881
0.4700844916475123	0.2659000755211802	0.5521794033898022
0.2656418053058236	0.5314230375323190	0.4452764984085374
0.5298495362659352	0.7353449968995793	0.5548734548016561
0.7344956949430071	0.5095801005110560	0.5511328265407799
0.5105502619152564	0.2647966173255369	0.6600983551599906
0.2633884979748656	0.4908735731162124	0.5534096190896529
0.4895835722788106	0.7369226899467276	0.6630711875487092
0.7301068255164055	0.5495584276661294	0.6589929598370750
0.5549703660085371	0.2700692128990501	0.7683746901444231
0.2697263935174689	0.4498884633655063	0.6612881477067357
0.4478454149682772	0.7300774725010482	0.7707054813084641
0.7184219516454773	0.5847264862467830	0.7683067289773194
0.5901602007288602	0.2832263476720455	0.8853675578406754
0.2807978573187545	0.4143065582503206	0.7705310061706857
0.4095346034177681	0.7151076123825892	0.8861091868335759

```
N-CENTERED MONOMER
   1.00000000000000
    39.4380390000000034    0.0000000000000000    0.0000000000000000
     0.0000000000000000   40.0849740000000025    0.0000000000000000
     0.0000000000000000    0.0000000000000000   18.2582340000000016
   C    H    N    O
    72    50     8     4
Direct
  0.1741121512254819  0.5914715349394526  0.5050388142100388
  0.1911877210129885  0.5550880491533632  0.6302460798666618
  0.1578014491854570  0.5652455230037546  0.6190261952450525
  0.5523254805482469  0.7425589320845406  0.3854776439768315
  0.5534097952501078  0.7797340489017996  0.3964052649771111
  0.5330493038396116  0.7969215209883934  0.4470020081779580
  0.5352187053197651  0.8315579891330939  0.4533685755015595
  0.5756111460451330  0.7979734289738281  0.3516359739067098
  0.5779357340559300  0.8325244843328242  0.3584072243956085
  0.5578087200184397  0.8494168150152300  0.4093961714786605
  0.5463342019395289  0.4033534171917689  0.4994953367615559
  0.4551924411389654  0.4370232007810753  0.4954837226496313
  0.4231398527942608  0.4836782787832988  0.5040324560251369
  0.4007747528145429  0.4554833206228843  0.5025622577129220
  0.4202213912679051  0.4271616125479514  0.4966759646801006
  0.5768192390718219  0.5178564314336747  0.5096390030521801
  0.5447581869431416  0.5641991071836011  0.4939322565923399
  0.5794878617864238  0.5743687457124813  0.5002260238074039
  0.5988797897537892  0.5463090316012662  0.5109139985507158
  0.4365250060769562  0.5446676364917041  0.5033335048970158
  0.4252093081939921  0.5792023801500602  0.4986483454121819
  0.4536390137394953  0.5980411474275387  0.4908151869577533
  0.5632889904664230  0.4567852373121861  0.5125192180017490
  0.5177547440169389  0.4263876055782214  0.5003193293441883
  0.5746770786703842  0.4222534911727910  0.5080031127115309
  0.4821798242583819  0.5749037433296437  0.4926589496266251
  0.5163541343147620  0.5852735973322297  0.4881520942670937
  0.4138629402089051  0.5174775379822719  0.5073685179047338
  0.4836659553206650  0.4158856690633224  0.4938359784195659
  0.5860320936772034  0.4840674439120502  0.5166617667178296
  0.5234206218041914  0.6215324495813875  0.4771568529670855
  0.5352819786818331  0.6332606064898580  0.4094403221732782
  0.5414088777801338  0.6670207410460627  0.3965227526975005
  0.5358060439733994  0.6902051941257100  0.4529525335488198
  0.5242690681125405  0.6787160250186310  0.5213605513198381
  0.5181136042646538  0.6449749015622013  0.5331214468983623
  0.6226101649924143  0.4765313393143757  0.5286805839561062
  0.6332205838459869  0.4606264941971980  0.5932837589639206
  0.6670824796584948  0.4527318600045162  0.6064175390478800
  0.6916978394802507  0.4606098748503499  0.5535337093301518
  0.6814717491594244  0.4766011187049860  0.4885853587176922
  0.6475643605447099  0.4844650695032895  0.4765487029059299
  0.4768078980153895  0.3795093706223593  0.4849935539644593
  0.4829854374442055  0.3567951593376394  0.5420309931508099
  0.4770084290751928  0.3228811123472464  0.5326986823876004
  0.4647950395875008  0.3104471546838881  0.4656585932608396
  0.4582254711367172  0.3328829128121242  0.4082630632319238
  0.4641869549395420  0.3668379181183423  0.4187424111304404
  0.3768197234600796  0.5248388799947861  0.5147714413225344
  0.3554170006347407  0.5263785616096254  0.4534843686659146
  0.3210897623368697  0.5336015190927307  0.4612911340067815
  0.3070277385364761  0.5393643226391560  0.5309296750692921
  0.3281214521086064  0.5376541656890758  0.5928326442427152
  0.3625017991471996  0.5305794820905643  0.5839207461249000
  0.1491631155942313  0.5834216498394013  0.5563657555458776
  0.7427136005681028  0.4372883373155819  0.6186833956881722
  0.7799644072528282  0.4314751527760109  0.6069341266287196
  0.8001700544524913  0.4280074374773548  0.6697479505682395
  0.8348461184580401  0.4220343106949151  0.6635009923274157
  0.8497611522518850  0.4189930616090863  0.5943374287904304
  0.8297709423810464  0.4218508147214244  0.5314931570740864
  0.7950667355703155  0.4281340482006893  0.5376800374102464
  0.4487355874755778  0.2571485082317482  0.4014449008054929
  0.4489622372262227  0.2200750272375012  0.4138788219792721
  0.4268242816677482  0.2007626230546089  0.3711937754385853
  0.4257095194897692  0.1662283665462729  0.3795714401020168
  0.4705432550847568  0.2040015760721473  0.4638590019123273
  0.4695680600159676  0.1693903039308790  0.4718718226488231
  0.4469983143457927  0.1504478047557906  0.4300837936130356
```

0.2518747642880108	0.5514333876113442	0.5950818749730779
0.2163329336280914	0.5626041679419859	0.5784288594222066
0.2075177197136338	0.5810797073141266	0.5159344734788668
0.3990130055923836	0.5875840671781443	0.4999443376780796
0.3787439014732106	0.5295308191446029	0.6323135666723411
0.5448608034520602	0.3764912796533487	0.4923696928492031
0.3733326831591218	0.4571286469183501	0.5055546111805467
0.4114610924527352	0.4015172775056448	0.4942518313608120
0.4553093170775595	0.6249261792107497	0.4842084450373770
0.5880541040059667	0.6000869611068239	0.4984186830696661
0.6260718707354705	0.5451481088385429	0.5195738937425736
0.5394792991848251	0.6154458525524875	0.3650933863537467
0.5503152214822774	0.6756755539370144	0.3434613032984267
0.4771299281245946	0.4855397088245287	0.5015261372895824
0.5200331018715871	0.6965033786499932	0.5658922834971477
0.5091319356013680	0.6365763240530642	0.5865918337242106
0.6143284287694178	0.4543602525671848	0.6347795315272632
0.6748142571456079	0.4406185206069541	0.6570062310287361
0.5230751596985546	0.5155070770752729	0.4991523167222365
0.7003183448666942	0.4826753195466329	0.4466751616403115
0.6401922151359355	0.4964387314601232	0.4252467804182413
0.4924919614318097	0.3659305273115394	0.5944690429245978
0.4819253815762468	0.3056929570731587	0.5780151783968110
0.3049172644241355	0.5348697502853825	0.4126736725424651
0.4487414257939151	0.3234749947498072	0.3563039636394694
0.4593009531008808	0.3840246289346618	0.3735349155814863
0.3658663887577714	0.5220696240923259	0.3989220512296318
0.1230367547166000	0.5915483443485807	0.5478278630694822
0.4461653415142906	0.1233852745910950	0.4365510244718643
0.7413791477329171	0.4610164285189066	0.5198247707083683
0.7879358111623892	0.4299723488344438	0.7231822837151256
0.8503409717855211	0.4196141949344197	0.7127960909297938
0.8768969550013722	0.4140938571907177	0.5894172880990183
0.8411585246503154	0.4188487908557603	0.4773995881514075
0.7797586956966607	0.4292261270660749	0.4878000741128573
0.4653531967772552	0.2627886932280398	0.5064821478892297
0.4107145726951099	0.2135186374767877	0.3314727503311487
0.4081898154451131	0.1514884327031058	0.3464405509487327
0.4891315265587225	0.2181712755016656	0.4955777858836232
0.4866490801312375	0.1571946346650638	0.5105620229163860
0.2605346021480602	0.5490780260691873	0.4850138094723772
0.2268393648920119	0.5885506953485973	0.4762647198884565
0.1676441618973784	0.6061389093932984	0.4566308573209304
0.1985434800715869	0.5413243273154724	0.6793123037018751
0.1383943204107841	0.5590843103282835	0.6594771249287160
0.5363740038372961	0.7382641069673821	0.4913074437644816
0.5144714854853162	0.7836389393633313	0.4805462520201254
0.5190403407234960	0.8446297576775432	0.4924841707107914
0.5908083538985911	0.7843539228495801	0.3116174372931167
0.5955107139978243	0.8463984985853952	0.3236679678664171
0.5595615787142845	0.8764942725888203	0.4145448141128485
0.6008532266858497	0.4138583492627307	0.5091861645272335
0.3173949759220537	0.5418219861802247	0.6469166261748925
0.2723161430892448	0.5471878046937541	0.5343997864571122
0.5408672200579496	0.7247247567665194	0.4452012127574144
0.5286560877959785	0.4587338470983603	0.5077368236129913
0.4556373736985249	0.4712928651759467	0.4997537746997545
0.5444610068065997	0.5299420368808851	0.4994923579226141
0.4712024206642788	0.5426268928397927	0.4999120587573072
0.7263135497318812	0.4531096721938727	0.5615866421969118
0.4601162394238757	0.2758050793039917	0.4600161310778121
0.7284873945548220	0.4285417979112902	0.6758460136086667
0.4387624460342592	0.2694283019635949	0.3432250001894006
0.2614536393493734	0.5464782425901198	0.6585614695865157
0.5614225401862986	0.7295165385577843	0.3273899413991409

```
N-CENTERED DIMER
   1.00000000000000
     40.3870999999999967    0.0000000000000000    0.0000000000000000
      0.0000000000000000   40.3914600000000021    0.0000000000000000
      0.0000000000000000    0.0000000000000000   23.2582340000000016
   C    H    N    O
   144   100    16     8
Direct
  0.1795528866418027  0.5829462129667711  0.3703338232841665
  0.1958398485088437  0.5345082638554278  0.4513987427944288
  0.1627379889981889  0.5413389723262051  0.4392331175535288
  0.5445587104691790  0.7440207165038660  0.3097695500590288
  0.5442746061631518  0.7804492353386999  0.3231131182664971
  0.5266498992156780  0.7944751771257604  0.3690759018691129
  0.5264619758822286  0.8287562156839514  0.3769904639362829
  0.5615959173346448  0.8013139310895130  0.2853436542631680
  0.5617505801865345  0.8354525774750785  0.2938184069368471
  0.5441020948991230  0.8492911205111776  0.3396341642934508
  0.5437928887986934  0.4022320999039996  0.3803517386079402
  0.4558881320577414  0.4379474918486094  0.3712529413118792
  0.4250413362851705  0.4846983486477910  0.3730081714030176
  0.4029054006702474  0.4569050289297403  0.3731901251548280
  0.4216431790834097  0.4285412501612610  0.3714246276820443
  0.5768205897072382  0.5150437126627467  0.3799757434399714
  0.5461821854159752  0.5616442958119048  0.3703076165601522
  0.5804010221202599  0.5710581729204083  0.3727425324736304
  0.5989795065252342  0.5428103083355208  0.3792571399720673
  0.4395413050676596  0.5448785864145278  0.3722643589799928
  0.4296879169304111  0.5795545520840306  0.3696344020556521
  0.4581651702566312  0.5973918889861157  0.3661187409201712
  0.5622035916167148  0.4549143833691388  0.3836668711184629
  0.5167742482429020  0.4260747415805782  0.3762899544167161
  0.5721014209776306  0.4202172076849097  0.3852854404396456
  0.4852192282663313  0.5734727061783466  0.3670306212874940
  0.5188724064039285  0.5830473435144948  0.3659990395064612
  0.4166175220357768  0.5184895465890684  0.3742250151591317
  0.4832267966478501  0.4164576325847963  0.3721481349881020
  0.5850929613005388  0.4813577901510636  0.3853399873105671
  0.5260740291693909  0.6193143859108540  0.3619197514652895
  0.5252256847766392  0.6358957985340323  0.3089813781596526
  0.5300276156195567  0.6700291899919981  0.3049756117650977
  0.5354533924392174  0.6885981426746766  0.3552056715842029
  0.5369738641975491  0.6721561421465454  0.4084118432865436
  0.5325276625396440  0.6380363735550453  0.4114725924472206
  0.6209392377495235  0.4733520291051816  0.3935269973299840
  0.6329032179538046  0.4634453150496195  0.4475278969915792
  0.6663141120810337  0.4564324107233121  0.4567014191941989
  0.6887383985149663  0.4590206421697015  0.4109059710920885
  0.6770620448023212  0.4684356992354303  0.3564476165040910
  0.6436708480016277  0.4755985773974279  0.3480301520596967
  0.4758810778437240  0.3801540267357847  0.3694963253394829
  0.4697506908951161  0.3618232114396785  0.4195923150303327
  0.4645495632061556  0.3277843642725374  0.4172505650793945
  0.4650631247692402  0.3110383629729248  0.3642691312740335
  0.4702433653972476  0.3292346282132345  0.3135471279945883
  0.4757292614138591  0.3632836757880640  0.3168239733238368
  0.3805548256234320  0.5264037914845127  0.3782092511636115
  0.3591100108599471  0.5211119334813381  0.3315957129113537
  0.3253293467405223  0.5274086236568777  0.3364431776759444
  0.3119599485083361  0.5390139017112614  0.3883931429947961
  0.3330964670615135  0.5449188982270378  0.4350881997994013
  0.3669247478507803  0.5387397245522374  0.4294595416068210
  0.1545068207356456  0.5655459109836191  0.3986873433237796
  0.7426030345830996  0.4527908865824549  0.4643411865379077
  0.7780485297270001  0.4432009449989909  0.4541682412016448
  0.7953714242053016  0.4288118985129522  0.5000980996869693
  0.8282897055103015  0.4192103850278351  0.4934224338675517
  0.8444918581222500  0.4243334304486519  0.4410653849568088
  0.8276453189016810  0.4392028541498112  0.3953925005808049
  0.7945685571898822  0.4484873350436093  0.4018081999648083
  0.4550179703680500  0.2555245094486811  0.3197983404098922
  0.4546366118489262  0.2191532455679472  0.3335917566407171
  0.4378497252896995  0.1984187423417338  0.2948940814717121
  0.4371961420816043  0.1642935305381204  0.3033924194542795
  0.4712070803059624  0.2050054288309299  0.3806080759915557
  0.4709154456482895  0.1707243408281033  0.3885799439684605
  0.4538327114073936  0.1503238470108479  0.3502263805189653
```

0.2563960915800435	0.5432652175937821	0.4368416423919489
0.2211270554821021	0.5514824744454963	0.4224462717081378
0.2127167871230685	0.5758788953179655	0.3819497144438186
0.1731480503285394	0.5483595577271744	0.6045637667858019
0.1904213360673301	0.5059860456822820	0.6944128611694702
0.1573690763373631	0.5145051060694940	0.6858042485304282
0.5088415003779090	0.7471038542387474	0.5485698043620396
0.5083149563221501	0.7835497574822844	0.5610073896757577
0.4854730257733930	0.7979413294657656	0.5990670456143088
0.4856575477152950	0.8320745575367774	0.6085497551180600
0.5311907598374948	0.8038545277966086	0.5324694169397355
0.5316389963899649	0.8378599011072497	0.5426548259648847
0.5088671620530133	0.8520573574382913	0.5806438490997429
0.5620819280000712	0.4134820642711257	0.6307718096635416
0.4684946089887177	0.4294471429052005	0.6251884839429878
0.4293046883890245	0.4694782087883649	0.6269099083274647
0.4128713274846900	0.4380144446568908	0.6251841577639957
0.4366557968275606	0.4137152217401001	0.6234971875877765
0.5696982520246138	0.5310353298173837	0.6330076011173552
0.5306989813272858	0.5709797557167755	0.6243537064213409
0.5625335195155592	0.5867163306833032	0.6258992413859780
0.5861661562553260	0.5624906270337424	0.6318471430555295
0.4307975679004036	0.5315173565262312	0.6246441337565463
0.4133693825583630	0.5630963878877943	0.6218635488736659
0.4370790536142106	0.5869134646474132	0.6186242591739480
0.5682175758004814	0.4689585118588196	0.6350812846463467
0.5303923508076116	0.4306708607414357	0.6282447470853111
0.5856860454287975	0.4373490201523134	0.6351134149504017
0.4688121310362792	0.5697239775292343	0.6198209924281717
0.4995788823780751	0.5863695097834450	0.6192688113265398
0.4141462591867400	0.5007795688629866	0.6268229975739461
0.4997094574873107	0.4140209026234319	0.6246835357043250
0.5848177625922154	0.4997604546938799	0.6367146339543913
0.4995796647237237	0.6231892202083685	0.6129845634907111
0.4913703682564483	0.6380776826492230	0.5603219231799353
0.4920776929485320	0.6723507950958431	0.5529725893326087
0.5009143668510231	0.6927110835727409	0.5993198424733364
0.5086177061820377	0.6782092902359262	0.6525125056187739
0.5080420983571724	0.6439531985646862	0.6590979467691216
0.6217841638230741	0.4990879991128669	0.6400436918318035
0.6383074433053364	0.4912661655303493	0.6912851198462950
0.6727310770231771	0.4885711680912724	0.6938226792216350
0.6915933396786084	0.4933695688054260	0.6437215952616372
0.6753199860876999	0.5020101883594095	0.5924370618505267
0.6410326241090752	0.5049722639944846	0.5908581586861875
0.4998646242366946	0.3771251560224506	0.6196492642505304
0.4906984858121912	0.3568623493701800	0.6660078504758216
0.4901837295255821	0.3225414156876020	0.6604395975526077
0.4986264976522231	0.3075050090220038	0.6080415417332619
0.5081856492287307	0.3273689318208270	0.5614876065771525
0.5088588851838212	0.3617105547890504	0.5678416419066915
0.3770915465753584	0.5014847936567206	0.6279534378834568
0.3585980663362677	0.4993938106928565	0.5770399790497349
0.3242326795473299	0.5020487435002419	0.5778032872125257
0.3072518793536763	0.5066772375765539	0.6299486604944288
0.3253961970882476	0.5077858638546561	0.6814820950943041
0.3598399649254977	0.5053790413987793	0.6798185663588047
0.1486376966231573	0.5358337256812302	0.6409141794507659
0.7482064641493100	0.4883064401051923	0.6863530349311820
0.7841927709667537	0.4854490744691922	0.6702009602088413
0.8059067457590883	0.4742803102164023	0.7131662455509590
0.8397463502950656	0.4717076158134611	0.7024485587447153
0.8524928540787670	0.4806808416598894	0.6487552956133555
0.8311403795273963	0.4921440402274158	0.6059154712502069
0.7971746184939049	0.4944413428688487	0.6164436498773238
0.4911456450967173	0.2525056524971105	0.5589569241772099
0.4919637249354463	0.2162297346052791	0.5728598259138304
0.4695056885103215	0.1952342900940479	0.5448246726472534
0.4694125975808962	0.1613821426805756	0.5564842469356479
0.5147326986814924	0.2026662580707605	0.6119706523873850
0.5148754009237314	0.1686905712759605	0.6230092409440453
0.4921028885598925	0.1480157619561706	0.5955321953259161
0.2503320771563919	0.5089831451493457	0.6718971010017317
0.2151936398158711	0.5182061572986343	0.6577442995933734
0.2062672942580023	0.5394854180876891	0.6126248438327716
0.4044168734237349	0.5887867308782025	0.3699124982384491
0.3832091100606133	0.5433848472429199	0.4660677764673441

0.5411865732536989	0.3754652072286641	0.3793876780430493
0.3761080155276880	0.4587284381221334	0.3745523884624117
0.4129281770700039	0.4031195354164060	0.3709548206725153
0.4608413302768084	0.6240976859566223	0.3631688655431042
0.5891916672772703	0.5964147751499094	0.3702516516595304
0.6257012640698334	0.5410220717237558	0.3831559350422939
0.5203829209902825	0.6217927392732732	0.2698019555963708
0.5294766807207385	0.6824280874693287	0.2634911378551800
0.4776037333947962	0.4862666034411184	0.3724779837532641
0.5409914563505318	0.6864612259712325	0.4476668025507385
0.5335549663165779	0.6257430670975093	0.4532515596289077
0.6156066539970952	0.4611869423335466	0.4834125023221028
0.6750183337226887	0.4491217098923859	0.4991005491371706
0.5243473917706706	0.5134406601029698	0.3744614923428939
0.6943592345423270	0.4703748347230668	0.3204314902291437
0.6350248730575739	0.4829472277416780	0.3054300506331478
0.4693244414962071	0.3743739386214814	0.4611559311206204
0.4606021289007153	0.3137848358182661	0.4568615678253587
0.3090344750123262	0.5229999402686051	0.2996755891375587
0.4700544753588701	0.3165962896489518	0.2722828204326874
0.4803024535053120	0.3770883298713659	0.2772300985697930
0.3690847203073666	0.5119159648800806	0.2909954767857173
0.1285682219665987	0.5709846465775199	0.3894313867787905
0.4536693496493950	0.1235306502918308	0.3565906887121683
0.7342695276840874	0.4447962379317199	0.3792431549428454
0.7825511057328348	0.4253102865904501	0.5409268415103413
0.8414061403433594	0.4077968076497110	0.5293545041313230
0.8703223784910271	0.4169293052285150	0.4359367290659055
0.8403426233361639	0.4437773204569443	0.3547199558176285
0.7821265040415226	0.4608902273232840	0.3660369605087679
0.4627337976514183	0.2660572777844618	0.4053338933761605
0.4256202817123821	0.2098124587797796	0.2580334130221512
0.4238544690635614	0.1484512932196113	0.2732193771432619
0.4851089499848668	0.2203903787051584	0.4108183469758973
0.4843206097408463	0.1599565766205988	0.4248062443165954
0.2663227652355170	0.5474370325369313	0.3515848213446235
0.2321036542176011	0.5901932105792856	0.3605896591729346
0.1732674557249812	0.6022405916109490	0.3393279247626641
0.2025230267726064	0.5158884145071315	0.4833404599918539
0.1432444925827747	0.5277192060487279	0.4615823646219220
0.5368065949018134	0.7338634315070500	0.3954451675413193
0.5123050458277443	0.7789301209404714	0.3984010301553625
0.5122696742288364	0.8394104448643931	0.4123963424925548
0.5746579080103378	0.7900386086697938	0.2492345406608590
0.5755277971138618	0.8514063424939855	0.2644183307790367
0.5439295518369305	0.8760822243104220	0.3459696745715792
0.5973112850016294	0.4110820653772608	0.3891150696897501
0.3230802920783150	0.5540445391896579	0.4755314386151771
0.3866654082707082	0.5663445839829324	0.6224862281665989
0.3737327464826194	0.5069135477962241	0.7200251357650475
0.5656011264905803	0.3868217617174218	0.6295913054792334
0.3861740376581529	0.4348942055559486	0.6247201749689654
0.4328116618386872	0.3871509617640075	0.6213069699176772
0.4336163126132150	0.6135571749942600	0.6161922188868467
0.5665031701846889	0.6132364151251847	0.6228873012036090
0.6127925123395487	0.5656938518823728	0.6348534776232558
0.4843860079440686	0.6223953212057585	0.5240423896387628
0.4859927356782347	0.6832546296883458	0.5115129187149653
0.4810965395258106	0.4805051169834765	0.6277872190168189
0.5153567492506436	0.6939334842629638	0.6889559473996453
0.5141863132349335	0.6330693822686823	0.7007148071135914
0.6238624246007061	0.4868479088774407	0.7302428928842911
0.6851690749310514	0.4825780670262844	0.7339538651777917
0.5180037258357266	0.5199519610752750	0.6279784191451910
0.6897910871537057	0.5057404985096436	0.5533276680418593
0.6288582719258262	0.5113784769168908	0.5504401723601025
0.4839450841914252	0.3681821675723343	0.7069846293727465
0.4829383381262897	0.3071762218162045	0.6970574063473096
0.3102884988723406	0.5012168475478912	0.5375791352435032
0.5149430650803054	0.3160165866050522	0.5206976499576063
0.5165200313639495	0.3770124948745532	0.5314916555215952
0.3713617725641497	0.4960242527595444	0.5360646196040660
0.1227778271900946	0.5428567551432322	0.6345604641630377
0.4921221246303096	0.1214703367774427	0.6043651801324667
0.7353090926516000	0.4853797840297154	0.6007814916802502
0.7954920639544844	0.4679593190780248	0.7550406293048009
0.8563222231247745	0.4628392742020309	0.7361789509421874

0.8790453306447628	0.4789269232209664	0.6404183030366100
0.8409362936287944	0.4994612324818836	0.5640571919636330
0.7810788568036907	0.5038775316658417	0.5824515255411521
0.4983018779041645	0.2609141355685619	0.6441048225499431
0.4521103710615998	0.2058639537315142	0.5140474474135250
0.4515304114567596	0.1453281513005898	0.5349731272213575
0.5331618476922014	0.2185019173502937	0.6326423857037018
0.5329645293416454	0.1583329712214778	0.6528992794382273
0.2636131810731346	0.5158380340877954	0.5871439544509901
0.2250486507919164	0.5498891999607844	0.5840281604845940
0.1665652751139848	0.5653160343324817	0.5698440260877182
0.1978774611401141	0.4899670822907019	0.7299050035322893
0.1383196129619456	0.5047063416488261	0.7144663380374159
0.5011867392057479	0.7396920095936335	0.6338993317375554
0.4667284194478548	0.7826291473585452	0.6201029862958417
0.4674711202826347	0.8431006834217479	0.6375176702600934
0.5486121197340739	0.7925660442818546	0.5024472932942096
0.5498037347642609	0.8533772266242905	0.5206964554466162
0.5090995259593744	0.8787228229245697	0.5882774354088591
0.6123384248473968	0.4341157834600475	0.6379411835076133
0.3124594833186731	0.5106350006667825	0.7221721468674531
0.2775761988851935	0.5452098395327474	0.3905456441254695
0.5385085996252169	0.7233141180350353	0.3556901552627558
0.5285273822451425	0.4579064906866513	0.3782578516935530
0.4566589574631852	0.4720237800524134	0.3718256034231728
0.5453193093270298	0.5276481679645176	0.3744361035353616
0.4733372854757679	0.5417430809187745	0.3706058393590519
0.7227705576704886	0.4514402172515533	0.4164033852195300
0.4612165408106385	0.2764051969143149	0.3654069593979284
0.2726969286221497	0.5111536863076993	0.6272949303944059
0.5016057376839952	0.7276013588957264	0.5955079417315242
0.5346713866905735	0.4643083396694342	0.6310802116520757
0.4627833221246985	0.4630129779317945	0.6272185126652974
0.5363003578804577	0.5374619248636244	0.6287596925995761
0.4644327242828421	0.5361380558037265	0.6235276375012863
0.7262060970387053	0.4893693459590006	0.6411988598230773
0.4980263766921860	0.2725857351795588	0.6053307015180893
0.7324109698512332	0.4607328441848621	0.5127535580631725
0.4499401789963831	0.2653834559211315	0.2701933849765792
0.2651653689951684	0.5351454981531369	0.4859636161274735
0.5500571066828143	0.7339680036920621	0.2604354040885479
0.7397400963416244	0.4897127260341234	0.7374658089376103
0.4844555041903212	0.2629829336985463	0.5102259980351854
0.2580251221422452	0.5001488800469293	0.7210679088008622
0.5156340061991149	0.7360009086306500	0.5002896604887669

```
N-CENTERED TRIMER
   1.00000000000000
    39.6180019999999971    0.0000000000000000    0.0000000000000000
     0.0000000000000000   40.8536920000000023    0.0000000000000000
     0.0000000000000000    0.0000000000000000   28.1856720000000003
   C    H    N    O
   216   150    24    12
Direct
  0.5225955588454354  0.3931686757142159  0.3005520483042070
  0.4418399211824061  0.4448237548708620  0.2954912248028873
  0.4204905904522644  0.4961253435564936  0.2996214023497222
  0.3927643112076374  0.4735119468447412  0.2992068133035753
  0.4057385471986433  0.4423897515074318  0.2963128499448139
  0.5777835037177613  0.4963578614725297  0.3080541858466862
  0.5568234960592485  0.5477876969349915  0.3030783112528432
  0.5929310393411509  0.5499911148585532  0.3045167976920453
  0.6056546664127386  0.5187878858977915  0.3079065318216760
  0.4470181755455946  0.5516946810303038  0.3026691574551822
  0.4439022524159193  0.5872337352735018  0.3025821491656966
  0.4758633191031069  0.5992664523818845  0.3008045697620310
  0.5514851364410694  0.4406771418065110  0.3044824486759856
  0.5003606859078265  0.4215540005368175  0.2975975537463714
  0.5544336636170910  0.4051039226473952  0.3053519912812533
  0.4983094119450793  0.5709728543101061  0.3002331530331750
  0.5338659311224755  0.5739387668903853  0.3002566311682689
  0.4188706667078486  0.5304564163540110  0.3027219768904273
  0.4648734514759317  0.4187066314555637  0.2949972557137969
  0.5794306993037599  0.4619516429364616  0.3089459128198166
  0.5482116707720512  0.6077732456665652  0.2976918657605054
  0.5489972893969587  0.6248682937097455  0.2545558970748192
  0.5603377571084969  0.6571139648706869  0.2516808471478565
  0.5708280405741216  0.6734276914937862  0.2930488787530238
  0.5707135806015337  0.6563778259138052  0.3363742615072164
  0.5598239249883724  0.6239944916376299  0.3384333957728576
  0.6135205924613818  0.4472868016054443  0.3167013426033348
  0.6290464270756397  0.4499506887825255  0.3612589984503413
  0.6606201122172525  0.4363870038458086  0.3706558798724150
  0.6777660734294326  0.4196002176700205  0.3345765774013816
  0.6625573943708463  0.4166707643419345  0.2896980537603761
  0.6309152057112967  0.4302557547366362  0.2809878681184232
  0.4505453881309208  0.3848164048872221  0.2938808480693720
  0.4430648537808858  0.3682611790307926  0.3362085963522525
  0.4325173903750321  0.3357863705695222  0.3358145779837956
  0.4287761888430544  0.3189236047368147  0.2926368626803308
  0.4349359711557901  0.3355772543878648  0.2498501576738161
  0.4458859311011307  0.3680370709174905  0.2510319545018440
  0.3844174508984770  0.5450780888937415  0.3068793690140608
  0.3621053858770047  0.5456824347499973  0.2681924732208251
  0.3297276970401921  0.5585709240189721  0.2728270961349785
  0.3186505723416378  0.5710156996046610  0.3165657179655763
  0.3407248769986903  0.5709001754875246  0.3554194545176086
  0.3731868040252135  0.5581817007022303  0.3501445790334357
  0.1671082650877589  0.6285727968800352  0.3270817981303996
  0.7313746224184826  0.4059964640510576  0.3788764497126636
  0.7631428280930858  0.3864426988091338  0.3725222020603197
  0.7781074024507487  0.3730366240948520  0.4132396031537881
  0.8075582020862760  0.3545320091292958  0.4096853947107281
  0.8227234557428473  0.3495664893475173  0.3654880473781285
  0.8083378018183980  0.3632816355529359  0.3248318388380341
  0.7786619301985908  0.3815486041022738  0.3282701537843637
  0.4112319901027063  0.2649996563812893  0.2584514231151365
  0.4061568665190960  0.2296722161533050  0.2716155729569553
  0.3873588967308119  0.2104880575350430  0.2398344797330753
  0.3822849011725192  0.1772824081731279  0.2484621756206478
  0.4201977138590813  0.2148562454866138  0.3119973556314087
  0.4154205046152659  0.1814599358901141  0.3202409886288906
  0.3963944250381973  0.1626380689667603  0.2886803499320680
  0.2638264282976385  0.5851786688206982  0.3567871256732007
  0.2304867218455975  0.6007550413908783  0.3455456399579627
  0.2273965839478441  0.6270248233347078  0.3136885483169540
  0.1958656242697925  0.6409503856021820  0.3047049787160467
  0.2015719434809628  0.5888297829192282  0.3684551659201810
  0.1700472023393587  0.6024866474455617  0.3589380144142691
  0.5896211235325143  0.7257454306870791  0.2554768791925321
  0.5967455335010495  0.7611235176851379  0.2661885285465070
  0.5842143716121089  0.7777755140477698  0.3060381885598799
  0.5906869993182937  0.8111579092835709  0.3117912544989224
```

0.6158643325678691	0.7784061543890355	0.2325323211582417
0.6226815689065497	0.8115738438592864	0.2387512576333650
0.6099867827624034	0.8280928369203187	0.2783738343871899
0.5484537881203901	0.4069613531979338	0.4961464325523033
0.4572957813686334	0.4388672766463045	0.4969422791605241
0.4245005061416445	0.4841848781518296	0.4992492424391516
0.4027726448987060	0.4561149942639174	0.4996775211290203
0.4226661522937121	0.4286164309146921	0.4976141665084384
0.5776628143689705	0.5197042709745844	0.5040661897145681
0.5449194221381650	0.5649302840892395	0.4984066503830847
0.5794233182159926	0.5752812445010625	0.5016158663295476
0.5992459522340420	0.5478690891039247	0.5059056297033673
0.4371676379754830	0.5442123290150512	0.4977837510915016
0.4256531353505462	0.5780440063732674	0.4948651715231695
0.4538851132959056	0.5968703197162132	0.4927712950885401
0.5651144481943142	0.4596097180813732	0.5019390901876225
0.5198659890758591	0.4293986482372269	0.4965361344019212
0.5766464064254118	0.4257701522222752	0.4992079418616255
0.4824478583276172	0.5743954470088961	0.4942245840092988
0.5163364644775336	0.5851613421054705	0.4945343865026179
0.4147832019539340	0.5172955251849257	0.4997227444929139
0.4859759621953572	0.4185845300234662	0.4954373625511590
0.5874389279292641	0.4865882493369689	0.5052597480456070
0.5226030636878636	0.6211122791714422	0.4915024417954767
0.5155818564172301	0.6385007018641891	0.4496730097358003
0.5207021860505622	0.6721892954800958	0.4467054628871002
0.5326830755521543	0.6893546950900700	0.4863103661416355
0.5399184317311647	0.6723130416209275	0.5282453992393336
0.5350722182647190	0.6385976888486928	0.5306017912838572
0.6242463299543154	0.4796610562696333	0.5093897585325090
0.6370836214958959	0.4616983384325929	0.5479297902168900
0.6712362746479661	0.4538883231642705	0.5512076659564382
0.6935072191265749	0.4637938774045303	0.5153897169125103
0.6812815411563886	0.4823539255757742	0.4773229120913483
0.6471252238999520	0.4902291962676609	0.4745235102441588
0.4796499967999740	0.3825451264781345	0.4937647274057095
0.4664382227778913	0.3660558101588738	0.5333047693427366
0.4612956810613923	0.3323393585418285	0.5324979705688532
0.4689310131829961	0.3141828550958358	0.4916723131109364
0.4817241020362708	0.3303302248890422	0.4516374767400368
0.4871297166257146	0.3640570435155949	0.4530872156444732
0.3779005229081331	0.5244631842197484	0.5017894750195718
0.3564030930649078	0.5166331875690774	0.4638581972945184
0.3222340565686413	0.5246142061662257	0.4652461132329613
0.3085976382747046	0.5403568824049874	0.5050610172101765
0.3296117573244362	0.5479178795975695	0.5435163889742127
0.3638310033025419	0.5401619127029557	0.5414914414151650
0.1499980623932119	0.5784665188442830	0.5166317506938287
0.7491724566139580	0.4545900354590173	0.5532859702770366
0.7840811664407379	0.4415054545404786	0.5446817999616825
0.8020051592867583	0.4295932829476860	0.5840047544871165
0.8345753188588343	0.4172405394602581	0.5786096820315210
0.8498565687307756	0.4170418510567727	0.5339051755487724
0.8323677443272639	0.4292141769318689	0.4946416948577999
0.7996104936570921	0.4412645666283926	0.4998904157868383
0.4528929355657560	0.2602563672394482	0.4572521768950721
0.4515793761436765	0.2243775368568668	0.4686338797008293
0.4248444690562696	0.2058749911260224	0.4500093468511386
0.4230542371451848	0.1723021733425444	0.4584282525925147
0.4767285254768658	0.2086746266105558	0.4952708817060187
0.4752697371752376	0.1748999558419819	0.5027415736807428
0.4483909913421824	0.1566841034894396	0.4845557445046478
0.2519313487694644	0.5464362652069714	0.5416278922950054
0.2166863576350677	0.5578449184603582	0.5313759717504299
0.2099255563823572	0.5852778524996561	0.5029562078139667
0.1767056037032624	0.5956868380701159	0.4960447256240439
0.1897712241290474	0.5409240347446778	0.5524664354089432
0.1566018990772808	0.5509546203987250	0.5446769362541322
0.5496091669527428	0.7421561697300947	0.4496802008595482
0.5500994604640425	0.7783811030283999	0.4586045081534799
0.5236532309201195	0.7945813010050208	0.4819576180753774
0.5245331184784672	0.8285360578202786	0.4876104237861594
0.5774757882537930	0.7966036891839204	0.4413053571686077
0.5786378209796665	0.8303958545680603	0.4479202504646221
0.5520723203341483	0.8464762623012319	0.4708576462804533
0.5638081176881463	0.4166001420310338	0.6994756441023431
0.4683555500448764	0.4325103443196248	0.6967351473076993

0.4285473216788901	0.4722256579165524	0.6986483031005041
0.4116747380032921	0.4411821966110852	0.6975593880213107
0.4358289472989844	0.4170748839006485	0.6959048376080856
0.5718147969987782	0.5327958023636686	0.7013521638055552
0.5321199432148060	0.5725151012361408	0.6960195666133520
0.5646903539978330	0.5878803552907612	0.6957983788685480
0.5887526586108586	0.5637883144496548	0.6995069879154080
0.4302085044738841	0.5335590534714206	0.6976761750815694
0.4124494589500357	0.5648023570843014	0.6960709808272137
0.4366129361873211	0.5883544398387826	0.6935118568210140
0.5701704960657610	0.4714454751127886	0.7023521808912302
0.5314911645391061	0.4336200143453583	0.6979067112338038
0.5879399355859941	0.4401730746899230	0.7023044744250292
0.4689727223885783	0.5713501615366702	0.6937620874192065
0.5003608770159198	0.5878096029068238	0.6927704771971293
0.4131713798730412	0.5031969102128537	0.6991231756365612
0.5001501431930128	0.4171881430697889	0.6958169029006617
0.5871920028305042	0.5018333103443268	0.7033676933950892
0.5002399527439257	0.6241931671947202	0.6874362917817245
0.4902264601541943	0.6387964832365857	0.6445588335115739
0.4905611021519924	0.6726654925669014	0.6382595347152965
0.5008511755924879	0.6929582948783539	0.6756656306243709
0.5104244845371724	0.6787656098122101	0.7189967460146751
0.5102219574879070	0.6449041921132939	0.7246509632164613
0.6249748749347626	0.5009255105678186	0.7042494921594046
0.6430108755377626	0.4964018504369188	0.7465189020415274
0.6781128276485643	0.4932211167787903	0.7469594292045255
0.6961071129568317	0.4942499292999993	0.7039640160139838
0.6782908213372497	0.4995189789779251	0.6614713138720554
0.6433754719205528	0.5029434055502515	0.6618069663617043
0.5001777754618800	0.3807011349960886	0.6919001622127836
0.4904449028647610	0.3607874900706063	0.7301212424354554
0.4897399514148888	0.3268397416932996	0.7257113767245090
0.4985801200326770	0.3118279623647356	0.6826721006451268
0.5086680114204336	0.3313300696871294	0.6442981076924374
0.5094972278336382	0.3652878735071688	0.6493321576504500
0.3754058294422072	0.5040622548425193	0.7005083575184542
0.3562163464012865	0.5016859658781255	0.6588242245105262
0.3212465222441124	0.5050048723690054	0.6597875817226991
0.3043329855465743	0.5104673476644996	0.7029314910994759
0.3231410163488074	0.5116635136496754	0.7452075283930245
0.3581886740240630	0.5087004041909090	0.7434254678772304
0.1438880599022606	0.5450959448105519	0.7125603721112876
0.7546255809278755	0.4887711204232713	0.7364210634505292
0.7909467083424939	0.4861488103589766	0.7212013028961300
0.8139055024065595	0.4742697693457523	0.7550241796497248
0.8481774476641479	0.4719932881977136	0.7444579696554432
0.8601064798759831	0.4820094083822991	0.7000717352807897
0.8375117597551062	0.4941562213968176	0.6663563016430886
0.8030915809281771	0.4961158060208932	0.6767076220554638
0.4913167456169371	0.2573619558455701	0.6422534492661753
0.4925728744886682	0.2214819218326143	0.6534469820864276
0.4708612617219014	0.2004133099410407	0.6287012321357522
0.4711483140745156	0.1668942322959223	0.6379315681266517
0.5150620592454815	0.2082971446045206	0.6869102345772706
0.5155639391660282	0.1746795248768752	0.6957175102908434
0.4934776889188678	0.1539328899182508	0.6714543690684477
0.2468413339596561	0.5163412168337215	0.7384918016476354
0.2112155606743397	0.5260140010848839	0.7266232893263563
0.2021561592447411	0.5443093675139763	0.6864405912030851
0.1686601403202209	0.5539359555152432	0.6797145058315172
0.1861901867585947	0.5174669377730041	0.7596701831863022
0.1527345784683952	0.5266929163130847	0.7525119494974019
0.5063385947953383	0.7469223502743716	0.6331788718157737
0.5048592295349340	0.7829485733473800	0.6432803351839934
0.4831878541607588	0.7964073424949342	0.6775696962074736
0.4824757124240523	0.8301533908863851	0.6852415870497153
0.5255272208636149	0.8038579301591806	0.6165759216868726
0.5250636648292428	0.8375090327004282	0.6246869354028624
0.5035604090127199	0.8507491907577560	0.6590316509208278
0.5776855424834526	0.3912970608532130	0.3094671946316345
0.3324366984325340	0.5804313134474512	0.3895386648582135
0.4204035274477123	0.6009364708536629	0.3033877908851010
0.3902127569520820	0.5581782148469410	0.3806162052876793
0.5146514999630823	0.3677204765650374	0.2995462417944963
0.3663645852851717	0.4804932880014219	0.3010451304753413
0.3919348192236910	0.4194445785721458	0.2953375680636052

0.4837356402915823	0.6247377475096288	0.3000359472682025
0.6069482153665128	0.5727970716387960	0.3029091446371164
0.6319992018802484	0.5115537625663544	0.3093386064694400
0.5402586611886309	0.6126914524002400	0.2223145187244369
0.5610362650445914	0.6698407788006862	0.2178271311883062
0.4733534948806683	0.4872575574731647	0.2976844798958339
0.5785988126987683	0.6689148604760533	0.3687900366137527
0.5596933372980011	0.6112835562795674	0.3725079787399246
0.6156755273338336	0.4625071879008389	0.3898058202729353
0.6718614835809019	0.4385018003082650	0.4056798391567870
0.5249886033206534	0.5056161517103663	0.3042395385242543
0.6757397069127686	0.4037216882283599	0.2612063467804864
0.6195677755894667	0.4277741427857163	0.2458740347245819
0.4461476418549801	0.3808535925087250	0.3700928819785847
0.4279415253447369	0.3229757900285699	0.3692571941475941
0.3127102517261572	0.5587506761537220	0.2422726501420903
0.4314376071916372	0.3228811131286795	0.2163155880427585
0.4514050885879638	0.3804800898604175	0.2176152601041253
0.3702793090992469	0.5359122112581982	0.2340187132399522
0.1424276779090553	0.6393670201601546	0.3198760192351011
0.3927299283261291	0.1365339905849035	0.2952710690432961
0.7170922148423281	0.3901736163163128	0.3137296734325939
0.7661795591613834	0.3772802883675821	0.4475792681807249
0.8187573169355968	0.3440025901521893	0.4416198131735379
0.8458653434207536	0.3351737643878526	0.3627392113620146
0.8203707099999837	0.3599407984391987	0.2903252740100986
0.7683190090221719	0.3928180539663576	0.2962226751806691
0.4202775927232745	0.2759635707602866	0.3284984068507242
0.3771511041707388	0.2223303051039508	0.2082376868734233
0.3674272332322005	0.1626845040921941	0.2236202958400849
0.4355743039078205	0.2288735744745278	0.3370055726546620
0.4268939868617978	0.1701199880596955	0.3513839905479431
0.2752724587688878	0.5899075660259224	0.2869635649452686
0.2498172362235243	0.6374180565127053	0.2968510370951161
0.1937914677111823	0.6616375578464251	0.2803667346086625
0.2041804103402526	0.5687024983179984	0.3936242623701692
0.1476427216000642	0.5927457236407936	0.3765204468121653
0.5817660810349655	0.7169248454172554	0.3265771898305972
0.5688171882666473	0.7652218415445463	0.3325959572589602
0.5803186921578595	0.8239869572014851	0.3424841757726795
0.6249017193038706	0.7651387552333728	0.2014254287344129
0.6377703864356048	0.8246827267846196	0.2124726767985045
0.6149668904633863	0.8541904726583747	0.2830363865650711
0.6027237099080005	0.4175865208182485	0.4992893721331033
0.3192172028518963	0.5600047137493954	0.5747017331802092
0.3995516615316866	0.5861701329533788	0.4943891453219286
0.3801285531414060	0.5465927225243968	0.5713074570286324
0.5470393993296393	0.3804658190571847	0.4936478794865312
0.3754237674332697	0.4571709675425035	0.5011460650734680
0.4143905731074926	0.4033023188564435	0.4968509728407551
0.4553560699439022	0.6233718623480536	0.4904943748875684
0.5876922012344563	0.6005961809254011	0.5010773631344347
0.6264635988362904	0.5470056806511785	0.5098844878696575
0.5057416924344901	0.6253680399211130	0.4189002183415456
0.5152451832134446	0.6851957179750380	0.4138607786475071
0.4781244929625128	0.4870647353104914	0.4978055315765138
0.5487660349026154	0.6857546527245767	0.5593004587165737
0.5405490525459014	0.6256471698737525	0.5635975833318919
0.6198559951899445	0.4535285513932010	0.5757462716855078
0.6806461278782748	0.4398678173246599	0.5813039496483215
0.5241847111632266	0.5166881399339842	0.4986096047342323
0.6986509559814367	0.4901827611253414	0.4494742670329486
0.6378961465119140	0.5043651456371767	0.4443203733272924
0.4605095915033945	0.3798159284189910	0.5654519036524640
0.4518013620413290	0.3197542377900202	0.5639445397409912
0.3059911682014564	0.5191392628038468	0.4349880734426234
0.4876102503499676	0.3165229136585688	0.4196363993432131
0.4976008964414414	0.3763325864015414	0.4219663092099383
0.3667519472613867	0.5046453941386061	0.4324376524942771
0.1240290930067081	0.5865614575196452	0.5110333526076782
0.4472450107171310	0.1302886528476197	0.4904947738831482
0.7361665182521717	0.4443302804994136	0.4836763464525390
0.7899370578374298	0.4303131508647383	0.6187738135338948
0.8481507741162919	0.4079146648852972	0.6094885719780253
0.8754635651057540	0.4075867404446427	0.5297065488184597
0.8442740847872610	0.4294123880356412	0.4597115417267408
0.7865465248690111	0.4509469621177102	0.4687817074438317

0.4676993048084804	0.2694482576975356	0.5260573528763706
0.4055964284183481	0.2182415632222943	0.4288899659919900
0.4018897446792595	0.1581956654934327	0.4443442516366299
0.4978783819635970	0.2227262730325565	0.5094997534005834
0.4952758635817526	0.1628188910071163	0.5228020269950958
0.2646179656143656	0.5554091769114159	0.4718134853276513
0.2306605498588206	0.5989542251318992	0.4868353605693994
0.1717372014305572	0.6173791284956749	0.4745217386146677
0.1952999069713088	0.5198741429985044	0.5749987646734633
0.1358032222760922	0.5373694531961068	0.5607584166409010
0.5320697892248528	0.7351067555022709	0.5178424828499580
0.5019570203829739	0.7807404039593164	0.4949721430368005
0.5035645969213768	0.8409795534900325	0.5051110196740989
0.5977319591619997	0.7838310174684527	0.4226991003486539
0.6002853665698094	0.8443214987621680	0.4349506810903627
0.5527945178549515	0.8730086582142115	0.4754338077081937
0.6151283877552001	0.4369789560540494	0.7042533064770781
0.3102056898070614	0.5151219028660512	0.7788488674142801
0.3852324836742230	0.5680184220282052	0.6969270554956879
0.3725985733845706	0.5103948283917581	0.7763783725601850
0.5673584114425162	0.3902306480146393	0.6988538664686545
0.3844446785289512	0.4382035626511540	0.6976992585977932
0.4317700905629959	0.3908214541698661	0.6943254341516365
0.4330476553720707	0.6147055673438668	0.6920724527273678
0.5688498938356138	0.6140698426570107	0.6930635761461543
0.6159732225757590	0.5667405651254961	0.7005393857896038
0.4821342367407485	0.6231928515294721	0.6152255809652497
0.4831455266976786	0.6833064168210702	0.6044360642404085
0.4813975273486164	0.4829404343231455	0.6984104738977451
0.5182733025329757	0.6944263123676939	0.7484795465154112
0.5177825120774328	0.6342885662750803	0.7585729485932938
0.6292651117954722	0.4948773280169186	0.7800457226467865
0.6917288450183865	0.4896081544086863	0.7800342091885119
0.5189414272481448	0.5221596695356787	0.6993532757545303
0.6920652499398684	0.5001720043694604	0.6279206047327378
0.6300144926262347	0.5066268410573340	0.6283544975044085
0.4833639301078442	0.3721032918014577	0.7637718045132428
0.4819996648978039	0.3117866502937139	0.7559033881041883
0.3068298437082024	0.5041619016316264	0.6267477667429445
0.5155223672134127	0.3200118696967633	0.6106973201882347
0.5173923302648357	0.3802875679813237	0.6192432012520453
0.3689167507569789	0.4977759549650911	0.6249215564092675
0.1177344832352532	0.5526895565807750	0.7071772902760747
0.4937736663569408	0.1276529363519124	0.6784963915116285
0.7397686638226216	0.4859551767113466	0.6665257748367556
0.8041029288691135	0.4671766477249047	0.7897106150624211
0.8657172188353404	0.4625446186885087	0.7710212128757947
0.8870057280078192	0.4805185462824466	0.6918866030499713
0.8466790516529424	0.5021959398840721	0.6317406786563168
0.7859722423855947	0.5058869547914481	0.6498439751121027
0.4982349216879122	0.2657595616700278	0.7125490128103641
0.4537915011194670	0.2107023211504215	0.6022498203258256
0.4539002396926993	0.1507827418781560	0.6187705666714022
0.5330593207042169	0.2241558358220642	0.7052223429309067
0.5334167330327857	0.1646415151018699	0.7213861613221941
0.2597051639283222	0.5189093276936049	0.6677463868119392
0.2210796565963185	0.5517869774136939	0.6603875081046457
0.1620033681283793	0.5685507144722709	0.6486742368106938
0.1938318672999861	0.5037230920567296	0.7910761972584156
0.1334757213787574	0.5197270146685298	0.7783294000094030
0.5012604333126738	0.7394684187449032	0.7039985474958347
0.4659190826360527	0.7806613051478588	0.6974040954739247
0.4652205771442997	0.8403964540613824	0.7115433455175083
0.5419672725149925	0.7933507397444375	0.5895205197196905
0.5415222593168335	0.8534930729271700	0.6039836099466337
0.5030942552767663	0.8771294719024789	0.6651982501467812
0.5184585991259754	0.4501329532867835	0.2997034765192404
0.4494994135745730	0.4776812922206329	0.2971047947521093
0.5489209367574538	0.5149900390889995	0.3051971563791595
0.4802447190830516	0.5423264313385946	0.3012210250901260
0.7096031162468140	0.4047876524768478	0.3410058677238803
0.4199124898189503	0.2856226423972467	0.2951276537478893
0.2856933920092082	0.5841317815469662	0.3188855921362432
0.5806109088633183	0.7065521292992669	0.2936829888232737
0.5305847694514301	0.4612645145306030	0.4997798854545910
0.4570849952619683	0.4725451292753897	0.4978782461747125
0.5451617511314527	0.5312615601691006	0.5000263179829821

0.4717162415992791	0.5425603762356009	0.4971226838911463
0.7278752682991441	0.4540177481133668	0.5150921101398643
0.4646667120748995	0.2798012028083099	0.4932486227566860
0.2740675373746503	0.5492410503589988	0.5044005719117524
0.5364797004765877	0.7237964956303667	0.4860333729162710
0.5359197608931727	0.4668795689765612	0.6998878821557197
0.4626532577233793	0.4657160509821610	0.6983528365614533
0.5377243699094002	0.5393482488209043	0.6995085784264871
0.4645035724746000	0.5381218982921180	0.6964483579128632
0.7312679690609686	0.4899680013434552	0.7003097520409595
0.4978879492516835	0.2772678073794394	0.6805260739946818
0.2692167839375413	0.5155680528255441	0.7010636198528926
0.5010541101012963	0.7274790040324350	0.6723502821472741
0.7255797733806413	0.4214009488282555	0.4160169376677742
0.4076359273808595	0.2743114572423188	0.2169560494255799
0.2704938537044790	0.5740286298362911	0.3966510414964466
0.5919375925559710	0.7150902229346564	0.2144998877573080
0.7405637892946182	0.4650803224696341	0.5929366643011252
0.4436262294959929	0.2707370307023524	0.4179769990731800
0.2597682838740878	0.5350789128332514	0.5810418542155176
0.5606257158192255	0.7303464281408444	0.4121170055927860
0.7471181334593336	0.4898808434667475	0.7790501479898931
0.4845383797155981	0.2677532655157529	0.6019829782087164
0.2549195810336899	0.5097501643205886	0.7798379222226248
0.5122539312841753	0.7360477200547191	0.5929068417870742

```
N-CENTERED TETRAMER
   1.00000000000000
    40.5026200000000003    0.0000000000000000    0.0000000000000000
     0.0000000000000000   39.8320900000000009    0.0000000000000000
     0.0000000000000000    0.0000000000000000   31.4138799999999989
   C    O    N    H
   288    16    32   200
Direct
  0.6368161204216226  0.5098200486584024  0.2956634508040954
  0.4262085813003783  0.7913489818933147  0.2492592283045894
  0.4065207105137811  0.5523380990221585  0.2431689779042318
  0.4272992687624930  0.5790951816424854  0.2412580590169092
  0.7926737776022428  0.5533162402368083  0.2592683054295341
  0.6754542861149507  0.5308690004452615  0.2277000732418196
  0.6416972012504971  0.5258352377930130  0.2220968818404260
  0.5399113087988644  0.2506768371065514  0.2101684466775011
  0.5306898399677209  0.3075080694580481  0.2413684215858711
  0.5292602168343742  0.3258499457043905  0.2031682461679551
  0.8267519561334543  0.5557751665643351  0.2534669811655698
  0.1702328414880111  0.4676964895287718  0.2399508038793090
  0.3260424986407296  0.4670035716160074  0.2172480239589022
  0.5725592903360093  0.4777249378421078  0.2495825822309372
  0.3093450204017810  0.4737187428257116  0.2554701634858222
  0.5162295815480805  0.3769966503069116  0.2426737690748052
  0.4142621908730640  0.8239890577068703  0.2545349161991581
  0.8014849971721343  0.5318027419763233  0.3304237663407553
  0.5166327547079202  0.3581546815710389  0.2803924795089033
  0.5529005961990907  0.2162303573321201  0.2202591759751465
  0.5443421990336000  0.1904097130236709  0.1918832746500823
  0.5562989844821246  0.1579608485259692  0.1977871878905715
  0.5739626920777576  0.2090020273953219  0.2545283384430823
  0.3780452303841353  0.4849246838193557  0.2486610622220031
  0.5862865245582921  0.1765295637162796  0.2600332588768783
  0.1688346607488476  0.4231496005304831  0.2922616075258279
  0.5774445208413803  0.1509507173209811  0.2318453582755475
  0.4555803587190763  0.8091793601872687  0.1860530259805981
  0.7797164538630894  0.5411062194184161  0.2977477119211607
  0.2555585514302902  0.4583719429912492  0.2905500434484242
  0.2198479129639733  0.4534322834051627  0.2794996291024137
  0.4469530798591022  0.7836570029371408  0.2148226823724284
  0.3269792218158044  0.4866874673214768  0.2901415526748712
  0.3599074533802014  0.4724334884579165  0.2140153512680767
  0.2036086679472631  0.4730033921183193  0.2487466654591838
  0.3608601442992442  0.4922598833527017  0.2863517785749207
  0.5393582217679500  0.4347760025933308  0.2453309490415475
  0.6901214779624072  0.5257567836481526  0.2676787393263106
  0.4595035918859458  0.7489905753425322  0.2050483404535769
  0.1528354592079562  0.4426332846933368  0.2615136599971262
  0.7433663798632981  0.5378988265541080  0.3062644273803444
  0.8355175228846874  0.5339285816280595  0.3244166299558471
  0.5239360571766519  0.3239974025656647  0.2799613720147086
  0.5223184767732079  0.3601390430958533  0.2042992935015278
  0.5934318846422590  0.4479077101338469  0.2499623180019424
  0.2020585244690319  0.4286760860739889  0.3014589541613569
  0.4607093665108856  0.5654392282164400  0.2416927652776063
  0.4440053266569500  0.8417979902958875  0.1917341547064418
  0.4891409204892789  0.5860231914246874  0.2414044352810356
  0.4146061671881943  0.4895385580816841  0.2459894526889792
  0.5109673591904995  0.4142006350123024  0.2436106298170581
  0.8482373260298541  0.5458662448352941  0.2859187852794335
  0.4774760848560210  0.6768455525115147  0.2758018730591840
  0.4231739357086672  0.8492700136755137  0.2259530563462497
  0.5727435086176507  0.4211216922407151  0.2471324265806943
  0.4781505811688294  0.4255970465124403  0.2437046374515318
  0.4337168476768934  0.4600412178375478  0.2452173853341391
  0.4849608036813757  0.6427355720541695  0.2767767588088437
  0.6216857167688218  0.5153462260842128  0.2560804510872861
  0.5852594362203591  0.5107370326562360  0.2509428980070919
  0.6705376183831899  0.5150068262569280  0.3018441572007887
  0.4838587101148821  0.6231798324502377  0.2396581256152925
  0.4760813866565394  0.6392917044598315  0.2012575583138259
  0.4690251818625392  0.6735360195867687  0.1995603786689511
  0.4691210928769173  0.6925894086994623  0.2372414860576645
  0.4220644349108160  0.4260179744900016  0.2458645666711755
  0.4490325775353396  0.4051285836911003  0.2445188592532651
  0.5662308506988831  0.5402086487459491  0.2483402323845839
  0.5219284349915333  0.5746482510398273  0.2436675525049735
```

0.5510559766171552	0.5950915495237820	0.2450192802239428
0.5779342806266555	0.5742104259122437	0.2482745651415420
0.4273652008486045	0.5225318320460201	0.2446822973599616
0.6352884286120815	0.5322995848388169	0.4641042374946874
0.3751987695260570	0.7745743700389773	0.4101101046289455
0.3984981072509358	0.5329372281750878	0.4160980517353340
0.4133163424551264	0.5635298606084936	0.4149034061274615
0.7829884293586037	0.5953183774081848	0.4173681396306033
0.6645520025923359	0.5665498285355373	0.3968340872377289
0.6314527854381664	0.5572918553985314	0.3944947002839995
0.5791108440721749	0.2615467337793685	0.3818623164259357
0.5657539894196092	0.3169389514934295	0.4133422278580394
0.5703997026044766	0.3383763344931507	0.3784904664004663
0.8163512234309691	0.6028753403908684	0.4116674942236635
0.1770190843059802	0.4293501076932070	0.4162087741527294
0.3366964606779173	0.4354199395074971	0.3851534986650727
0.5753708261042400	0.4932966929109976	0.4229253177747034
0.3172088059672835	0.4415963012731979	0.4211627891334624
0.5403034641807505	0.3829005607305465	0.4151499608168125
0.3595563767215187	0.8054949486310908	0.4158357342449416
0.7932935289235233	0.5838238818071986	0.4918464487742740
0.5353459197566725	0.3608917114877652	0.4494598119820990
0.5968220685386035	0.2291974126435239	0.3906254425002608
0.5833486919350931	0.1993584302724431	0.3744778371029523
0.5994518538333795	0.1688488283457842	0.3808911401993772
0.6269038277377491	0.2281082605908703	0.4127213382082142
0.3840069848258680	0.4616878781922654	0.4194309393281718
0.6433733260640404	0.1976051967536451	0.4182190763188861
0.1818789108938669	0.3766707208719991	0.4539954069354561
0.6296167126915940	0.1679248984030860	0.4025520232114762
0.4183636803653122	0.8019943154258058	0.3696311307027913
0.7711495956760888	0.5856886498817860	0.4575614034080465
0.2662709847777366	0.4173430137573190	0.4523344727411227
0.2302344506336788	0.4117165406954672	0.4438583889360366
0.4046683738049658	0.7726019764677833	0.3867770055656168
0.3312354844014236	0.4575615770288090	0.4565280815479964
0.3697152706718406	0.4452372798063392	0.3844728306355396
0.2107857090937015	0.4350394680097481	0.4216640556957458
0.3641789678875417	0.4675767531693708	0.4553534235463840
0.5516727514793592	0.4442503184654810	0.4184734695138049
0.6833163299131046	0.5586415737445214	0.4329561846210824
0.4216466531562986	0.7398533931446512	0.3779504515851639
0.1625445782889518	0.4000951713274722	0.4321142354216735
0.7354306626996315	0.5783743515900537	0.4664676793960685
0.8266674010315020	0.5909784902716645	0.4859768880358733
0.5480569699603194	0.3284002019860650	0.4487957827096410
0.5579733525508060	0.3709826243218329	0.3797410266190310
0.6018853273046821	0.4685262065376319	0.4225507087789889
0.2154339567993025	0.3826277607573010	0.4602891140230724
0.4487952004909552	0.5572786933546283	0.4153128243139017
0.4030965237517889	0.8329216120267502	0.3762376995381354
0.4725372894410932	0.5833523929672605	0.4153688565608882
0.4190760875311770	0.4732038703292379	0.4183750195291350
0.5279834504365787	0.4181938463489111	0.4166723954671477
0.8382804200209958	0.6005944258881860	0.4458721657952728
0.4516283985108224	0.6731835679217441	0.4464170927306581
0.3735325950949351	0.8347208041921997	0.3991426929019666
0.5871063879507847	0.4379690767019787	0.4200276000153624
0.4935311747791294	0.4227619139170825	0.4168835178462978
0.4434856185833614	0.4480661474183489	0.4178795498113235
0.4643337437308574	0.6407116736368104	0.4476856799756210
0.6162588226488739	0.5396819762668058	0.4279011274517768
0.5812595118706235	0.5282877291864015	0.4246004420782807
0.6683669629767353	0.5416924798455383	0.4669414182240856
0.4603083130155903	0.6186469732664011	0.4132146795307937
0.4435766682525433	0.6305419812967598	0.3770372896619273
0.4311508919329216	0.6631343551451112	0.3751852723804264
0.4348649814797139	0.6846285879171893	0.4101880562775209
0.4383470226523784	0.4124367209502144	0.4173501939577834
0.4687051233600346	0.3970944701067406	0.4161601551698116
0.5568860925537327	0.5534375992335562	0.4226821059603827
0.5069372216737759	0.5787548293318806	0.4180994310071404
0.5317513667657144	0.6044139207684969	0.4195105325111104
0.5620075792836750	0.5890715131678048	0.4229160989031244
0.4249942964012349	0.5081862759357340	0.4175757665475020
0.6264805955743097	0.5575268627741936	0.6249257547793593
0.3290029974124447	0.7468861087289709	0.5714138097394459

0.3928238187131047	0.5133804100490583	0.5787561356267490
0.4021213521408196	0.5461399861783537	0.5774338491876290
0.7610213781977097	0.6444753710385560	0.5744474831487720
0.6486220692819548	0.5948960511133419	0.5557913964019661
0.6176625479591926	0.5797119857785729	0.5545700138780801
0.6191149496333290	0.2805108323954962	0.5422692965460360
0.5956428583664719	0.3320619288245530	0.5748663378004402
0.5960090235298514	0.3542233353562266	0.5402427195042240
0.7927747704650681	0.6572337036116828	0.5683615995099391
0.1939678015414619	0.3679281805516846	0.5731269104047007
0.3498482722408539	0.4052885797059062	0.5476662134028109
0.5740489759787268	0.5071998790793714	0.5858429380846695
0.3287462380237137	0.4087109130934624	0.5827470148807403
0.5589929631905732	0.3923053547355327	0.5780621055004697
0.3075974867416096	0.7740175088007182	0.5769722059892055
0.7733828614832682	0.6351899721397022	0.6488903984293399
0.5584897380842471	0.3695831224767630	0.6122197210951189
0.6422366448999576	0.2518623046063779	0.5507640101200186
0.6354236421085390	0.2207600884823152	0.5317425624906068
0.6565760371859183	0.1935903490575450	0.5379015566937577
0.6708629084814485	0.2554995757720341	0.5755156449324897
0.3910382158236145	0.4405651378762527	0.5821495770496490
0.6923542160967754	0.2284108181803770	0.5809334189175592
0.2084074858927163	0.3184194670240845	0.6136590396251379
0.6851880256003707	0.1973890924686230	0.5623672497448909
0.3652983433873421	0.7819986463231149	0.5292813634543369
0.7510217325970384	0.6333357357969122	0.6148414601880839
0.2823662250902830	0.3764093477071139	0.6138070279932011
0.2486674824106472	0.3628511469266218	0.6039565414109557
0.3579965898115655	0.7506825644786959	0.5474262846809933
0.3388903703372033	0.4279576557128855	0.6177230612410938
0.3806412979407311	0.4208519013092742	0.5476049269776222
0.2256068802168751	0.3808664820544632	0.5797015891205092
0.3695696072072292	0.4437877293157840	0.6170532186810619
0.5592370324634374	0.4546543267693509	0.5819815538619324
0.6687892011111795	0.5915133641227672	0.5917936313666570
0.3809365120252883	0.7219006545194706	0.5388921060737838
0.1853648205746027	0.3366417931834293	0.5898607970901264
0.7169441357339631	0.6204032497460016	0.6241987215493358
0.8052047671060845	0.6475573832714591	0.6426354974274973
0.5766783202980036	0.3399097398694247	0.6108352517690661
0.5780041395225385	0.3840008057843336	0.5421706993188969
0.6044407515153907	0.4875887254723986	0.5860332247503860
0.2397416992210883	0.3316330291683657	0.6211338908885464
0.4381099799957811	0.5463556449525040	0.5779633362866604
0.3442144870257703	0.8092070718577755	0.5355973621527562
0.4568918029526491	0.5762860824799915	0.5776350509190721
0.4234917118380692	0.4582712727731799	0.5814257219350013
0.5405323716634571	0.4247185711503842	0.5800031471737906
0.8149682737005549	0.6586953920902868	0.6023574459724902
0.4216434493576351	0.6614759219716146	0.6078573164864632
0.3152271181220600	0.8052421560574514	0.5592800870129061
0.5952001248926264	0.4548467657881120	0.5838723101464828
0.5058470930272555	0.4227491250207653	0.5800766613912923
0.4520297955091067	0.4381080687795397	0.5810368250099539
0.4391741986651040	0.6314413950161669	0.6095395126927892
0.6061235884803815	0.5604400262677262	0.5889092951556060
0.5737260322869230	0.5427090358730077	0.5866133408181667
0.6573473208872012	0.5729286752267033	0.6266942221676761
0.4387473938946252	0.6088254311046013	0.5752621031258346
0.4204535487702575	0.6176180162667819	0.5389382810597332
0.4030595528755538	0.6477594471504075	0.5367349199438505
0.4033705811547156	0.6698017133497041	0.5714788993679477
0.4535037060679226	0.4021401072825317	0.5807951671088635
0.4861457762282865	0.3928278605207778	0.5795812687143487
0.5452319847113302	0.5628331598492075	0.5843897204217774
0.4915407130884503	0.5782212981014418	0.5800412146850228
0.5112649115201351	0.6081352854296107	0.5807374789610861
0.5438038890758886	0.5987917142185072	0.5839595366218082
0.4231999330990858	0.4937791679133720	0.5805165710668259
0.6145865511282970	0.5818637811080500	0.7998280124768827
0.3005571432964234	0.7235114511223385	0.7441577905609736
0.3936374750323884	0.4946299948716564	0.7544348126789379
0.3970545012385834	0.5285487577124430	0.7528794465696846
0.7357612988918053	0.6834126760237065	0.7381243904782887
0.6350161737425922	0.6133625701479900	0.7254862841787272
0.6062164947153484	0.5943475371483078	0.7257738286195050

0.6501392267675374	0.3006775816193272	0.7063514764224111
0.6199230551010633	0.3479580450111312	0.7426316370414761
0.6149961658999427	0.3697104105363022	0.7081443114746460
0.7627655117375745	0.7041508472359050	0.7295852531656386
0.2217760022683886	0.3189646190515757	0.7355872461230042
0.3639619693034879	0.3886814118072639	0.7185938587271679
0.5729333180091388	0.5198298436991425	0.7609256160900109
0.3477398190058718	0.3810925417745289	0.7569610893451121
0.5774002678532656	0.4037849565597587	0.7510446136121637
0.2783818964687125	0.7496055336689561	0.7520636122284874
0.7544833248832358	0.6772113127882834	0.8107998550836775
0.5828308568778928	0.3819242869511343	0.7853895616347160
0.6743460163079379	0.2733606112272738	0.7166160870378470
0.6720423489751360	0.2429392536267949	0.6943451207624349
0.6941107089226748	0.2169342552229060	0.7028241162859995
0.6995290727317698	0.2775378316703926	0.7469146540313251
0.4043191019206424	0.4227249174463569	0.7564258960293011
0.7218380768656321	0.2515996029458434	0.7549845276886894
0.2312795994881633	0.2857019822529517	0.7987955974083759
0.7190422115954851	0.2211848437121434	0.7331442896225774
0.3282386002177079	0.7578010228758946	0.6914389985612442
0.7313411733807705	0.6696926192197550	0.7788991995145050
0.3035866153338432	0.3447059126188246	0.7876150372110142
0.2717538818638090	0.3272625524183541	0.7768616968942161
0.3257565673974877	0.7274545732888245	0.7138417875637835
0.3603713288841048	0.3940084464037667	0.7953005228721922
0.3919341393014790	0.4090050194352798	0.7185325543800546
0.2518650612610748	0.3354101105820842	0.7417656672290255
0.3881575256326273	0.4146788061479227	0.7945412445412361
0.5674772906259761	0.4654803668387582	0.7568620543150459
0.6541439571519267	0.6167624990787234	0.7626007411653756
0.3497945776932930	0.6999583046648710	0.7036606500542069
0.2114646533613796	0.2940547823811374	0.7639082910832886
0.7024779523290301	0.6481628501936978	0.7919144281638384
0.7815264940461288	0.6977162941281514	0.8021643285791139
0.6038404365114429	0.3544309323124171	0.7813595290047242
0.5940493364785873	0.3972665770994259	0.7127324815364208
0.6062758101901748	0.5059757369060113	0.7605226485202772
0.2610638420565307	0.3023803764969035	0.8053750985217323
0.4324499071032098	0.5352522025285543	0.7530819301381255
0.3063391237494081	0.7839792481248217	0.6997967376282503
0.4458276030155227	0.5680229957448669	0.7526617967948959
0.4333482462238055	0.4459727309607359	0.7561083179673672
0.5541331021546814	0.4327752963272184	0.7546146288335884
0.7857243382283540	0.7113239481840826	0.7614851318224836
0.3962518891393532	0.6465619359365692	0.7788429867544355
0.2813580237063500	0.7799460293386353	0.7300906511083475
0.6028494694071496	0.4720935924394009	0.7580293105014015
0.5202965837752006	0.4248750751533651	0.7548936977462888
0.4647735948176153	0.4310801939887934	0.7558128863552644
0.4173251094727119	0.6191389455020567	0.7829966992832962
0.5957381244610148	0.5776843328792090	0.7627575991698031
0.5665459938407009	0.5547321292559778	0.7616531189565441
0.6433058769771802	0.6012214593970557	0.8002976141764017
0.4226126072190370	0.5970087517779132	0.7488696716980879
0.4056798796929476	0.6032247427751896	0.7106623304667172
0.3846782295855559	0.6307289713888901	0.7059390344669372
0.3799506809137226	0.6527523464988856	0.7401934831374557
0.4719583797546589	0.3958488935431262	0.7544082397493597
0.5056611561933146	0.3920771335325349	0.7534412468191983
0.5351217832548034	0.5696378685076744	0.7596787497652776
0.4796464016218838	0.5759048216444310	0.7552931395096236
0.4943291529597816	0.6087006861301123	0.7557043742441970
0.5279529940028161	0.6048766095759082	0.7588145204482303
0.4269707367241279	0.4808370122349913	0.7554988968174985
0.7318243390708008	0.5410399392546452	0.3427067726677373
0.4685888364260991	0.7415103050593728	0.1686319296854949
0.5301862594106350	0.2575872604208815	0.1738444657153520
0.2663108103469233	0.4537970805875844	0.3271045839092062
0.7235926298550103	0.5827712630739376	0.5026498287265641
0.4353747241845747	0.7343977148826042	0.3432084088518865
0.5649723664121939	0.2665500126478311	0.3472813268741097
0.2792216511361053	0.4082838503160415	0.4863893345136657
0.7047208369748882	0.6231312365676100	0.6604152767153070
0.3954836677733286	0.7190014708129550	0.5041845429208200
0.6047876942887193	0.2834755519283300	0.5074132752467561
0.2954194534854807	0.3719094433256695	0.6491922473291117

0.6965089834940715	0.6429556603167824	0.8300242247206508
0.3608369687111243	0.6955824553008499	0.6672069357533970
0.6389795350187284	0.3048645444077378	0.6699150138962149
0.3148640364562583	0.3436527534635212	0.8242294799027895
0.4601564303157534	0.7267302947793407	0.2386500275394659
0.5398418598099456	0.4691855546288332	0.2466483467699005
0.4676427994880810	0.4584802531654130	0.2439405197526619
0.5323643892642375	0.5417754494761450	0.2455092437046948
0.4601585589676245	0.5310465987542361	0.2436113417391055
0.7246134402074993	0.5307231128616364	0.2710763942001166
0.2749372802793308	0.4678328212548195	0.2566671155876641
0.5396642638697154	0.2733870859471669	0.2432761309710806
0.4209921673657736	0.7172496891759748	0.4108588367966358
0.5451181297680174	0.4779970797807039	0.4202788047546017
0.4771159966479132	0.4530212687531827	0.4177482009539326
0.5233124578995627	0.5484931128160904	0.4199653395182777
0.4553021945838238	0.5235225090941511	0.4169830856403374
0.7173958974124991	0.5668350977073303	0.4327441587898411
0.2833198134174585	0.4326818639119774	0.4200983556368626
0.5797203364149119	0.2843445107245269	0.4145294553828611
0.3844838917547059	0.6997348676079035	0.5719090793125925
0.5469125763940773	0.4866907808407479	0.5833839878567212
0.4841635235113401	0.4493609620241790	0.5807830644728657
0.5131399883756081	0.5515993272738536	0.5821566520586137
0.4503765813081226	0.5143116844574093	0.5799262131114955
0.7008239929498425	0.6057822775603297	0.5907196430744345
0.2971882913350002	0.3934827102790289	0.5810794591199516
0.6150423137608846	0.3024520563893260	0.5754267112485343
0.3584755122793664	0.6806624907450508	0.7381857845053049
0.5497609422976278	0.4947777450876448	0.7588490066509769
0.4946738622700950	0.4475192303987444	0.7562811807557045
0.5052221478993654	0.5532278310657545	0.7577380110057639
0.4501509290893881	0.5059532542477674	0.7548480985817964
0.6835599302991007	0.6354143723384433	0.7593081833314957
0.3188546623901393	0.3615121974140248	0.7546953634654795
0.6414401645574401	0.3200721214043318	0.7407695583781072
0.3796938935243421	0.5525654780780533	0.2432480081501018
0.3744320612144825	0.5022213079846514	0.3135994563997434
0.5791782870712211	0.3946503383492994	0.2462412775783892
0.3962493683702794	0.4187779333933901	0.2471453120237381
0.4492428596962296	0.3778760931005063	0.2444115029595534
0.4208813108578635	0.6055474075627758	0.2396425037654729
0.5509206155333705	0.6223273081266111	0.2438272546085369
0.6037293824028096	0.5814210074964459	0.2502795945953453
0.4752201829008916	0.6246135866376904	0.1719048954444362
0.4633188616312287	0.6856224851370767	0.1694199779083794
0.4827228841184046	0.4790844491000863	0.2440547848631546
0.4773222688711898	0.6913990098346187	0.3052903794031719
0.4911063220601382	0.6309076933718883	0.3071087840353777
0.6217577555056594	0.5014451440114688	0.3224744284863519
0.6816025315834385	0.5110284575352593	0.3329882869822247
0.5172555024640912	0.5211948774801125	0.2455256619487454
0.6905845514937775	0.5391133342267906	0.2008250902401966
0.6306355045918788	0.5300894676892817	0.1908661282272115
0.5116888911585356	0.3705597962778914	0.3107131372518858
0.5251898807825993	0.3099791107346396	0.3098224780026655
0.3124131389637083	0.4571234863069832	0.1899305102855594
0.5337078586137300	0.3131730071631133	0.1730679266625822
0.5219690763746915	0.3742426460828758	0.1744801837184048
0.3725732975127122	0.4668725433480152	0.1841622492819999
0.1267650103681501	0.4384056192743501	0.2545077681708595
0.5871597002614055	0.1256078116303987	0.2362704781619943
0.7372403462260719	0.5289211824310943	0.2431749560072024
0.7912212837333403	0.5227939913166216	0.3604302253459957
0.8521860854439254	0.5262959924525742	0.3499528970570774
0.8748931119428524	0.5476133191857484	0.2812611838362093
0.8365395785922477	0.5656177500259301	0.2235926783812592
0.7762791370821135	0.5620028205857353	0.2339102100865457
0.5454164265441134	0.2647892316430759	0.2730032265646539
0.5283599831655541	0.1966461189514010	0.1650616527403025
0.5493059803212966	0.1380740965735152	0.1756176217667031
0.5814457783468896	0.2285849663440383	0.2769137314233112
0.6030390014387017	0.1713323095171517	0.2864434785385209
0.2638873037954091	0.4660527785071212	0.2276580283620160
0.2167392208645152	0.4932292348236567	0.2324329830049132
0.1577392372583576	0.4833327530699705	0.2163904442941186
0.2146714020650008	0.4138422281562879	0.3256979744577962

0.1553349218991629	0.4035812754615788	0.3092739017082289
0.4550090430961982	0.7357884922527205	0.2683381022593109
0.4186658958789934	0.7719892782447573	0.2719245634022466
0.3977359192427201	0.8295504088783201	0.2810635061421278
0.4713242908042668	0.8026012452351882	0.1591298205354981
0.4510637829159265	0.8614597797253527	0.1692809290210520
0.4137640212771080	0.8747463620365533	0.2302165380425868
0.6202206528757878	0.4477092226758251	0.2518322633752126
0.3143461743560137	0.4919710897846217	0.3199705055442384
0.3722084908209380	0.5275609667967248	0.4160715641949427
0.3748433055443701	0.4803002557909464	0.4829011166306488
0.5989661863867173	0.4135234632147908	0.4192082699638410
0.4143198138676639	0.4003843667306186	0.4175090974119123
0.4737658954890392	0.3703566645997395	0.4150391140964468
0.4014779850910257	0.5879814380472599	0.4138797724973898
0.5267496771323029	0.6311558624171922	0.4182247675229811
0.5859570923737594	0.6011544574593637	0.4250846147184943
0.4401056712796438	0.6138325407767248	0.3498516244758680
0.4183673717798437	0.6718597315632789	0.3467602955713102
0.4884110292209720	0.4759906776576290	0.4182488665783242
0.4540033318441907	0.6896790809225757	0.4739890050927859
0.4769622618111664	0.6320094849614974	0.4763348435488950
0.6239143031292135	0.5187898937391296	0.4905555653475905
0.6826316044638517	0.5358162783401029	0.4953513604884120
0.5120235571923366	0.5255221368262368	0.4193604742751257
0.6762597186727302	0.5794305232718688	0.3701502672736481
0.6172823050132535	0.5631962753619763	0.3658965818550908
0.5219531836915360	0.3696394921621316	0.4775060394199495
0.5449203907434167	0.3119087453257472	0.4762378663770350
0.3256747900215756	0.4233624644911450	0.3573805701996193
0.5839270891555810	0.3295909835883845	0.3506641867378538
0.5622056572402964	0.3876926780081368	0.3527336101482261
0.3845669755084234	0.4405363574213138	0.3560852536978905
0.1361676651704784	0.3956141918297350	0.4276616299461579
0.6424428171787494	0.1440651900168380	0.4070287652652571
0.7290041158858420	0.5631071242172970	0.4041460542965684
0.7837832484770423	0.5767946158690817	0.5230892305919526
0.8436510719741012	0.5892388745283452	0.5128761599210674
0.8643832062700695	0.6064495446508839	0.4413159939859927
0.8252068731890363	0.6106200872149867	0.3803474982240159
0.7661917789688871	0.5975761962521969	0.3903241279800803
0.5883599922260916	0.2767678115640472	0.4436825598189297
0.5601474731628319	0.2005023169601443	0.3568769110864056
0.5885414331480288	0.1456843715319716	0.3687300085946685
0.6377883929700984	0.2512205673995342	0.4250808805250040
0.6670379746429954	0.1971061910570856	0.4348413497579188
0.2719031222806811	0.4355460909873554	0.3912154570903581
0.2218844970727689	0.4580109019932760	0.4091952160641968
0.1620059243508087	0.4478908981062191	0.3994639037811137
0.2306287806422082	0.3648661703188510	0.4780472423607978
0.1707041992381468	0.3537327295555967	0.4664340615812876
0.4126414131194688	0.7251923966664882	0.4399829782370508
0.3641361447066999	0.7518519258324160	0.4233424980187990
0.3363731175823044	0.8066852723132284	0.4334948693819654
0.4410969920889583	0.8001612495353903	0.3511330909479656
0.4142183292117524	0.8557200500541234	0.3633124968746399
0.3613592282772460	0.8589063528451165	0.4038255689862836
0.6281583710905728	0.4738442825183594	0.4240188015596515
0.3163475001141169	0.4621908205716429	0.4848018918659319
0.3678780188096586	0.5033815584494353	0.5785922660762606
0.3770586098791441	0.4591195028391404	0.6441964587099505
0.6111150284907467	0.4329020804651179	0.5835180836995262
0.4321127527043937	0.3857456213326160	0.5813444112743534
0.4960092015289810	0.3675063871601417	0.5788095808161029
0.3862286434516861	0.5680801601477139	0.5761985012075309
0.5014979578988391	0.6334682474642950	0.5791946057269719
0.5651840514589450	0.6151472556482530	0.5857082523439403
0.4198880745142123	0.6004617045735205	0.5118389132903702
0.3891902878989693	0.6541749728425394	0.5081656491178143
0.4908864129910103	0.4741178703728230	0.5810583025350018
0.4214828966419529	0.6783917697590433	0.6351791134036227
0.4530528002874604	0.6251256524840094	0.6382298080544818
0.6180337162985117	0.5426892142872149	0.6520462191798556
0.6726857590999753	0.5703788831486063	0.6549603005219351
0.5063882659808904	0.5268502161285828	0.5819672646241713
0.6576531779199739	0.6089293087184647	0.5283196128599820
0.6024494851273980	0.5821636920613971	0.5260548935365719

0.5440767628754428	0.3755334109231288	0.6406042971290798
0.5767343103011032	0.3228904021308505	0.6380588761726378
0.3416864387490670	0.3908711594569980	0.5200606688144180
0.6104135079063675	0.3481964197408082	0.5119837495950733
0.5787194612656487	0.4012988948172713	0.5152215856810478
0.3966766870071482	0.4182626881803234	0.5198644137522797
0.1606667508107660	0.3264816844050845	0.5845096769286162
0.7019549765961711	0.1761964322638284	0.5667861822788990
0.7128153781618793	0.6036537079206085	0.5620874239869508
0.7652783677307952	0.6268697116752787	0.6802741719657149
0.8223839026473855	0.6486711109835617	0.6693859441720919
0.8398268642012737	0.6686100981281509	0.5974927747920321
0.8001800578782320	0.6660522192051105	0.5368766582830281
0.7440120412114103	0.6437023692151468	0.5474989832845333
0.6245579255044800	0.2960349408762911	0.6045300595861587
0.6133630327559010	0.2182875481860535	0.5120627775199401
0.6508184521930953	0.1694016350215983	0.5233295894536100
0.6767343707044223	0.2796743079771520	0.5900704527016760
0.7147890189550175	0.2316207533442808	0.5997621980763744
0.2864649465734102	0.3926600172653689	0.5515286994448869
0.2321656415653602	0.4053304755193569	0.5664759014111602
0.1760285127194947	0.3824011256758637	0.5548078415956094
0.2577205506108332	0.3179929597474123	0.6403753766674058
0.2018614562335136	0.2938731682175960	0.6266471447234789
0.3748463951499720	0.7061536088606953	0.6009357738505244
0.3227878818139782	0.7225147894557128	0.5851842164404020
0.2848763284211864	0.7706855468852073	0.5951853090387361
0.3876479344854490	0.7846198591385936	0.5101783973720113
0.3503331960061216	0.8335882160128142	0.5218027045691135
0.2985263133892875	0.8264735257630519	0.5637853321746589
0.6293627560344702	0.4975715268581349	0.5876116372863148
0.3227272438972041	0.4306493521269328	0.6452230279115547
0.3709237069288853	0.4801178208081739	0.7549868546846676
0.3974463682636538	0.4249701246557115	0.8244643947620910
0.6222678337417561	0.4532856690999639	0.7570827946504155
0.4533339158529235	0.3762555462759831	0.7539111713170735
0.5194691965972147	0.3687546275844242	0.7519856068479928
0.3776140227869631	0.5473343896166343	0.7520495628542970
0.4806321198327238	0.6320755572335350	0.7539348340967162
0.5465355746999709	0.6244792933002377	0.7602439369325887
0.4092556283697384	0.5862266114936884	0.6837837704828187
0.3722768889661080	0.6352917284688789	0.6757626257100467
0.4975789891506450	0.4730256379715771	0.7568021300080032
0.3925446794478412	0.6635229679180797	0.8058022933824839
0.4298903581292964	0.6147194116548700	0.8132244658854679
0.6068266143550340	0.5693130664330445	0.8290824514156435
0.6575870070008322	0.6042577780886725	0.8293437054488230
0.5023001506225577	0.5277204563489271	0.7573284793883635
0.6433463472424558	0.6251702429437110	0.6960026198976483
0.5919859920441363	0.5916836518793654	0.6964509214014271
0.5704002314310951	0.3865808843325596	0.8156545543927448
0.6076711013712482	0.3376544889005703	0.8084803683797910
0.3541378186552701	0.3789753394008997	0.6886142317097291
0.6272284720501203	0.3649029608081619	0.6779170372093798
0.5903411612291697	0.4140831334877700	0.6856944164806247
0.4041667511851106	0.4146619483374032	0.6884089648182549
0.1879271956512515	0.2812618441047351	0.7589137983433727
0.7364012769008925	0.2008451255172435	0.7395975562157643
0.6916395630957609	0.6390003030223136	0.7288135919897801
0.7504139034718962	0.6666794469025424	0.8424364055981566
0.7993593592730109	0.7032391583049975	0.8272851861289465
0.8067889041557006	0.7275857246476531	0.7546996560069847
0.7658152580927068	0.7147250791881463	0.6977635849217706
0.7182547712279714	0.6783734378556464	0.7124657904176788
0.6488735664195479	0.3110654037324916	0.7695291014843569
0.6527518867819093	0.2399948299884174	0.6703985413078321
0.6919054178444707	0.1932448015143940	0.6855751064979294
0.7024466269436214	0.3015134021420610	0.7634047976474552
0.7415727855145769	0.2552527483308060	0.7781984245326899
0.3093858631316894	0.3585893261535561	0.7247891839440941
0.2592413825366536	0.3548981602103553	0.7191847545168646
0.2063091693424957	0.3258353018877445	0.7085006924269874
0.2766507322541273	0.2967918649135433	0.8328091894572255
0.2233597166654446	0.2662495595962831	0.8210638734862276
0.3511889223604945	0.6898653569861437	0.7669134798638273
0.2975200858578645	0.6996075046215552	0.7607897965176507
0.2586094523996797	0.7461314261495916	0.7752670699537101

0.3475778916397098	0.7605603915498977	0.6675222790817575
0.3087485411436432	0.8076243973344116	0.6824988350866948
0.2640924725911246	0.8003913502676366	0.7364339033640710
0.6290186713814986	0.5203889667419079	0.7618584992489555
0.3483793257448435	0.3877669328851605	0.8252263175096053

```
N-CENTERED PENTAMER
   1.00000000000000
     40.4128619999999970    0.0000000000000000    0.0000000000000000
      0.0000000000000000   40.3914600000000021    0.0000000000000000
      0.0000000000000000    0.0000000000000000   38.2582340000000016
   C    H    N    O
   360   250    40    20
Direct
  0.1801304699407043  0.5808475462299890  0.2187421507456912
  0.1971060956837054  0.5326360105819103  0.2680067218237177
  0.1639378794854923  0.5388637344531066  0.2604716372826625
  0.5451109140031845  0.7454166106122665  0.1916129168574217
  0.5446625500698596  0.7817662265910520  0.2000648980716093
  0.5285294513776245  0.7954544609156952  0.2291634697337226
  0.5278325166215853  0.8297510979240166  0.2338580158632993
  0.5601225973702845  0.8029634422827738  0.1759828437639821
  0.5598071032690499  0.8371142088105769  0.1810134670539685
  0.5435204905437375  0.8506287270224707  0.2099404592043053
  0.5443120906350478  0.4021955639110999  0.2251410086066438
  0.4565983066769437  0.4383271008433643  0.2199918509977980
  0.4261407920961035  0.4853459539359749  0.2206353050697306
  0.4038080927123011  0.4577174339667490  0.2208473900247377
  0.4223074871296159  0.4291906664086080  0.2202085483384150
  0.5776806856110720  0.5149789325324063  0.2273086885350920
  0.5474285218402044  0.5620082123252426  0.2231711976781423
  0.5817080348737939  0.5710807304243487  0.2247565510531756
  0.6000549008708967  0.5425642712414602  0.2276032290895065
  0.4409082898949435  0.5455218028136252  0.2215822081545118
  0.4311095058274867  0.5802342230706534  0.2210236489395879
  0.4596009959416003  0.5981038930336275  0.2201763689477370
  0.5628654647150761  0.4548034133265668  0.2278064158266359
  0.5173248643618467  0.4261309401067241  0.2236391612859417
  0.5726936586940756  0.4200731692786127  0.2278826898035741
  0.4866298879446666  0.5741654842810743  0.2203600111294470
  0.5203020232680449  0.5836890512282482  0.2208684441904555
  0.4179190804519223  0.5191764283185868  0.2217852641135807
  0.4837466179384431  0.4166249185992621  0.2211754022495277
  0.5858034079691780  0.4811623585782475  0.2292931971377327
  0.5276766383248925  0.6199732468500421  0.2196240330568929
  0.5250619389005032  0.6377743999852645  0.1882657330240885
  0.5298039088174570  0.6719521607647753  0.1869551889527408
  0.5368393350940613  0.6894479193464136  0.2178527647335348
  0.5401580882513194  0.6717981978147991  0.2493251251627880
  0.5358965619493921  0.6375913998172071  0.2500219097083784
  0.6217054755503097  0.4730014457789827  0.2335435742952079
  0.6342396830363041  0.4630702635452070  0.2661111770318881
  0.6677561637195776  0.4562122954574989  0.2710628806247615
  0.6896845042818338  0.4589621334501384  0.2428344174842035
  0.6774324373430037  0.4683154397171182  0.2099404912126123
  0.6439480113967970  0.4753392793170746  0.2054591830428643
  0.4760906803520366  0.3803735946968601  0.2209447234692093
  0.4758285890219351  0.3622196474848469  0.2522142039000884
  0.4703286753324716  0.3282291424294760  0.2522931410516935
  0.4647895490634618  0.3112244522014816  0.2207472598386181
  0.4640066927931558  0.3292259905289704  0.1892860153944619
  0.4696958259158872  0.3633136649612887  0.1897752383260504
  0.3818574418026353  0.5271017356085225  0.2240896472487982
  0.3606095533037439  0.5223278900270805  0.1954894613151774
  0.3267989691466208  0.5283628450981257  0.1983937263286895
  0.3132130246546982  0.5391412416887392  0.2301914830405917
  0.3341400561872103  0.5445318522367539  0.2588394243817925
  0.3680045754356937  0.5386489355921024  0.2554666391544829
  0.1553652035833081  0.5629466268524961  0.2358294982746345
  0.7442596624617152  0.4530096108359208  0.2741365640332702
  0.7796135196663529  0.4437285531140318  0.2669861868045793
  0.7975756207424062  0.4287973575371323  0.2941213083891458
  0.8304212495855228  0.4194200139203723  0.2890474448526829
  0.8459307786606041  0.4253146705480940  0.2569876548731115
  0.8284411328936222  0.4407271843270346  0.2300132879025601
  0.7954422316710622  0.4497802553150442  0.2349241466465656
  0.4528149934972723  0.2550597926338584  0.1962165917448020
  0.4544775525791833  0.2188133972391322  0.2050551089580303
  0.4369487278566406  0.1971710465209035  0.1830873989728295
  0.4382216250492117  0.1630836048278325  0.1884059869778650
  0.4736037542410558  0.2056136460528223  0.2323290761605448
  0.4751974934924248  0.1713848640925115  0.2373093416835896
  0.4574862464323626  0.1500701487892399  0.2154878495682546
```

0.2574605528946441	0.5423953922595627	0.2593454871800514
0.2221282258390335	0.5500658323174235	0.2505088372080639
0.2133672607066678	0.5743582528330555	0.2259099516390719
0.1739409613499267	0.5447228397168206	0.3612398921493041
0.1931549549698195	0.4887196212197074	0.3989888968520232
0.1596865904440065	0.4951875686483491	0.3934299025182659
0.5092784997838119	0.7493204577996199	0.3339626851106815
0.5054956177770628	0.7855610023113389	0.3415899488811199
0.4787879530434034	0.7982296201729721	0.3608253149070642
0.4756122699404832	0.8323362346638633	0.3658296514711059
0.5290696902877374	0.8074398962242684	0.3277609713361908
0.5262610653439830	0.8413992555234187	0.3335092301263241
0.4994005633497443	0.8539479505825870	0.3523834120488323
0.5631608814303146	0.4133001478818017	0.3697282541306744
0.4697766642437671	0.4304888133834499	0.3658376603658618
0.4308667757730330	0.4708185448441762	0.3656097974355153
0.4142585444454949	0.4394330095778879	0.3652786162514954
0.4378673752483098	0.4149774663022806	0.3649175545006237
0.5726077129946033	0.5307779670126352	0.3695070610879022
0.5337560571807292	0.5709917287014759	0.3656561658939929
0.5656102952074701	0.5865709267896816	0.3668523237786119
0.5891524850422702	0.5621961346030158	0.3696613365237386
0.4331957637910940	0.5328754697231874	0.3646254669390960
0.4163294852199593	0.5647444403307624	0.3629436534605353
0.4404086997341525	0.5882056363042802	0.3621375844807997
0.5703038181778444	0.4686912402807404	0.3706043266659020
0.5317678246540815	0.4309678997703628	0.3677544737112037
0.5871809067522656	0.4367942896529684	0.3711897292619430
0.4718378061881734	0.5705149295444026	0.3629865740677263
0.5028082682620145	0.5867958746151433	0.3633138897937863
0.4160852157569623	0.5023575701618074	0.3654436713039418
0.5008140418895450	0.4146651055939422	0.3663022209504793
0.5873761795449863	0.4992694243671253	0.3713859650547439
0.5030033531283339	0.6237182102763130	0.3618808689812394
0.4919046096177683	0.6407634957173038	0.3320920428889104
0.4914219412273045	0.6752503934016421	0.3308547068126990
0.5017431447831315	0.6935910230385198	0.3599845567376164
0.5128496160851478	0.6769438496483202	0.3899047433477302
0.5136574551877242	0.6424872437842049	0.3906431743806407
0.6241970112863420	0.4982301496801330	0.3739479531613337
0.6398957484749638	0.4850224640651797	0.4035965883902876
0.6742447602132416	0.4828172157869804	0.4058216991948568
0.6938377009263590	0.4937198696964605	0.3778944699265255
0.6785700520827548	0.5073240366912712	0.3482984261608941
0.6442340809009685	0.5096476325910021	0.3464831813829677
0.5007789074487445	0.3777438191137480	0.3655997061687477
0.4873192799206127	0.3592984257562052	0.3932636691987280
0.4882588073489895	0.3248234455897602	0.3930996775625847
0.5023671637711602	0.3078521705995725	0.3648650371002236
0.5156196679128970	0.3258684788994465	0.3368682772513925
0.5149516153068542	0.3603486167457334	0.3375139438531918
0.3791552051419800	0.5034070236886754	0.3660552788180153
0.3603564512128055	0.4924487749654794	0.3374493820328405
0.3259274955329523	0.4942755148966383	0.3378395257618079
0.3094028576915130	0.5068956938898159	0.3671532785502822
0.3277798822809894	0.5175477512885321	0.3960817719283210
0.3622137952320221	0.5159276917554788	0.3952701216365882
0.1499862085737718	0.5232892876039447	0.3746763201016130
0.7495822121722310	0.4940996388521011	0.4054155931692411
0.7856930445563215	0.4889088730498996	0.3979332922688436
0.8058216931664390	0.4779726415755260	0.4255052723242805
0.8396414143090793	0.4732195349862980	0.4204385094378358
0.8538909464950527	0.4798629163612669	0.3878817922052150
0.8341052434625637	0.4911238972641589	0.3603969517542787
0.8001557649318226	0.4954794758499573	0.3653004886846024
0.4959542204966023	0.2521728787623718	0.3388497042163218
0.5002673090567654	0.2159343403454704	0.3461422997778102
0.4775527248800793	0.1937089103660683	0.3313582579984331
0.4810529088731666	0.1597495638879501	0.3366484381148322
0.5268126484061988	0.2036191074645845	0.3658726633699871
0.5306989585886354	0.1695312643575649	0.3704315023229375
0.5077586388256154	0.1475593631118277	0.3560300320406566
0.2530358660466028	0.5024544828429595	0.3925800520960190
0.2173507756891780	0.5098968872855031	0.3851505656993423
0.2075056783280531	0.5379398722849873	0.3661601570807401
0.1679722536983171	0.4817240625340091	0.4918934719693803
0.1986089557712341	0.4324016658053422	0.5315148259386141

0.1646274331379571	0.4310870368003274	0.5254658785251219
0.4637416141538229	0.7474417929005225	0.4676023819236843
0.4516471307466172	0.7819316786428935	0.4745805225774232
0.4235571062938959	0.7884105240527695	0.4950325540761657
0.4123524978285051	0.8208669571831220	0.4993280865886895
0.4684290393999884	0.8083884616721623	0.4587596593326698
0.4575853449494373	0.8408103656778769	0.4636875142492371
0.4293978013431519	0.8471247604476420	0.4838315330046474
0.5741012987892479	0.4250781182762349	0.5025782014227573
0.4791295208775602	0.4272041607780599	0.5010671630966737
0.4341250150442553	0.4605903010230352	0.5013485050113258
0.4228893194306713	0.4269107035179315	0.5015112793931206
0.4501897768086696	0.4066644543365550	0.5008891667338075
0.5648416076670382	0.5424627437888375	0.5032684651096097
0.5198199302583546	0.5757392447644909	0.4999835968568639
0.5487403073770843	0.5963198409209721	0.5005876816889375
0.5760100468874075	0.5761553136866729	0.5030695421028685
0.4263359076874289	0.5221405825483231	0.5003379429232913
0.4046630665956252	0.5509567338517255	0.4989449421325824
0.4247630506397471	0.5779134510821813	0.4979346529161331
0.5725068504355457	0.4808915190517527	0.5041006241862106
0.5402946276206566	0.4376189004285426	0.5016063437431637
0.5941745532217163	0.4520279021817191	0.5038873103551466
0.4586086022922342	0.5653840415616482	0.4983083124922419
0.4866759315085471	0.5863326722722438	0.4980940957081346
0.4143353205139729	0.4892674085597987	0.5011655375635046
0.5122997353493993	0.4166226688082776	0.5007302259197588
0.5845734594483041	0.5137733769006101	0.5048300701410423
0.4809246581841944	0.6228024359869619	0.4963669420421363
0.4664448008949115	0.6374492765034562	0.4667723672880019
0.4601064944955013	0.6713336749936052	0.4653356190734197
0.4678379355864110	0.6914596358944641	0.4940274989740245
0.4824720676747878	0.6772669435002280	0.5236585132200484
0.4890875495283649	0.6434379593222125	0.5246164703171206
0.6211475258886007	0.5185199611207905	0.5068551392915088
0.6391085806856914	0.5076223724484015	0.5361293064604444
0.6734199178103070	0.5106664919934344	0.5378151257185849
0.6907663134493671	0.5245741110436275	0.5097047667481109
0.6731866392169695	0.5358286087336276	0.4804463752752182
0.6388519557108594	0.5329841227726014	0.4792056161383520
0.5182323297053134	0.3801576700885537	0.4995779922500179
0.5097705080108194	0.3596706597872724	0.5278414103567237
0.5166569527424136	0.3258721121369070	0.5272276812705005
0.5318765965369207	0.3116224040162224	0.4979611976293080
0.5399486320552032	0.3316294035206469	0.4692827836078111
0.5333121767279586	0.3654528557809489	0.4703557573233175
0.3777671112976073	0.4840258154214382	0.5011795516709444
0.3618959911271463	0.4694789263892484	0.4723676966572752
0.3277331401582234	0.4649416156332462	0.4719357035681538
0.3085593323387743	0.4745201517746296	0.5007201825874734
0.3240288859189638	0.4888260565717821	0.5298080495310069
0.3581790245506213	0.4936410921680141	0.5297664010202250
0.1491836465020844	0.4558592644254871	0.5058053329356694
0.7457187012100316	0.5345508777119279	0.5366551113562555
0.7822476853924920	0.5351144645788160	0.5294168196052258
0.8036540286515622	0.5300367379154548	0.5578075821940659
0.8378436530956027	0.5309010757659210	0.5531393575226459
0.8511181214426248	0.5373613163749115	0.5200876757327723
0.8299992540364339	0.5427424829724923	0.4917375865469934
0.7957461145826527	0.5414648967434504	0.4962725718554848
0.5362564455135684	0.2557231303074197	0.4716760468273392
0.5483014342713365	0.2212211192850003	0.4785070927521082
0.5314324743510679	0.1948571090344073	0.4626179556245757
0.5422777129212557	0.1624103377543342	0.4673008017034173
0.5764821111861331	0.2146495346750931	0.4987780396200724
0.5876965829788476	0.1821736894636940	0.5028077780378790
0.5705599926431061	0.1560015958967293	0.4872576319142009
0.2540070600761192	0.4589024643788695	0.5253155924169841
0.2176910370611128	0.4580799362416920	0.5172523491286110
0.2021429541372279	0.4826976362356810	0.4972548397307406
0.1757757117049292	0.4234970354277398	0.6232576300907261
0.2143550077588709	0.3806730446838409	0.6635482252122784
0.1812613335500425	0.3731689824511978	0.6570365578800689
0.4210991776972998	0.7359160537624023	0.6003025626445350
0.4036498429279552	0.7679741371421653	0.6075465207505479
0.3746447738354930	0.7696246010522677	0.6276506766600127
0.3585311640365014	0.7998689641533442	0.6324698102257578

0.4162858639908134	0.7970095177065476	0.5924817114790562
0.4004967561100815	0.8272622345232908	0.5978801362589820
0.3714964254133672	0.8287494489500354	0.6177787149497790
0.5864234719525840	0.4384093196240220	0.6349454407246751
0.4926189332889718	0.4231182756209282	0.6327490889136393
0.4422691065850963	0.4476991823456559	0.6329834977977890
0.4373645873054334	0.4125393081276203	0.6328767273797712
0.4679039482644593	0.3976275381266030	0.6323035333224957
0.5558280747887753	0.5521178493174561	0.6350665630088204
0.5055253548674602	0.5766515230327212	0.6321589009076111
0.5302236268039545	0.6021542783553613	0.6327300165439272
0.5607096000675040	0.5872787384919810	0.6349612441221224
0.4234473570291023	0.5068178266103535	0.6326466514175016
0.3968750728121218	0.5312049135538015	0.6316202843203872
0.4117049355571704	0.5613846803392669	0.6307387871065189
0.5746470145680509	0.4929914970849904	0.6361058480850210
0.5508952724369990	0.4445595976158302	0.6337518703239634
0.6012358990490445	0.4685855261161715	0.6361305912198285
0.4472582234784024	0.5552419695832189	0.6308682321905429
0.4709980310909049	0.5809692490400601	0.6305581848938027
0.4176073781667692	0.4722987176440352	0.6331211006507539
0.5271785922427881	0.4188274553726820	0.6326884902896838
0.5804642507895069	0.5275301900734652	0.6365801244744236
0.4587073077136796	0.6157760559487280	0.6289452109824308
0.4420793433275578	0.6277474074259345	0.5992733808208975
0.4300239755666718	0.6600507850211332	0.5978118006517912
0.4341129124475023	0.6811672785524802	0.6265381794323636
0.4507729725549044	0.6696368042356284	0.6562739872640255
0.4629967524927414	0.6374179757182761	0.6573040903114462
0.6154977945710995	0.5390481738549278	0.6381046180881911
0.6354659943619637	0.5322193023916933	0.6672924355119187
0.6685753240132690	0.5417781304690247	0.6684109288433314
0.6826443127661606	0.5582802250026240	0.6398228162793794
0.6629690661114335	0.5655149252802606	0.6106563533111217
0.6298033785511141	0.5561812397688823	0.6099749109175513
0.5396090737517200	0.3840646925681410	0.6316273350365593
0.5344884387095308	0.3623620570101216	0.6597717939737545
0.5469777747133504	0.3302233795261453	0.6591569407779448
0.5647692338624898	0.3189116123581058	0.6300775749895610
0.5696851020709028	0.3401004744205623	0.6015599446332226
0.5573272434465762	0.3722829196354074	0.6025789782430842
0.3825460180920336	0.4606630057518442	0.6333043676900265
0.3689985047851256	0.4442440177806761	0.6042972456439940
0.3360065419892350	0.4342708825563003	0.6040001886468414
0.3157362356193832	0.4401193614569301	0.6330810466622095
0.3289826781302052	0.4561379014114804	0.6624210784302154
0.3619136389577344	0.4663918534283066	0.6622297091546038
0.1618230289973011	0.3946921800028369	0.6370628120096711
0.7345567112870708	0.5795121642625172	0.6659444062844243
0.7702692265707397	0.5869319315000217	0.6583483777663893
0.7923251232212067	0.5871166400523925	0.6866847501119620
0.8256721070546387	0.5944913863273554	0.6816257033461104
0.8373515866978851	0.6022553109172760	0.6482198860535755
0.8155202745077544	0.6024185605499064	0.6199038218460423
0.7821849695897631	0.5946439132305311	0.6248462657663103
0.5786964054982727	0.2644318724946241	0.6040623248271231
0.5965124848126840	0.2326007949557318	0.6113598761624434
0.5842474328648184	0.2034023751357207	0.5963179696019114
0.6004654391069293	0.1733788420646018	0.6016953290928020
0.6255531914833172	0.2313664967575533	0.6314339645586953
0.6421236973801974	0.2013603853316440	0.6361855326706394
0.6295445670062629	0.1723072555009552	0.6214977876564406
0.2641052923488239	0.4167123709788059	0.6579679531160753
0.2286263629317246	0.4093532254035145	0.6493755190540341
0.2091448825075074	0.4306639265331505	0.6290055178877825
0.1911995543771536	0.3748940631614868	0.7571775996068855
0.2249811839027976	0.3535759295467214	0.8166199648841104
0.1929170061473279	0.3424562287751257	0.8102631581654099
0.3871646149030916	0.7215643884902082	0.7351687528793955
0.3700920871132595	0.7534811518886777	0.7439069269512000
0.3452772906990699	0.7556363448990944	0.7695242680512270
0.3299237668406089	0.7858767039459275	0.7765195284747990
0.3790501781314559	0.7820058668572540	0.7252683222453437
0.3639732499935248	0.8122533028090165	0.7326081834221360
0.3393867720564735	0.8142728419714518	0.7582306505106248
0.5941056906704344	0.4533314559976268	0.7786661622262869
0.5042622107843742	0.4224557424003993	0.7781004397718828

0.4506684987335914	0.4387473085671555	0.7788030816609171
0.4513736240115697	0.4032610009242416	0.7783080368752523
0.4838857151222933	0.3933571099437098	0.7775385422009156
0.5447311067846869	0.5602867746854552	0.7788686362414032
0.4911621386100322	0.5765328151011659	0.7762609111870067
0.5115301947139035	0.6056343909398062	0.7759995094953266
0.5440112231453547	0.5957648243542987	0.7779193100495002
0.4223213072095209	0.4939422769667468	0.7777633940624782
0.3919164527234741	0.5133356413260467	0.7763711480872751
0.4013014353023708	0.5456147700382457	0.7748358340340036
0.5730920042054214	0.5050722115398432	0.7801283801223109
0.5580434672824167	0.4532598281313188	0.7782875698921090
0.6034982294619443	0.4856215714578451	0.7797207570801771
0.4373726329313543	0.5457310129096260	0.7752670394737392
0.4563963205324900	0.5750824859658662	0.7745985116245189
0.4223703977929358	0.4589600627320459	0.7786998971351223
0.5390445955153887	0.4239075006661328	0.7774392438981504
0.5730477921435631	0.5400880511885550	0.7800999228738320
0.4387484357912969	0.6074814912763399	0.7716476835433594
0.4232813572640078	0.6169099965353250	0.7403241227060869
0.4077468001886819	0.6475617023267075	0.7366309484068478
0.4072210093599247	0.6696576759182042	0.7648700738271604
0.4219865353729564	0.6603141556645902	0.7965931441109891
0.4375799192206088	0.6297309286619748	0.7998335316865000
0.6058741115547912	0.5571719511496325	0.7800096792349794
0.6257941729626940	0.5584202887038645	0.8099994997165763
0.6572673759912048	0.5726414888340776	0.8095820699204720
0.6698407917482110	0.5858403251691591	0.7782560873078891
0.6498583458751881	0.5851803798844435	0.7481655060184138
0.6183679215954714	0.5713103062878219	0.7491937481455617
0.5567728582071904	0.3915330162260154	0.7749033399199667
0.5574779259756268	0.3691846067087209	0.8030077672069578
0.5731769864963061	0.3386318817782603	0.7999135214156802
0.5885129575978635	0.3294760463840699	0.7684379023514183
0.5884180832217335	0.3516698089134207	0.7402823228404563
0.5727772084514211	0.3822642168622493	0.7438266129090573
0.3895561245901881	0.4417928837113653	0.7787726794409436
0.3760148086567041	0.4290217861837784	0.7478076284254808
0.3446775706685078	0.4146954671799883	0.7474066813051602
0.3260276266932164	0.4123157644895970	0.7783188043209497
0.3396511239513142	0.4242293691326585	0.8097165891408308
0.3708962030064143	0.4389002978774815	0.8095189277813497
0.1758700422844429	0.3532136191361711	0.7805524723379125
0.7226881235265720	0.6094196290750139	0.8012310649140786
0.7538723697886721	0.6267243002689444	0.7898642857751577
0.7793732396371318	0.6289625487339277	0.8146915822470330
0.8086879867820830	0.6455286568516657	0.8068771431021919
0.8128248734669247	0.6604770570775941	0.7741924015614015
0.7875117067749106	0.6586102601118595	0.7494196263436188
0.7582163002931976	0.6417732796739820	0.7571121802801591
0.6091615184277904	0.2778254096195388	0.7387889680342532
0.6267714273958835	0.2461853023702792	0.7474171662458603
0.6184533994249206	0.2175949111037926	0.7285664680755736
0.6340955275357093	0.1876103774289294	0.7357931843848177
0.6515449098149253	0.2443949882805012	0.7731109720566373
0.6674505155934253	0.2144255316262210	0.7800052723033021
0.6585941500141864	0.1859350582753500	0.7615343784579328
0.2747842211419850	0.3868061016815736	0.8028682646258217
0.2407018577456592	0.3751299018584817	0.7930822052123726
0.2235523376591527	0.3856655391300294	0.7631819247707281
0.4058612419831468	0.5894909317117546	0.2210725393248018
0.3841079246339155	0.5427485613544570	0.2779773042545456
0.5416286091810174	0.3754544716510373	0.2239443489289106
0.3770387622650251	0.4597478274862788	0.2215683768542097
0.4134267775883634	0.4038277056410519	0.2203554783012267
0.4623166739933952	0.6248556062914939	0.2195397428280667
0.5906889490138212	0.5963959351094570	0.2238944823595478
0.6267719706510761	0.5404866653403705	0.2295401710614133
0.5188815852758417	0.6245793560625255	0.1642119200218093
0.5279532488205709	0.6852376991343528	0.1623219850351157
0.4787195200512591	0.4864022105985089	0.2206814416145512
0.5453278452204822	0.6852166132435833	0.2735180106454649
0.5382537000554969	0.6244081148636138	0.2748102841427698
0.6173494533137770	0.4607830726033293	0.2882631965193114
0.6769360959383449	0.4489883468639206	0.2966781095081117
0.5251543670550143	0.5140166020012985	0.2242724553486590
0.6943294695921333	0.4703466862136894	0.1877108714700802

0.6348475725220463	0.4827371343712596	0.1797502123299842
0.4801283213047081	0.3749652629031668	0.2769465490282167
0.4707889239103555	0.3144624334841776	0.2768994406045979
0.3106568208646893	0.5243332817076779	0.1758436197939963
0.4591814156781010	0.3163538361673179	0.1648602051619056
0.4695822285176783	0.3768989332067282	0.1650817684588182
0.3707589352934471	0.5136839240666665	0.1706696681916250
0.1293725351086834	0.5679001164119816	0.2300750573470321
0.4588329962299850	0.1233259944872269	0.2194578934759874
0.7349339640527011	0.4450889434510406	0.2226833720855687
0.7852854867356996	0.4246014966065277	0.3191228831566512
0.8440088865346799	0.4075057664784414	0.3102599483794647
0.8716854828833988	0.4180541939898597	0.2530595608846387
0.8405672679766784	0.4458918915208765	0.2051068733536733
0.7824678404086972	0.4625651544279736	0.2137867542555118
0.4653665554038714	0.2668040011558439	0.2468790908566658
0.4226576276329881	0.2078584651774666	0.1616801540279524
0.4243286611873691	0.1465467438526806	0.1712417620959337
0.4879091581542310	0.2216756403263922	0.2496589950754731
0.4905227653582510	0.1614107810638442	0.2583106299592226
0.2676102548522870	0.5474185290272764	0.2077243218609953
0.2325162838592036	0.5890628347954582	0.2130269049137671
0.1735659394241656	0.6000750777230075	0.1999272288228856
0.2040383373507094	0.5141444814711318	0.2874564013473397
0.1446709118403548	0.5248823456770920	0.2739715587181236
0.5384616221289630	0.7341063705331274	0.2437492924604098
0.5158871343083931	0.7796959926154434	0.2481694465855391
0.5147994315863118	0.8401280361070096	0.2563186782383977
0.5720615364074754	0.7918950324438985	0.1532265988928574
0.5721006848189835	0.8533015121548007	0.1621941255021820
0.5429091009437926	0.8774233348514565	0.2137317131547243
0.5979079595027957	0.4108371281461779	0.2294363939430346
0.3239317564063701	0.5528529003783976	0.2836474821875851
0.3897284275557266	0.5685558852354957	0.3625313186068070
0.3763578212884226	0.5245884387546034	0.4178000835915183
0.5663412939267193	0.3866062697749879	0.3699552785767522
0.3875942004898982	0.4362913256310068	0.3649982424286767
0.4338143156720192	0.3884363267940724	0.3640968241156813
0.4373149429533225	0.6148956057712865	0.3610843146840400
0.5696494982645665	0.6131092041702885	0.3658229071772792
0.6157486385616097	0.5654242003759434	0.3715014175805087
0.4834115826886767	0.6266989365710051	0.3094313508873346
0.4829282029183062	0.6879522638473314	0.3074067919006681
0.4825175672861336	0.4816574717218772	0.3664957348265802
0.5202421150503707	0.6911911563808825	0.4128572513584133
0.5220942191983898	0.6298551243954362	0.4142388324548479
0.6248104248985176	0.4761751795270429	0.4253505723246502
0.6858758840293278	0.4725765712756739	0.4290950091651953
0.5210387707054793	0.5198186587192019	0.3667683513484447
0.6937640507431236	0.5154915315958130	0.3263493348405413
0.6326844909570435	0.5198675181163835	0.3230529940170043
0.4765910864573863	0.3722064234417013	0.4156254463449656
0.4785954710372737	0.3108295492597680	0.4152608886118441
0.3116599271701131	0.4864030924384877	0.3150918485010509
0.5266529380802109	0.3128556198611280	0.3148116512922746
0.5258476936686310	0.3741851169771006	0.3158664030874083
0.3729360211523692	0.4828029812420708	0.3143370296652436
0.1237471471830810	0.5285443259740946	0.3706864384906677
0.5107329094289660	0.1209168737625715	0.3597618497747140
0.7385117385781910	0.4829371282995967	0.3540817461564056
0.7944519134996098	0.4733375292918758	0.4508977092765942
0.8549748914796732	0.4644583920523983	0.4420395730938325
0.8804309593579146	0.4763826578374299	0.3839627391073589
0.8451171891732177	0.4965953743573416	0.3349893865506670
0.7851242299531349	0.5045744457494901	0.3436083948187960
0.5083233509067169	0.2627694627986051	0.3901853687503907
0.4572256611810968	0.2034949748982612	0.3156816180858336
0.4629812789505605	0.1426869721879428	0.3254300319319180
0.5448288509373957	0.2206347793151247	0.3772704318006391
0.5517453025200958	0.1601861631785378	0.3853462849034641
0.2648041425298559	0.5160738918961417	0.3421028948560975
0.2260769954464200	0.5548656805764604	0.3556400601603354
0.1665360871100592	0.5668476362570903	0.3468371492306750
0.2010099443666080	0.4672356523167596	0.4141563416838893
0.1410642758217260	0.4783128653851025	0.4039353410640098
0.4953457046471456	0.7388116011146622	0.3846901458759738
0.4601032923914792	0.7815134072418566	0.3714965530353977

0.4544455268413731	0.8419817194809955	0.3803390236020152
0.5495360322459653	0.7973807963720796	0.3124824662621786
0.5449985506650569	0.8581725093160731	0.3230498958777021
0.4969747184952902	0.8805941851832885	0.3564903990784982
0.6137381622461038	0.4329312407906086	0.3725932588276178
0.3151407198241613	0.5271633319085745	0.4190900600644319
0.3777887573575880	0.5505155671509672	0.4988740235613076
0.3699953311522025	0.5049631381636979	0.5524786270603373
0.5814103916616299	0.3992077285103279	0.5022099688608290
0.3971018329614512	0.4194379201650099	0.5017983589183047
0.4505578117591887	0.3798049831267866	0.5005087332634053
0.4174727020745314	0.6037779280976977	0.4969980784637602
0.5483812942657708	0.6231575295370427	0.4994555592338206
0.6017488474606821	0.5836948971308765	0.5044290560668044
0.4599543054989411	0.6219193419469244	0.4444442279034762
0.4490269436239187	0.6821817112408060	0.4420502551000418
0.4832028438148893	0.4798476391817457	0.5012808317065535
0.4878989574576560	0.6927786258751967	0.5462991453762914
0.5000951129733119	0.6326530741602402	0.5480518166150199
0.6258675425136883	0.4964198492933439	0.5580146340182058
0.6868016727551076	0.5021935791706840	0.5608325321433645
0.5158557834543402	0.5230775512013661	0.5013971087554244
0.6866697310743256	0.5461980777327706	0.4582872889518489
0.6255798389091396	0.5413790620086667	0.4559873450805931
0.4982809844277241	0.3705267797186274	0.5509852840335537
0.5108737795245806	0.3104217187616919	0.5498224835195441
0.3156293108489857	0.4544527214628906	0.4489641390708400
0.5514923641707720	0.3207254780242006	0.4462807491644562
0.5400418604680987	0.3809096073443738	0.4480484615086745
0.3765343226072158	0.4620782396482906	0.4496960908244506
0.1224859036712322	0.4550484168964111	0.5014569242501009
0.5793005408311176	0.1306078694347652	0.4905716407294617
0.7364730973407198	0.5208700847195826	0.4854505419409977
0.7929436251280030	0.5254936933150388	0.5835350930548839
0.8541909547640247	0.5266901692446556	0.5754235513386472
0.8779034654445144	0.5382977642201798	0.5164368789856045
0.8401815784158088	0.5479315496930568	0.4658830075576976
0.7797074920000253	0.5458067271295899	0.4737678407155822
0.5468790636439966	0.2684298290532726	0.5227738910864975
0.5097994973847157	0.2002458426418141	0.4465371623006513
0.5287105632466761	0.1420429591573293	0.4551823410869323
0.5900411535212011	0.2349364486119681	0.5110569941715545
0.6099254557023739	0.1773697051357875	0.5182033504401929
0.2635088913772647	0.4738958432953901	0.4746677171287044
0.2166286583453579	0.5030158483337580	0.4862801801194501
0.1560581908208181	0.5012863349351819	0.4767547226335251
0.2108312460087774	0.4136112092783750	0.5474204605383899
0.1500883821634449	0.4107497051290478	0.5363216275074068
0.4528851087171889	0.7346549990879921	0.5186203086017337
0.4100166365530367	0.7680592923138513	0.5072255861861242
0.3901934906143543	0.8255741160706795	0.5148685646430101
0.4900020178602408	0.8030863129610336	0.4425548286916823
0.4710936516508107	0.8612513344189251	0.4516264668351993
0.4206727893782474	0.8725007402971520	0.4873291719684579
0.6210310877560271	0.4523935961138251	0.5046605325322098
0.3093373031839578	0.4961382867440855	0.5523944702357521
0.3705354648509676	0.5258580738820213	0.6316562036746852
0.3718698433125107	0.4792381588076606	0.6850324567687766
0.5983356799141314	0.4143090320722548	0.6348235967257660
0.4133587559901123	0.4005071591555289	0.6330894542887820
0.4731492966521170	0.3712841470327731	0.6318383921160357
0.3998121258779795	0.5854882766752549	0.6300473943370632
0.5250067384102814	0.6284906717622867	0.6317985847966223
0.5846612954798899	0.5993494775988195	0.6363106390765034
0.4385332949625851	0.6113733097375412	0.5768754628228761
0.4173878715820043	0.6688737231879632	0.5744558479745364
0.4870124434534535	0.4756144180247354	0.6331713802019051
0.4535523383425889	0.6859016575797682	0.6788608425276008
0.4756485692861492	0.6286638280440160	0.6807628565923406
0.6248495328785936	0.5190041241673368	0.6895161535764202
0.6836037600755217	0.5363089173792825	0.6913108946970170
0.5111514224132309	0.5241600316630713	0.6332739477434893
0.6740151920419276	0.5779173097931680	0.5881605925051445
0.6149006031968015	0.5615389770532232	0.5868592105344617
0.5209682748702120	0.3709954806003151	0.6827365388277200
0.5435330832988295	0.3138772384862524	0.6815726371816559
0.3255529294868982	0.4224198385736072	0.5808582549811592

0.5832289229231569	0.3314083085741112	0.5787184493285089
0.5615039275581074	0.3887331227196754	0.5803702105700276
0.3844190792815371	0.4398006252744046	0.5813380035605465
0.1357716926036748	0.3890360686793723	0.6323697888445171
0.6424600211407315	0.1488366480889742	0.6253394538412147
0.7281443893601643	0.5624960054910634	0.6152671067107852
0.7827649635237685	0.5815344628997722	0.7126854010172483
0.8425623454699116	0.5943974500317655	0.7038998124209525
0.8634263839972116	0.6082990107303876	0.6442658517069548
0.8244240956666969	0.6086137475706177	0.5937692404373127
0.7655423871642933	0.5950457246065339	0.6023641346529398
0.5865146820218428	0.2789001133637020	0.6551423863397265
0.5619080126798194	0.2046750961249056	0.5803604710144407
0.5904837054325746	0.1507240183110292	0.5902296721162392
0.6356160002278057	0.2539880365397695	0.6428969192054890
0.6649496671674092	0.2007239657364219	0.6514284930843159
0.2714861578184081	0.4319581936406291	0.6070096892762039
0.2198853738272576	0.4531660128563679	0.6179768520307837
0.1606951899192851	0.4405033238690125	0.6078324569142015
0.2296150697888161	0.3644673361420932	0.6797050785979292
0.1705151873195088	0.3505349586694864	0.6677571470803171
0.4122566009743578	0.7213190080190520	0.6511063344956142
0.3642459377243460	0.7471574121658959	0.6391221518317144
0.3357455674378175	0.8008153547595259	0.6477651402797886
0.4385880980967861	0.7954372177818774	0.5764953331466763
0.4107663324744084	0.8497662160330560	0.5863682392623989
0.3589388837607871	0.8524039157571415	0.6216913779168183
0.6275680665044433	0.4738866409121965	0.6369840264415220
0.3134863724176978	0.4606756381280623	0.6852073266318287
0.3669441982970230	0.5033763450690232	0.7765963785094679
0.3809791296745315	0.4485459504811601	0.8339753104420400
0.6099398681431847	0.4315965936908646	0.7782594753636847
0.4294793428019874	0.3876938962896223	0.7783038279507348
0.4932172296576152	0.3681737305705713	0.7766724947593403
0.3854656261443574	0.5673266297819347	0.7736802956717055
0.5022194475028070	0.6308014797046834	0.7745805307866817
0.5658740658149548	0.6113631839612406	0.7784962247115397
0.4236811941919110	0.5999240645389569	0.7181216476199831
0.3964262360592986	0.6544653384300642	0.7118772830840896
0.4906743125167620	0.4733293216072906	0.7787872288001444
0.4214738962235792	0.6772978268998040	0.8188552980517438
0.4490781457570839	0.6229209220141908	0.8246343432876675
0.6165631609726622	0.5477534103607551	0.8343586932442598
0.6723128124696791	0.5735304066220170	0.8331171059305668
0.5047494175143875	0.5256806352782653	0.7781086405516662
0.6595153108544655	0.5951235145545817	0.7236546332957644
0.6034203501605232	0.5707964606509566	0.7254257571405794
0.5455439471561786	0.3758677228930212	0.8276157125060005
0.5733371742202961	0.3215407812001488	0.8220880785152593
0.3341712226401936	0.4057579889404703	0.7228301015040992
0.6001769510321525	0.3448888745211319	0.7157220656509138
0.5727085356647333	0.3993446723487104	0.7217044685724231
0.3900517345093455	0.4307762075108973	0.7234953398180344
0.1505842348882328	0.3448362190460177	0.7757180836499243
0.6709159863278010	0.1624596849782887	0.7670757749492827
0.7099588777423033	0.6016505359391137	0.7496831202922000
0.7753499577886844	0.6175995328693821	0.8402167030403862
0.8283642444950184	0.6469432078901672	0.8263881235617254
0.8356944203546299	0.6736721979289695	0.7680838761612319
0.7905149304378459	0.6703019558838210	0.7238892071004187
0.7388205192942547	0.6408141036019420	0.7372766704388476
0.6092320857994326	0.2878875430712313	0.7907092297724273
0.5995383884903538	0.2192418495617292	0.7082987866392946
0.6271660465944298	0.1654446677728591	0.7211986463515258
0.6592675097823479	0.2666858119048912	0.7871072738741326
0.6869577680377081	0.2133757404167398	0.7997125159949244
0.2850906826535267	0.3963718312558829	0.7510499278241711
0.2349035304882444	0.4027319020592254	0.7445293284897635
0.1779353984705422	0.3836515110117736	0.7341083668573792
0.2385312084247050	0.3460941025357920	0.8399926238847959
0.1810656186993709	0.3254992945063420	0.8286401971847820
0.3867611002109343	0.7113714505608272	0.7870437364116455
0.3370892054389982	0.7334412246609302	0.7833923734533076
0.3103693772122034	0.7872024802142867	0.7961577389705200
0.3980102777340450	0.7801088039083800	0.7050729233138605
0.3713983692116232	0.8343650591785643	0.7181900223274923
0.3274800169128005	0.8379487127051615	0.7638228403707178

0.6284814695326513	0.4955304692465350	0.7801681491582233
0.3256932878295118	0.4219273863281158	0.8338818338554742
0.2787439448171056	0.5449226535400284	0.2314390130431276
0.5396897517207477	0.7241663427842774	0.2192328216772715
0.5291707961405795	0.4579209908105234	0.2251220750226062
0.4576319468948808	0.4724084881355159	0.2199095577133378
0.5462885126794453	0.5279520841268182	0.2246475189494840
0.4746943372344810	0.5423845511488884	0.2210795135897408
0.7238461239809549	0.4516493362250912	0.2455295198633624
0.4611726782511751	0.2766245520518870	0.2226837703269524
0.2744674528402265	0.5099397141402743	0.3660073262750675
0.5002723492916593	0.7285981236365316	0.3607224480853686
0.5366949497791552	0.4645542977231889	0.3683973634197661
0.4642585574762825	0.4641201466547182	0.3660725103407074
0.5392680360191069	0.5373870907903573	0.3672094197845359
0.4668732261720793	0.5369634120023902	0.3644985077280127
0.7287488321230534	0.4900933033385300	0.3776268932464075
0.5039930463095917	0.2728329031870813	0.3659866386628131
0.2737486104374861	0.4705260769060195	0.4989374604959599
0.4598999647704357	0.7256141560140524	0.4947006441899429
0.5399175975603117	0.4715596804409463	0.5025790292055652
0.4681675418014792	0.4594737084911100	0.5013251086876455
0.5308219776392510	0.5435031115344581	0.5015450342561083
0.4589565085710151	0.5314817419127372	0.4998587431647581
0.7258265236024443	0.5263611123741856	0.5090961101855478
0.5399517503777821	0.2774909281963462	0.4988230298314584
0.2820583847938550	0.4306435719531742	0.6313372682451670
0.4205955646073066	0.7135381010743072	0.6271877695107950
0.5443114950458674	0.4778455264757670	0.6345896133314748
0.4759467506283976	0.4528361660393718	0.6330577329320234
0.5221884132203169	0.5469520874358041	0.6334788555101286
0.4538179997339244	0.5219668037326196	0.6321597987270534
0.7166644581611827	0.5667132170051241	0.6387661781378872
0.5785811191123466	0.2866770820960802	0.6310531873543082
0.2939985761978162	0.3987840212803856	0.7759027152641463
0.3916292394823057	0.7009450541204407	0.7633761342146268
0.5457850912136463	0.4849066769271160	0.7793667139992719
0.4831183826419311	0.4491566774320734	0.7789043211458913
0.5122930448921771	0.5498576687334856	0.7780422905607723
0.4496284525519654	0.5141206348240118	0.7771600825944392
0.7016779743198711	0.5996099461927046	0.7748661754968575
0.6042931387483264	0.2982771741481027	0.7670418494238955
0.7347103678451425	0.4607139075750958	0.3039260030307869
0.4444994066659069	0.2642462887490115	0.1666094703665237
0.2662015205336437	0.5341505359070318	0.2892174396562069
0.5501168678427971	0.7357844402187672	0.1613505041257924
0.7399176542911557	0.5015439078347250	0.4353408408081136
0.4855676930055807	0.2620729083854151	0.3100749339257934
0.2618497860879551	0.4902476557276443	0.4209751959866215
0.5201629889620942	0.7394863775662325	0.3053749348191238
0.7348126590358468	0.5412500856220276	0.5663109372324647
0.5237257741673464	0.2634073368218894	0.4430540968289413
0.2648292953768497	0.4494026589589733	0.5540964925674980
0.4763986563726290	0.7398682607721662	0.4390237617819477
0.7225253026223142	0.5849351107909444	0.6953723782114913
0.5650913679158959	0.2696092573990982	0.5753753752237812
0.2757695284080838	0.4100922450518527	0.6872364851339927
0.4350008996294735	0.7306901227682364	0.5717878928081019
0.7168021060094562	0.6049972844887066	0.8326643078904382
0.5996206031381305	0.2844147889055444	0.7087227920632069
0.2844240989014272	0.3854375844215130	0.8335494091238320
0.3966501980522759	0.7149479319739805	0.7050996212889187

```
N-CENTERED HEXAMER
   1.00000000000000
    40.5026200000000003    0.0000000000000000    0.0000000000000000
     0.0000000000000000   39.8320900000000009    0.0000000000000000
     0.0000000000000000    0.0000000000000000   41.4138799999999989
   C    O    N    H
   432    24    48   300
Direct
  0.6379679088595792  0.5089851263698211  0.2237029021041858
  0.4259564488036727  0.7909335284543211  0.1886305029048140
  0.4071085671856401  0.5525521500390136  0.1847206264673526
  0.4279398155290929  0.5792554718497975  0.1831491540892382
  0.7926150011874333  0.5556705751607161  0.1944993710534109
  0.6757076431288707  0.5314301706757513  0.1720474847710135
  0.6419177945931074  0.5263486706268850  0.1682001979036792
  0.5401199508568912  0.2507043259189738  0.1602212873171021
  0.5309041786756482  0.3075566273073240  0.1839305321398568
  0.5286162132064411  0.3257726846612997  0.1549289886663288
  0.8265272095227453  0.5590838375218802  0.1895097543839023
  0.1708647155561109  0.4673544364266286  0.1817579957861099
  0.3265435161528527  0.4672958184205412  0.1650011178259868
  0.5730769807567040  0.4776649595546149  0.1898615386670208
  0.3098160192379234  0.4740912093291773  0.1939574620712313
  0.5165206330722301  0.3770612235271552  0.1850547037334626
  0.4136788577631172  0.8234545160992272  0.1925884353724882
  0.8030468219457048  0.5327240593564315  0.2476498030274603
  0.5177854795454678  0.3583516795209509  0.2137174749012317
  0.5536282946434063  0.2164537274991569  0.1678115364620726
  0.5449276642067081  0.1903948673774990  0.1466179428830591
  0.5574095403718339  0.1581377678761459  0.1510197050583313
  0.5753742088942246  0.2096732130390333  0.1933785365283808
  0.3785092547329290  0.4852011669808488  0.1889004511651820
  0.5882395147300985  0.1774019386315377  0.1974542902282825
  0.1696760617218752  0.4224132598092434  0.2210256394931598
  0.5792434803803315  0.1515772029734934  0.1764109066833975
  0.4554626035114370  0.8091025065941491  0.1408794401100015
  0.7805614456173838  0.5422892087016639  0.2235838390938210
  0.2560561392972519  0.4584919126178230  0.2204574908963091
  0.2204105059998413  0.4533586171486359  0.2119563926967930
  0.4469381825487292  0.7834778999636974  0.1626307746341353
  0.3273989906827955  0.4870743033116394  0.2202834437342528
  0.3604136324927598  0.4726855098965830  0.1626032662190775
  0.2041233020430097  0.4729915398879954  0.1887116107179288
  0.3612924486798475  0.4926009438633120  0.2174549962477979
  0.5397381891609603  0.4347975399064056  0.1870403502257589
  0.6908618870434154  0.5257212903521795  0.2020442368851053
  0.4598569958929071  0.7489393059377232  0.1552713161093904
  0.1536386919828743  0.4419181200580908  0.1977456839317187
  0.7444173788142897  0.5382083541335662  0.2306729420963072
  0.8369194284033167  0.5357646656102125  0.2424651890854777
  0.5250355012731154  0.3241865204185649  0.2133141430081769
  0.5217003265937434  0.3600680137277055  0.1558542774386255
  0.5938645261850318  0.4477813188579078  0.1902808719568718
  0.2027832388141244  0.4282570575298758  0.2282879040628032
  0.4613268463895192  0.5655222975377988  0.1833674732328169
  0.4435715214741620  0.8416065882245654  0.1451600612885139
  0.4898049132696806  0.5860378900335901  0.1829958441770159
  0.4150740583587721  0.4897435953306248  0.1869253467432334
  0.5113149708774121  0.4142749614582501  0.1857347733851046
  0.8487457103711532  0.5489228556039699  0.2133931958691334
  0.4780084179461561  0.6768662802832798  0.2089499213986740
  0.4225065474584177  0.8488471559521559  0.1709954412036299
  0.5730800880775080  0.4210528654848110  0.1884123571904258
  0.4785171638110561  0.4257157809044526  0.1856249425108605
  0.4341200505612698  0.4602027515573726  0.1864693089213608
  0.4855815845340171  0.6427820171006604  0.2097239587484264
  0.6223301672591329  0.5152088957685746  0.1940502975698828
  0.5858693585827622  0.5106606031732175  0.1905156639509203
  0.6717499370315436  0.5142172100429852  0.2279906266468836
  0.4845661725516338  0.6232026878775778  0.1815814757279374
  0.4768820135311047  0.6392870333615842  0.1524156887511532
  0.4697721760353266  0.6735170075445792  0.1510922115283277
  0.4696752489925489  0.6925703931325343  0.1796747852765927
  0.4224240515371901  0.4261924541371227  0.1870920594894584
  0.4493806808732054  0.4052761230436935  0.1862643830547691
  0.5668740523276150  0.5401582696305831  0.1883774630918185
  0.5225787910383664  0.5746163413689523  0.1847042519998869
```

0.5517155765751697	0.5950459006454055	0.1856974195642576
0.5785840773627590	0.5741582535943465	0.1882399718741592
0.4278968425866125	0.5227040511876591	0.1858385554670673
0.6357493628559635	0.5317683017326977	0.3510081762427855
0.3744863672456202	0.7735054768748416	0.3107978303906364
0.3990337537380855	0.5329268958218395	0.3155447378965931
0.4138800270600083	0.5634962252276133	0.3144619084122637
0.7841209464023343	0.5933245931143135	0.3158380347314989
0.6651347251152153	0.5657858547688112	0.2999125287914503
0.6320054748519086	0.5566096577593568	0.2981480768806872
0.5787599022893078	0.2607297844768698	0.2909612139519008
0.5655798354386756	0.3161749142097352	0.3146892421283188
0.5712530087411659	0.3376848925778355	0.2884892883724165
0.8176293047802365	0.6003198685165969	0.3117344126904122
0.1775697696516663	0.4297828232136030	0.3147641892772537
0.3371068285557119	0.4355257610148335	0.2921888331018316
0.5757679572004384	0.4928221448091442	0.3201176258284263
0.3176302361496384	0.4417563967153338	0.3194975593408393
0.5404966723955900	0.3823548184911989	0.3154070386529274
0.3581505661261800	0.8040893440420273	0.3148910586833732
0.7937377492093164	0.5823720209830852	0.3725452206502176
0.5345758301007777	0.3602922981479478	0.3412089615867921
0.5957952589900195	0.2280420343145351	0.2977704692282541
0.5824741206464561	0.1986492820362651	0.2844018687895868
0.5979378295985607	0.1678090195337866	0.2893235576699516
0.6250856074994895	0.2261665333521916	0.3157615289865117
0.3844706430628363	0.4617447850990410	0.3181559712706581
0.6409486863415447	0.1953428120688791	0.3199975434998434
0.1818436229205850	0.3775684256760374	0.3443050824862210
0.6273418759220724	0.1661096916991368	0.3069688132706589
0.4168738088038987	0.8016009912412848	0.2796300206770623
0.7718566985129971	0.5842782188988995	0.3463283511531581
0.2665245329635819	0.4176320021233274	0.3430415014608032
0.2304961112430286	0.4121541178081066	0.3364374856327118
0.4039153254931628	0.7720382839687540	0.2929875469234910
0.3316708664474491	0.4577667689291304	0.3462892190574577
0.3701377448669718	0.4452807035358527	0.2916701404381823
0.2113343533752787	0.4353251577240739	0.3190966019671641
0.3646364695009500	0.4677370949314005	0.3453816567711525
0.5520271900112443	0.4437418070391519	0.3173286405644016
0.6838521281072936	0.5579566216600448	0.3273622224181678
0.4214645247142668	0.7395846232146767	0.2863763409653658
0.1627997577874324	0.4008412559222777	0.3271835755513517
0.7359862437797356	0.5775199327967598	0.3528583092025103
0.8272546354698321	0.5889645331401648	0.3683252790503577
0.5470947311253239	0.3277167070348054	0.3410370234509826
0.5590107996893708	0.3703699349622855	0.2891113805442884
0.6022537298569076	0.4680165765416973	0.3201750003638894
0.2154065480990578	0.3833676898492985	0.3492206159269337
0.4493445228778642	0.5572040533776310	0.3145816605021824
0.4009081000753013	0.8322087519546361	0.2843733457889149
0.4731106905007312	0.5832443319701837	0.3143756939009901
0.4195720504961231	0.4731759556938036	0.3172943352206599
0.5282972260886122	0.4176947079019547	0.3162089032169821
0.8392782699736091	0.5980491618652168	0.3379112151842941
0.4519966950681323	0.6729476621843119	0.3382045292919461
0.3713856484281313	0.8334946884332908	0.3018571519321897
0.5874507773909217	0.4374486015835616	0.3186811232926505
0.4938522155578145	0.4224116489624397	0.3163023138006822
0.4439189898664577	0.4479678685867093	0.3169429345201811
0.4648211520724925	0.6405146578315346	0.3390346820688228
0.6167406339693311	0.5391300544963867	0.3235252547441708
0.5817015547146532	0.5278240738163925	0.3210767683799149
0.6688416989562512	0.5410949394823323	0.3531586265741959
0.4608663712482798	0.6185426730136416	0.3128089581512555
0.4440883838119677	0.6304899654221178	0.2854167721455239
0.4315732790103321	0.6630548465870679	0.2841254381619099
0.4352076530671512	0.6844571898779813	0.3107627886223868
0.4386286909634078	0.4123614078402451	0.3166007160061045
0.4689112488822866	0.3968676796900697	0.3157633139252763
0.5573846367157055	0.5530405102035020	0.3195069553033213
0.5075108615748265	0.5785580609596813	0.3162057236381368
0.5324115709167764	0.6041264210729790	0.3170816504939312
0.5626222016554042	0.5886642381989138	0.3195518103528996
0.4255132734878498	0.5081496388255839	0.3165911946677409
0.6269540008793478	0.5554530400098170	0.4718458219201266
0.3303101008945800	0.7488197162404586	0.4338122512899347

0.3925227491564373	0.5143070605678014	0.4383621233029654
0.4021129203322746	0.5469722063498825	0.4371985757340063
0.7610873450477604	0.6435883732691199	0.4340885803892744
0.6487761018710284	0.5941196279346159	0.4201364908075043
0.6177401500019606	0.5790927862984321	0.4191078229651195
0.6183197078182404	0.2795497228756443	0.4126817529624150
0.5943376778336641	0.3312189270171705	0.4364622990690487
0.5947397325493209	0.3530150516506865	0.4099096971252593
0.7927381776474699	0.6566634879255782	0.4296311996596433
0.1929355322648956	0.3694900059044657	0.4350345875861596
0.3492916534322409	0.4055240236329719	0.4151062885389052
0.5737168674719348	0.5062898372941240	0.4423787286514494
0.3280352695609270	0.4091324355634464	0.4415664135242384
0.5575515122806829	0.3914348529713379	0.4379445920320573
0.3093602962737251	0.7764024337930584	0.4375274344095614
0.7730011420443899	0.6351264063964162	0.4907877754697111
0.5568435320909906	0.3689731473469590	0.4640568339491197
0.6422101599211881	0.2515863037453862	0.4191976462861823
0.6357987489137545	0.2200085385383889	0.4055627889048341
0.6578071469334602	0.1935377525903037	0.4101546780777029
0.6712186257357174	0.2563793674617927	0.4371339115131518
0.3901434187075332	0.4413533543898662	0.4411485758540563
0.6935797913614756	0.2300095826704299	0.4410878193543364
0.2064816396619424	0.3204377101708339	0.4668115454673259
0.6868624040155873	0.1985287005339123	0.4277693202264672
0.3667763401032737	0.7825121602513668	0.4006694888604571
0.7509266559703118	0.6327032203108549	0.4647634312785928
0.2817794978861694	0.3765945272046722	0.4650432209379008
0.2477244821882220	0.3636802325728872	0.4580944505151237
0.3591378483830622	0.7516647637586982	0.4152179630910411
0.3379170589332888	0.4287409454666580	0.4679327608404734
0.3799911103627688	0.4212880981944961	0.4150902518477335
0.2248971639502006	0.3817999304398523	0.4395019823436890
0.3685131011220628	0.4447387517805417	0.4674820740769038
0.5583920964113240	0.4538422794959083	0.4399370251857023
0.6691462945946213	0.5899271921153743	0.4471862444630956
0.3815904159036251	0.7224413783828095	0.4089632125213593
0.1837159005756632	0.3387395383305819	0.4485044398359982
0.7169146267973836	0.6195101804283294	0.4716749868912450
0.8047391585068465	0.6477645180597269	0.4861947441746973
0.5751185837252922	0.3393421724942663	0.4634884134491839
0.5767018125885490	0.3827811017144068	0.4109008001931062
0.6039248359393671	0.4863853015892863	0.4421441447623907
0.2381653309135588	0.3330175553201301	0.4719015786955756
0.4381068468251909	0.5468476892597897	0.4374920599080883
0.3461825500394603	0.8101803279033678	0.4049777343602329
0.4571644591632336	0.5766051773243895	0.4371196754592601
0.4226891789883754	0.4589148441316732	0.4406269877148032
0.5394048811252151	0.4240425152615144	0.4391372618878934
0.8146633731521618	0.6586617403265675	0.4556020474856944
0.4220540939863824	0.6616626158265548	0.4608752246588749
0.3173138422552705	0.8071427048294152	0.4232815276190180
0.5943742235916426	0.4537208197689684	0.4408089004890150
0.5047004741341136	0.4224770074088730	0.4397050975638076
0.4510512244641117	0.4384753557104049	0.4405122516509857
0.4397371806921096	0.6316879055218551	0.4617438133021999
0.6063384835182277	0.5591967566013568	0.4447866018786257
0.5737538347517965	0.5418103198524143	0.4430364035655682
0.6579070248108037	0.5706697315626773	0.4732683251346078
0.4392259345156530	0.6092945905608824	0.4355656958858556
0.4207494110469766	0.6182980209890696	0.4081902182018630
0.4032997426349010	0.6484206453532970	0.4068858131670270
0.4036521750268168	0.6702326327890114	0.4334344731395573
0.4521501995934624	0.4024881280281354	0.4406807956511033
0.4846786781999640	0.3927826934884946	0.4397502642159335
0.5454422528462851	0.5622562482627557	0.4416059597270965
0.4918471565219334	0.5781653034665520	0.4387204345399082
0.5118357187159384	0.6078840097745458	0.4392118146157982
0.5443124423545893	0.5982336556223474	0.4414108545350623
0.4227112099455861	0.4944192505054453	0.4397089786997112
0.6153772030033506	0.5797076760278250	0.5918851154959400
0.2887320095739816	0.7141090813482373	0.5554919267679637
0.3925765061093826	0.4951545279063901	0.5599737047757540
0.3959267399145086	0.5290846898617916	0.5592077722235511
0.7324495001731488	0.6895787385592203	0.5533119983533578
0.6299626608903178	0.6213341208086947	0.5398919657739251
0.6020909558285948	0.6008738000856927	0.5390082691834635

0.6538611954860900	0.3045168328800582	0.5333221838631649
0.6230756019604629	0.3520492390931428	0.5573266876437446
0.6204437347843377	0.3738360233246912	0.5308913849931246
0.7613644312176611	0.7080650266016341	0.5487662115211980
0.2221758051335631	0.3173214450830176	0.5577979626322669
0.3692769368322790	0.3810654031061048	0.5351622163504915
0.5721171870289075	0.5213038720481967	0.5626131254211738
0.3484975450104627	0.3798428222510533	0.5621695552534458
0.5771283390221034	0.4053014936374505	0.5584484571006179
0.2629972336307541	0.7371225424453728	0.5592496956373374
0.7446857724618526	0.6853128299463793	0.6103637408964603
0.5797793069326143	0.3829736673602939	0.5845517787915872
0.6809281080170460	0.2797267052002226	0.5397317868055352
0.6780638542293532	0.2474232334440680	0.5265298660738593
0.7030838067351312	0.2238691468364692	0.5310738111720793
0.7094291125671528	0.2882655852065448	0.5571425681921672
0.4037246961735418	0.4230574727445161	0.5614860647330019
0.7347987067829168	0.2648590872245751	0.5609960796712830
0.2445595679959336	0.2705118058617379	0.5878982310050472
0.7316079288505837	0.2325945706105141	0.5481590302695715
0.3188235412264573	0.7547738076614878	0.5235010916264347
0.7238306671808439	0.6780228851293132	0.5842169741428156
0.3094680159990148	0.3387462835462658	0.5856916763939995
0.2778120603969267	0.3203851808743313	0.5793242190031935
0.3168126272709764	0.7227481441769240	0.5374673626013126
0.3554725195717165	0.4000967433257248	0.5889737349192085
0.3966343500392435	0.4022678205292433	0.5350036432944498
0.2517731885935305	0.3348099523854018	0.5618069811615766
0.3826558775059833	0.4215187508785247	0.5884001034922235
0.5667662223412528	0.4668735915841360	0.5602402693244937
0.6507522156553419	0.6211604007681263	0.5669357204303545
0.3443666144717194	0.6984205600818656	0.5312676507479428
0.2185645431563629	0.2851068322205051	0.5706526015233822
0.6926828171003852	0.6588843459188377	0.5912292610088723
0.7737311062836196	0.7034334785601790	0.6057162327404343
0.6025659974680057	0.3567808027382193	0.5841796121613686
0.5978329533473891	0.4001825834022032	0.5316765626018687
0.6054776214971797	0.5075185081169372	0.5624822059493214
0.2738647815892026	0.2882037209776259	0.5925394121827986
0.4313072933203354	0.5358590474911703	0.5589248331801787
0.2934144245395194	0.7779323918480416	0.5278542642037934
0.4445652927065423	0.5687211980840535	0.5585899883559344
0.4324783287457534	0.4465072641997156	0.5608613179819980
0.5535164469784004	0.4340426516368777	0.5593477001625546
0.7821165174119364	0.7149344095125484	0.5748910564016002
0.3953447144716565	0.6461947795502002	0.5826563803994573
0.2653430296241222	0.7690999819459309	0.5456003221440220
0.6021279351642167	0.4736076493709770	0.5612238222168680
0.5196772098234360	0.4260317978431649	0.5596321762460904
0.4640600293727821	0.4317794480683054	0.5603619204988344
0.4180443570191668	0.6199321384114970	0.5833819489977827
0.5944312730050040	0.5794103755834377	0.5648409922691338
0.5655614769120206	0.5561867385352711	0.5632460446901659
0.6431164297801747	0.6003657340512827	0.5931820112581716
0.4209875885579857	0.5975049348262298	0.5573733788687437
0.4006031208914075	0.6026373566847514	0.5303583341024048
0.3780948986863326	0.6290720613284790	0.5292109647239255
0.3751855469432158	0.6509384257611033	0.5555485658510508
0.4716054022070048	0.3966018679744664	0.5602581569178670
0.5053292174560722	0.3931191237660335	0.5594145694970383
0.5340028098238460	0.5709408271748070	0.5619921391386221
0.4784085358650449	0.5767140892919900	0.5597244579180770
0.4927734283832272	0.6096249089787383	0.5600992295324918
0.5264587203953391	0.6061152717083990	0.5619174084269000
0.4259243059592984	0.4813879515414026	0.5605145268655011
0.6009598232077089	0.5987630464337048	0.7133160737305682
0.2556807216362286	0.6716447188476237	0.6785653247292875
0.3958947119198407	0.4752953168328290	0.6824855570803444
0.3933653700578234	0.5092977669639825	0.6817721476079668
0.6944876659835162	0.7296021619038493	0.6738640803250161
0.6069996539462509	0.6420961583462785	0.6609700694797112
0.5833182313266218	0.6167428172303809	0.6605703848432622
0.6855261953075944	0.3339539512560603	0.6546602310924602
0.6470425914595758	0.3751640645320911	0.6791531859859430
0.6403857941660152	0.3961587784777721	0.6528166561342845
0.7193101565262401	0.7534322601703509	0.6691684390546598
0.2600168466854209	0.2675387246537427	0.6773887380610307

0.3929992413690091	0.3588780978174679	0.6570740452818320
0.5681413012755340	0.5331292348900787	0.6849620857778724
0.3723121849474286	0.3538829360955870	0.6837611116223530
0.5929725641414139	0.4198048434915008	0.6811887746358294
0.2264274349666942	0.6896770422712640	0.6828214694779029
0.7088448659178808	0.7264879207637867	0.7305196771952444
0.5997586408860456	0.3983159957219701	0.7072257119428947
0.7169060882368535	0.3150334550195354	0.6608251984107718
0.7205884493860383	0.2831603319303805	0.6468704244618593
0.7496675192552789	0.2650230210533281	0.6512927136019225
0.7429737897233901	0.3287099962319206	0.6788621559870034
0.4192536699622640	0.4062966395674584	0.6838907934733131
0.7723289242784249	0.3107585975598937	0.6827116121347496
0.2913450385579713	0.2258430986944036	0.7067873017572430
0.7756707715355121	0.2788481006472526	0.6691088074647051
0.2774922436088957	0.7168624783264942	0.6458034393967086
0.6890357406470100	0.7158984017581466	0.7046439700306950
0.3406292158946894	0.3065253365831671	0.7069465401974163
0.3135902940558837	0.2819144182452005	0.6996748640111227
0.2814317220559488	0.6851056509714130	0.6599450786900236
0.3751982046326159	0.3750721509982126	0.7106318448469540
0.4162787950492230	0.3846221113347558	0.6573163284092660
0.2853328638518224	0.2908467455341007	0.6820760463285011
0.3982755482851003	0.4010164805834359	0.7104303674093582
0.5721876373014690	0.4785698889978839	0.6830251880292841
0.6278045918274237	0.6460381305079929	0.6877366700237735
0.3127499588204664	0.6661894565516588	0.6533321043461429
0.2630354570968413	0.2349826645030212	0.6895532149891858
0.6621819847340765	0.6909938033820888	0.7118477538847716
0.7338795619319373	0.7499615780434403	0.7256922385612768
0.6265151733693484	0.3763548953913182	0.7063588584844341
0.6137519488431951	0.4182532303439180	0.6540668211233163
0.6033412571329693	0.5254733566849210	0.6844829039814010
0.3162864965504127	0.2492962359667656	0.7121864346719909
0.4270406153177112	0.5222400991493806	0.6817921884130697
0.2485211535360352	0.7350724696040284	0.6506023738482842
0.4344524183913908	0.5569353312768223	0.6814884196441068
0.4435207795979885	0.4344829882661841	0.6836862210726735
0.5647886702008024	0.4438962379112429	0.6823434926921779
0.7391254454029987	0.7635690352313325	0.6949991324458371
0.3722033357808311	0.6241389787121643	0.7051027865588424
0.2228416822316849	0.7214644368622349	0.6690121595635578
0.6058553400185578	0.4914789864264434	0.6835422288853071
0.5328849982526821	0.4299365455735015	0.6827652651470754
0.4771581092140857	0.4256384386297430	0.6833978772518542
0.3990592827026950	0.6023230794573038	0.7061297171443984
0.5800244477120546	0.5944031945919426	0.6865981467082415
0.5557015997771384	0.5663082871413262	0.6855327191762214
0.6244719296400941	0.6242834876608477	0.7141372256074757
0.4062880903228694	0.5810487526176527	0.6800362675153065
0.3858829012017206	0.5827859528177950	0.6526484271529787
0.3591323803402703	0.6047489246239762	0.6512298160881774
0.3519717705672830	0.6254214710491205	0.6776786151051500
0.4906607653101344	0.3923561292377650	0.6833498758752626
0.5244582830608546	0.3949640788187317	0.6825637383927733
0.5220757997190439	0.5751600912366823	0.6846579805923526
0.4663615650356977	0.5708715004970392	0.6826955965550066
0.4747965392643052	0.6058396127841715	0.6831269446255039
0.5085640523800872	0.6084352726042328	0.6847497184731492
0.4310850286330979	0.4676560080259649	0.6833242036947301
0.5786840110655826	0.6179948003121479	0.8462953871653024
0.2379980612640386	0.6411878597154012	0.8096827908013433
0.4031871989431338	0.4557544480258041	0.8140556368467280
0.3944649622832009	0.4886834789689024	0.8129300969776390
0.6544240992482370	0.7567693733315932	0.7972151911676990
0.5879728536816538	0.6526346140806117	0.7888176130170849
0.5677331579098573	0.6244336857281777	0.7896152274438836
0.7098243672874783	0.3650882637296982	0.7780040533617449
0.6659144050787733	0.3995816443587210	0.8053890613154729
0.6543232049673324	0.4187218646232357	0.7793564854217392
0.6719204779878000	0.7860596742155175	0.7900821045397469
0.3015526164595418	0.2316064460905878	0.7988301710386636
0.4126873274923553	0.3460368158787943	0.7867338301029199
0.5620460525175313	0.5438500615409247	0.8177327482512893
0.3993423023226804	0.3335046957846081	0.8156533233112061
0.6068757790701399	0.4368675608103613	0.8114561347287301
0.2093336268974131	0.6593309825971945	0.8164383352393009

0.6780989899389378	0.7564972476137871	0.8508389391662823
0.6190697738909768	0.4179082475081097	0.8374342839416471
0.7408509773343974	0.3462922985866231	0.7860564609462889
0.7478418159773720	0.3167977055155548	0.7688705232309656
0.7763191699438344	0.2984396765075176	0.7755999306744590
0.7632237389024812	0.3574538211160739	0.8096550782868704
0.4382581583700246	0.3924990440287231	0.8155970848189320
0.7919281780197266	0.3392404726785914	0.8160805204027768
0.3209985452541632	0.2038954815347195	0.8471679579347298
0.7984140581705311	0.3095861950239716	0.7992201040482687
0.2528920980908693	0.6819907944114638	0.7688685257577814
0.6573533703452775	0.7416683489115523	0.8277082519746961
0.3695278160286504	0.2842267971149892	0.8387373595794545
0.3455224293967927	0.2567739660962086	0.8304249578244572
0.2601152857647677	0.6524671980774713	0.7859144831113847
0.4061536198435292	0.3503321322688644	0.8447510985943339
0.4320050230434235	0.3749548847451994	0.7868654427706444
0.3244044727207024	0.2574201499751326	0.8035630483168608
0.4251782405785597	0.3795147072433546	0.8443412856316237
0.5760522010435678	0.4910589948241491	0.8152662262506462
0.6040295553070248	0.6638573720626875	0.8169319388459030
0.2911272087135222	0.6338064163111314	0.7775509983848455
0.2998505084575880	0.2047881943207510	0.8204897320204162
0.6383833591658847	0.7110629398562170	0.8382444520432697
0.6957486808529639	0.7855577055853783	0.8436012325630757
0.6482108880787796	0.3996085026796858	0.8345342882311474
0.6252189746539174	0.4371335053286618	0.7826882716031931
0.5980457384825165	0.5430246982140001	0.8173691262439136
0.3434577102061768	0.2298415096247556	0.8522170484941630
0.4251877867548746	0.5077661957032494	0.8131505960430859
0.2244592721043333	0.7002885536424905	0.7759368893480972
0.4262354807646598	0.5432326201771699	0.8128479091102984
0.4573478249080115	0.4246712188239586	0.8154171061571446
0.5750249852365240	0.4556286999514731	0.8139547247762604
0.6926501989262502	0.8004892776070489	0.8131826630150634
0.3529663519829332	0.5989622319615669	0.8337474345254791
0.2026180124400641	0.6890275123860695	0.7997306134702133
0.6067687880822765	0.5101154373986817	0.8158875509982609
0.5460975212347778	0.4360675876070661	0.8143859263713875
0.4919414456854115	0.4220432526731350	0.8152079046308626
0.3821049736101625	0.5806588821608036	0.8365176626742570
0.5631469532378984	0.6061789763366289	0.8182576276427254
0.5438629491847959	0.5741539026854775	0.8180980132295093
0.5987392959851252	0.6464954962982762	0.8460548530389106
0.3944193539207646	0.5620390922090465	0.8103593814718149
0.3761545425149255	0.5621231623893452	0.7815351500699814
0.3470277605716907	0.5805330332794515	0.7783392314077178
0.3353416527356229	0.5993376947469180	0.8045571972925504
0.5108548413176158	0.3916278274513411	0.8142831968467680
0.5437170946284495	0.4001276693273326	0.8134691090323862
0.5092714828154232	0.5767796522864529	0.8170039898988567
0.4551470965682980	0.5627681724956528	0.8145029257922861
0.4575265873032014	0.5987116378143388	0.8144412266833095
0.4903536902590412	0.6071977329147484	0.8162516887538469
0.4391678541063933	0.4549439676710337	0.8149243095187739
0.7333443230439164	0.5408051083309892	0.2585513212625025
0.4691919391860043	0.7415560710013768	0.1277094822025998
0.5300263560116953	0.2574339960723382	0.1327549182274583
0.2667842607088078	0.4538303333573587	0.2481801637587082
0.7240201167853236	0.5822360551434871	0.3802203242826717
0.4352036298011691	0.7342840205127390	0.2599951098354910
0.5652194473683717	0.2660987520432062	0.2644875749740633
0.2793028430144534	0.4085488376308890	0.3689577260886503
0.7043303115943392	0.6227450178044073	0.4989840553483152
0.3961525333235727	0.7192835106280479	0.3826451830023964
0.6035795564955230	0.2816447704340990	0.3863540460984518
0.2952602794495214	0.3715591111441811	0.4916439000681201
0.6798515223189553	0.6596337622444836	0.6185987597987888
0.3595524313629727	0.6983659059536199	0.5051048600285195
0.6390408155806175	0.3049304420904269	0.5069587421356525
0.3246097976170207	0.3352796936834185	0.6116624348499746
0.6498444230560868	0.6889800558272849	0.7393367649223274
0.3272231901466188	0.6687016322452863	0.6269174544762174
0.6705662478975866	0.3313948853081078	0.6285030506577901
0.3548831073322206	0.3065561059392483	0.7335624662916395
0.6352429663528121	0.7043417131333646	0.8672859099529746
0.3023770687880857	0.6331378532953450	0.7496684065900022

0.6983244956248478	0.3659184161595637	0.7502176012522681
0.3798718715214668	0.2874484517864002	0.8666985148540175
0.4605177432306830	0.7266551472910877	0.1807411596280605
0.5403289766549265	0.4692140123021040	0.1878200489272190
0.4680398559241887	0.4586082565673740	0.1856085033091160
0.5330032648899997	0.5417389846406523	0.1862082710765444
0.4607071129182277	0.5311362476558258	0.1848919819981606
0.7253507500166558	0.5309598286220057	0.2042188115529888
0.2754099039201735	0.4681863532182915	0.1948256643229029
0.5399092996857221	0.2734498432832517	0.1853094094267073
0.4211865609172574	0.7170077211265277	0.3113772286138538
0.5455073248822141	0.4775162500133967	0.3182443458091670
0.4775744219348119	0.4527565438370474	0.3168552339583827
0.5237842716244462	0.5482278025054351	0.3175752234381669
0.4558305198824409	0.5234461005951663	0.3159331604627285
0.7179635172141300	0.5660783375239477	0.3272474199556938
0.2837326877431559	0.4328612503469526	0.3186532890334159
0.5791934854693243	0.2834353940327658	0.3158327953160919
0.3848169477477573	0.7002054491404283	0.4339816354793099
0.5463789429158712	0.4860163392554888	0.4409317640399026
0.4833074101916586	0.4493440282399369	0.4401310874331393
0.5132329634228514	0.5513380831560537	0.4400897206980753
0.4500741195383851	0.5146874155856174	0.4390754671204297
0.7012779182391241	0.6041396595541227	0.4464842539357132
0.2964737611962435	0.3937808288041014	0.4403043617749326
0.6141152056044454	0.3018730334108932	0.4374621051257172
0.3513271086091861	0.6769795093069950	0.5561779440537498
0.5490283256506798	0.4961807203684374	0.5611610646837274
0.4938226999335991	0.4484640222018330	0.5600734319406553
0.5042477827708783	0.5542712743130987	0.5607247243215946
0.4490326543695193	0.5065451652649464	0.5598323458195087
0.6797960785829682	0.6410339247281153	0.5660481744318789
0.3201463595024833	0.3590038474052018	0.5611231270954619
0.6468826729424381	0.3259605385833190	0.5581993848756092
0.3238721712177841	0.6466398332744153	0.6782464841329482
0.5496971774844698	0.5042800392353654	0.6839727282977832
0.5035662747247025	0.4473969407742167	0.6832000244864906
0.4956773434705936	0.5534072056363365	0.6835621611217598
0.4495258642305499	0.4965279821266088	0.6828607606009923
0.6525732515401149	0.6711385593052981	0.6865768879101031
0.3481984412228290	0.3281816052114710	0.6823121153349482
0.6749256060484404	0.3536594325985362	0.6796606454403259
0.3058336096897983	0.6182389970912288	0.8034500635777008
0.5492087502114074	0.5120120474838944	0.8165871476999862
0.5142776819439767	0.4481183329678247	0.8154182649861463
0.4869414465239539	0.5507024035483301	0.8161029858829967
0.4520086637043850	0.4868047759926967	0.8144723499980292
0.6248878590143214	0.6919474199797927	0.8138922370029490
0.3788770743250328	0.3050034657502025	0.8137327544555858
0.6953873736355840	0.3806337743443944	0.8040632554157514
0.3802826747064219	0.5528526977277505	0.1849302478308842
0.3748550336303453	0.5025698858379837	0.2381303365951769
0.5794266080402091	0.3945552327957814	0.1878779661953973
0.3966009803637703	0.4189783431177957	0.1880107682585910
0.4495820066939730	0.3780228292648614	0.1863541524733941
0.4215891544270241	0.6057245698443087	0.1819243895197716
0.5515923367021773	0.6222788152449522	0.1847324483980341
0.6043735621640147	0.5813822618171378	0.1898142957639374
0.4761437662709463	0.6245990878187150	0.1301532680700138
0.4641509854839524	0.6855945373543774	0.1282047793352299
0.4831067596130512	0.4792219173988844	0.1856568944203230
0.4777396800061280	0.6914301096517460	0.2313106074180761
0.4916869374181771	0.6309811640954595	0.2327546086479961
0.6232575731318182	0.5000670145057391	0.2440710332137792
0.6832047302283643	0.5097244849142315	0.2513517843369323
0.5179052209153714	0.5211489470581513	0.1863174259187527
0.6904882384193185	0.5402024885374570	0.1516178401008534
0.6304653702806420	0.5310761665500127	0.1447752078204430
0.5135508871541367	0.3708524735068882	0.2368034940654740
0.5269316987239060	0.3102536670737547	0.2359755878259355
0.3129355358041517	0.4573572785475428	0.1442888122238485
0.5323946506720220	0.3129936250854502	0.1320326068843544
0.5206787383474508	0.3740702043366034	0.1331949361487270
0.3731295905232181	0.4670541070309811	0.1400005703131038
0.1276703310816394	0.4373930313558783	0.1921671137106544
0.5893624484798606	0.1263892786278111	0.1796883693540173
0.7376569809964745	0.5296885343569590	0.1828373262773500

0.7934849467380422	0.5228131677433154	0.2703577889397670
0.8541746638336417	0.5279076546153192	0.2612527683996024
0.8752740576380464	0.5514182951821681	0.2093886960748647
0.8356023895095569	0.5698809861926478	0.1669685411163353
0.7756030326866604	0.5645369577742452	0.1758580931007130
0.5459371246282364	0.2648877460938540	0.2078032177075057
0.5284235834640695	0.1962902034715731	0.1265775358059592
0.5502853856073030	0.1380526858754626	0.1344770058858954
0.5829586100165967	0.2294623795332580	0.2100876487408651
0.6054938110195465	0.1725531112743958	0.2171577262596968
0.2643907435965278	0.4664280958588935	0.1728040033718296
0.2171207355595259	0.4934891638510863	0.1766179811821263
0.1583231177665713	0.4830124828759927	0.1639370420708697
0.2154274146583670	0.4134015730224069	0.2466363829177750
0.1563088213052463	0.4025499262494545	0.2336376388569051
0.4552019891070671	0.7356589805237590	0.2032422656314895
0.4184917182963737	0.7714832864166676	0.2057600595719074
0.3969776223921838	0.8288325636975368	0.2126161169357976
0.4713682336873492	0.8026825099968643	0.1205295411095964
0.4505492143655027	0.8613512191866477	0.1281850580287569
0.4128366643799165	0.8742292536996009	0.1741861508917064
0.6206604332193427	0.4474846785213744	0.1915440328546837
0.3147279608198045	0.4924101842293989	0.2428778596164799
0.3727352102539552	0.5275943536999705	0.3157184637522048
0.3752957932917015	0.4805053070055891	0.3662528873900959
0.5993005848361581	0.4129939511974779	0.3185069822357610
0.4145545783877913	0.4004075528743732	0.3166903690887209
0.4738420759352644	0.3701084237026111	0.3149013076294818
0.4020692974305700	0.5879609984806838	0.3136910859240855
0.5274850683110841	0.6308826730018355	0.3160969622609064
0.5866263267158844	0.6006580567678730	0.3210630398257350
0.4406327066355237	0.6138455165521962	0.2647439637494663
0.4187815042279756	0.6718254947289218	0.2625878262911748
0.4890445298327992	0.4756385741161914	0.3171654722960250
0.4542658568005601	0.6893392910274512	0.3592052782705913
0.4774395671045996	0.6317562429975959	0.3607504137010125
0.6243479528396846	0.5183146854801669	0.3710898505742006
0.6830705805046100	0.5352261710536177	0.3747346310420026
0.5123422236964634	0.5253316849396441	0.3172785501384429
0.6768761127009566	0.5785788243639169	0.2796375480100092
0.6178708649036895	0.5624720011561232	0.2764207483902977
0.5205730281082642	0.3690670229970978	0.3620907687673429
0.5431795694986745	0.3112166174874974	0.3617229358552892
0.3260698186455535	0.4234517397235081	0.2711397771470948
0.5854289536332677	0.3288713868137491	0.2678009729004013
0.5639994087880833	0.3870770895125349	0.2687868777208750
0.3849758517083786	0.4405166433967673	0.2701408722191443
0.1364245139288060	0.3964726959711347	0.3236603716478310
0.6396855974490427	0.1420023084285370	0.3104516906421580
0.7296432564622304	0.5622232356913690	0.3056117634882985
0.7839140762056421	0.5757193666389068	0.3962175275481717
0.8440356847288929	0.5871677310079425	0.3888860322242165
0.8654925889292912	0.6034821678759912	0.3346308205113475
0.8268205125970590	0.6076324278906315	0.2879746833535805
0.7675691010914998	0.5955285750243123	0.2951293508795171
0.5872609455955418	0.2756947136060617	0.3381086755082571
0.5599080090322511	0.2004220476442563	0.2701084932330855
0.5871229508526478	0.1450144464307651	0.2791874898740687
0.6358548392770303	0.2488885066930153	0.3260923319611550
0.6640140450042354	0.1942491095345253	0.3335918554200525
0.2723862071653705	0.4357130156534593	0.2967069630158954
0.2226510352817858	0.4580642865473183	0.3093729993126976
0.1627902773324058	0.4481884341566930	0.3016253528982493
0.2303941960615991	0.3657165050740471	0.3630419591034912
0.1704455941540290	0.3548682804372021	0.3540170864701768
0.4128723936159138	0.7248866404634015	0.3335089504006735
0.3639869619932041	0.7506459862498074	0.3211171524415536
0.3350160186197907	0.8048773377242061	0.3283874463571251
0.4395604000887507	0.8001482228986899	0.2654867535654353
0.4114363578749698	0.8551500018657207	0.2742497263616509
0.3586476116433393	0.8574129951707994	0.3051976558243504
0.6285328224924814	0.4732947455250084	0.3212775110505747
0.3167873876883425	0.4624717083889316	0.3677257464242509
0.3674814048576558	0.5045545903291819	0.4383589758844281
0.3759154945896484	0.4601285948875468	0.4880608710902392
0.6100883690163661	0.4316321006687348	0.4403930309396208
0.4305927186324071	0.3863254852464033	0.4411960920748434

0.4942758374418818	0.3673494415388509	0.4393341636935145
0.3864188070640567	0.5690546330008208	0.4361799971640855
0.5022598864676533	0.6333022833592648	0.4382104839306749
0.5658214748805553	0.6144244800017158	0.4426064261000425
0.4200461197352269	0.6013171360375551	0.3875037484798410
0.3893287484008855	0.6550063089747421	0.3853293431747945
0.4903460040927984	0.4740121115714739	0.4400522419380852
0.4217985195901666	0.6782859387648188	0.4818214574912766
0.4536545328963665	0.6251530560110885	0.4834219860357014
0.6185505333176337	0.5402180562913124	0.4921674214730513
0.6734237968325640	0.5675680695968767	0.4945290050287441
0.5062539275676682	0.5266526220341170	0.4400302901392383
0.6576935345337653	0.6086619280104846	0.3995786996325233
0.6023603065818696	0.5821397221697407	0.3976619726605150
0.5423937392565801	0.3752248360657354	0.4854811628817052
0.5752587011462732	0.3226924309471795	0.4844128187159273
0.3413424853706326	0.3908334924908769	0.3942662759308840
0.6093205909941790	0.3467491844373861	0.3886453356430907
0.5775527508831695	0.3998368838249383	0.3902772544454350
0.3961908003238749	0.4185210428091738	0.3941919538762220
0.1587632487062461	0.3290713893325570	0.4448450130409687
0.7042978396592656	0.1778877920265643	0.4310258187731330
0.7134310474151102	0.6017884451299697	0.4248510122418442
0.7647690400354390	0.6269715683515474	0.5146084115779296
0.8217478252103820	0.6492680026115967	0.5065987261201448
0.8394456715241031	0.6688034208560234	0.4520244550661521
0.8002716098823393	0.6652919609801563	0.4057231179066342
0.7442788614841293	0.6423179910837882	0.4135143356531060
0.6240591104315395	0.2962557526699877	0.4596194117096267
0.6133853454219599	0.2166101528860585	0.3913179642117318
0.6523629098236762	0.1689394707017141	0.3998005327896182
0.6766321550315885	0.2808832869730366	0.4476999566510920
0.7162818919856832	0.2341292770801841	0.4547285528459901
0.2855592407050652	0.3934001660130753	0.4179529022371875
0.2318808376267834	0.4058674105309241	0.4288771677628349
0.1752286980891767	0.3840305169111271	0.4209196130750681
0.2559323235997245	0.3192982212144085	0.4866663065997993
0.1994567120552167	0.2963226100182985	0.4773075237471883
0.3752456429607262	0.7066012644804147	0.4560730485345500
0.3238758500375010	0.7249000384451301	0.4450547100085913
0.2867567078240049	0.7737834449162301	0.4516584276911355
0.3890244660801959	0.7843797229377875	0.3859279262331127
0.3525866229235626	0.8341721162623246	0.3938612060901321
0.3009770141220827	0.8287264396877329	0.4263400752555798
0.6289611505028141	0.4961118360841477	0.4429331542445604
0.3216525576524629	0.4314530617516167	0.4887189916881944
0.3698030014985916	0.4807626922951426	0.5602293191880096
0.3877510697783121	0.4374040652327741	0.6093005377432279
0.6216405867780603	0.4549095654931024	0.5609041813942428
0.4532968551223693	0.3767186958205681	0.5605838684397884
0.5193197455675728	0.3698915339280102	0.5589132074020906
0.3764045769610112	0.5477733754048748	0.5588131456947567
0.4788224660130870	0.6328676214662147	0.5593085998668113
0.5447581252554485	0.6259814045838255	0.5629385135000206
0.4024729825376060	0.5855498112926764	0.5098242443240171
0.3627152316142703	0.6327325417359467	0.5079221413309761
0.4964025489118003	0.4740101905592627	0.5601960278917835
0.3926272435306374	0.6627872753954220	0.6034533592588951
0.4333363718733294	0.6163071369799342	0.6048044241410045
0.6098356004220017	0.5633053658450736	0.6122984459498905
0.6589234621112052	0.6003266513737725	0.6144415951350979
0.5016668952586546	0.5287229994383034	0.5606183946641129
0.6361978109683722	0.6370689907987667	0.5192002799802621
0.5864253968721755	0.6008904727397810	0.5175579624153335
0.5641778421425981	0.3866165207641687	0.6057608050220922
0.6050399450919995	0.3402044199803801	0.6050208552572356
0.3635124771162945	0.3657684756469842	0.5140371204168828
0.6361060237533911	0.3701592560773444	0.5098052778845158
0.5961244138855646	0.4171996381481859	0.5110737860903524
0.4124785161539400	0.4032198003370238	0.5136975135124888
0.1954617997413333	0.2713966894323546	0.5673278053169440
0.7513946399749907	0.2142624198507079	0.5513519698678853
0.6919170703481997	0.6410112690428112	0.5442863491554586
0.7376226745799465	0.6765346944985408	0.6343338299335710
0.7898116180785794	0.7087262728169452	0.6262602779871959
0.8047670529386471	0.7293205874928674	0.5712418938036689
0.7676872984581877	0.7170584755110160	0.5246470077946788

0.7165784669632900	0.6845738300812728	0.5325540155157126
0.6573379664381628	0.3214764114841888	0.5803624011588853
0.6560200412984125	0.2411439535215647	0.5126469590729644
0.7004153481472808	0.1986611756283742	0.5210482130780597
0.7120836322454372	0.3134225892198987	0.5672903197689385
0.7570984093596240	0.2719188649900248	0.5741447619688460
0.3085997984286293	0.3574615495032407	0.5391173274722453
0.2543978197293171	0.3599464707635190	0.5516014348779926
0.2019118422307674	0.3289567683216664	0.5445085028991588
0.2940849228876069	0.2773711455430707	0.6064772888215120
0.2419265027423376	0.2452934200365203	0.5979052242998686
0.3405220846282356	0.6812040190651607	0.5782041146449836
0.2866558463995605	0.6891594317083742	0.5662381572965486
0.2410677422174705	0.7299642739173888	0.5729387085619094
0.3405571625824010	0.7611403218840316	0.5091941170017321
0.2954403112597228	0.8029374928394105	0.5172245552622363
0.2452668413609904	0.7871212331254663	0.5486869893922108
0.6282635620913237	0.5218599524030285	0.5632923879493911
0.3395923902105245	0.3990924162858510	0.6101769266093587
0.3759409438443470	0.4570812857809968	0.6824945679087753
0.4000668737262521	0.4176346089003869	0.7313224471016122
0.6282836804867067	0.4765445178784196	0.6831444411722211
0.4760743402365690	0.3695130915989707	0.6837626456021569
0.5422409554279457	0.3745952745229491	0.6820961104903819
0.3709332490863680	0.5242277025770992	0.6812102336914017
0.4570308287602245	0.6262183593483371	0.6824929192756705
0.5231259807893123	0.6312715719357518	0.6858145806740908
0.3910980728074426	0.5665377095111941	0.6320360481801081
0.3437587804038971	0.6058029213180619	0.6296765896011092
0.5016626668589775	0.4730052419939248	0.6832566899010295
0.3663794584461231	0.6398823740618214	0.7259021095116627
0.4144067609262947	0.6013376679548185	0.7277808935185285
0.5988491622299524	0.5817007935390092	0.7338428473686975
0.6403605238602388	0.6272869916756929	0.7351414071695724
0.4975945273883778	0.5278008001855863	0.6833590218912472
0.6099355674838494	0.6586261788028868	0.6401523989966423
0.5675855241353962	0.6136992420243076	0.6393736348179037
0.5841097610961326	0.3991883846797649	0.7286736919563478
0.6319536407250510	0.3603347150760204	0.7270632300476304
0.3902930821678757	0.3428367514987241	0.6358723570864818
0.6560523961545276	0.3952133345263869	0.6314573875014866
0.6088678528712582	0.4346985738043849	0.6335231955601810
0.4320338620988926	0.3884199022779824	0.6362400001876727
0.2432950042949087	0.2167383335358196	0.6857105321274130
0.7985971961719441	0.2647873825479150	0.6722506656619618
0.6640628728081163	0.6737917339082794	0.6646474046808666
0.7041167595833491	0.7160631848081751	0.7544320290736178
0.7492108579065119	0.7578170155730727	0.7460227857545954
0.7585746616743928	0.7821481334042089	0.6912284671654794
0.7231680463400939	0.7640628724026475	0.6451642732017125
0.6792360486375508	0.7221200385462342	0.6533479384068495
0.6864254569422075	0.3513807634155517	0.7015910097493770
0.7003604615530583	0.2729363404541426	0.6325199619412513
0.7521296939057390	0.2400636894827312	0.6406565119740868
0.7406142845381128	0.3536824701601040	0.6895009777790654
0.7926423992937002	0.3217863484078001	0.6964117983661656
0.3380798794702993	0.3239868435211358	0.6599549535270822
0.2828420963747798	0.3162135325229530	0.6723754038449852
0.2379048764289401	0.2749095814125030	0.6640931486133828
0.3381718530035400	0.2426911438229217	0.7261844486551221
0.2939051293147049	0.2003781572347980	0.7162346950506663
0.3126183258973103	0.6489011966123092	0.7002926917123085
0.2581985935497228	0.6467520822879123	0.6893490695836216
0.2063785327797162	0.6787846422445555	0.6969865353161188
0.2974566062142060	0.7269559916159214	0.6310164467955308
0.2458958810453135	0.7599437023136371	0.6398093185233571
0.2000006970145403	0.7355859823223422	0.6724658312062044
0.6233133300453498	0.5436578644457859	0.6848543254015673
0.3593194127491788	0.3712984689803339	0.7315435088688410
0.3870674959936652	0.4339531579394704	0.8143451982608879
0.4298327536556878	0.3926697194831459	0.8670340294835484
0.6315249215144528	0.4996221010922621	0.8153381558039990
0.5001825951680273	0.3666307902960650	0.8140825386865610
0.5646828308980149	0.3831907660672665	0.8123906932278206
0.3697127963037559	0.4991792443752581	0.8122351611084595
0.4365859690889404	0.6156631046595250	0.8131999515493672
0.5009907076653840	0.6322033214480333	0.8168706709768505

0.3851548963487918	0.5476528866714331	0.7609384103816705
0.3336457115501388	0.5806432093239084	0.7555589137517090
0.5082987600671666	0.4730686338928299	0.8156469095279424
0.3438667325977791	0.6133753043999590	0.8543884713682087
0.3955889544857285	0.5807534961155858	0.8593423822773784
0.5753246543298774	0.6043472210210531	0.8689385489700797
0.6105738665210882	0.6553435862396237	0.8680241902784798
0.4929297263252576	0.5257526055154614	0.8159338441952179
0.5920772390103343	0.6658444308768402	0.7660551950750957
0.5558338224833459	0.6159389747547150	0.7674112677228572
0.6054929349689669	0.4175220214876904	0.8602024569837194
0.6572183664534863	0.3849047199479753	0.8550281278489998
0.4072759076125730	0.3332783159428110	0.7639752795667509
0.6677607503887255	0.4188653194635917	0.7566073411537939
0.6162929784409217	0.4518664420761118	0.7622403922237514
0.4420444060077279	0.3843688743059125	0.7641393173836760
0.2819525723193433	0.1846635617797907	0.8166678202443676
0.8207820763145391	0.2952627915020427	0.8043728265334349
0.6311632128473421	0.6978314191553572	0.7906485003248966
0.6796690543821102	0.7447144830512681	0.8745766153850880
0.7118633712534022	0.7967580738254753	0.8617974803614987
0.7062565152079597	0.8234238199882988	0.8075379857265133
0.6693006028474308	0.7976102819629093	0.7663147258303480
0.6383188343445858	0.7461557284924464	0.7787033594851078
0.7048793877651803	0.3741053312313295	0.8259350420363891
0.7306183957419695	0.3084782406686568	0.7502689478395131
0.7813079190616111	0.2753257503101613	0.7622946182577172
0.7588156620528781	0.3810422815157699	0.8224326682344827
0.8093595807736844	0.3483513270256906	0.8341741232555200
0.3713791893991901	0.2988051763648913	0.7909494172832556
0.3251013912658529	0.2781699655092315	0.7863020239898518
0.2849354542564835	0.2326177166404339	0.7781049574052706
0.3597357830086630	0.2300701607330847	0.8732110567868201
0.3198035637734264	0.1829947928424329	0.8641943302842543
0.2964465993670764	0.6246181627072948	0.8254057473678069
0.2425733646964849	0.6175706346552498	0.8223570854285676
0.1920995115093139	0.6501201664045179	0.8346667844083850
0.2699259406484905	0.6904163073486204	0.7501458008561332
0.2193095117810843	0.7234447479581569	0.7627665554308550
0.1802816199054121	0.7033011757767502	0.8051515670749835
0.6142088938135655	0.5647734816353431	0.8181422352697963
0.3964994911221536	0.3404687056190852	0.8673279644623579

```
N-CENTERED HEPTAMER
   1.00000000000000
     40.4128619999999970    0.0000000000000000    0.0000000000000000
      0.0000000000000000   40.3914600000000021    0.0000000000000000
      0.0000000000000000    0.0000000000000000   48.2582340000000016
   C    H    N    O
   504   350    56    28
Direct
  0.1811139292801284  0.5891279518328386  0.1834321275890624
  0.1968179390489108  0.5374730087476757  0.2196444111801652
  0.1638242131257844  0.5455186000791796  0.2145830486033017
  0.5454741064942147  0.7434167414954751  0.1486258811602092
  0.5458563675462950  0.7799812991885454  0.1543269415130873
  0.5293249069487731  0.7949650381978425  0.1766152712340389
  0.5295829863792535  0.8293810951450125  0.1794842731500585
  0.5626631776359421  0.7999637728707161  0.1351223033635497
  0.5633343883614549  0.8342276576476432  0.1382950027275361
  0.5466445206319560  0.8490534483574530  0.1604502954902996
  0.5430033317142741  0.4020064166235324  0.1820441640767407
  0.4555757931126302  0.4389041301387048  0.1785477301320461
  0.4251924642204994  0.4859365207965496  0.1801890066239608
  0.4028124269821650  0.4583442826176956  0.1802549318892986
  0.4212650293265107  0.4298185311124064  0.1789641468779559
  0.5774736353829981  0.5143304648433913  0.1827013028315603
  0.5472987967505888  0.5612979508817986  0.1786198543033316
  0.5816257446933289  0.5702992701868178  0.1792411631459787
  0.5999307187822760  0.5418316745477579  0.1820552051141428
  0.4404458633528106  0.5459146257957551  0.1802827933086502
  0.4310718446706984  0.5807380076003502  0.1795535962467653
  0.4597451913538539  0.5981956683681710  0.1776887731948360
  0.5621673365735905  0.4543855674089885  0.1841275960588076
  0.5163188927434962  0.4262555284953002  0.1805218143881523
  0.5715708844180973  0.4195448223268576  0.1844751060321020
  0.4864700376227918  0.5739104942900987  0.1774848056648255
  0.5202444298578379  0.5830580492874979  0.1767811393074018
  0.4171901340340736  0.5198124117448789  0.1812176889997743
  0.4826471041717190  0.4170851179221806  0.1786244340950038
  0.5853826099360797  0.4805216167650376  0.1849910727776817
  0.5278338026858657  0.6192021919039862  0.1745984512026374
  0.5242390019053140  0.6360282818340217  0.1493496856097921
  0.5289755659368204  0.6701296213953658  0.1472800024991269
  0.5370024695243476  0.6884919486664572  0.1710969040894229
  0.5413676640308206  0.6718242582109816  0.1963918808739054
  0.5371113571524733  0.6376722234228597  0.1979649581247165
  0.6211614187005137  0.4722093010269404  0.1887722017999974
  0.6330666475163267  0.4619268017878426  0.2147101252945343
  0.6664786292533518  0.4550168139477411  0.2191264951652041
  0.6889449652392954  0.4580613580496732  0.1971566500411076
  0.6773474038700994  0.4677636628542509  0.1709692842771390
  0.6439475991233066  0.4748369635465469  0.1669141577946830
  0.4748318054156971  0.3808667560764410  0.1775574019034636
  0.4706343721666835  0.3626230271428199  0.2020277379618152
  0.4653455213858659  0.3285878109105632  0.2013380560136991
  0.4637084598667077  0.3117645807513638  0.1758852600506336
  0.4668577008206423  0.3298887107612132  0.1511508111419121
  0.4725152385503016  0.3639426855830064  0.1522732659833458
  0.3812511142426793  0.5281695998643208  0.1836032475185703
  0.3595697346209469  0.5241392276902940  0.1611174992857271
  0.3259475955286972  0.5310157524422807  0.1637929304120460
  0.3129866257771226  0.5419107011318748  0.1891863521709909
  0.3343365223928399  0.5463887161115260  0.2117515176011623
  0.3680142249071426  0.5396797703090611  0.2087260058698649
  0.1558845110748602  0.5713581723898854  0.1964843462917402
  0.7427919388850653  0.4517397343611079  0.2229290746879002
  0.7779543217709191  0.4411038427815555  0.2182377296163707
  0.7945052582090348  0.4258426483169159  0.2403686396968516
  0.8270877634342059  0.4151380422450727  0.2373250380014383
  0.8437442945494825  0.4200154113380294  0.2122735069138468
  0.8276655082616576  0.4357354287409710  0.1902760086245651
  0.7949130589502594  0.4461215661689941  0.1931849438093082
  0.4500362034732349  0.2567506830068209  0.1553372618012637
  0.4510340818743506  0.2201916680791625  0.1610939870342581
  0.4316357788380553  0.1998649649510311  0.1439678236173807
  0.4321794001836242  0.1655900153254080  0.1470933886954050
  0.4712875320935210  0.2055147067728274  0.1812716232920320
  0.4721406058040621  0.1711053462325251  0.1841012923970575
  0.4525677336438807  0.1510925608342233  0.1671231617686289
```

0.2574060593983938	0.5453831739738640	0.2125177232015610
0.2222916638707789	0.5548508182457289	0.2062931044829200
0.2141592871816800	0.5808502451975255	0.1882014029079985
0.1732665853153083	0.5463935939644404	0.2939059490493333
0.1928837820650307	0.4895585671045670	0.3224939868466745
0.1593494681408976	0.4963568594180016	0.3188675958688671
0.5083868265350672	0.7474981939388868	0.2605375496068909
0.5030780424289297	0.7837825589126381	0.2654544901053061
0.4753009371460125	0.7959497569826963	0.2796002136028915
0.4704938983510848	0.8300329522408503	0.2823334722427993
0.5260944173454792	0.8061572722514830	0.2543634368053861
0.5216325293434783	0.8401412055826507	0.2576887214165807
0.4936935524806677	0.8521726853579419	0.2715289268146998
0.5624733673470855	0.4126711555295289	0.2934288710526476
0.4691277961163777	0.4297310921170813	0.2896599437446434
0.4299743229339841	0.4698044053300454	0.2907553549555156
0.4135601519073565	0.4383129973751416	0.2904835112699204
0.4373033967355250	0.4140246484408101	0.2893220177803999
0.5718050506823613	0.5300500949509608	0.2941763231841995
0.5328504137281018	0.5699545901739793	0.2896201077719915
0.5646136132960724	0.5857171700386534	0.2907882617031853
0.5881907758571208	0.5615444784510681	0.2940362719060619
0.4322032594410491	0.5318374394787519	0.2901710090364040
0.4153681529538145	0.5637176895478253	0.2887939085773493
0.4394717721393043	0.5871028813558918	0.2871586200969075
0.5695953608979140	0.4680311344065499	0.2951762568887783
0.5311344936990910	0.4303843462936313	0.2915082193526110
0.5864445209933760	0.4361202760787884	0.2955911527652347
0.4708826236714070	0.5693624753763605	0.2875149685741958
0.5018767495569524	0.5856181012701753	0.2872585129982320
0.4150990790708752	0.5013062308541720	0.2912935603292329
0.5002336388007873	0.4140452619466582	0.2897625828765355
0.5866230460751406	0.4986181811900098	0.2963145696012184
0.5021241924690917	0.6224812568990911	0.2852662205839142
0.4910794503388388	0.6387613128609775	0.2612577071492276
0.4907821072371663	0.6732110749013114	0.2594847611327056
0.5011688421105535	0.6922496907784674	0.2821547955997277
0.5122849376522769	0.6763520718095136	0.3062220780962453
0.5129325529599691	0.6419384862748051	0.3076008692133094
0.6233425530521390	0.4976801049694841	0.2993439685955143
0.6382039638857689	0.4843396907401152	0.3231813111641066
0.6724915888786248	0.4821354883480700	0.3257844606517125
0.6928333644653034	0.4931835910879714	0.3041839526655646
0.6784085152818984	0.5070197150385062	0.2804494648089791
0.6441527755153393	0.5093170805306997	0.2781648895645198
0.5003574967789670	0.3771251803955504	0.2885641413084435
0.4873176691279171	0.3580455917963782	0.3102945434381629
0.4884766111106976	0.3235922076955305	0.3095476834206908
0.5024174843383310	0.3072888718805399	0.2867502209462397
0.5151257231093176	0.3259403522059612	0.2647044314009206
0.5142594347728549	0.3604015499107070	0.2658256705436259
0.3782198655003573	0.5023592587919107	0.2928347937188849
0.3585151617874585	0.4909744797358179	0.2708516271511943
0.3241369552857909	0.4931317240488982	0.2719748089464069
0.3085592335713000	0.5065411923052207	0.2953575902608158
0.3278202895547739	0.5174483928408057	0.3176864929125839
0.3622082405219133	0.5154735168889550	0.3162410273024498
0.1494453416930670	0.5248827296101344	0.3046774149256382
0.7481294859280131	0.4928489924417359	0.3269264588734209
0.7842075929599311	0.4866134032529192	0.3215865615196679
0.8035569985124987	0.4754067292401192	0.3438406629286028
0.8372917000422537	0.4695592057803195	0.3403708142881894
0.8522579306223954	0.4753523658347407	0.3147024655487943
0.8332689087906893	0.4869206127675503	0.2925305321023925
0.7993893432731011	0.4923656275970398	0.2958718428749910
0.4965563917756881	0.2518450631924435	0.2655582562039006
0.5024799488003330	0.2156727144081504	0.2705705221627696
0.4801414539649557	0.1928340100809446	0.2591746189708710
0.4852430696223966	0.1589467388592500	0.2625630849150506
0.5302288751418770	0.2040717163337522	0.2850887716031484
0.5356863091127955	0.1700968621800305	0.2878942508512639
0.5131450272248252	0.1474916481984450	0.2767890641463000
0.2526846000639844	0.5029064369749385	0.3165174384839734
0.2169268969167049	0.5108062210948571	0.3113933920525399
0.2068849495951463	0.5392693760721886	0.2969935659778055
0.1685967746269221	0.4875105915256724	0.3959973231517364
0.1975946381119131	0.4368486645442389	0.4269907496651921

0.1634142165043872	0.4374393273701705	0.4231549319049826
0.4629578109748178	0.7446163748644011	0.3650054012131323
0.4500501200694006	0.7790121519115210	0.3694833412513441
0.4215190617537513	0.7854591190000036	0.3851527403770630
0.4095181153574490	0.8177574429480573	0.3875256273854353
0.4664583989989698	0.8053550860564449	0.3564261829098276
0.4548342432129974	0.8376511803732356	0.3593238208852690
0.4262148510827568	0.8439144364831099	0.3747597519885369
0.5747241941476039	0.4237942408365051	0.3982094694068329
0.4797623337485405	0.4256994689631133	0.3958967443496563
0.4345520330740684	0.4587743921570128	0.3973302984820937
0.4235380145694477	0.4250229861514050	0.3970138583607730
0.4509446070369318	0.4049809573801908	0.3956685530490859
0.5650526365536009	0.5410725055663367	0.3998643851709281
0.5200403797325276	0.5740990537139765	0.3956186000266839
0.5488788319146632	0.5947843483282456	0.3962529853013276
0.5761168754272156	0.5747919428089258	0.3993232066166567
0.4266527546400098	0.5202957925543485	0.3967412154903150
0.4049497298758521	0.5490660914562374	0.3953292315538790
0.4250035629383894	0.5760139278015307	0.3937894371837087
0.5728963226371139	0.4795504165397950	0.4006882874995765
0.5409078806246496	0.4362642990503187	0.3969589313981000
0.5946528751116218	0.4507575733585658	0.4004718054233771
0.4588676869329947	0.5635448366583116	0.3940825934385301
0.4868955982614332	0.5845591646505164	0.3934831063942267
0.4146735313562182	0.4874025849934776	0.3977176776686099
0.5129782594532756	0.4152362987895440	0.3953841318731143
0.5848296428240031	0.5124673580048547	0.4017900547792531
0.4810980870179219	0.6209329896871493	0.3910496066330416
0.4661073573750875	0.6344938292856548	0.3673466017662321
0.4596216212697243	0.6682874374107108	0.3652539875628255
0.4677275846503359	0.6894294635418656	0.3872498919433525
0.4830287584660864	0.6763606078160596	0.4108938097929209
0.4897492686528974	0.6426131120818533	0.4126153590770394
0.6212952301999932	0.5174494628023922	0.4043046157739175
0.6386335011951589	0.5064981829045571	0.4278294559150390
0.6728756123347978	0.5098881271331673	0.4299338502568336
0.6907472433192160	0.5241964632847533	0.4081286332061311
0.6737681570434012	0.5355837026353897	0.3846690764117097
0.6395276553176100	0.5323814775837572	0.3829100563893043
0.5189399197896263	0.3788119450142747	0.3936461725975772
0.5100904589949062	0.3576586840012303	0.4155086575013252
0.5169854855150552	0.3239001255330216	0.4144169680785530
0.5326086829883958	0.3103506317345544	0.3911152938510803
0.5409373843395352	0.3309894189294968	0.3688461280120183
0.5343053257159323	0.3647953592111867	0.3703106075147772
0.3781101486196819	0.4822600055919751	0.3985679792464371
0.3614102170137994	0.4669622699957410	0.3764935181623713
0.3271945022092770	0.4628450230770080	0.3769627043824013
0.3087679615522733	0.4736764105244722	0.3998191220489017
0.3250695677160682	0.4886292418157874	0.4221702908625483
0.3592656440874233	0.4929636776770966	0.4213481300618879
0.1488053685059865	0.4628907379786530	0.4077679460812982
0.7454661665668896	0.5339037883209924	0.4302242455424162
0.7820806211473854	0.5339693616724980	0.4247998077459915
0.8031157320359227	0.5278190716905609	0.4473635968498196
0.8373559302716375	0.5279897631352687	0.4439348136450508
0.8510681044192289	0.5348407435911638	0.4179610764465599
0.8303298719718905	0.5413512301100768	0.3954470905826208
0.7960130175768848	0.5407530883911378	0.3987626724152471
0.5376060711075556	0.2548519741978354	0.3696395856803522
0.5504840276056084	0.2205395830838356	0.3745074627848481
0.5342045588733852	0.1940086237835447	0.3616147701488305
0.5458256034090975	0.1617621455954655	0.3649057047865624
0.5788706633291781	0.2143384656721128	0.3904251670027697
0.5908441406391803	0.1820820305525887	0.3932215805362499
0.5742735595322425	0.1557325162413750	0.3806242973396334
0.2541755819885714	0.4602047530331930	0.4204140392440068
0.2176566802772458	0.4612571983147892	0.4149298857603888
0.2029124165919077	0.4865631621070254	0.3993012127618533
0.1751256679017899	0.4338591606315824	0.5008561148468816
0.2125202958928616	0.3890708376048256	0.5318770267266236
0.1786997034404501	0.3838711414108331	0.5283031212986473
0.4207819525227142	0.7352451086217229	0.4693106441137691
0.4022375394825891	0.7669199922019881	0.4739668009963960
0.3725284422203479	0.7681419941153608	0.4889759054863813
0.3553131447969852	0.7979654446490991	0.4915524095621125

0.4145216326539257	0.7959569635619141	0.4617669014746966
0.3976590468690472	0.8258209476646651	0.4648811339022333
0.3679092788419360	0.8268645288615915	0.4796673526770679
0.5868526540227162	0.4383593608251014	0.5008142049588787
0.4929914957306689	0.4237327724056933	0.4982225618032699
0.4427527881241628	0.4485546298865773	0.4997419925849687
0.4377043600485940	0.4134118375058695	0.4995501741533322
0.4681458127126335	0.3983722540892937	0.4981522862564687
0.5570927948172352	0.5522863653435900	0.5023433203637993
0.5070439355004589	0.5771074357872094	0.4982003644283615
0.5318773157518952	0.6024510878843066	0.4991466734246000
0.5621555877779751	0.5874257570280035	0.5021586346611420
0.4244120223649154	0.5078242255302604	0.4993001195025679
0.3980423256139535	0.5324120549219633	0.4980847390398567
0.4131219010364278	0.5624197268161628	0.4964842848365876
0.5754337148322297	0.4929899130189008	0.5030664961927879
0.5513937082151803	0.4447920780724868	0.4994180811960145
0.6018301890887215	0.4683677158797128	0.5030078360884549
0.4486404622152632	0.5560115128157572	0.4966044406793609
0.4725671639144577	0.5816197598114029	0.4961302590862911
0.4182668211151892	0.4733554206430086	0.5004012650040274
0.5275350130337405	0.4192037019054948	0.4978852803840393
0.5815056168336179	0.5274950632576983	0.5042443337973007
0.4603615652819188	0.6164134793486404	0.4940761317311900
0.4431871839282794	0.6274065427429713	0.4704812352138537
0.4306446184707864	0.6594798651581427	0.4687535373666099
0.4347074935138854	0.6813774961005654	0.4910083959499134
0.4519970043671095	0.6708921066899223	0.5145931656820222
0.4647797632090978	0.6389100984517402	0.5159549749506068
0.6165447916284751	0.5387138539352085	0.5069863676058814
0.6353122100832697	0.5308234017846030	0.5306048760052255
0.6684518086172108	0.5400006280787900	0.5329044764240625
0.6837810836105073	0.5571992087745833	0.5112118492050537
0.6652821049588462	0.5656715850815519	0.4876992109601996
0.6321076787839027	0.5566656514104915	0.4857351551090073
0.5397886841223403	0.3843594877935643	0.4964788386165506
0.5345559942175570	0.3621970090205474	0.5184607232845440
0.5472084337244384	0.3301263214131820	0.5176682636613925
0.5651812237657819	0.3192608460323586	0.4945712115667933
0.5700547553752672	0.3408430085669787	0.4722135203336524
0.5576369495328496	0.3730002171461833	0.4733598681041389
0.3831042731432692	0.4621485334632825	0.5017984909634308
0.3686326834255001	0.4444694901147039	0.4798705831208794
0.3354713168122038	0.4351298805635361	0.4806894588409920
0.3159478802646196	0.4429437677063492	0.5037706901312989
0.3301389689315480	0.4600504559444048	0.5260567108637703
0.3632351775379811	0.4696321227157482	0.5248694540083487
0.1598712354423227	0.4063864468583802	0.5129284223883112
0.7359382076715882	0.5758593095749972	0.5335868704418000
0.7719895730263443	0.5823245103209815	0.5282772517158451
0.7936883675434004	0.5801577211479171	0.5509144751791704
0.8273416197625506	0.5865555678007891	0.5475647576495405
0.8396909915135332	0.5956633201225260	0.5215947210492163
0.8182273026029673	0.5981552774710194	0.4989949862018708
0.7845812217587640	0.5913706014158652	0.5022420149207486
0.5792840433936118	0.2650226090256674	0.4736731475151431
0.5980844529259055	0.2335751685386681	0.4787070659970553
0.5860514075201535	0.2041428024288386	0.4670069271359302
0.6034286379254619	0.1745992571472328	0.4703041198488799
0.6279988257922592	0.2330455474179014	0.4934609152686164
0.6457376717258488	0.2035549665007875	0.4961931860033136
0.6334337745306305	0.1742747848363050	0.4847542559223009
0.2642665512626133	0.4215460915044264	0.5248704698462411
0.2280606117528405	0.4164252608403454	0.5195608255067271
0.2091427450405906	0.4387316022435100	0.5038855180612359
0.1912159946680744	0.3776343179642303	0.6053137573461230
0.2364051781209194	0.3395278643687262	0.6353094448036943
0.2040774957624527	0.3283368630155422	0.6316572200685880
0.3811289343067173	0.7186825929593870	0.5732270308654511
0.3580065120753184	0.7471464477710168	0.5781123205457085
0.3287696043554377	0.7438659102588663	0.5935593777496231
0.3071969929363129	0.7706769580106912	0.5963042164905268
0.3654071937512922	0.7776649743465586	0.5656817338785939
0.3441898683727909	0.8045820454799029	0.5689703105942155
0.3149223290440533	0.8011025358477430	0.5841642235035754
0.5953099378008075	0.4545968205556020	0.6049260646184444
0.5055268433373603	0.4236870221167067	0.6024521011243874

0.4518016861865643	0.4395305435839972	0.6040103905925528
0.4528503613616849	0.4040349001892353	0.6038147219287829
0.4854173155468918	0.3944309860761784	0.6024172758177994
0.5460350841131328	0.5615161240754742	0.6064422578588859
0.4925071656332939	0.5773798906459130	0.6023542087417073
0.5126396897717889	0.6066209217258317	0.6030799450656438
0.5450435374011430	0.5969980831005574	0.6060367069068892
0.4234144856784497	0.4947490938231722	0.6037044798140997
0.3931098109183362	0.5142889202098669	0.6025941132385556
0.4026405141692976	0.5464935349886197	0.6009780708925055
0.5743877099592564	0.5063099883223620	0.6072330789286645
0.5592705268496770	0.4546363775746930	0.6035874156061520
0.6047332392071613	0.4867837933952196	0.6071200555351012
0.4387175940971049	0.5464499369363435	0.6009792054144985
0.4577827129216016	0.5758332316850494	0.6003731531615213
0.4234254436201834	0.4597363333939117	0.6047046873882496
0.5403147945646517	0.4252532902776847	0.6020433114263015
0.5743373927893444	0.5413280153975876	0.6083595432711814
0.4398226618933712	0.6080069017455287	0.5982563313084472
0.4211717423473285	0.6159841826010186	0.5746041940183229
0.4035819822362533	0.6455876014734395	0.5727827703941358
0.4039516014390919	0.6678968032254161	0.5950101901163083
0.4226505973781395	0.6604301107204861	0.6186506634263054
0.4404622756752121	0.6309709684740443	0.6200977246089728
0.6069558180344859	0.5583942999148127	0.6109401895430244
0.6265757831368113	0.5544949366571358	0.6347597087454612
0.6577026217367150	0.5691509782649664	0.6369120926130459
0.6701209011870035	0.5879621686567178	0.6148427252573498
0.6506924842094676	0.5923699735343649	0.5910756329443857
0.6194718791678643	0.5779456616043623	0.5892868493228590
0.5585337821371305	0.3931444582492047	0.6005174285008604
0.5576058919521527	0.3704151385378950	0.6225181971599529
0.5757852145623812	0.3411331235309987	0.6215734301153577
0.5951028690062664	0.3336540353093481	0.5982998510212443
0.5958440790134995	0.3557731176601858	0.5759576765693826
0.5779003884789745	0.3851938396826030	0.5772635319434459
0.3907529490747668	0.4425715902070430	0.6060132411308258
0.3794423385946952	0.4230604306655287	0.5838757603082492
0.3483968536335846	0.4081045185076048	0.5846367263793987
0.3279707896297646	0.4120051378672951	0.6078567965599635
0.3391133776612158	0.4308909169146641	0.6303423851761804
0.3700419729466085	0.4460833970102556	0.6292250928278115
0.1813421360630154	0.3474554832111570	0.6167708064605915
0.7180942237596262	0.6160003353423801	0.6369206280260173
0.7526127279132246	0.6282312432752788	0.6315677803097256
0.7742938386807823	0.6300198396465726	0.6542411358332288
0.8064972431642856	0.6416818130220542	0.6508217813411149
0.8172984559947838	0.6522517213751879	0.6247561697310874
0.7957873433321342	0.6508655837380874	0.6021256188938321
0.7636704588538631	0.6387645138404726	0.6054348131981290
0.6179743349870346	0.2826961773109532	0.5771050084281414
0.6412369560874374	0.2543903551109613	0.5820582847736276
0.6336775093740333	0.2236477038934099	0.5700960146538149
0.6550759962623600	0.1968741864109053	0.5733524097169537
0.6707703242655160	0.2580481329305427	0.5970295697065009
0.6925207989481010	0.2313729733576944	0.5997323475760931
0.6846559069410871	0.2007287810740966	0.5880499789145923
0.2813542945302886	0.3810166745960459	0.6287625219455698
0.2465978799044880	0.3696144537959854	0.6235564230305439
0.2237865074148448	0.3885656411381165	0.6084271264913002
0.2173517757324159	0.3266576025216214	0.7087686735006249
0.2683228413683780	0.2959756327056743	0.7382335170760860
0.2383579193672405	0.2795643837752254	0.7342572204583835
0.3444840076788919	0.6942978713704976	0.6773069236599009
0.3161586969172737	0.7176437058883861	0.6820131953408286
0.2876697083726458	0.7083747503947171	0.6967239650715997
0.2612391246645739	0.7304395310318889	0.6993399569083054
0.3178524785715433	0.7493029750646520	0.6701398260701922
0.2917215449016736	0.7715060413937869	0.6732727134790180
0.2632696776487728	0.7620699138570747	0.6877656422921955
0.6022084181386587	0.4729932612946394	0.7099526742674362
0.5193083149233693	0.4266432140963569	0.7072149445688078
0.4636105279892476	0.4324415403978946	0.7089330900448908
0.4710613057737634	0.3977348479076697	0.7084693985378545
0.5048134080409536	0.3942258044689894	0.7069554965946618
0.5347141627789433	0.5693705302711536	0.7112060454260134
0.4791849182060005	0.5752471932114024	0.7072366065086736

0.4937099674380487	0.6076450906534077	0.7077265198724049
0.5273300484019827	0.6040793729467682	0.7106187720497874
0.4258144708097110	0.4816145307879294	0.7088607168968135
0.3925359302865098	0.4954816904011222	0.7077526816506480
0.3962315462215215	0.5288606994476641	0.7061308669932669
0.5725085885604431	0.5202139357122301	0.7121607396351473
0.5667211315847168	0.4666992776768393	0.7085589566697843
0.6058218157486921	0.5063395393967082	0.7121180960314991
0.4317502207171994	0.5352007610650471	0.7061264660062856
0.4453022868264424	0.5674838732756400	0.7053407854043634
0.4320545612902171	0.4471568883120741	0.7096924811727432
0.5532376178671704	0.4344640215139704	0.7068015593941553
0.5662042266297921	0.5546551268575165	0.7130796672088224
0.4218822442190215	0.5959090792779309	0.7029709413077564
0.4023363827468450	0.6002983060695203	0.6791638358184612
0.3797177181019392	0.6262384320707326	0.6770966170624976
0.3759075791921865	0.6483549781164579	0.6992163896737318
0.3954547726578412	0.6445319752970908	0.7229820343857565
0.4182061952645021	0.6187262236426420	0.7247076238665293
0.5952311732360039	0.5773187222411150	0.7153179854903963
0.6154994176220476	0.5771472103349145	0.7389755397079487
0.6434865021937564	0.5971952769151638	0.7408397083967926
0.6521155569871193	0.6177678141027090	0.7185987862123470
0.6319697467797281	0.6184362450475741	0.6949722602616739
0.6038245640244730	0.5986313044933176	0.6934913826327935
0.5768754628044418	0.4061636244567658	0.7048915895830951
0.5801775973568200	0.3833663777335980	0.7266737904889279
0.6031572659181794	0.3577120917152307	0.7253004303601647
0.6232649899995160	0.3540737018678126	0.7018483404538315
0.6198599635005154	0.3762412711859804	0.6797128938852457
0.5970311218276834	0.4020252950141492	0.6814155910563739
0.4029768857514924	0.4244089470263999	0.7107392961220745
0.3953894291177086	0.4034462660734114	0.6884160618906959
0.3674394498499749	0.3832694796182012	0.6889141115838693
0.3465015327787834	0.3832309150283311	0.7120544010878198
0.3540656651419780	0.4035307637826435	0.7347379569020546
0.3818364774275478	0.4239584958662539	0.7338602069400915
0.2127108767549855	0.2949681101457811	0.7196667495622899
0.6945091427746970	0.6538171385491993	0.7404393349527358
0.7265000930183858	0.6716367407269046	0.7349915485792481
0.7476470745678528	0.6770122100446549	0.7576213630808215
0.7774395543690713	0.6938957395254920	0.7541501726810326
0.7862912748096902	0.7060645938019567	0.7280573641275169
0.7652687441259406	0.7010711722499130	0.7054634139138471
0.7356107398032783	0.6838039536026733	0.7088304239266708
0.6549125707724180	0.3081841381469613	0.6802058448145402
0.6831636397120938	0.2848277951207671	0.6850787587157069
0.6815287264645328	0.2530730683094637	0.6733855898293203
0.7076612672677747	0.2309176645897189	0.6767486815783809
0.7115689833002572	0.2942530060307437	0.6998210721009348
0.7379987024022987	0.2722464208455521	0.7026519734411115
0.7360349003315781	0.2405093290290278	0.6912765904025665
0.3055726779674954	0.3447345606338999	0.7326476699048581
0.2732727929059772	0.3276547802729018	0.7270574552685307
0.2476161476704589	0.3428506373350741	0.7121733684797540
0.2552093912390576	0.2770387530165411	0.8127662553696957
0.2996929475590808	0.2658511349285719	0.8559608638999008
0.2727623220985060	0.2447777214555804	0.8523439316314910
0.3153133527410676	0.6654437856770286	0.7826411203290693
0.2857177680297724	0.6867875612125899	0.7884170681145575
0.2605415915414304	0.6772353985445386	0.8068360743665098
0.2332214905404622	0.6977235482329612	0.8112102091977348
0.2828888613319910	0.7170059968247979	0.7742959028040407
0.2557745657267690	0.7376010571753402	0.7789428032367922
0.2308915170389567	0.7280411660516042	0.7974153103448125
0.6059541943976858	0.4921706640596697	0.8208972972889278
0.5338603823705300	0.4304595715321086	0.8184804785619982
0.4780407551077423	0.4262867607022168	0.8198575213083832
0.4915173868682772	0.3934419771664013	0.8190800439546783
0.5253890436623887	0.3959740749701738	0.8179042793346717
0.5205574113982274	0.5733414929050189	0.8230848160518329
0.4648830957807157	0.5692730682602035	0.8190519543106614
0.4733586810020741	0.6037559778925828	0.8199319767291680
0.5071126369787458	0.6062030069727010	0.8227112949651320
0.4314118520076140	0.4673073629202101	0.8194059630536015
0.3959974345097017	0.4740845614975983	0.8181309831778167
0.3927899080155665	0.5075225313651307	0.8164611738502388

0.5672019953386080	0.5322953311418601	0.8234529791715404
0.5725023641065833	0.4787658835653528	0.8197601572908351
0.6026685749376128	0.5255350527214969	0.8232852633551683
0.4262604257987311	0.5209711434832635	0.8169500701798580
0.4331252119963453	0.5552752042574851	0.8166530407398090
0.4443451191596124	0.4348142783263793	0.8203402314242132
0.5656880858483634	0.4445275633217820	0.8180789913785967
0.5542018432757531	0.5647595836955306	0.8246742654544049
0.4048530659718763	0.5788550872528497	0.8135924739088317
0.3883074423038225	0.5821948236527725	0.7881501737321684
0.3625178823064250	0.6047635343103316	0.7845583308531958
0.3524241294488953	0.6245241809152888	0.8068730880328272
0.3683329083580099	0.6210695582958662	0.8325713354479790
0.3942254707334832	0.5986412536956087	0.8358056878937802
0.5786048087070911	0.5924886627475172	0.8266416009212570
0.5944376296102105	0.6001512141449223	0.8516828246012833
0.6191286401684640	0.6242368583226062	0.8532501427729099
0.6289381808948342	0.6410839230233568	0.8291026862387140
0.6127436726941468	0.6340393496814947	0.8039990233479257
0.5878176245661242	0.6103355693820239	0.8029271692960265
0.5941192218061331	0.4210407865898798	0.8155544887420989
0.6038719146968951	0.4008291952094915	0.8377708943996000
0.6299058465877774	0.3784701182750244	0.8349378199479870
0.6468001594034885	0.3756063279209926	0.8096453130574092
0.6375973835387480	0.3958349566246519	0.7873558528328067
0.6116514273949599	0.4182773558756083	0.7905393384214129
0.4200554444454879	0.4068901148782207	0.8213694758827788
0.4108423932886640	0.3898539995509619	0.7972523382288756
0.3865338146445855	0.3654396191714141	0.7979173514213396
0.3710501090675876	0.3569923658815295	0.8230036447089802
0.3808275641704088	0.3731046048817568	0.8474904771247428
0.4048379634219020	0.3978558268549278	0.8463638475119477
0.2503426965362908	0.2504110924143599	0.8307716878782286
0.6690151621139298	0.6807887365719874	0.8502829942378775
0.6949142881414830	0.7062818324924716	0.8432185772752919
0.7119860661480822	0.7202985392294823	0.8656918364070619
0.7358797455737945	0.7446330316148332	0.8614641836641945
0.7430146293731047	0.7555220328048068	0.8346215840439524
0.7262075430823278	0.7417428194902157	0.8121108516117818
0.7023332305645853	0.7172422106574666	0.8163062527179498
0.6840756668875181	0.3349123138938551	0.7855728444969332
0.7137284324682398	0.3136382671644375	0.7912108590854524
0.7164374300879316	0.2833314929691731	0.7772115817610933
0.7437564520662930	0.2629265842004612	0.7815981971493443
0.7391434412127706	0.3234468799369389	0.8092970648682378
0.7666912199047728	0.3031687905545469	0.8133687855804229
0.7689485818881271	0.2727922582922759	0.7996596633488127
0.3338017793286487	0.3147032108626002	0.8439908598083288
0.3049252267293245	0.2924945379108004	0.8377942975089683
0.2824834508156819	0.2979161351470579	0.8160812795285206
0.4059635110361351	0.5903441882453103	0.1801674779773667
0.3844553840369795	0.5431713007142870	0.2264488637712755
0.5399870330812138	0.3752933011984244	0.1812554817065912
0.3760514258417544	0.4603875585737328	0.1811499665175436
0.4123432622717523	0.4044740996026015	0.1786448080291394
0.4627775279636834	0.6248879169993589	0.1766247699318084
0.5906459861701883	0.5955542176593210	0.1778272697938979
0.6266598029351458	0.5397437767058002	0.1833922813932699
0.5172714473501456	0.6221330016213017	0.1308324059810978
0.5263474455195223	0.6826856675462911	0.1274746359386285
0.4776671445789980	0.4871005841756718	0.1793600552085932
0.5471641227486929	0.6860099387459849	0.2150445796123833
0.5401944375234111	0.6252508511853612	0.2178910964640565
0.6157521747356586	0.4594120491470236	0.2319564550298413
0.6751544896757059	0.4475458754674497	0.2395289356218115
0.5250166456812262	0.5132651977129619	0.1808179092565483
0.6946800408811293	0.4700140385744074	0.1536680040092490
0.6353377785814337	0.4824703863301282	0.1464423343387112
0.4719281540419796	0.3752278844362986	0.2220008059691126
0.4631533086064520	0.3146361674927819	0.2206510187583786
0.3094780563125666	0.5276242646873802	0.1460377345900438
0.4649867643538304	0.3172214552193792	0.1313351203637389
0.4755667191649803	0.3776227312059046	0.1329145719594432
0.3692344706636279	0.5154926494214542	0.1412717924305084
0.1300355316057212	0.5777672949462223	0.1926754270597805
0.4533478808558125	0.1241966355358342	0.1693743478865816
0.7344082147502009	0.4432758380623538	0.1820653023808596

0.7813287287267987	0.4224331955434468	0.2598895987564224
0.8395589105992155	0.4029755529392378	0.2546177921280259
0.8692965847582166	0.4117037142607763	0.2099100973902238
0.8407165052961709	0.4400831649570253	0.1708098471746675
0.7830568721139883	0.4590930002271199	0.1759569848959593
0.4641742750987964	0.2665886186139935	0.1956130501124354
0.4165039324719893	0.2117153504551380	0.1280810819924594
0.4168458819429341	0.1500678955253626	0.1337337982845334
0.4870258420957693	0.2206163096370529	0.1946632509284699
0.4883409092096546	0.1599596257461049	0.1996139579736452
0.2676624225547208	0.5531359966624998	0.1718906830767315
0.2337034997089321	0.5954609889237029	0.1784313105628803
0.1750805570812403	0.6096737479432089	0.1696320397804219
0.2032479793314866	0.5176184381553567	0.2339916297691824
0.1442017326223746	0.5316056540909926	0.2248926885551745
0.5389104381419736	0.7339587172782354	0.1903204742139533
0.5156026407291411	0.7800952602134685	0.1916512723898249
0.5162067889868828	0.8408221680722575	0.1966473341265402
0.5748681089341944	0.7878623554958513	0.1176779275266161
0.5767396350243509	0.8494894134873306	0.1233599438397970
0.5468122367128639	0.8759514663977571	0.1627820950857328
0.5966432904182321	0.4099953783134858	0.1860538102422182
0.3246249485217454	0.5547655269755221	0.2315835490099634
0.3887724116344246	0.5675912383316042	0.2890845187158366
0.3770564559611537	0.5243651766902242	0.3336188660710624
0.5656117462357377	0.3859709867464549	0.2931686654697619
0.3869238964563665	0.4349719669151124	0.2908809388375811
0.4333999583020265	0.3874677177069752	0.2884843538632225
0.4364060136945463	0.6137764895614763	0.2859087252733842
0.5685799463013752	0.6122363477341970	0.2894966741642455
0.6147138475663427	0.5649697171820471	0.2959752503688122
0.4825325221492784	0.6241216265566126	0.2436318472684537
0.4823844546154298	0.6853525700328851	0.2405967052934222
0.4814853922986028	0.4810408598564617	0.2905878540127413
0.5196115283514559	0.6911569844200727	0.3241065130858510
0.5213108332059472	0.6299013816210307	0.3265926989889990
0.6225216574668165	0.4753164765246451	0.3399897742865505
0.6834727480591382	0.4717372817380508	0.3444534926549427
0.5204415169413332	0.5186744217523241	0.2912654508390063
0.6942178222885190	0.5152805524755071	0.2634722755871499
0.6332687267300722	0.5195978680714982	0.2593386193080175
0.4767751838625420	0.3704191894937334	0.3283632914917384
0.4792296385589563	0.3090853127632075	0.3269763049368052
0.3091818395359985	0.4850365848322656	0.2544122598811759
0.5258671583059567	0.3134409689310797	0.2468319592936795
0.5247015300831762	0.3747282238128818	0.2487427427727138
0.3703629281161905	0.4808726100809003	0.2523619321806887
0.1231630542021380	0.5304160875389445	0.3021403913447602
0.5173830159695989	0.1209259137974472	0.2790789600810705
0.7377124508982370	0.4817954574195809	0.2860935565588939
0.7916467031898031	0.4714317516378476	0.3638527693367429
0.8519995564601237	0.4605917335320719	0.3578033126408221
0.8787288455177246	0.4709637814229481	0.3119993126817004
0.8448519512977102	0.4917547222458225	0.2724985663639919
0.7850104863529371	0.5016873777235217	0.2783728575452083
0.5098978171222350	0.2620356833820696	0.3061439203490411
0.4588407381736109	0.2020783853292273	0.2476281617249406
0.4674889852225576	0.1413680185136953	0.2539306071926662
0.5479563463395396	0.2215964206927908	0.2938370709845450
0.5576474652290077	0.1613153483568107	0.2988246286513372
0.2636445556565232	0.5168367900810105	0.2763998082359155
0.2253555302611839	0.5562523777943233	0.2885784152094477
0.1657024499899275	0.5688638754492903	0.2830435487609719
0.2009020581420353	0.4677374063592941	0.3340007122059742
0.1408435342924820	0.4794350274060183	0.3273101584614044
0.4937397000954194	0.7378269678553590	0.3007952442594994
0.4570388520501238	0.7787982453078112	0.2880946616679645
0.4485053576251677	0.8392509932874060	0.2929659614656444
0.5474202947797774	0.7964570001720035	0.2431143232772761
0.5399354086783051	0.8573419813597135	0.2493269906760006
0.4899604967705977	0.8788203624416631	0.2737584025593256
0.6129498788826067	0.4322354590042229	0.2973252624164577
0.3158972042221563	0.5276092906984198	0.3360615453954247
0.3780758174255252	0.5486393638104375	0.3955128266105626
0.3717242131311170	0.5048550135013925	0.4387806317444464
0.5821136384953788	0.3979592300245030	0.3974269810027316
0.3978166788723832	0.4173624512287127	0.3975062229549070

0.4514547132891388	0.3781406953462310	0.3948121268249582
0.4176757900432961	0.6018420463352273	0.3925928280550043
0.5485021492858320	0.6215796084535714	0.3947315053107448
0.6017704454505556	0.5824829896519884	0.4008557035340448
0.4593723698061107	0.6181679179768652	0.3502155366421751
0.4481620772286096	0.6782700546905149	0.3466095258117486
0.4834524303767581	0.4783854601640209	0.3970047083960934
0.4887630620270991	0.6926749831871107	0.4282627502623269
0.5011408639531190	0.6327011931351705	0.4313657003761917
0.6249687170350137	0.4950014801563846	0.4448157813306403
0.6857808014033869	0.5013393892967747	0.4484015718001612
0.5162132510076323	0.5214580080664303	0.3976375027112374
0.6876701130322120	0.5462403800967390	0.3674614571479342
0.6267547680913331	0.5408191670554842	0.3642735797388321
0.4983726399650492	0.3679885499905983	0.4339585587378735
0.5110627026450593	0.3079457537231553	0.4319753261733690
0.3144625732333085	0.4518247317337849	0.3592855123536719
0.5526891907375180	0.3205885125808727	0.3504932995176775
0.5412344104373549	0.3807667142877634	0.3530025080231005
0.3754199111386807	0.4587401208632901	0.3584185338717734
0.1219850384150770	0.4635777760589148	0.4050306531980584
0.5835820137813708	0.1304981453782670	0.3829509425823698
0.7367185841925854	0.5203041779858617	0.3895662386246467
0.7920861814097698	0.5229608656574252	0.4675904685079136
0.8533945818003650	0.5229269999355106	0.4616460662545114
0.8779042356463356	0.5352074366135585	0.4152984079634264
0.8408825342316693	0.5469028268712534	0.3751474581933212
0.7802542434563530	0.5460036067671662	0.3809166903136834
0.5482545595607493	0.2671327338347078	0.4102910243007222
0.5124346019324635	0.1990996591650565	0.3489439337031519
0.5327597002594819	0.1412636715587589	0.3550083357858214
0.5919884213581836	0.2347753821317214	0.4003618976042910
0.6131874429655346	0.1775905257493397	0.4053601762237888
0.2632066092382224	0.4744861347887770	0.3799788437253216
0.2181959832361517	0.5059014139043068	0.3900147387272528
0.1573589196610107	0.5075593981223151	0.3841026803412368
0.2092189023521931	0.4175229728744129	0.4394302769451258
0.1480785550478503	0.4180722690565565	0.4323453736711464
0.4520104492867707	0.7329894015537300	0.4057189677014001
0.4082758892491301	0.7651769044667961	0.3951965060238968
0.3870519792796983	0.8224334017337862	0.3994545674894880
0.4883637384363869	0.8000760135563543	0.3439751194466557
0.4680545469565995	0.8580160592531531	0.3493801272244995
0.4168847633257723	0.8691849918652529	0.3767568932187652
0.6214722909857512	0.4511872779662491	0.4018069435182574
0.3109650475339887	0.4969120498651701	0.4401154960377645
0.3716551570697491	0.5273249424516181	0.4984261182778140
0.3739711948326370	0.4832836531192055	0.5422046619963465
0.5986013189997113	0.4141863024738576	0.5001729796858650
0.4136829312230228	0.4014297550276586	0.5001575931483581
0.4732473475014187	0.3720127264317045	0.4974076569513523
0.4013933964727574	0.5865739525755279	0.4953725333651491
0.5268762777011657	0.6288009318817439	0.4979190176819404
0.5860818742971354	0.5994224114888466	0.5039180262469112
0.4395391740460820	0.6104124681494709	0.4531527627697157
0.4175712800611112	0.6675124636726505	0.4501902043154037
0.4875651769105914	0.4762767096849320	0.4991843919675234
0.4545807085626681	0.6876778843034556	0.5321762502913620
0.4776767435700539	0.6309203890431900	0.5346691424307739
0.6237028658052026	0.5170710638123149	0.5474864919252382
0.6824915665020866	0.5337359299593817	0.5514347256687869
0.5123505030592546	0.5245764093938983	0.4998179341208770
0.6773199060403917	0.5786037787431640	0.4705991879161384
0.6182179737612542	0.5628285196880773	0.4670365531742954
0.5210299081777979	0.3704900340341384	0.5367679246883021
0.5439391719996628	0.3135528155611925	0.5353113743781890
0.3242699062919746	0.4224063778211529	0.4630850400388724
0.5836485642282823	0.3324781110299518	0.4540151582813429
0.5618886061446606	0.3897658462978869	0.4559689155259012
0.3834093015918147	0.4386575292489173	0.4615722301985821
0.1332845875883713	0.4025546024806635	0.5104345153997073
0.6472701584821255	0.1512050963116970	0.4869997690632428
0.7298670545966045	0.5609253061299069	0.4928910859796045
0.7836180538447496	0.5734599555435401	0.5711235994857735
0.8439655255753320	0.5845615304674524	0.5653250004875796
0.8660199996165384	0.6008984521434193	0.5189777970385440
0.8276597686957607	0.6054078090192874	0.4786862175879771

0.7681981513592715	0.5936206731015502	0.4843489279867412
0.5882685228155919	0.2796813909398308	0.5140591488008406
0.5629846523383059	0.2048377913271262	0.4552671688591999
0.5936567918717494	0.1517515440578655	0.4613907823869517
0.6377894813345421	0.2558422953847999	0.5024451291803349
0.6692363406446564	0.2035305701156876	0.5073421783592039
0.2706718516664480	0.4363402044665194	0.4842145150898332
0.2208798104337789	0.4602450174324704	0.4943384244787969
0.1605504948054800	0.4516101767618498	0.4889442704401765
0.2273631567056869	0.3720650103530097	0.5442872653090171
0.1669403001219941	0.3622653096579152	0.5376848257895960
0.4113896039959441	0.7212401710959639	0.5097779545474297
0.3625140678138905	0.7456608344373927	0.4983403159573211
0.3319976750875865	0.7985554297937300	0.5029609285974427
0.4374067792951665	0.7946750675556978	0.4498192001821584
0.4076653615577746	0.8483535579038812	0.4556058928015028
0.3545088689287404	0.8502004626747667	0.4818407814321657
0.6281661307275630	0.4734137688815341	0.5044608284064723
0.3152326233177749	0.4659545335426687	0.5441996979737391
0.3680385593522177	0.5046174447478785	0.6029939610834306
0.3783767685771134	0.4610402533525483	0.6467322056388765
0.6111407056901074	0.4328828296419007	0.6042192131550094
0.4312292952179536	0.3881251138990160	0.6044488875967169
0.4949782155616507	0.3693386307355311	0.6016952240873107
0.3868377233714864	0.5682014796175798	0.5998788608870236
0.5032088181506735	0.6317174302610133	0.6017389487401742
0.5666156281552900	0.6128811512450374	0.6075941966705026
0.4204679129267239	0.5986087175269172	0.5572777951427599
0.3894997843705873	0.6513662535112500	0.5541534361801820
0.4913130308606468	0.4744840109926575	0.6034050591033967
0.4224505519204337	0.6774650453044528	0.6361988626492170
0.4545292674026077	0.6252626403911847	0.6388064271389942
0.6172890672101569	0.5395088435945946	0.6519113784101593
0.6724315428890302	0.5658883411858918	0.6556100451720041
0.5065922654491741	0.5265703329159378	0.6041625539784005
0.6605865277917744	0.6064937228424949	0.5736732817244166
0.6048926479063589	0.5811257010386428	0.5704373534610304
0.5430302921751710	0.3761572300142104	0.6409475521367286
0.5757357132439829	0.3242386733388974	0.6392156044887497
0.3394697581147005	0.3939270563701315	0.5669017555825052
0.6105386668723278	0.3499931870457297	0.5576652916739250
0.5789349834321029	0.4024818234812599	0.5598893407629008
0.3949224642808108	0.4202120740399045	0.5654923731567296
0.1558976708640211	0.3388647721629779	0.6141972125428778
0.7016275627488694	0.1798563755629256	0.5902837922821580
0.7149562120710030	0.5993000286163016	0.5964779954445798
0.7654030957120480	0.6221329210956302	0.6745389172379470
0.8231812748653643	0.6426696733704146	0.6686027414437018
0.8424425771966532	0.6616418043467562	0.6220839038370950
0.8040134234404320	0.6591705597429314	0.5817318100069397
0.7472338074182193	0.6379679091813583	0.5874882604162051
0.6247690602473452	0.2985185901025033	0.6174727219754965
0.6108717820311182	0.2211640779960898	0.5581761030468300
0.6487783700857901	0.1729641097980726	0.5642073900300117
0.6771128903827544	0.2819189179677659	0.6062361465326116
0.7156957323297389	0.2346085656182903	0.6110357179622166
0.2841975382144738	0.3983402605993677	0.5884948332114727
0.2313017438898508	0.4121726353213624	0.5993693723961375
0.1735382039360155	0.3927920552035468	0.5938438112651084
0.2541909608605075	0.3251792555804354	0.6473521465232768
0.1965240354080105	0.3046886350590272	0.6406150033815007
0.3746916684693347	0.7035765916726002	0.6137814814002660
0.3225051378735759	0.7201817799091303	0.6031304417818363
0.2842708613374615	0.7677179186295024	0.6080058535888891
0.3879744308666414	0.7798922638509874	0.5534140261691514
0.3504155829924280	0.8283187233073500	0.5594811586417553
0.2981010249161115	0.8220906825195063	0.5864367363016370
0.6297582670278967	0.4964460193769002	0.6085024360956869
0.3235268613929257	0.4337488604399791	0.6486014291224309
0.3695736838375069	0.4815256573149874	0.7081493350501553
0.3872172275011230	0.4400534038556855	0.7514740502774893
0.6216268827866182	0.4544221311124095	0.7092862671933767
0.4526775610619623	0.3781703062387577	0.7090849140432869
0.5187231078563209	0.3712781242043685	0.7060260500402039
0.3768453268148289	0.5474373838328659	0.7050358499002602
0.4799127611896476	0.6306231624663311	0.7062552698531211
0.5456860109238639	0.6236063931632524	0.7120452972861698

0.4048817804399154	0.5829491693324133	0.6619414765380721
0.3649268608066235	0.6292544322502469	0.6583909594739017
0.4960431913385548	0.4740171268806875	0.7085216105072909
0.3921545483503300	0.6614654205343804	0.7403755143403028
0.4329307349869791	0.6157843605048285	0.7435093449254847
0.6093156858496324	0.5607782030774012	0.7562218340721094
0.6587939512996773	0.5967332292220260	0.7594022796611235
0.5023018670403742	0.5278351310888766	0.7092211341391689
0.6389929769418051	0.6339678412007277	0.6774350223950175
0.5887289257443625	0.5990075246548965	0.6747412858988812
0.5649283816590510	0.3861841114701759	0.7451976971226174
0.6061233607435043	0.3407384305100045	0.7427132790736026
0.3612244208100847	0.3679577753001544	0.6710269930097144
0.6351530087596972	0.3733475640089064	0.6612763565655682
0.5947901196893937	0.4194268843794912	0.6641998030250424
0.4112091503133374	0.4035628846898565	0.6700794698522750
0.1890861516294637	0.2822725589815719	0.7168640482271122
0.7566752991792733	0.2232788192971119	0.6936213796307785
0.6940690446008031	0.6368193838312349	0.6999403693167419
0.7402341554808582	0.6677572370551680	0.7779294480392811
0.7937336443923275	0.6977182643646994	0.7719162090931231
0.8095087709686112	0.7195227017205276	0.7253513025791567
0.7719483032602611	0.7106191099689910	0.6850570034890344
0.7194891408342056	0.6802874270735871	0.6909079946713782
0.6587680323344931	0.3244942093999194	0.7205953805927006
0.6595493099074391	0.2461048396737702	0.6616231992761802
0.7059727191540640	0.2061182269019838	0.6678585642835652
0.7133273353383247	0.3190714366235333	0.7086751212193270
0.7601976689310969	0.2799807436082098	0.7137936890423240
0.3061517977098732	0.3619755534926495	0.6922700248986430
0.2510791822230550	0.3675911412488705	0.7035261023501033
0.1973917669933407	0.3388945039380246	0.6974882131691746
0.2882895868462417	0.2845243084474234	0.7500602617593582
0.2349210388064787	0.2547128445461392	0.7426931613436996
0.3402246957464593	0.6778475311319357	0.7176532148486459
0.2858118872011397	0.6836293201107654	0.7057136577879864
0.2389675411104431	0.7228087553321620	0.7104391416302962
0.3399022218183073	0.7561383665948594	0.6584145299024587
0.2934725582334149	0.7962242919010708	0.6642307766054937
0.2426238368401291	0.7793321908525392	0.6899020594565702
0.6287503778514127	0.5202651118095739	0.7135081608173997
0.3380955467814384	0.4034643469628235	0.7529223907441507
0.3764814596867813	0.4555890404182681	0.8184955721399783
0.4117657920781590	0.4106931607898448	0.8654212342778108
0.6287157759995121	0.4778777606288726	0.8201114276794985
0.4767186673323489	0.3710142119780371	0.8192111051846115
0.5431894597514902	0.3758872550767807	0.8168811890147910
0.3700565322198405	0.5218258149958587	0.8152611110681035
0.4556374658338526	0.6238966689475272	0.8186831257367186
0.5218495009454964	0.6286020103021662	0.8242242673336856
0.3959845065120904	0.5669014557093373	0.7706272194961620
0.3504739223569899	0.6072281711542019	0.7644909819651897
0.5030775984859400	0.4730005347339651	0.8198394471466542
0.3605048458554241	0.6362768630886849	0.8501406253792483
0.4064200920581142	0.5963318620096899	0.8559014453482806
0.5875842552215668	0.5866719245084682	0.8704499971881586
0.6310957482364675	0.6299700088419133	0.8728465345492441
0.4955900200268800	0.5266758485640914	0.8205380243073536
0.6204535483162861	0.6467620925079446	0.7850967411717090
0.5758409274517410	0.6049615008374648	0.7831571277728470
0.5908831014548521	0.4026806952655502	0.8575457806476124
0.6370430131982017	0.3628788598597967	0.8524709397707907
0.3788034559314485	0.3532505929360537	0.7787727205021397
0.6504610518586571	0.3937954895180413	0.7676183887781279
0.6046392463416329	0.4339450580058590	0.7730582280387208
0.4224190435254725	0.3962591502474102	0.7775310206375576
0.2289932168104813	0.2341345417830377	0.8280883518701885
0.7904511306383244	0.2568953209776160	0.8029059235591834
0.6634238240444660	0.6694340757100882	0.8088413231502440
0.7057476408408161	0.7116180899099938	0.8864513410724106
0.7489458639318834	0.7552362226615302	0.8791756838368844
0.7615958472533992	0.7747464022181599	0.8312482176907616
0.7316688908801688	0.7500444415306015	0.7910754980356133
0.6898232937519628	0.7070294519687075	0.7981421394365233
0.6836243956191255	0.3464353836632467	0.8265208017519998
0.6968551622200576	0.2760462897535692	0.7629000070443847
0.7454379933871106	0.2392421309476779	0.7707830514086307

0.7380404695235249	0.3475375361473003	0.8195695319621338
0.7865631831601709	0.3112388503512303	0.8271273115630767
0.3360607819536162	0.3287453709075536	0.8028813037203717
0.2856699366763936	0.3187021889946328	0.8018329260059560
0.2376337588702204	0.2817917564278394	0.7960567125609505
0.3171087446509712	0.2623898145318088	0.8729955274090869
0.2690705292368948	0.2240008808242650	0.8664930404574221
0.3154479651644239	0.6535990023524426	0.8234641068676095
0.2616333603411280	0.6531888867610699	0.8171784596093801
0.2135889356854095	0.6898534662768808	0.8252806380988540
0.3022268572725175	0.7240932772064078	0.7596812025412717
0.2540198673584117	0.7612181983080952	0.7680418752124070
0.2095797495403177	0.7441144593845974	0.8009276827990833
0.6221598032632690	0.5439704953980370	0.8247716737398837
0.3695176987754996	0.3662395371358903	0.8671029391964956
0.2787063130745648	0.5489350407316377	0.1905141245335812
0.5398887520536693	0.7232288877593233	0.1711658858120496
0.5285375984917402	0.4578963820563384	0.1817350488164978
0.4566539778307871	0.4729698584182795	0.1791042790067019
0.5460901536960300	0.5273080288872749	0.1806044480224307
0.4741634322620064	0.5423291862731384	0.1790064690962040
0.7230134971477594	0.4504927430352335	0.1998666596039416
0.4600737921669152	0.2770997095027213	0.1766606451504028
0.2736351553469885	0.5102019137200779	0.2951249648553247
0.4994497491935107	0.7272679108144235	0.2820613119756895
0.5360531591493823	0.4639485388635204	0.2926328240238474
0.4634046930642409	0.4633185314020580	0.2902889518370109
0.5384906917645029	0.5364295012521628	0.2915871590721615
0.4658709095959521	0.5358574428493389	0.2893037554571416
0.7277199591650118	0.4892445848961454	0.3045943038925090
0.5046586850459888	0.2722843292463775	0.2871691030504153
0.2738023599840291	0.4705985982520489	0.3990365313752305
0.4593722824764644	0.7235063922628109	0.3869754295240965
0.5403995766008823	0.4701598918532439	0.3985054900154958
0.4685973206829138	0.4578800135091302	0.3968388200923987
0.5310881137210504	0.5419530797780535	0.3977407667454220
0.4592559987706182	0.5296729187248275	0.3959411309848430
0.7258288002574841	0.5260342545379697	0.4081755280070802
0.5410058538341396	0.2762841559505125	0.3913928513256160
0.2818661245798141	0.4345934341484551	0.5032661212717132
0.4204227248471083	0.7134445803590447	0.4909914792390963
0.5450449032732011	0.4781078161000611	0.5008299729914131
0.4764493205216979	0.4535218665748367	0.4990067190603635
0.5234874332445570	0.5473237140745979	0.5000534650879842
0.4549021198893102	0.5227092241143021	0.4983206154523317
0.7180424566044588	0.5649025676469620	0.5114182953095012
0.5793965614248605	0.2871512801838548	0.4951123743874549
0.2958765892307326	0.3978390069499408	0.6073821037339876
0.3846792855484584	0.6972439122161024	0.5949618939451606
0.5471032519837365	0.4862866576033633	0.6050399812986574
0.4841386246044371	0.4501977846498180	0.6032562415947875
0.5137782579166528	0.5508595688729123	0.6042901900759233
0.4507839792432217	0.5147864032635324	0.6026731745228187
0.7025597708181308	0.6013944835934818	0.6149593534073432
0.6146014398229783	0.3044489492520185	0.5986167531709172
0.3173795665510634	0.3637275382488277	0.7112945216033858
0.3515454585135748	0.6736410723625952	0.6989752078893491
0.5491779138888425	0.4956940876181109	0.7100407731402600
0.4934777761199352	0.4488208485672755	0.7082869659702410
0.5049025117306282	0.5530290869370744	0.7092760989499263
0.4492225620549246	0.5061765067329049	0.7078700579726039
0.6816249558704498	0.6367321064614657	0.7184738849512153
0.6477022852474725	0.3288525567088809	0.7018287867898599
0.3454982290415027	0.3333106299545854	0.8221469783535055
0.3258721494437216	0.6472378707603493	0.8050229764681074
0.5493603424298924	0.5035280944592034	0.8213795902271377
0.5045363651264886	0.4477130386939498	0.8195676099748628
0.4941209153480364	0.5519617136135178	0.8210056763648643
0.4493159329399700	0.4961406739442091	0.8187516313838433
0.6547350564623303	0.6644850887912838	0.8282796379045189
0.6734228004407786	0.3529398444779241	0.8080239037266791
0.7328152604400604	0.4602877865609320	0.2462125421843323
0.4407402977818692	0.2672754128408115	0.1325322695687643
0.2660038198588352	0.5349356907888037	0.2356211743471228
0.5501294373490879	0.7327413126681032	0.1248978305176292
0.7381411592113416	0.5007003174283954	0.3504651175385980
0.4849780230917835	0.2618612880716866	0.2431801720063315

0.2619220454896850	0.4903960535870199	0.3388069376634815
0.5202784706772707	0.7372539374612993	0.2383502731345362
0.7343880311251783	0.5406313008494512	0.4536840593887118
0.5246810287038843	0.2626832923375362	0.3471244268154318
0.2652096575010017	0.4503477434621881	0.4430786129662628
0.4760473923743713	0.7364881279752926	0.3426418174516132
0.7237483307828201	0.5804769323602107	0.5570180468517969
0.5647826614927954	0.2699989526392155	0.4512586177916256
0.2768362232119020	0.4140801240887159	0.5476121742272267
0.4354165255870556	0.7298476798761742	0.4470287419095671
0.7050519119899388	0.6192375689763899	0.6601848915476713
0.6029193467443678	0.2855014896354458	0.5546817455562110
0.2955324040827803	0.3750718901272238	0.6511468004702317
0.3959271955901481	0.7154820383119316	0.5507261031454649
0.6811546357966677	0.6548672101389726	0.7636890778568479
0.6394437746109848	0.3081880440097923	0.6578844212207855
0.3200971653081556	0.3413395126225008	0.7552084913514218
0.3601445123627081	0.6943440557704721	0.6550778111895631
0.6612247177053103	0.6754976272586891	0.8747112686785449
0.6704634030878829	0.3356964658589904	0.7625098508594207
0.3458865608499928	0.3157282581382413	0.8675464942359608
0.3289706973350397	0.6648427840540790	0.7595968019868593
